# Supplementary material for: Modeling Heterogeneity of Triple‐Negative Breast Cancer Uncovers a Novel Combinatorial Treatment Overcoming Primary Drug Resistance
Source: Adv Sci (Weinh). 2020 Dec 16;8(3):2003049. doi: 10.1002/advs.202003049 (PMC7856896; doi:10.1002/advs.202003049)

© 2020 Wiley-VCH GmbH

ADVANCED SCIENCE
Open Access

Supporting Information

for *Adv. Sci.,* DOI: 10.1002/advs.202003049

Modeling Heterogeneity of Triple-Negative Breast Cancer Uncovers a Novel Combinatorial Treatment Overcoming Primary Drug Resistance

Fabienne Lamballe, Fahmida Ahmad, Yaron Vinik, Olivier Castellanet, Fabrice Daian, Anna-Katharina Müller, Ulrike A. Köhler, Anne-Laure Bailly, Emmanuelle Josselin, Rémy Castellano, Christelle Cayrou, Emmanuelle Charafe-Jauffret, Gordon B. Mills, Vincent Géli, Jean-Paul Borg, Sima Lev, and Flavio Maina**

Supporting Information

Modeling Heterogeneity of Triple-Negative Breast Cancer Uncovers a Novel Combinatorial Treatment Overcoming Primary Drug Resistance

Fabienne Lamballe[a,*], Fahmida Ahmad[a], Yaron Vinik, Olivier Castellanet, Fabrice Daian, Anna-Katharina Müller, Ulrike A. Köhler, Anne-Laure Bailly, Emmanuelle Josselin, Rémy Castellano, Christelle Cayrou, Emmanuelle Charafe-Jauffret, Gordon B. Mills, Vincent Géli, Jean-Paul Borg, Sima Lev[b], Flavio Maina[b,*]

Supplementary Experimental Section

Ethics statement: All procedures involving the use of animals were carried out in accordance with the European Community Council Directive of 22 September 2010 on the protection of animals used for experimental purposes (2010/63/EU). The experimental protocols were performed according to the institutional Ethical Committee guidelines for animal research (Comité d'éthique pour l'expérimentation animale – Comité d'éthique de Marseille) and in compliance with the French law under the agreement number D13-055-21, delivered by the "Préfecture de la Région Provence-Alpes-Côte-d'Azur et des Bouches-du-Rhône". Mice were housed under pathogen-free conditions in enriched cages, with a light/dark cycle, and fed ad libitum according to Safe Complete Care Competence (SAFE A04). The mouse project authorization of the Maina laboratory is: APAFIS #8214-2016121417291352.v5, delivered by the "Ministère de l'Enseignement Supérieur, de la Recherche et de l'Innovation". Orthotopic experiments were approved by animal ethics committees (APAFIS#13349-2018013116278149 v2).

Xenograft and orthotopic studies: Eight week-old nude mice (Rj:NMRI-Foxn1 nu/nu; JanvierLabs) were used for xenograft studies. Healthy 8-week-old female NOD.Cg-Prkdc ^{scid}/J (NSG) mice, used for orthotopic studies, were bred in the animal facility of the Cancer Research Center of Marseille (CRCM) and maintained under specific pathogen-free conditions with sterilized food, water provided ad libitum and on a 12-h light and 12-h dark cycle.

In vivo bioluminescence imaging: Imaging was conducted as previously described[1]. Briefly, anesthetized *MMTV-R26*$^{Met\text{-}Luc}$ mice were injected intraperitonealy with luciferin (3 mg/mouse). Analysis of the luminescent images was performed with a NightOwl apparatus, using Berthold Technologies software.

Immunohistochemistry: For histopathological analysis, control mammary glands or dissected tumors were fixed for 4hrs in 4% paraformaldehyde (PFA, Sigma), processed for paraffin embedding, and sectioned. They were then either stained with hematoxylin, and counterstained with eosin (H&E) or subjected to immunostaining with antibodies directed against estrogen receptor (ER), progesterone receptor (PR), human EGFR2 (HER2), or Ki67 (antibodies, and dilutions used are listed in Table S12).

Human cell lines: Human TNBC (MDA-MB-231, MDA-MB-468, SUM-159, Hs578t, HCC-1937 and BT-549) and non-TNBC (MCF-7, SKBR-3, and BT-474) cell lines were obtained from the American Type Culture Collection (ATCC) without further authentication. All cells were tested by PCR-based assay to verify that they were free of *Mycoplasma* contamination. All human breast cancer cells were grown in DMEM/F12 medium supplemented with P/S, glutamine (2mM), sodium pyruvate (1mM, ThermoFisher Scientific), non-essential amino acids (ThermoFisher Scientific), and insulin (10µg/ml; Sigma).

$MGT11^{Luc}$ cells were generated by lentivirus infection of the parental *MMTV-R26*^{Met} MGT11 cells. Briefly, the pHAGE PGK-GFP-IRES-LUC-W lentivirus construct, which allows expression of luciferase and GFP in the infected cells, was used to produce the lentivirus and perform infection. Infected cells were grown in complete DMEM/F12 medium. GFP-positive cells were then sorted by FACS, amplified, and tested for their bioluminescence.

Drugs: Drug concentration and sources are reported in Table S13. Calculation of the Synergy maps and Bliss score has been done using online SynergyFinder tool v1.0[2] using "Viability" parameter as readout and "Bliss Method" with correction activated.

Synergy or additive effects of drug combinations were measured by employing the CompuSyn software v1.0 using the Chou-Talalay equation. Combination index CI<1 indicates synergism, CI<0.5 indicates strong synergism, CI=1 means additive effect and CI>1 stands for antagonism.

Cell viability assay: MMTV-R26^{Met} MGT and human breast cancer cells were plated in 96-well plates at 10,000 cells per well (150µl media/well). After 24hrs, cells were treated with single or combined drugs at the indicated concentrations (Table S13) and cell viability was assessed 48hrs later using the Cell Titer Glo Luminescent Assay (Promega) by detecting the luminescent signals with a luminometer microplate reader (Berthold). Concerning cell death rescue experiments, *MMTV-R26*^{Met} MGT and MDA-MB-231 cells were pre-treated for 1hr with inhibitors of apoptosis (Z-VAD-FMK) or ferroptosis (Ferrostatin-1) at the indicated concentrations, then for additional 24hrs in the absence or presence of A-1155463 (0.3µM) and Adavosertib (3µM). Effects of cell death inhibitors on specific death inducers were assessed as positive control. Data are mean values of at least three independent experiments done in triplicates.

Cell cycle analysis by flow cytometry: MMTV-R26^{Met} MGT cells were cultured in standard conditions before cell cycle analysis of non-treated cells. Regarding cell cycle analysis after

drug treatment, cells were treated with vehicle, A1155463 (1µM), Adavosertib (3µM), alone or in combination for 12hrs. After trypsinisation, cells were resuspended at 10^6 cells per condition. Single cell suspensions were first incubated with the antibodies indicated in Table S12 and Fixable Viability Dye eF506 (1:1000, eBioscience) in PBS-0.5% BSA for 20 min at 4°C. Cells were then fixed and permeabilized using eBioscience™ Foxp3 / Transcription Factor Staining Buffer Set, according to manufacturer's instructions. After centrifugation, cells were resuspended in permeabilization solution containing anti-Ki67antibody, propidium iodide (PI, 40µg/ml, ThermoFisher Scientific) supplemented with RNaseA (50µg/ml, ThermoFisher Scientific). Cells were incubated 20 minutes at room temperature, washed and rapidly analyzed by a BD LSR-II FACS (lasers 405, 488, 561, and 633nm). Cell cycle distribution was analyzed with the BD-Diva version 8.0.1 software. Three independent experiments were performed.

Apoptosis assay by flow cytometry: Quantification of apoptosis was assessed by Annexin V-PI staining, using the eBioscience™ Annexin V Apoptosis Detection Kit FITC, according to the manufacturer's instructions. Briefly, harvested cells were washed in cold PBS and incubated for 15 minutes at room temperature with Annexin V-FITC / PI (1µg/ml) solution prepared in binding buffer. Samples were rapidly analyzed by a BD LSR-II FACS (lasers 405, 488, 561, and 633nm). Results were analyzed with BD-Diva version 8.0.1 software. Four independent experiments were performed.

Cell migration assay: MGT cells (10^4) were seeded in the upper compartment of an 8µm-pore Boyden-like chamber (Transwell, Corning) in 200µl of DMEM/F12 complete medium supplemented with 0.5%FBS. To create a chemo-attractant gradient, the bottom chambers were filled with 700µl of complete DMEM/F12 medium containing 10%FBS, 20ng/ml EGF, 10ng/ml HGF. Media also contained Cytosine arabinoside (AraC, 10µM) to prevent cell proliferation. After 36hrs of incubation at 37°C, non-migrating cells present on the upper surface of the filter were removed with a cotton swab. The invading cells located on the underside were fixed with 4% PFA, and nuclei were stained with DAPI. The number of migrating cells was directly analyzed under a Zeiss Axiophot fluorescent microscope, and determined in at least five fields of each filter. Experiments were performed in triplicate in three independent experiments.

Tumor spheroid forming assay: Cells were seeded in 35mm low attachment plates at a density of 25,000 cells/dish in DMEM/F12(1:1) medium supplemented with P/S (50mg/ml), glutamine

(2mM), bovine serum albumin (BSA, 0.01%, Sigma), B27 without vitamin A (2%, ThermoFisher Scientific), N2 supplement (1%, ThermoFisher Scientific), insulin (10µg/ml), hydrocortisone (0.5 µg/ml), EGF (20ng/ml), HGF (10 ng/ml), and bFGF (10ng/ml, ThermoFisher Scientific). Medium was changed every 2 days. After 10 days, primary spheres were dissociated into single cells and re-plated at the same density as previously described. Subspheroid forming assay (also called passage) was repeated every 10 days. Brightfield pictures of the whole dishes were acquired using an inverted Zeiss Axiophot microscope. Number and size of tumor spheroids were determined using the ImageJ software. Values are expressed as mean ± SEM of three independent experiments.

β-Gal and immunofluorescence staining on MGT cells: For β-Gal staining, cells cultured on glass coverslips were fixed for 2 min in PBS, 25% Gluteraldehyde, 37% formaldehyde, then washed with PBS, and incubated with the staining solution (1mg/ml X-Gal, 0.5mM K3K4, 2mM MgCl2 in PBS) for 4hrs at 37°C. Cells were then fixed with 4% PFA, counterstained with eosin, and mounted on glass microscopic slides with Eukitt hardening solution.

For immunofluorescence staining, cells cultured on coverslips and fixed with 4% PFA were permeabilized for 15 min in PBS/TritonX-100 (PBST) solution (see TritonX-100 concentrations in Table S12), incubated for 1hr in blocking solution (PBST, 10% BSA), then overnight at 4°C with primary antibodies (MET, $pY_{1234/1235}$MET, Ki67) diluted in the above blocking solution. The next day, cells were washed 4 times with PBST, subsequently incubated for 1hr with fluorescence-labelled secondary antibodies (Table S12), incubated for 15 min in presence of DAPI (1:1000), then mounted on glass microscopic slides with Prolong Gold antifade reagent (ThermoFischer Scientific). Images were taken with a Zeiss Axiophot fluorescent microscope.

In vivo tumorigenesis assays: Xenografts. To assess the in vivo tumorigenicity of the *MMTV-R26^Met^* MGT cell lines, cells ($5x10^6$) were resuspended in 200µl of a PBS:Matrigel (1:1) solution (CorningBV), and injected subcutaneously into both flanks of nude mice (n=4 mice / cell line). Animals were sacrificed when the tumor volume reached a maximum of 2000 mm^3. Tumor volume, measured with a caliper, was determined by the formula: $(L \times W^2)/2$. L: length; W: width.

Orthotopic injections. To evaluate the in vivo efficacy of the drug combination, $MGT11^{Luc}$ ($1x10^5$) cells, suspended in 50% phenol red–free Matrigel (Becton Dickinson Bioscience), were grafted into the fourth mammary fat pad of NSG mice (TrGET Preclinical Platform). After

seven days of graft, mice were randomly divided into two groups (n=8 per group). Starting from the eighth day post-graft, control vehicle (2% DMSO, 30% PEG300, 5% Tween-80) or drug combination were administered (A1155463, 5mg/Kg, intraperitoneally; Adavosertib, 60mg/Kg, *per os*) for a total of 29 days. Mice body weight was checked daily, before drug administration. Tumor volume was measured every three days and calculated with the formula: (3.14 x Length x Width x Height)/6. The experiment was terminated when tumor volume reached a maximum of 1500mm^3. After completion of the study, lung luminescence was assessed following addition of endotoxin-free luciferin (30 mg/kg) and autopsy of mice. Bioluminescence analysis was performed using Optima-PhotonIMAGER (Biospace Lab).

Quantitative RT-PCR analysis: Total RNA was extracted from tissues or cells using the RNeasy Mini Kit (Qiagen) as previously described[3]. DNase (RNase-free DNAse I Set, Qiagen) treatment was included to avoid possible genomic DNA contamination. cDNA was synthesized using a Reverse Transcription Kit (iScript Reverse Transcription Supermix, Bio-Rad). Real-time PCR reactions were performed in a qPCR CFX 96 apparatus (Bio-Rad), using the SYBR® Green detection method (SYBR GreenER qPCR SuperMix, ThermoFisher Scientific), and specific primers (0.1µM; primer sequences are listed in Table S14). mRNA expression levels were normalized to the *Beta-2-microglobuline* (*B2M*) housekeeping gene, and analyzed using the $2^{-\Delta\Delta Ct}$ method. All reactions were run in triplicate and repeated in three independent experiments.

Western blotting: Protein extracts were biochemically analyzed as previously described[4]. Briefly, *MMTV-R26^{Met}* MGT cells were lysed in EMB lysis buffer (1% Triton X-100, 50mM HEPES, 1mM EGTA, 150mM NaCl, 1.5mM MgCl2, 10% glycerol, 10mM NaF, 1mM NaPP, 1mM Na_3VO_4, 10mM β-glycerophosphate, 5µg/ml leupeptin, 5 µM pepstatin A, 2µg/ml aprotinin, 5mM benzamidin, 1mM phenylmethylsulfonyl fluoride (PMSF), and centrifuged at 14,000 rpm for 15 min at 4 °C. Protein concentration was measured by Bradford assay (Bio-Rad), and equal amounts of proteins were separated on 10% polyacrylamide gels or SDS-PAGE (for phospho-ATM).

For nuclear and chromatin extracts, cells were pelleted, washed in PBS, swollen in hypotonic buffer (20mM HEPES pH 7.9, 1.5mM MgCl2, 10mM KCl, 1mM DTT, 1mM PMSF, 1x protease Inhibitor cocktail (Sigma)) on ice, then centrifuged at 1500rpm for 5min. Nuclei were resuspended in nuclear extract low salt buffer (20mM HEPES pH 7.9, 350mM sucrose, 1.5mM MgCl2, 150mM KCl, 0.5mM EDTA, 0.2% NP-40, 1mM PMSF, 1x protease Inhibitor cocktail)

for 30 min on ice. The nucleoplasmic extract was recovered in the supernatant after centrifugation at 3000rpm for 10min. Residual nuclei were then resuspended in nuclear extract buffer (20mM HEPES pH 7.9, 350mM sucrose, 1.5mM $MgCl_2$, 300mM KCl, 0.5mM EDTA, 0.2% NP-40, 1mM PMSF, 1x protease Inhibitor cocktail). After centrifugation at 3000rpm for 10min, the nuclear extract (supernatant) was separated from the chromatin (pellet). DNA from pellet was digested with Universal Nuclease (PierceTM) in nuclear extract high salt buffer (20mM HEPES pH 7.9, 350mM sucrose, 1.5mM $MgCl_2$, 600mM KCl, 0.5mM EDTA, 0.2% NP-40, 1mM PMSF, 1x protease Inhibitor cocktail) for 30 min on ice. Protein concentration was measured by Qubit Protein Assay Kit (ThermoFisher) and equal amounts of proteins were separated on 15% polyacrylamide gels.

Membranes were incubated in blocking buffer (5% milk, PBS, 0.1% Tween-20, 50mM NaF), then analyzed using standard procedures. PageRuler Plus Prestained protein ladder (ThermoFisher Scientific) was used as a molecular weight marker. Pictures of the membranes stained with Ponceau (Sigma) and immunoblotted were taken through BioRad ChemiDoc imager. Antibodies used are reported in Table S12.

Author contributions

F.L.: designed and supervised studies, performed the majority of the experiments, data analysis, and interpretation; contributed to write the manuscript.

F.A.: performed the majority of the experiments under supervision of F.L., data analysis, and interpretation.

Y.V.: performed computational work with mouse and human TNBC databases, machine learning analyses, and interpretation; provided inputs on studies and on the manuscript.

O.C.: contributed to cell viability and biochemical studies; data processing.

F.D.: contributed to computational work with drug response data on cells, data analysis, and interpretation.

A.K.M.: performed histological analysis on *MMTV-R26^{Met}* samples, data analysis, and interpretation; provided input on the manuscript.

U.A.K.: performed histological analysis on *MMTV-R26^{Met}* samples, data analysis, and interpretation; provided input on the manuscript.

A.L.B.: performed FACS studies, data analysis, and interpretation.

E.J.: performed orthotopic studies and data analysis.

R.C.: performed orthotopic studies and data analysis.

C.C.: performed cell fractionation studies and data analysis.

E.C.J.: supervised and performed histological analysis on *MMTV-R26^{Met}* samples, data analysis, and interpretation; provided input on the manuscript.

G.B.M.: contributed to RPPA studies; provided input on the manuscript.

V.G.: provided input on studies and contributed to write the manuscript.

J.P.B.: provided input on studies and contributed to write the manuscript.

S.L.: designed and supervised studies on histological and RPPA analysis; provided input on studies; contributed to interpret data and to write the manuscript.

F.M.: designed and supervised the study, contributed to experimental work, analyzed and interpreted data, ensured financial support, and wrote the manuscript.

Supplementary Figure Legends

Figure S1. Expression of the wild-type *Met* transgene in the mouse mammary gland leads to tumor formation. a) Western blot analysis of total protein extracts from either mammary gland or liver from adult wild-type (wt), *Alb-R26^{Met}*, *MMTV-R26^{Met}*, or *R26^{stopMet}* mice. The MET^{tg} is specifically expressed in the mammary gland of *MMTV-R26^{Met}* mice. *Alb-R26^{Met}* mice were used as control for expression of MET^{tg} in the liver[1]. Expression levels of ERKs were used as a loading control. b) Kaplan-Meier analysis of mammary gland tumor incidence in *MMTV-ErbB2* mice kept in the same genetic background as the *MMTV-R26^{Met}* (*MMTV-ErbB2^{mix}*; data already shown in Figure 1d), and in FVB/mix background (*MMTV-ErbB2*)[5]. c) Example of *MMTV-R26^{Met}* mammary gland tumor and lung metastasis. Hematoxylin and eosin (H&E) staining of corresponding sections are shown on the bottom. d) Heatmap reporting upregulated (red) and downregulated (blue) signals in *MMTV-R26^{Met}* tumors. e) Graph reporting total *Met* mRNA levels (endogenous plus exogenous, using primers in the *Met* extracellular domain (*Met EXT*)) in *MMTV-cre* (n=5) and *MMTV-R26^{Met}* pre-tumorigenic (n=6) glands, and *MMTV-R26^{Met}* tumors (n=24). f) Random Forest method, a very robust machine learning method for classification problems, was used to build the model predicting TNBC subtypes. TCPA was used as training (80%) and validation (20%) sets. The graph reports the accuracy (repeated cross-validation) of the number of proteins randomly selected at each split of the trees. Based on the high accuracy achieved, this model was used for analysis of the *MMTV-R26^{Met}* tumors.

Figure S2. Biological properties of *MMTV-R26^{Met}* MGT cell lines. a) Efficiency of Cre recombination (β-Gal staining, top images), and immunohistochemical analysis of MET^{tg} (detected by human MET antibodies) and phosphoY$_{1234/1235}$-MET (pMET) in *MMTV-R26^{Met}* cell lines. Lack of β-Gal expression, due to the excision of the LacZ-stop cassette, reveals that Cre recombination has occurred in cells. Percentages of cells with efficient recombination and positive for MET^{tg} or phospho-MET are indicated on the corresponding images. Nuclei stained with DAPI are in blue. Scale bars: 100µm b) Western blot analysis of total protein extracts from MGT2, MGT4, and MGT9 cell lines. The quantity of total protein extracts loaded in each lane is indicated. Ponceau staining was used as a loading control. The MET^{25H2} antibodies recognize both the endogenous MET and the MET^{tg}. c) Comparison of the proteomic profile of in *MMTV-R26^{Met}* MGT9 versus both MGT4 and MGT11. Enrichment analyses were done by using the Enrichr software. Histograms show enriched pathways according to Reactome, Kyoto Encyclopedia of Genes and Genomes (KEGG), and BioCarta databases. In all panels,

enrichments are ranked according to combined scores (values are indicated on the X axis). The 20 top ranked enrichments are presented. Enrichments in Notch and PI3K/AKT/mTOR pathways are indicated by green arrowheads. One-way ANOVA was used to analyze the RPPA outcomes using the Partek Genomic Suite.

Figure S3. Proliferation capacities of *MMTV-R26*^{Met} MGT cell lines. a) Histogram showing the percentage of cells from each cell line in each phase of the cell cycle as determined by flow cytometry using propidium iodide and Ki67 staining. Statistical analyses were performed by two-way ANOVA followed by Tukey test. All statistics are reported in Table S8. b) Representative graphs showing cell cycle distribution as measured by flow cytometry using PI and Ki67 staining. c-d) Quantification (c) and representative images (d) of cell proliferation capacity determined by analysis of the percentage of Ki67-positive cells compared to the total number of cells. The mean percentages are indicated on the corresponding images. Statistical analyses shown here were performed by using MGT2 as the control cell line. For multiple comparisons, statistical significance was assessed by One-way ANOVA followed by Tukey test. All statistical analyses performed by comparing one cell line to another are reported in Table S15. Scale bar: 100µm. e) Western blot analysis of p53 expression levels in *MMTV-R26*^{Met} MGT cell lines compared to normal mammary epithelial cells (control). For all analyses described in this figure, three independent experiments were performed.

Figure S4. Multiple signaling networks are enriched in the *MMTV-R26*^{Met} cell lines. a) Heatmap reporting expression or phosphorylation levels of proteins in the *MMTV-R26*^{Met} cell lines as determined by RPPA. Note the hierarchical clustering of MGT4, MGT9, and MGT11 compared to MGT13, among the tumorigenic cell lines. Red: upregulated; blue: downregulated. b) Proteomic profiles of cells from the different clusters identified by PCA were compared one to another, as indicated. Enrichment analyses were done by using the Enrichr software. Histograms show: i) enriched pathways according to the Kyoto Encyclopedia of Genes and Genomes (KEGG) database; ii) cell signaling pathway enrichment using the WikiPathways database; iii) kinases identified based on their phosphorylation targets (Kinase Enrichment Analysis, KEA database); iv) enrichment according to the Jensen compartments database. In all panels, enrichments are ranked according to combined scores (values are indicated on the X axis). The 20 top ranked enrichments are presented. Note that the majority of the signaling changes are related to signals involved in DNA repair, cell cycle regulation, metabolism, and

stemness. One-way ANOVA was used to analyze the RPPA outcomes using the Partek Genomic Suite.

Figure S5. A1155463 and Adavosertib exhibit synergistic effects on *MMTV-R26^{Met}* MGT cell lines. a-b) Representation of the drug screen outcomes performed on the MGT4 cell line, highlighting single and combined treatments tested. Drugs were used at a concentration of 3µM (a) and 10µM (b). Numbers indicate the percentage of viable cells in the presence of drugs compared to controls (untreated cells), and labelled by the green-to-red color code. Values are expressed as the mean ± s.e.m. Grey squares correspond to untested drug effects. Note that targeting BCL-XL (with Wehi) and WEE1 (with Adavosertib) is highly deleterious (red color) for *MMTV-R26^{Met}* MGT4 cells compared to other drugs exhibiting no or minimal effects (green, yellow, orange). c) Data from cell viability assays of combined drug effects were used by the Compusyn software to simulate combination index (Y-axis) for each affected fraction (X-axis, from 0 to 1). Each black dot corresponds to a tested dose. Based on the combination index scores, combinations of BCL-XL and WEE1 inhibition resulted in strong synergistic interactions for high doses on MGT4, and for all doses tested for MGT9, MGT11, and MGT13. (d-f) MGT11 and MGT11^{Luc} cells exhibit similar biological properties. (d) Viability assay performed on MGT11 and MGT11^{Luc} cells when exposed to A1155463 (A11, 0.3µM) alone or in combination with Adavosertib (Adav, 3µM). Percentage of cell viability in the presence of drugs compared to untreated cells is reported using the defined color code. Values are expressed as the mean ± s.e.m. (e) Graph representing the percentage of cells in each phase of the cell cycle as determined by flow cytometry using PI and Ki67 staining. Statistical analyses were performed by two-way ANOVA followed by Tukey test. No significance in cell cycle distribution was observed between the two cell lines. (f) Tumor sphere assay performed with MGT11 and MGT11^{Luc} cells. Values are expressed as means ± s.e.m. For all these *in vitro* assays, three independent experiments were performed. g) Effect of the combined A1155463+Adavosertib treatment (A11+Adav) on lung metastasis formation monitored using bioluminescence imaging in the treated mice. Quantification of the normalized photon flux in A11+Adav-treated mice compared to the control mice (vehicle). Results show a trend of reduced metastasis formation in the group of mice treated with the drug combination, although not significant. Wilcoxon test was used for statistical analysis. Data represent mean ± SD (n = 8, per group of mice). ns: P>0.05.

Figure S6. Signaling changes in MGT9 and MGT13 cells when exposed to drugs targeting BCL-XL and WEE1. a-b) Western blot analysis of total protein extracts from MGT9 and MGT13 cells treated for 12hrs with either A1155463 (A11, 1µM) or Adavosertib (Adav, 3 µM) alone or in combination. At least two independent experiments were performed.

Figure S7. Kaplan-Meier curve reporting the probability of the overall survival of human TNBC patients according to *MET* levels. *MET* high versus low groups were performed, using as cut-off, the median of *MET* levels expressed as the median-centered and magnitude-normalized data (from NCBI dataset with accession number GSE31519 Affymetrix Human Genome U133A Array). The database includes 580 patients (high *MET*: 380 patients; low *MET*: 200 patients). For 196 patients, no data concerning survival were available (high *MET*: 123 patients; low *MET*: 73 patients).

Supplementary Table Legends

Table S1. Mammary gland (tumors and controls) used for histopathological and RPPA analyses.

Table S2. Antibodies used for RPPA analysis of *MMTV-R26^{Met}* tumors and cells. RPPA analysis on tumors and non-treated MGT cells was performed using the 247 rabbit antibodies. RPPA analysis done on treated MGT cells included mouse and rabbit antibodies (n= 426).

Table S3. Molecular/signaling characteristics of *MMTV-R26^{Met}* tumors identified through RPPA analysis.

Table S4. Molecular/signaling characteristics of *MMTV-R26^{Met}* MGT cells identified through RPPA analysis.

Table S5. Comparison of RPPA outcomes of *MMTV-R26^{Met}* cells and tumors belonging to "subtype A" versus "subtype B".

Table S6. Comparisons of RPPA outcomes of *MMTV-R26^{Met}* cells belonging to "subtype A" and "subtype B" versus those from the non-tumorigenic cells.

Table S7. Changes in protein expression and/or phosphorylation levels occurring in *MMTV-R26^{Met}* MGT4 cells when exposed to single or combined treatment targeting BCL-XL (A1155463) and WEE1 (Adavosertib).

Table S8. Statistical analysis of the MMTV-R26Met MGT cell lines cell cycle distribution.

Table S9. Statistical analysis of the in vitro tumorigenic capacity of the MMTV-R26Met cell lines (determined by the tumor sphere assay).

Table S10. Statistical analysis of the migrating capacity of the MMTV-R26Met cell lines.

Table S11. Statistical analysis of cell cycle distribution of MGT11 cells treated with A1155463, or Adavosertib, alone or in combination.

Table S12. Antibodies used in the study.

Table S13. Drugs used for cell viability assays, with the indicated targets and the concentrations used.

Table S14. Oligonucleotides used for RT-qPCR experiments.

Table S15. Statistical analysis of the proliferation capacity (mitotic index) of the MMTV-R26Met cell lines.

Supplementary References

1 Fan, Y. N. *et al.* A Phosphokinome-Based Screen Uncovers New Drug Synergies for Cancer Driven by Liver-Specific Gain of Nononcogenic Receptor Tyrosine Kinases. *Hepatology* **66**, 1644-1661, doi:10.1002/hep.29304 (2017).

2 Ianevski, A., He, L., Aittokallio, T. & Tang, J. SynergyFinder: a web application for analyzing drug combination dose-response matrix data. *Bioinformatics* **33**, 2413-2415, doi:10.1093/bioinformatics/btx162 (2017).

3 Arechederra, M. *et al.* Hypermethylation of gene body CpG islands predicts high dosage of functional oncogenes in liver cancer. *Nat Commun* **9**, 3164, doi:10.1038/s41467-018-05550-5 (2018).

4 Furlan, A. *et al.* Combined drug action of 2-phenylimidazo[2,1-b]benzothiazole derivatives on cancer cells according to their oncogenic molecular signatures. *PLoS One* **7**, e46738, doi:10.1371/journal.pone.0046738 (2012).

5 Henry, M. D., Triplett, A. A., Oh, K. B., Smith, G. H. & Wagner, K. U. Parity-induced mammary epithelial cells facilitate tumorigenesis in MMTV-neu transgenic mice. *Oncogene* **23**, 6980-6985 (2004).

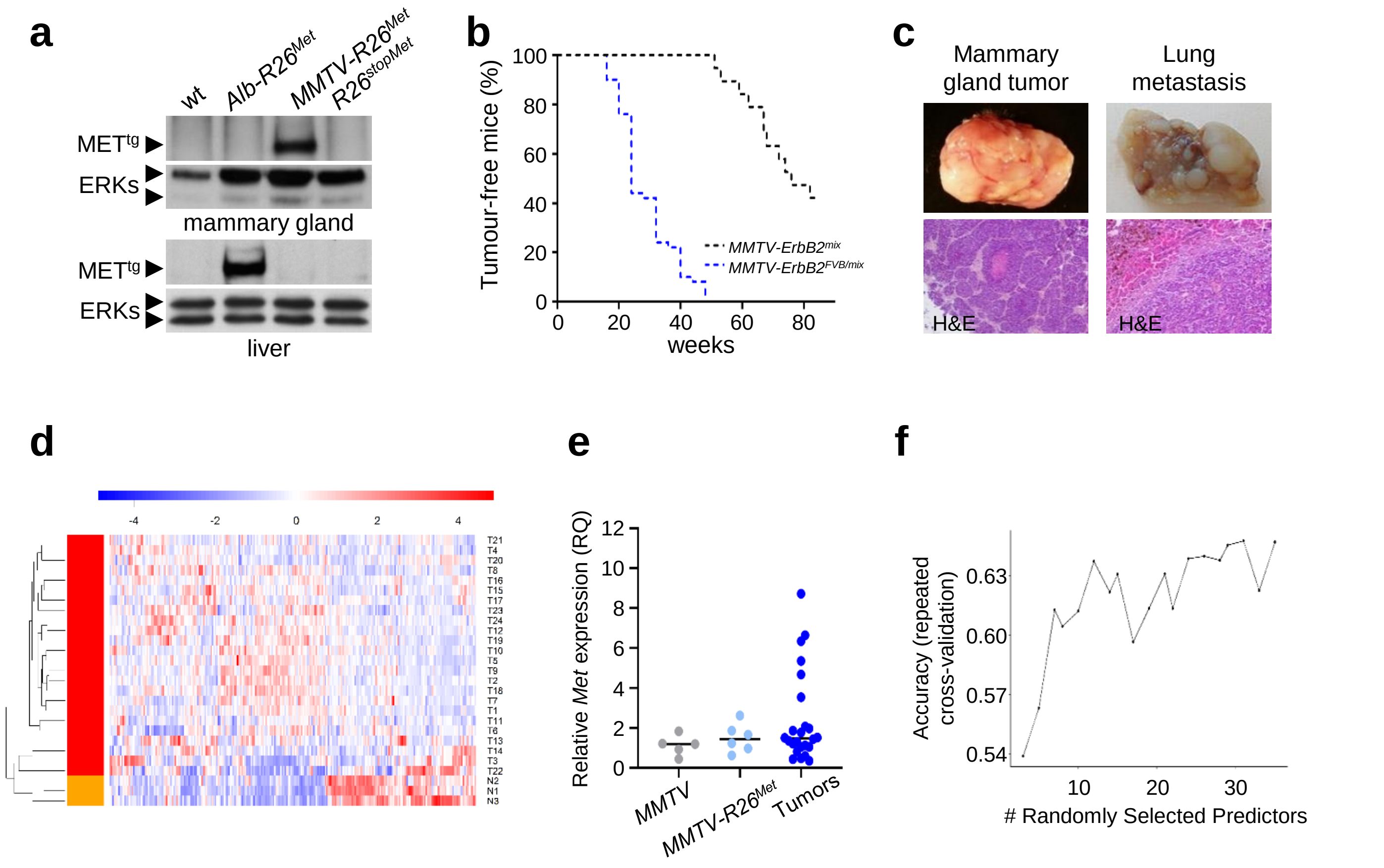
a
wt
Alb-R26Met
MMTV-R26Met
R26stopMet
METtg
ERKs
mammary gland
METtg
ERKs
liver
b
Tumour-free mice (%)
100
80
60
40
20
0
0
20
40
60
80
weeks
MMTV-ErbB2mix
MMTV-ErbB2FVB/mix
c
Mammary gland tumor
Lung metastasis
H&E
H&E
d
-4
-2
0
2
4
T21
T4
T20
T8
T16
T15
T17
T23
T24
T12
T19
T10
T5
T9
T2
T18
T7
T1
T11
T6
T13
T14
T3
T22
N2
N1
N3
e
Relative Met expression (RQ)
12
10
8
6
4
2
0
MMTV
MMTV-R26Met
Tumors
f
Accuracy (repeated cross-validation)
0.63
0.60
0.57
0.54
10
20
30
Randomly Selected Predictors

Figure S1

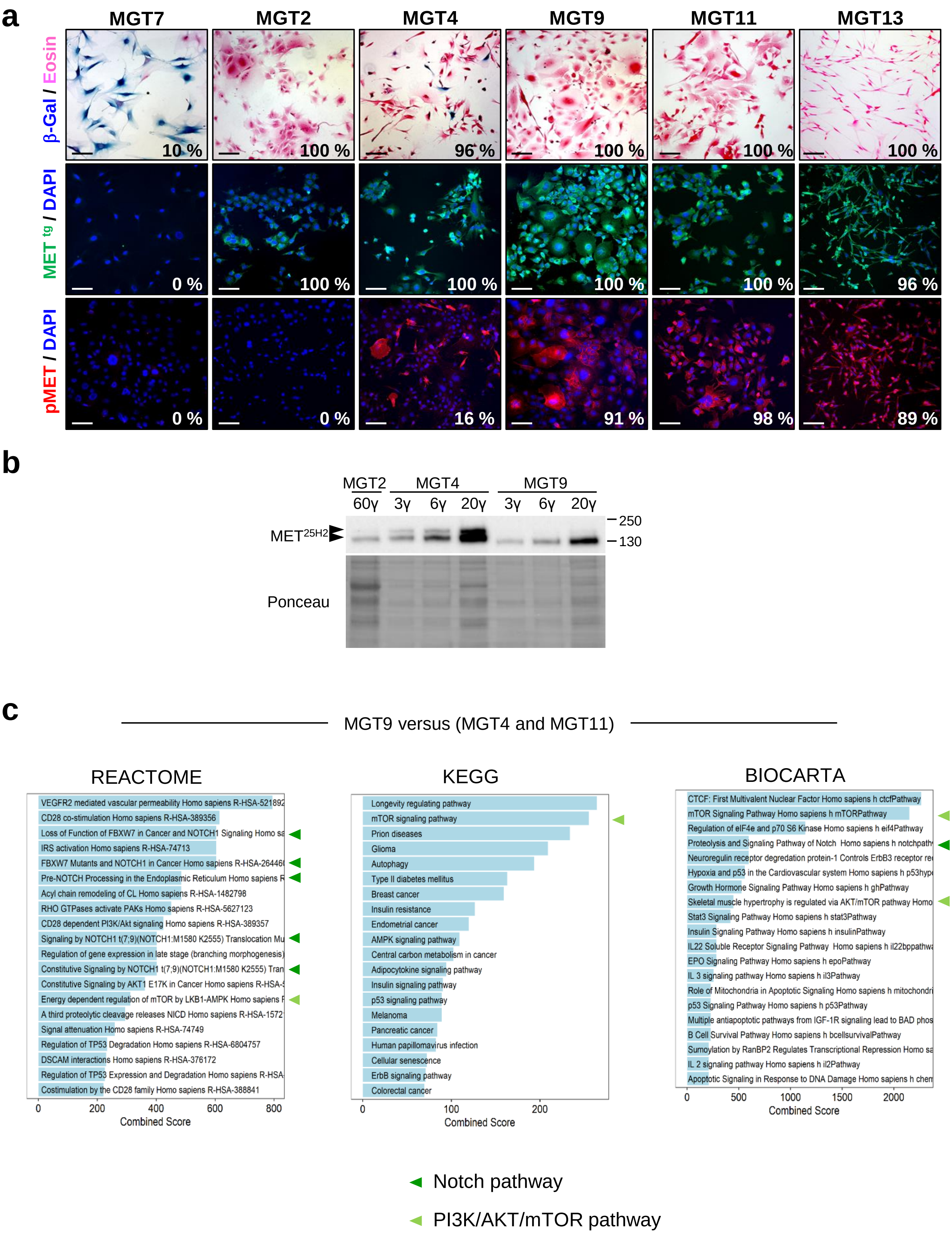
a
MGT7
MGT2
MGT4
MGT9
MGT11
MGT13
β-Gal / Eosin
10 %
100 %
96 %
100 %
100 %
100 %
MET tg / DAPI
0 %
100 %
100 %
100 %
100 %
96 %
pMET / DAPI
0 %
0 %
16 %
91 %
98 %
89 %
b
MGT2
MGT4
MGT9
60y
3y
6y
20y
3y
6y
20y
MET25H2
250
130
Ponceau
c
MGT9 versus (MGT4 and MGT11)
REACTOME
VEGFR2 mediated vascular permeability Homo sapiens R-HSA-521892
CD28 co-stimulation Homo sapiens R-HSA-389356
Loss of Function of FBXW7 in Cancer and NOTCH1 Signaling Homo sa
IRS activation Homo sapiens R-HSA-74713
FBXW7 Mutants and NOTCH1 in Cancer Homo sapiens R-HSA-264460
Pre-NOTCH Processing in the Endoplasmic Reticulum Homo sapiens R
Acyl chain remodeling of CL Homo sapiens R-HSA-1482798
RHO GTPases activate PAKs Homo sapiens R-HSA-5627123
CD28 dependent PI3K/Akt signaling Homo sapiens R-HSA-389357
Signaling by NOTCH1 t(7;9)(NOTCH1:M1580 K2555) Translocation Mu
Regulation of gene expression in late stage (branching morphogenesis)
Constitutive Signaling by NOTCH1 t(7;9)(NOTCH1:M1580 K2555) Tran
Constitutive Signaling by AKT1 E17K in Cancer Homo sapiens R-HSA-
Energy dependent regulation of mTOR by LKB1-AMPK Homo sapiens F
A third proteolytic cleavage releases NICD Homo sapiens R-HSA-1572
Signal attenuation Homo sapiens R-HSA-74749
Regulation of TP53 Degradation Homo sapiens R-HSA-6804757
DSCAM interactions Homo sapiens R-HSA-376172
Regulation of TP53 Expression and Degradation Homo sapiens R-HSA
Costimulation by the CD28 family Homo sapiens R-HSA-388841
0
200
400
600
800
Combined Score
KEGG
Longevity regulating pathway
mTOR signaling pathway
Prion diseases
Glioma
Autophagy
Type II diabetes mellitus
Breast cancer
Insulin resistance
Endometrial cancer
AMPK signaling pathway
Central carbon metabolism in cancer
Adipocytokine signaling pathway
Insulin signaling pathway
p53 signaling pathway
Melanoma
Pancreatic cancer
Human papillomavirus infection
Cellular senescence
ErbB signaling pathway
Colorectal cancer
0
100
200
Combined Score
BIOCARTA
CTCF: First Multivalent Nuclear Factor Homo sapiens h ctcfPathway
mTOR Signaling Pathway Homo sapiens h mTORPathway
Regulation of eIF4e and p70 S6 Kinase Homo sapiens h eif4Pathway
Proteolysis and Signaling Pathway of Notch Homo sapiens h notchpath
Neuroregulin receptor degredation protein-1 Controls ErbB3 receptor re
Hypoxia and p53 in the Cardiovascular system Homo sapiens h p53hyp
Growth Hormone Signaling Pathway Homo sapiens h ghPathway
Skeletal muscle hypertrophy is regulated via AKT/mTOR pathway Homo
Stat3 Signaling Pathway Homo sapiens h stat3Pathway
Insulin Signaling Pathway Homo sapiens h insulinPathway
IL22 Soluble Receptor Signaling Pathway Homo sapiens h il22bppathwa
EPO Signaling Pathway Homo sapiens h epoPathway
IL 3 signaling pathway Homo sapiens h il3Pathway
Role of Mitochondria in Apoptotic Signaling Homo sapiens h mitochondri
p53 Signaling Pathway Homo sapiens h p53Pathway
Multiple antiapoptotic pathways from IGF-1R signaling lead to BAD phos
B Cell Survival Pathway Homo sapiens h bcellsurvivalPathway
Sumoylation by RanBP2 Regulates Transcriptional Repression Homo sa
IL 2 signaling pathway Homo sapiens h il2Pathway
Apoptotic Signaling in Response to DNA Damage Homo sapiens h chem
0
500
1000
1500
2000
Combined Score
Notch pathway
PI3K/AKT/mTOR pathway

Figure S2

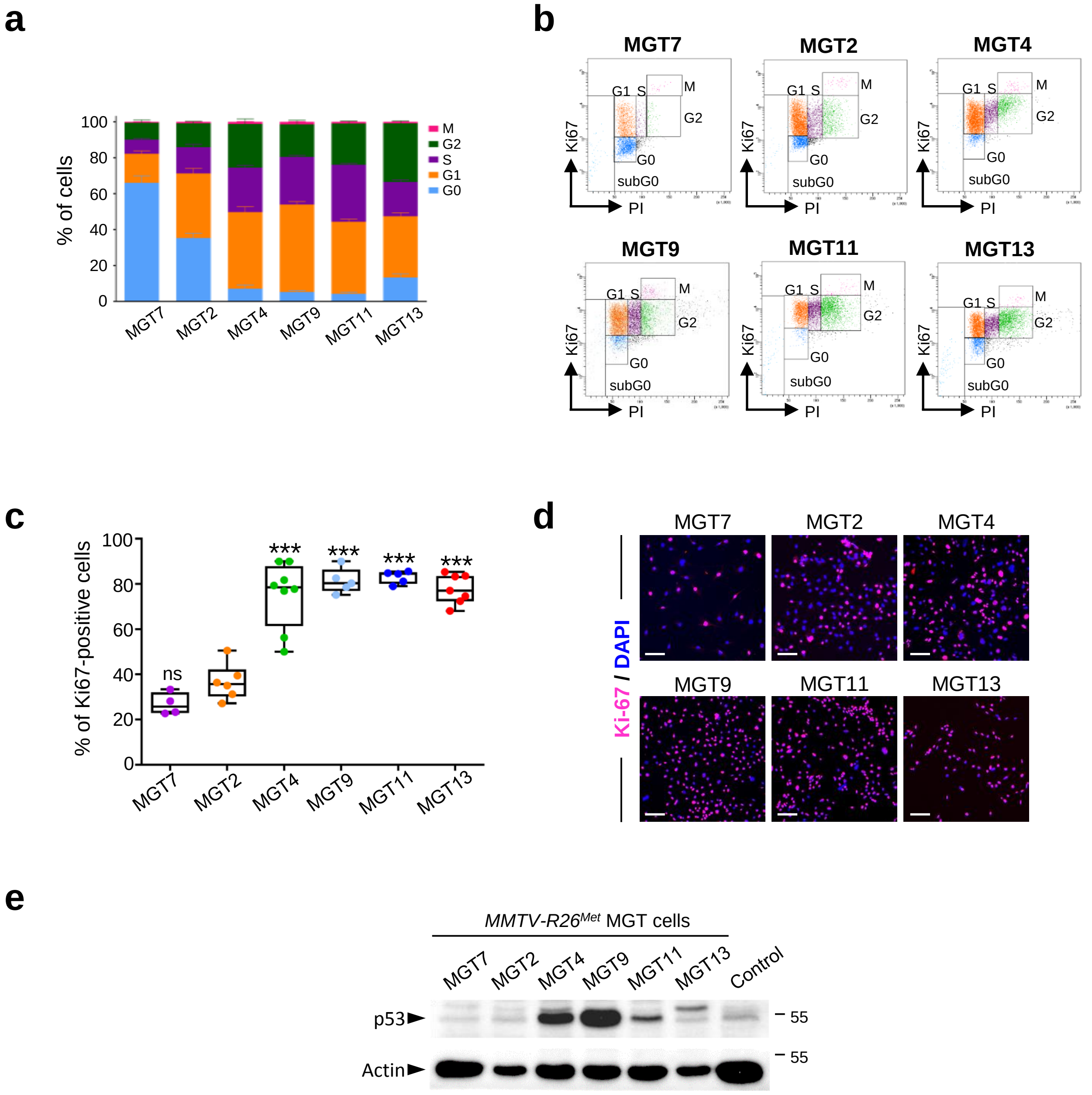
a
% of cells
M
G2
S
G1
G0
MGT7
MGT2
MGT4
MGT9
MGT11
MGT13
b
MGT7
MGT2
MGT4
MGT9
MGT11
MGT13
Ki67
PI
G1
S
M
G2
G0
subG0
c
% of Ki67-positive cells
ns
***
d
Ki-67 / DAPI
e
MMTV-R26^Met MGT cells
Control
p53
Actin
55

Figure S3

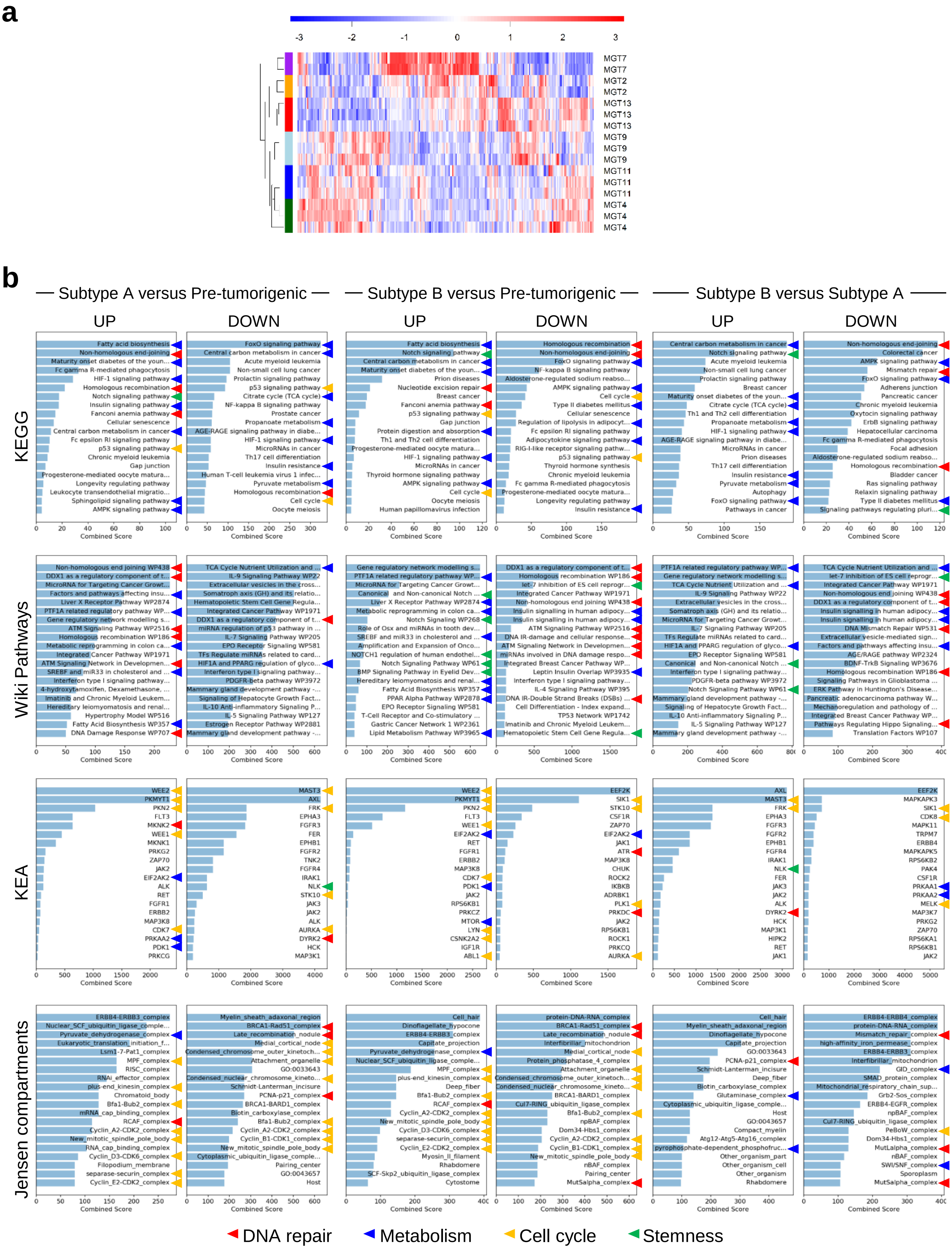
a
-3
-2
-1
0
1
2
3
MGT7
MGT7
MGT2
MGT2
MGT13
MGT13
MGT13
MGT9
MGT9
MGT9
MGT11
MGT11
MGT11
MGT4
MGT4
MGT4
b
Subtype A versus Pre-tumorigenic
Subtype B versus Pre-tumorigenic
Subtype B versus Subtype A
UP
DOWN
KEGG
Wiki Pathways
KEA
Jensen compartments
Combined Score
DNA repair
Metabolism
Cell cycle
Stemness

Figure S4

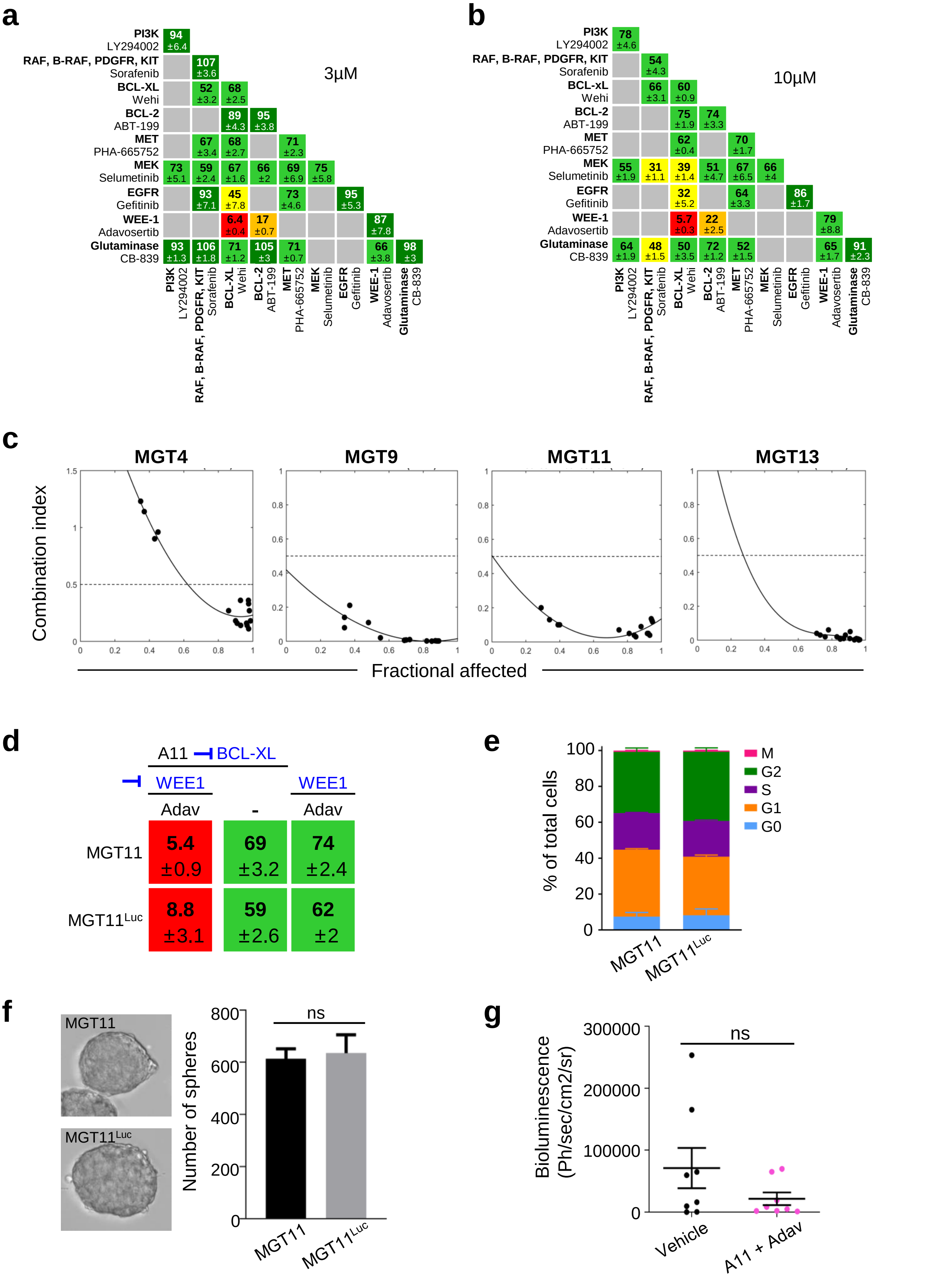
a
3µM
PI3K LY294002
RAF, B-RAF, PDGFR, KIT Sorafenib
BCL-XL Wehi
BCL-2 ABT-199
MET PHA-665752
MEK Selumetinib
EGFR Gefitinib
WEE-1 Adavosertib
Glutaminase CB-839
94 ±6.4
107 ±3.6
52 ±3.2 68 ±2.5
89 ±4.3 95 ±3.8
67 ±3.4 68 ±2.7 71 ±2.3
73 ±5.1 59 ±2.4 67 ±1.6 66 ±2 69 ±6.9 75 ±5.8
93 ±7.1 45 ±7.8 73 ±4.6 95 ±5.3
6.4 ±0.4 17 ±0.7 87 ±7.8
93 ±1.3 106 ±1.8 71 ±1.2 105 ±3 71 ±0.7 66 ±3.8 98 ±3
b
10µM
PI3K LY294002
RAF, B-RAF, PDGFR, KIT Sorafenib
BCL-xL Wehi
BCL-2 ABT-199
MET PHA-665752
MEK Selumetinib
EGFR Gefitinib
WEE-1 Adavosertib
Glutaminase CB-839
78 ±4.6
54 ±4.3
66 ±3.1 60 ±0.9
75 ±1.9 74 ±3.3
62 ±0.4 70 ±1.7
55 ±1.9 31 ±1.1 39 ±1.4 51 ±4.7 67 ±6.5 66 ±4
32 ±5.2 64 ±3.3 86 ±1.7
5.7 ±0.3 22 ±2.5 79 ±8.8
64 ±1.9 48 ±1.5 50 ±3.5 72 ±1.2 52 ±1.5 65 ±1.7 91 ±2.3
c
MGT4
MGT9
MGT11
MGT13
Combination index
Fractional affected
d
A11 BCL-XL
WEE1
WEE1
Adav
-
Adav
MGT11 5.4 ±0.9 69 ±3.2 74 ±2.4
MGT11Luc 8.8 ±3.1 59 ±2.6 62 ±2
e
% of total cells
M
G2
S
G1
G0
MGT11
MGT11Luc
f
MGT11
MGT11Luc
Number of spheres
ns
MGT11
MGT11Luc
g
Bioluminescence (Ph/sec/cm2/sr)
ns
Vehicle
A11 + Adav

Figure S5

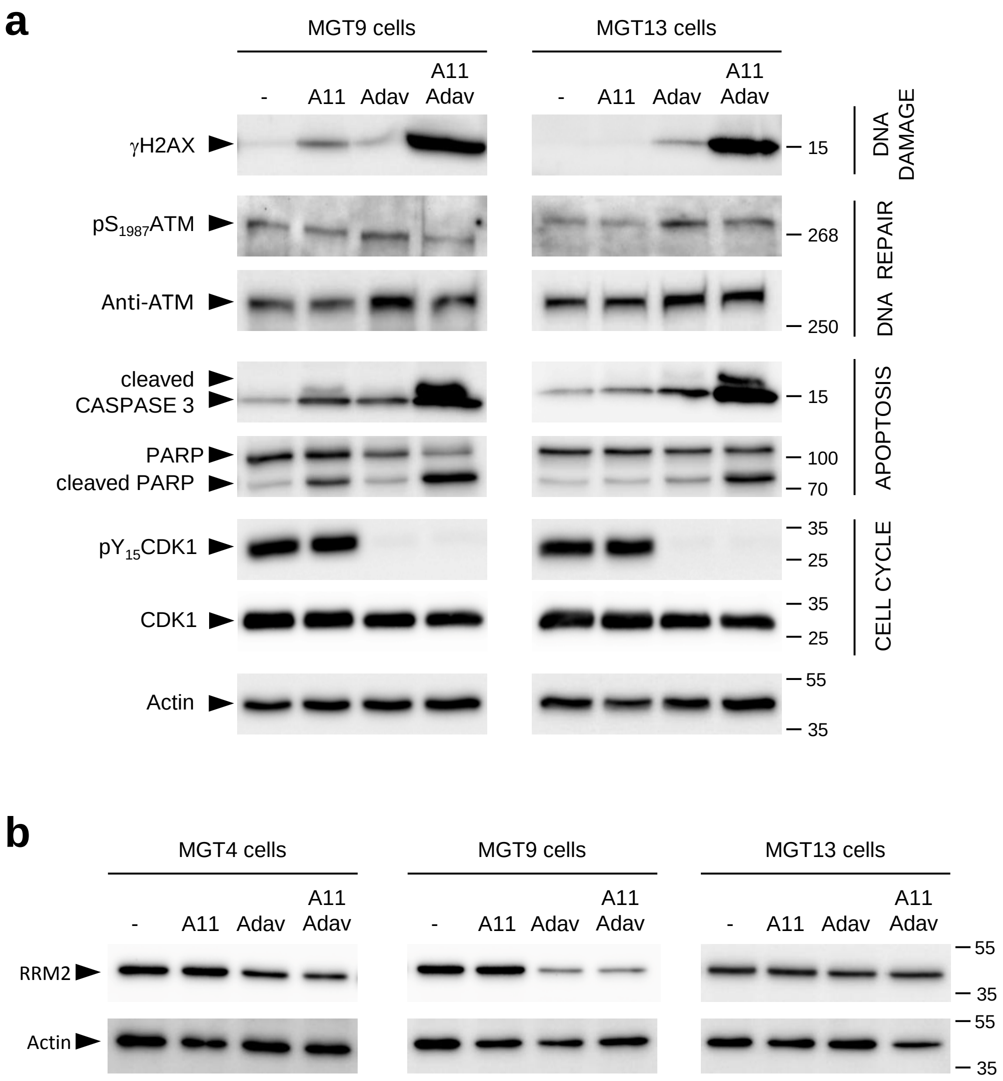
a
MGT9 cells
MGT13 cells
-
A11
Adav
A11 Adav
γH2AX
pS1987ATM
Anti-ATM
cleaved CASPASE 3
PARP
cleaved PARP
pY15CDK1
CDK1
Actin
15
268
250
100
70
35
25
55
DNA DAMAGE
DNA REPAIR
APOPTOSIS
CELL CYCLE
b
MGT4 cells
MGT9 cells
MGT13 cells
RRM2
Actin

Figure S6

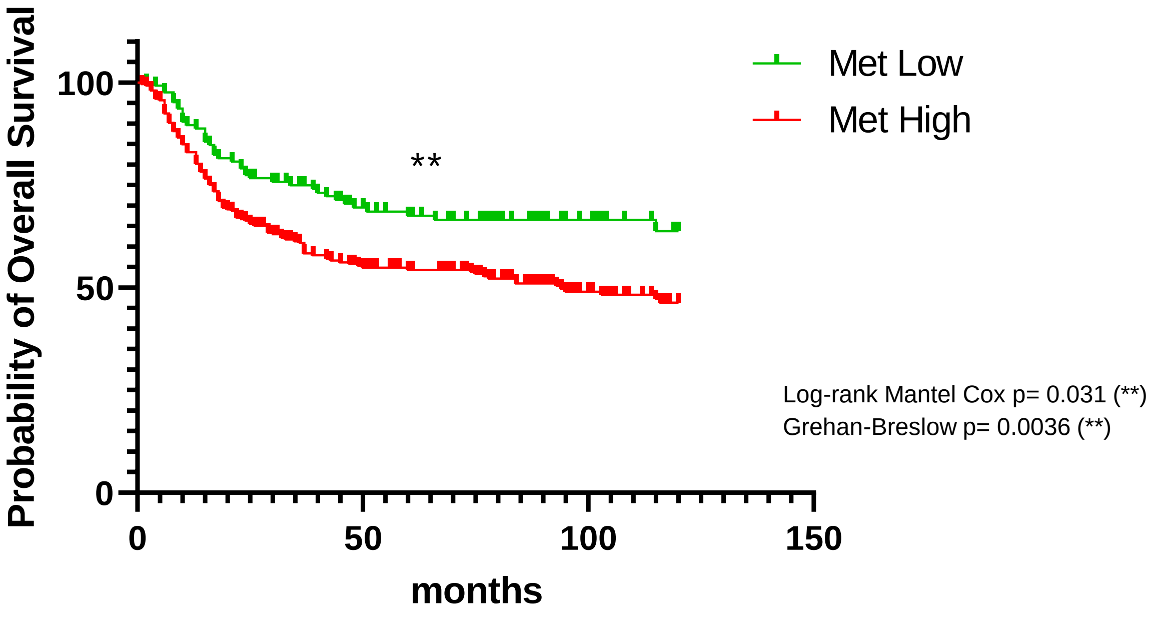

Probability of Overall Survival
100
50
0
0
50
100
150
months
**
Met Low
Met High
Log-rank Mantel Cox p= 0.031 (**)
Grehan-Breslow p= 0.0036 (**)

Figure S7

Table S1: Mammary gland (tumors and controls) used for histopathological and RPPA analyses.

Mouse #	Genotype	Gross aspect of gland	Histopathological analysis						Lung metas-tases	RPPA (Tumor #)
			Tumor state	Grade	Necrosis	Prolif. status	ER/PR/HER2 scoring	Met		
669 201	*MMTV-R26^{Met}*	Tumour	carcinoma	nd	nd	nd	0	nd	Yes	Yes (T1)
669 352	*MMTV-R26^{Met}*	Tumour	carcinoma	nd	+	nd	0	nd	Yes	Yes (T2)
669 713	*MMTV-R26^{Met}*	Tumour	carcinoma	nd	nd	nd	0	nd	_	Yes (T3)
669 163	*MMTV-R26^{Met}*	Tumour	carcinoma	nd	nd	nd	0	nd	_	Yes (T4)
669 789	*MMTV-R26^{Met}*	Tumour	carcinoma	nd	nd	nd	0	nd	Yes	Yes (T5)
669 722	*MMTV-R26^{Met}*	Tumour	carcinoma	nd	nd	nd	0	nd	Yes	Yes (T6)
669 175	*MMTV-R26^{Met}*	Tumour	carcinoma	nd	nd	nd	0	nd	_	Yes (T7)
669 715	*MMTV-R26^{Met}*	Tumour	carcinoma	nd	nd	nd	0	nd	_	Yes (T8)
671 802 (Tumor A)	*MMTV-R26^{Met}*	Tumour	carcinoma	3	+	3	0	+	_	Yes (T9)
671 607	*MMTV-R26^{Met}*	Tumour	carcinoma	3	+	3	0	+	_	Yes (T10)
671 662	*MMTV-R26^{Met}*	Tumour	carcinoma	3	++	2	0	+	_	Yes (T11)
671 670	*MMTV-R26^{Met}*	Tumour	carcinoma	3	+(+)	3	0	+	Yes	Yes (T12)
671 975	*MMTV-R26^{Met}*	Tumour	carcinoma	2	_	1	0	+	_	Yes (T13)
672 961	*MMTV-R26^{Met}*	Tumour	carcinoma	3	++	3	0	+	_	Yes (T14)
671 711	*MMTV-R26^{Met}*	Tumour	carcinoma	3	++	2	0	+	_	Yes (T15)
672 046	*MMTV-R26^{Met}*	Tumour	carcinoma	3	_	2	0	+	_	Yes (T16)
671 893	*MMTV-R26^{Met}*	Tumour	carcinoma	3	_	2	0	+	Yes	Yes (T17)
672 867	*MMTV-R26^{Met}*	Tumour	carcinoma	3	+	2	0	+	_	Yes (T18)
672 790	*MMTV-R26^{Met}*	Tumour	carcinoma	2	_	2	0	+	_	Yes (T19)
672 955	*MMTV-R26^{Met}*	Tumour	carcinoma	2-3	+	2	0	+	_	Yes (T20)
672 941	*MMTV-R26^{Met}*	Tumour	carcinoma	3	_	2	0	+	_	Yes (T23)

672 876	*MMTV-R26*^{Met}	Tumour	nd	nd	nd	nd	nd	nd	_	Yes (T21)
672 302	*MMTV-R26*^{Met}	Tumour	nd	nd	nd	nd	nd	nd	_	Yes (T22)
672 870	*MMTV-R26*^{Met}	Tumour	nd	nd	nd	nd	nd	nd	_	Yes (T24)
671 509	*MMTV-R26*^{Met}	Tumour	carcinoma	3	+	2	0	+	_	_
671 802 (Tumor B)	*MMTV-R26*^{Met}	Tumour	carcinoma	3	_	1	0	+	_	_
672 802	*MMTV-R26*^{Met}	Tumour	carcinoma	3	+	2	0	+	_	_
669 527	*MMTV-R26*^{Met}	Tumour	carcinoma	nd	nd	nd	nd	nd	_	_
669 355	*MMTV-R26*^{Met}	Tumour	carcinoma	nd	nd	nd	nd	nd	_	_
669 717	*MMTV-R26*^{Met}	Tumour	carcinoma	nd	nd	nd	nd	nd	_	_
669 854	*MMTV-R26*^{Met}	Tumour	carcinoma	nd	nd	nd	nd	nd	_	_
671 720	*MMTV-R26*^{Met}	Tumour	nd	nd	nd	nd	nd	nd	_	_
672 246	*MMTV-R26*^{Met}	Tumour	nd	nd	nd	nd	nd	nd	_	_
671 594	*MMTV*	Normal	_	nd	_	nd	nd	-	_	Yes (N1)
671 600	*MMTV*	Normal	_	nd	_	nd	nd	-	_	Yes (N2)
672 603	*MMTV-R26*^{Met}	Normal	_	nd	_	nd	nd	nd	_	Yes (N3)

Table S2: Antibodies used for RPPA analysis of *MMTV-R26^{Met}* tumors and cells

#	Official Ab Name	Ab Name Reported on Dataset	Gene Name	Company	Catalog #	Species	RPPA Dilution
1	14-3-3 beta	14-3-3-beta	YWHAB	Santa Cruz	sc-628	Rabbit	1:75
2	14-3-3 epsilon	14-3-3-epsilon	YWHAE	Santa Cruz	SC-23957	Mouse	1:50
3	14-3-3 zeta	14-3-3-zeta	YWHAZ	Santa Cruz	sc-1019	Rabbit	1:5000
4	4E-BP1	4E-BP1	EIF4EBP1	CST	9452	Rabbit	1:100
5	4E-BP1 (phospho S65)	4E-BP1_pS65	EIF4EBP1	CST	9456	Rabbit	1:250
6	4E-BP1 (phospho T37/46)	4E-BP1-pT37-T46	EIF4EBP1	CST	9459	Rabbit	1:2000
7	53BP1	53BP1	TP53BP1	CST	4937	Rabbit	1:300
8	A1Up	UBQLN4	UBQLN4	Santa Cruz	sc-136145	Mouse	1:125
9	Acetyl-CoA-Carboxylase	ACC1	ACACA, B	Epitomics/ Abcam	1768-1/ ab45174	Rabbit	1:1500
10	Acetyl-CoA-Carboxylase (phospho S79)	ACC_pS79	ACACA, B	CST	3661	Rabbit	1:500
11	ACSL1 (D2H5)	ACSL1	ACSL1	CST	9189	Rabbit	1:500
12	ACVRL1	ACVRL1	ACVRL1	Epitomics/ Abcam	2940-1/ ab108207	Rabbit	1:30
13	ADAR1	ADAR1	ADAR	Abcam	ab88574	Mouse	1:100
14	Akt	Akt	AKT1, 2, 3	CST	4691	Rabbit	1:7500
15	Akt (phospho S473)	Akt_pS473	AKT1, 2, 3	CST	9271	Rabbit	1:150
16	Akt (phospho T308)	Akt_pT308	AKT1, 2, 3	CST	2965	Rabbit	1:250
17	Akt1	Akt1	AKT1	CST	2938	Rabbit	1:1000
18	Akt1 (phospho S473)	Akt1_pS473	AKT1	CST	9018	Rabbit	1:1000
19	Akt2	Akt2	AKT2	CST	3063	Rabbit	1:3000
20	Akt2 (phospho S474)	Akt2_pS474	AKT2	CST	8599	Rabbit	1:1000
21	Ambra1 (phospho S52)	Ambra1_pS52	AMBRA1	Millipore	ABC80	Rabbit	1:250
22	AMPK alpha 2 (phospho S345)	AMPK-a2_pS345	PRKAA1, 2	Abcam	ab129081	Rabbit	1:200
23	AMPKa	AMPKa	PRKAA1, 2	CST	2532	Rabbit	1:75

**24**	AMPKa (phospho T172)	AMPKa_pT172	PRKAA1, 2	CST	2535	Rabbit	1:100
**25**	Androgen Receptor (D6F11)	AR	AR	CST	5153	Rabbit	1:250
**26**	Annexin I	Annexin-I	ANXA1	BD Biosciences	610066	Mouse	1:5000
**27**	Annexin VII	Annexin-VII	ANXA7	BD Biosciences	610668	Mouse	1:20
**28**	A-Raf	A-Raf	ARAF	CST	4432	Rabbit	1:200
**29**	A-Raf (phospho S299)	A-Raf_pS299	ARAF	CST	4431	Rabbit	1:25
**30**	ARID1A	ARID1A	ARID1A	Sigma-Aldrich	HPA005456	Rabbit	1:1000
**31**	ASNS	ASNS	ASNS	Sigma-Aldrich	HPA029318	Rabbit	1:500
**32**	Atg3	Atg3	ATG3	CST	3415	Rabbit	1:72
**33**	Atg4B	Atg4B	ATG4B	CST	13507	Rabbit	1:200
**34**	Atg5	Atg5	ATG5	CST	12994	Rabbit	1:1000
**35**	Atg7	Atg7	ATG7	CST	8558	Rabbit	1:1000
**36**	ATM	ATM	ATM	CST	2873	Rabbit	1:250
**37**	ATM (phospho S1981)	ATM_pS1981	ATM	CST	5883	Rabbit	1:20
**38**	ATP5A	ATP5A	ATP5A	Abcam	ab14748	Mouse	1:500
**39**	ATP5H	ATP5H	ATP5H	Abcam	ab110275	Mouse	1:30
**40**	ATR	ATR	ATR	CST	2790	Rabbit	1:30
**41**	ATR (phospho S428)	ATR_pS428	ATR	Abcam	ab178407	Rabbit	1:1000
**42**	ATRX	ATRX	ATRX	Abcam	ab97508	Rabbit	1:300
**43**	Aurora B/AIM1	Aurora-B	AURKB	CST	3094	Rabbit	1:38
**44**	Axl	Axl	AXL	CST	8661	Rabbit	1:500
**45**	B7-H3	B7-H3	CD276	CST	14058	Rabbit	1:200
**46**	B7-H4	B7-H4	VTCN1	CST	14572	Rabbit	1:50
**47**	Bad (phospho S112)	Bad_pS112	BAD	CST	9291	Rabbit	1:50
**48**	Bak	Bak	BAK1	Epitomics/ Abcam	1542-1/ ab32371	Rabbit	1:400
**49**	BAP1	BAP1	BAP1	Santa Cruz	sc-28383	Mouse	1:200
**50**	Bax	Bax	BAX	CST	2772	Rabbit	1:100
**51**	b-Catenin	b-Catenin	CTNNB1	CST	9562	Rabbit	1:1500
**52**	Bcl2	Bcl2	BCL2	Dako	M0887	Mouse	1:50
**53**	Bcl2A1	Bcl2A1	BCL2A1	Abnova	PAB8528	Rabbit	1:250
**54**	Bcl-xL	Bcl-xL	BCL2L1	CST	2762	Rabbit	1:100

**55**	Beclin 1	Beclin	BECN1	ThermoFisher	PA1-16857	Rabbit	1:500
**56**	beta Actin	b-Actin	ACTB	CST	4970	Rabbit	1:50
**57**	beta Catenin (phospho T41/S45)	b-Catenin_pT41_S45	CTNNB1	CST	9565	Rabbit	1:30
**58**	Bid	Bid	BID	CST	2002	Rabbit	1:500
**59**	Bim (C34C5)	Bim	BCL2L11	Epitomics/ Abcam	1036-1/ ab32158	Rabbit	1:400
**60**	BiP/GRP78	BiP-GRP78	HSPA5	BD Biosciences	610978	Mouse	1:150
**61**	BMK1/Erk5 (phospho T218/Y220)	BMK1-Erk5_pT218_Y220	MAPK7	Millipore	07-507	Rabbit	1:500
**62**	B-Raf	B-Raf	BRAF	CST	14814	Rabbit	1:500
**63**	B-Raf (phospho S445)	B-Raf_pS445	BRAF	CST	2696	Rabbit	1:75
**64**	BRD4	BRD4	BRD4	CST	13440	Rabbit	1:1000
**65**	CA9 (CAIX)	CA9	CA9	CST	5649	Rabbit	1:200
**66**	c-Abl	c-Abl	ABL1	CST	2862	Rabbit	1:100
**67**	c-Abl (phospho Y412)	Abl_pY412	ABL1	CST	2865	Rabbit	1:200
**68**	Caspase 3 (cleaved asp175)	Caspase-3-cleaved	CASP3	CST	9661	Rabbit	1:500
**69**	Caspase 7 (cleaved)	Caspase-7-cleaved	CASP7	CST	9491	Rabbit	1:60
**70**	Caspase 8	Caspase-8	CASP8	CST	9746	Mouse	1:150
**71**	Caspase 8 (cleaved asp391)	Caspase-8-cleaved	CASP8	CST	9496	Rabbit	1:500
**72**	Caspase-3	Caspase-3	CASP3	Epitomics/ Abcam	1476-1/ ab32042	Rabbit	1:250
**73**	Caveolin 1	Caveolin-1	CAV1	CST	3238	Rabbit	1:3000
**74**	CD134/OX40	CD134	TNFRSF	Abcam	ab76000	Rabbit	1:100
**75**	CD171 (L1)	CD171	L1CAM	Biolegend	826701	Mouse	1:1000
**76**	CD20	CD20	MS4A1	Epitomics/ Abcam	1632-1/ ab78237	Rabbit	1:75
**77**	CD26	CD26	DPP4	Abcam	ab28340	Rabbit	1:1000
**78**	CD29	CD29	ITGB1	BD Biosciences	610467	Mouse	1:30
**79**	CD31	CD31	PECAM1	Dako/ Fisher	M0823/ MS353S	Mouse	1:25
**80**	CD38	CD38	CD38	Abcam	ab108403	Rabbit	1:250
**81**	CD4	CD4	CD4	Abcam	ab133616	Rabbit	1:500

**82**	CD44	CD44	CD44	CST	3570	Mouse	1:20
**83**	CD45	CD45	CD45	DAKO/ ThermoFisher	M070129-2/ MS355P	Mouse	1:1000
**84**	CD49b	CD49b	ITGA2	BD Biosciences	611016	Mouse	1:50
**85**	CD86	CD86	CD86	Abcam	ab53004	Rabbit	
**86**	Cdc2 (phospho Y15)	cdc2_pY15	CDK	CST	4539	Rabbit	1:38
**87**	cdc25C	cdc25C	CDC25C	CST	4688	Rabbit	1:250
**88**	CDK1/2/3 (phospho T14)	CDK1_pT14	CDK1, 2, 3	Abcam	ab32384	Rabbit	1:1000
**89**	CDKN2A/p16INK4 a	p16INK4a	CDKN2A	Abcam	ab81278	Rabbit	1:500
**90**	Chk1	Chk1	CHEK	CST	2360	Mouse	1:100
**91**	Chk1 (phospho S296)	Chk1_pS296	CHEK1	Abcam	ab79758	Rabbit	1:125
**92**	Chk1 (phospho S345)	Chk1_pS345	CHEK1	CST	2348	Rabbit	1:30
**93**	Chk2	Chk2	CHEK2	CST	3440	Mouse	1:50
**94**	Chk2 (phospho T68)	Chk2_pT68	CHEK2	CST	2197	Rabbit	1:250
**95**	c-IAP2	c-IAP2	BIRC3	CST	3130	Rabbit	1:50
**96**	CIITA	CIITA	CIITA	CST	3793	Rabbit	1:250
**97**	c-Jun (phospho S73)	c-Jun_pS73	JUN	CST	9164	Rabbit	1:30
**98**	c-Kit	c-Kit	KIT	Epitomics/ Abcam	1522-1/ ab32363	Rabbit	1:250
**99**	Claudin 7	Claudin-7	CLDN7	Abcam	ab79481	Rabbit	1:250
**100**	c-Myc	c-Myc	MYC	Santa Cruz	sc-764	Rabbit	1:250
**101**	COG3	COG3	COG3	ProteinTech	11130-1-AP	Rabbit	1:750
**102**	Collagen-VI/COL6A1	Collagen-VI	COL6A1	Santa Cruz	sc-20649	Rabbit	1:6000
**103**	Complex II Subunit	Complex-II-Subunit	SDHB	Life Technologies	459230	Mouse	1:200
**104**	Connexin 43	Connexin-43	GJA1	CST	3512	Rabbit	1:150
**105**	Coup-TFII	Coup-TFII	NR2F2	CST	6434	Rabbit	1:50
**106**	Cox2	Cox2	PTGS2	CST	4842	Rabbit	1:75
**107**	Cox-IV	Cox-IV	COX4I1	CST	4850	Rabbit	1:5000
**108**	C-Raf	C-Raf	RAF1	Millipore	04-739	Rabbit	1:100
**109**	C-Raf (phospho S338)	C-Raf_pS338	RAF1	CST	9427	Rabbit	1:200

**110**	Creb	Creb	CREB1	CST	9197	Rabbit	1:75
**111**	CSK	CSK	CSK	CST	4980	Rabbit	1:300
**112**	CtIP	CtIP	RBBP8	CST	9201	Rabbit	1:500
**113**	Cyclin B1	Cyclin B1	CCNB1	Epitomics/ Abcam	1495-1/ ab32053	Rabbit	1:1500
**114**	Cyclin D1	Cyclin-D1	CCND1	Millipore Sigma	SAB4502603	Rabbit	1:200
**115**	Cyclin D3	Cyclin D3	CCND3	CST	2936	Mouse	1:1000
**116**	Cyclin E1	Cyclin E1	CCNE1	Santa Cruz	sc-247	Mouse	1:25
**117**	Cyclophilin-F	Cyclophilin-F	PPIF	Abcam	MSA04/ ab110324	Mouse	1:50000
**118**	Cytokeratin 19	Cytokeratin-19	KRT19	Dako	M0888	Mouse	1:50
**119**	DAP Kinase 1 (phospho S308)	DAPK1_pS308	DAPK1	GeneTex	GTX10524	Mouse	1:200
**120**	DAP Kinase 2	DAPK2	DAPK2	Abcam	ab51601	Rabbit	1:250
**121**	DDB-1	DDB-1	DDB1	CST	6998	Rabbit	1:5000
**122**	Detyrosinated alpha-Tubulin	D-a-Tubulin	TUBA4A, TUBA3C	Abcam	ab48389	Rabbit	1:1500
**123**	Di-Methyl-Histone H3 (Lys4/C64G9)	DM-Histone-H3	HIST1H3A	CST	9725	Rabbit	1:100
**124**	Dimethyl-K9 Histone H3	DM-K9-Histone-H3	HIST3H3	Abcam	ab1220	Mouse	1:250
**125**	DNA Ligase IV	DNA-Ligase-IV	LIG4	CST	14649	Rabbit	1:1000
**126**	DNA Polymerase gamma (D1Y6R)	POLG	POLG	CST	13609	Rabbit	1:500
**127**	DNMT1 (D63A6)	DNMT1	DNMT1	CST	5032	Rabbit	1:500
**128**	DRP1 (D8H5)	DRP1	DNM1L	CST	5391	Rabbit	1:1000
**129**	DUSP4/MKP2	DUSP4	DUSP4	CST	5149	Rabbit	1:150
**130**	DUSP6	DUSP6	DUSP6	Abcam	ab76310	Rabbit	1:750
**131**	Dvl3	Dvl3	DVL3	CST	3218	Rabbit	1:30
**132**	E2F1	E2F1	E2F1	Santa Cruz	sc-251	Mouse	1:20
**133**	E-Cadherin	E-Cadherin	CDH1	CST	3195	Rabbit	1:150
**134**	eEF2	eEF2	EEF2	CST	2332	Rabbit	1:50
**135**	eEF2K	eEF2K	EEF2K	CST	3692	Rabbit	1:50
**136**	EGFR	EGFR	EGFR	CST	2232	Rabbit	1:75
**137**	EGFR (phospho Y1173)	EGFR_pY1173	EGFR	Epitomics/ Abcam	1124-1/ ab32578	Rabbit	1:300
**138**	eIF4E	eIF4E	EIF4E	CST	9742	Rabbit	1:75
**139**	eIF4E (phospho S209)	eIF4E_pS209	EIF4E	Abcam	ab76256	Rabbit	1:250

**140**	eIF4G	eIF4G	EIF4G1	CST	2498	Rabbit	1:1000
**141**	Elk1 (phospho S383)	Elk1_pS383	ELK1	CST	9181	Rabbit	1:50
**142**	Enolase-2 (D20H2)	Enolase-2	ENO2	CST	8171	Rabbit	1:250
**143**	ENY2	ENY2	ENY2	GeneTex	GTX629542	Mouse	1:500
**144**	Eph Receptor A2	EPHA2	EPHA2	Abcam	ab133501	Rabbit	1:1000
**145**	Epithelial Membrane Antigen	EMA	MUC1	DAKO	M061329-2	Mouse	1:750
**146**	ErbB3/HER3	HER3	ERBB3	Santa Cruz	sc-285	Rabbit	1:300
**147**	ErbB3/HER3 (phospho Y1289)	HER3_pY1289	ERBB3	CST	4791	Rabbit	1:50
**148**	ERCC1	ERCC1	ERCC1	Santa Cruz	sc-17809	Mouse	1:38
**149**	Erk5	Erk5	MAPK7	CST	3552	Rabbit	1:500
**150**	ERRalpha (E1G1J)	ERRalpha	ESRRA	CST	13826	Rabbit	1:500
**151**	ERRFI1/MIG6	MIG6	ERRFI1	Sigma-Aldrich	WH0054206 M1	Mouse	1:50
**152**	Estrogen Receptor	ER	ESR1	Lab Vision	RM-9101	Rabbit	1:40
**153**	Estrogen Receptor alpha	ER-a	ERSA	CST	13258	Rabbit	1:500
**154**	Estrogen Receptor alpha (phospho S118)	ER-a_pS118	ESR1	Epitomics/ Abcam	1091-1/ ab32396	Rabbit	1:500
**155**	Ets-1	Ets-1	ETS1	Bethyl	A303-501A	Rabbit	1:100
**156**	FAK	FAK	PTK2	Epitomics/ Abcam	1700-1/ ab40794	Rabbit	1:1000
**157**	FAK (phospho Y397)	FAK_pY397	PTK2	CST	3283	Rabbit	1:25
**158**	Fatty Acid Synthase	FASN	FASN	CST	3180	Rabbit	1:1000
**159**	FGF-basic	FGF-basic	FGF2	VWR	10775-082 (500-P18)	Rabbit	1:1000
**160**	Fibronectin	Fibronectin	FN1	Epitomics	1574-1	Rabbit	1:10000
**161**	FoxM1	FOXM1	FOXM1	CST	5436	Rabbit	1:30
**162**	FoxO3a	FoxO3a	FOXO3	CST	2497	Rabbit	1:20
**163**	FoxO3a (phospho S318/S321)	FoxO3a_pS318_S3 21	FOXO3	CST	9465	Rabbit	1:30
**164**	FRS2-a (phospho Y196)	FRS2-a_pY196	FRS2	CST	3864	Rabbit	1:100
**165**	G6PD	G6PD	G6PD	Santa Cruz	sc-373887	Mouse	1:75

**166**	Gab2	Gab2	GAB2	CST	3239	Rabbit	1:300
**167**	GAPDH	GAPDH	GAPDH	Ambion/ Invitrogen	AM4300	Mouse	1:75000
**168**	GATA3	GATA3	GATA3	BD Biosciences	558686	Mouse	1:150
**169**	GATA6	GATA6	GATA6	CST	5851	Rabbit	1:200
**170**	GCLC	GCLC	GCLC	Proteintech Group	12601-1-AP	Rabbit	1:500
**171**	GCLM	GCLM	GCLM	Abcam	ab124827	Rabbit	1:500
**172**	GCN5L2	GCN5L2	KAT2A	CST	3305	Rabbit	1:30
**173**	Gli1	Gli1	GLI1	CST	3538	Rabbit	1:3000
**174**	Gli3	Gli3	GLI3	Abcam	ab69838	Rabbit	1:1000
**175**	Glucose-6 Phosphate Dehydrogenase	G6PD	G6PD	CST	8866	Rabbit	1:30
**176**	Glutamate Dehydrogenase1/ 2	Glutamate-D1-2	GLUD1	Novus	NBP2-16679	Rabbit	1:500
**177**	Glutaminase	Glutaminase	GLS	Abcam	ab156876	Rabbit	1:150
**178**	Glycogen Synthase	Gys	GYS1	CST	3886	Rabbit	1:2000
**179**	Glycogen Synthase (phospho S641)	Gys_pS641	GYS1	CST	3891	Rabbit	1:300
**180**	GPBB	GPBB	PYGM	Novus	NBP1-32799	Rabbit	1:200
**181**	Granzyme B	Granzyme-B	GZMB	CST	4275	Rabbit	1:500
**182**	GRB7	GRB7	GRB7	Abcam	ab183737	Rabbit	1:500
**183**	Grp75 (D13H4)	Grp75	HSPA9	CST	3593	Rabbit	1:250
**184**	GSK-3alpha/beta	GSK-3a-b	GSK3A, B	Santa Cruz	sc-7291	Mouse	1:750
**185**	GSK-3alpha/beta (phospho S21/S9)	GSK-3a-b_pS21_S9	GSK3A, B	CST	9331	Rabbit	1:200
**186**	GSK-3B	GSK-3B	GSK3B	CST	9315	Rabbit	1:750
**187**	GSK-3beta (phospho S9)	GSK-3b_pS9	GSK3B	CST	5558	Rabbit	1:250
**188**	H2AX (phospho S140)	H2AX_pS140	H2AFX	Pierce Biotechnolog y	MA12022	Mouse	1:100
**189**	Hamartin/TSC1	TSC1	TSC1	CST	4906	Rabbit	1:200
**190**	HER2	HER2	ERBB2	Lab Vision	MS-325-P1	Mouse	1:300
**191**	HER2 (phospho Y1248)	HER2_pY1248	ERBB2	R&D systems	AF1768	Rabbit	1:1500
**192**	Heregulin	Heregulin	NRG1	CST	2573	Rabbit	01:30

**193**	HES1	HES1	HES1	CST	11988	Rabbit	1:500
**194**	Hexokinase II	Hexokinase II	HK2	CST	2106	Rabbit	1:100
**195**	Hif-1-alpha	Hif-1-alpha	HIF1A	BD Biosciences	610958	Mouse	1:20
**196**	Histone H3	Histone H3	HIST3H3	Abcam	ab1791	Rabbit	1:5000
**197**	HLA-DQA1	HLA-DQA1	HLA-DQA1	Abcam	ab128959	Rabbit	1:3000
**198**	HLA-DR/DP/DQ/DX	HLA-DR-DP-DQ-DX	HLA-DRA	Santa Cruz	sc-53302	Mouse	1:250
**199**	HMHA1	HMHA1	HMHA1	ProteinTech	14832-1-AP	Rabbit	1:3000
**200**	HSP27	HSP27	HSBP1	CST	2402	Mouse	1:75
**201**	HSP27 (phospho S82)	HSP27_pS82	HSBP1	CST	2401	Rabbit	1:75
**202**	HSP60	HSP60	HSP60	CST	12165	Rabbit	1:1000
**203**	HSP70	HSP70	HSPA1A	CST	4872	Rabbit	1:50
**204**	Hsp75/TRAP1	TRAP1	TRAP1	BD Biosciences	612344	Mouse	1:750
**205**	IDO	IDO	IDO1	CST	86630	Rabbit	1:200
**206**	IGF1R (phospho Y1135/Y1136	IGF1R_pY1135_Y1136	IGF1R, INSR	CST	3024	Rabbit	1:30
**207**	IGF-1Receptor beta	IGF1R-b	IGF1R	CST	3018	Rabbit	1:50
**208**	IGFBP2	IGFBP2	IGFBP2	CST	3922	Rabbit	1:50
**209**	IGFBP3	IGFBP3	IGFBP3	BD Biosciences	611504	Mouse	1:1000
**210**	IGFRb	IGFRb	IGF1R	CST	3027	Rabbit	1:250
**211**	IL-6	IL-6	IL6	CST	12153	Rabbit	1:250
**212**	INPP4b	INPP4b	INPP4B	CST	4039	Rabbit	01:30
**213**	Insulin Receptor beta	IR-b	INSR	CST	3025	Rabbit	1:100
**214**	IRF-1	IRF-1	IRF1	CST	8478	Rabbit	1:250
**215**	IRS1	IRS1	IRS1	Millipore	06-248	Rabbit	1:250
**216**	IRS2	IRS2	IRS2	CST	4502	Rabbit	1:100
**217**	JAB1	JAB1	COPS5	Santa Cruz	sc-13157	Mouse	1:30
**218**	Jagged1	Jagged1	JAG1	Abcam	ab109536	Rabbit	01:50
**219**	Jak2	Jak2	JAK2	CST	3230	Rabbit	1:750
**220**	JNK (phospho T183/Y185)	JNK_pT183_Y185	MAPK8	CST	4668	Rabbit	01:30
**221**	JNK2	JNK2	MAPK9	CST	4672	Rabbit	1:25
**222**	KAP1	KAP1	TRIM28	Abcam	ab10484	Rabbit	1:2000
**223**	KMT3A/HYPB/HIF-1	SETD2	SETD2	abcam	ab184190	Rabbit	1:1000

**224**	LAD1	LAD1	LAD1	Atlas	HPA028732	Rabbit	1:500
**225**	Lasu1/Ureb1	Lasu1	HUWE1	Bethyl	IHC-00439	Rabbit	1:1000
**226**	LC3A/B	LC3A-B	MAP1LC3A, B	CST	4108	Rabbit	1:250
**227**	Lck	Lck	LCK	CST	2752	Rabbit	1:75
**228**	LDHA	LDHA	LDHA	CST	3582	Rabbit	1:250
**229**	LRP6 (phospho S1490)	LRP6_pS1490	LRP6	CST	2568	Rabbit	1:250
**230**	MAPK (phospho T202/Y204)	MAPK_pT202/Y204	MAPK1, 3	CST	4377	Rabbit	1:25
**231**	Mcl-1	Mcl-1	MCL1	CST	5453	Rabbit	1:100
**232**	MDM2 (phospho S166)	MDM2_pS166	MDM2	CST	3521	Rabbit	1:60
**233**	MEK1	MEK1	MAP2K1	Epitomics/ Abcam	1235-1/ ab32576	Rabbit	1:1500
**234**	MEK1 (phospho S217/S221)	MEK1_p_S217/ S221	MAP2K1, 2	CST	9154	Rabbit	1:50
**235**	MEK2	MEK2	MAP2K2	CST	9125	Rabbit	1:50
**236**	MelanA	MelanA	MLANA	Abcam	ab51061	Rabbit	1:500
**237**	Melanoma gp100	Melan-gp100	PMEL	Abcam	ab137078	Rabbit	1:500
**238**	MERIT40	MERIT40	MERIT40	CST	12711	Rabbit	1:3000
**239**	MERIT40 (phospho S29)	MERIT40_pS29	BABAM1	CST	12110	Rabbit	1:300
**240**	Merlin/NF2	Merlin	NF2	Novus	22710002	Rabbit	1:250
**241**	MIF	MIF	MIF	Santa Cruz	sc-130329	Rabbit	1:100
**242**	MITF (D5G7V)	MITF	MITF	CST	12590	Rabbit	1:500
**243**	Mitofusin-1	Mitofusin-1	MFN1	CST	14739	Rabbit	1:500
**244**	Mitofusin-2	Mitofusin-2	MFN2	CST	11925	Rabbit	1:1000
**245**	MLH1 (4C9C7)	MLH1	MLH1	CST	3515	Mouse	1:500
**246**	MLKL	MLKL	MLKL	CST	14993	Rabbit	1:1000
**247**	MMP2	MMP2	MMP2	CST	4022	Rabbit	1:75
**248**	Mnk1	Mnk1	MKNK1	CST	2195	Rabbit	1:750
**249**	Monocarboxylic Acid Transporter 4	MCT4	SLC16A4	Millipore	AB3314P	Rabbit	1:500
**250**	MR1	MR1	MR1	Santa Cruz	sc-377312	Mouse	1:500
**251**	MRAP	MRAP	MRAP	Abcam	ab103319	Rabbit	1:500
**252**	MSH2 (D24B5)	MSH2	MSH2	CST	2017	Rabbit	1:750
**253**	MSH6	MSH6	MSH6	Novus	22030002	Rabbit	1:1000
**254**	MSI2 (EP1305Y)	MSI2	MSI2	Abcam	ab76148	Rabbit	1:1000

**255**	MTCO1	MTCO1	MTCO1	Abcam	ab14705	Mouse	1:500
**256**	mTOR	mTOR	MTOR	CST	2983	Rabbit	1:3000
**257**	mTOR (phospho S2448)	mTOR_pS2448	MTOR	CST	2971	Rabbit	1:50
**258**	MTSS1	MTSS1	MTSS1	Novus	H00009788-M01A	Mouse	1:250
**259**	Myosin Heavy Chain 11	Myosin-11	MYH11	Novus	21370002	Rabbit	1:1000
**260**	Myosin IIa	Myosin-IIa	MYH9	CST	3403	Rabbit	1:1000
**261**	Myosin IIa (phospho S1943)	Myosin-IIa_pS1943	MYH9	CST	5026	Rabbit	1:750
**262**	Myt1	Myt1	PKMYT1	CST	4282	Rabbit	1:100
**263**	NAPSIN-A	NAPSIN-A	NAPSA	Epitomics/ Abcam	5795-1/ ab129189	Rabbit	1:150
**264**	N-Cadherin	N-Cadherin	CDH2	CST	4061	Rabbit	1:25
**265**	NDRG1 (phospho T346)	NDRG1_pT346	NDRG1	CST	3217	Rabbit	01:50
**266**	NDUFB4	NDUFB4	NDUFB4	Abcam	ab110243	Mouse	1:25
**267**	NF-kB p65 (phospho S536)	NF-kB-p65_pS536	RELA	CST	3033	Rabbit	1:30
**268**	Notch1	Notch1	NOTCH1	CST	3268	Rabbit	01:30
**299**	Notch1 (Cleaved)	Notch1-cleaved	NOTCH1	CST	4147	Rabbit	1:100
**270**	Notch3	Notch3	NOTCH3	Novus	H00004854-M01	Mouse	1:250
**271**	NQO1	NQO1	NQO1	CST	3187	Mouse	1:15000
**272**	N-Ras	N-Ras	NRAS	Santa Cruz	sc-31	Mouse	1:50
**273**	NRF2	NRF2	NRF2	CST	12721	Rabbit	1:500
**274**	Oct-4	Oct-4	POU5F1	CST	2750	Rabbit	1:40
**275**	p16/INK4a	p16-INK4a	CDKN2A	Epitomics/ Abcam	1712-1/ ab40803	Rabbit	1:500
**276**	p21	p21	CDKN1A	Santa Cruz	sc-6246	Rabbit	1:150
**277**	p27 (phospho T157)	p27_pT157	CDKN1B	R&D Systems	AF1555	Rabbit	1:30
**278**	p27 (phospho T198)	p27_pT198	CDKN1B	Abcam	ab64949	Rabbit	01:50
**279**	p27 KIP 1	p27-Kip-1	CDKN1B	Epitomics/ Abcam	1591-1/ ab32034	Rabbit	01:40
**280**	p38 (phospho T180/Y182)	p38_pT180_Y182	MAPK11, 12, 13, 14	CST	9211	Rabbit	1:38
**281**	p38 alpha MAPK	p38-a	MAPK1	CST	9228	Mouse	1:300
**282**	p38 MAPK	p38-MAPK	MAPK11, 12, 14	CST	9212	Rabbit	1:1500

**283**	p38/MAPK (phospho T180/Y182)	p38-MAPK_pT180_Y182	MAPK14	CST	9215	Rabbit	1:250
**284**	p44/42 MAPK	p44-42-MAPK	MAPK1, 3	CST	4695	Rabbit	1:2000
**285**	p53	p53	TP53	CST	9282	Rabbit	1:2500
**286**	p70 S6 Kinase (phospho T389)	p70-S6K_pT389	RPS6KB1	CST	9205	Rabbit	1:50
**287**	p70/S6K1	p70-S6K1	RPS6KB1	Epitomics/ Abcam	1494-1/ ab32529	Rabbit	1:300
**288**	p90RSK (phospho T573)	p90RSK_pT573	RPS6K	CST	9346	Rabbit	1:25
**289**	PAI-1	PAI-1	SERPINE1	BD Biosciences	612024	Mouse	1:50
**290**	PAICS	PAICS	PAICS	Sigma-Aldrich	HPA035895	Rabbit	1:250
**291**	PAK1	PAK1	PAK1	CST	2602	Rabbit	1:750
**292**	PAK4	PAK4	PAK4	CST	3242	Rabbit	1:300
**293**	PAR	PAR	PAR	Trevigen	4336-BPC-100	Rabbit	1:30000
**294**	PARG	PARG	PARG	CST	66564	Rabbit	1:1000
**295**	PARK7/DJ1	DJ1	PARK7	Abcam	ab76008	Rabbit	1:5000
**296**	PARP	PARP	PARP1	CST	9532	Rabbit	1:1000
**297**	Patched	Patched	PTCH1	Abcam	ab53715	Rabbit	1:1000
**298**	Paxillin	Paxillin	PXN	CST	2542	Rabbit	1:250
**299**	P-Cadherin	P-Cadherin	CDH3	CST	2130	Rabbit	1:38
**300**	PCNA	PCNA	PCNA	CST	2586	Mouse	1:250
**301**	PD-1	PD-1	PDCD1	CST	43248	Mouse	1:500
**302**	Pdcd4	Pdcd4	PDCD4	Rockland	600-401-965	Rabbit	1:750
**303**	PDGFRB	PDGFR-b	PDGFRB	Invitrogen	MA5-15143	Rabbit	1:500
**304**	PDH	PDH	PDH	Abcam	ab110332	Mouse	1:100
**305**	PDHK1	PDHK1	PDHK1	CST	3820	Rabbit	1:300
**306**	PDK1	PDK1	PDPK1	CST	3062	Rabbit	01:50
**307**	PDK1 (phospho S241)	PDK1_pS241	PDPK1	CST	3061	Rabbit	01:50
**308**	PD-L1	PD-L1	CD274	CST	13684	Rabbit	1:250
**309**	PEA-15	PEA-15	PEA15	CST	2780S	Rabbit	1:100
**310**	PED/PEA-15 (phospho S116)	PEA-15_pS116	PEA15	Life Technologies	44836G	Rabbit	1:100
**311**	PHGDH	PHGDH	PHGDH	CST	13428	Rabbit	1:1000
**312**	PI3 Kinase p110 alpha	PI3K-p110-a	PIK3CA	CST	4255	Rabbit	1:50
**313**	PI3K p110 beta	PI3K-p110-b	PIK3BC	Santa Cruz	sc-376412	Mouse	1:40

**314**	PI3K p85	PI3K-p85	PIK3R1	Millipore	06-195	Rabbit	1:15000
**315**	PKA RI alpha	PKA-a	PRKAR1A	CST	5675	Rabbit	1:250
**316**	PKC alpha/beta II (phospho T638/641)	PKC-a-b-II_pT638_T641	PRKCA, B	CST	9375	Rabbit	1:1000
**317**	PKC(pan) beta II (phospho S660)	PKC-b-II_pS660	PRKCA, B, D, E, H, Q	CST	9371	Rabbit	1:200
**318**	PKC delta (phospho S664)	PKC-delta_pS664	PRKCD	Millipore	07-875	Rabbit	1:75
**319**	PKCalpha	PKCa	PRKCA	CST	2056	Rabbit	1:200
**320**	PKM2	PKM2	PKM	CST	4053	Rabbit	1:300
**321**	PLC gamma2 (phospho Y759)	PLC-gamma2_pY759	PLCG2	CST	3874	Rabbit	01:25
**322**	PLK1	PLK1	PLK1	CST	4513	Rabbit	1:125
**323**	Met (phospho Y1234/Y1235)	c-Met_pY1234_Y1235	MET	CST	3129	Rabbit	1:100
**324**	PMS2	PMS2	PMS2	Novus Biologicals	22510002	Rabbit	1:1500
**325**	PRAS40	PRAS40	AKT1S1	Life Technologies	AHO1031	Mouse	1:75
**326**	PRAS40 (phospho T246)	PRAS40_pT246	AKT1S1	Life Technologies	441100G	Rabbit	1:500
**327**	PREX1	PREX1	PREX1	Abcam	ab102739	Rabbit	1:100
**328**	Progesterone Receptor [YR85]	PR	PGR	abcam	206926	Rabbit	1:500
**329**	PTEN	PTEN	PTEN	CST	9552	Rabbit	1:500
**330**	PTPN12	PTPN12	PTPN12	Abcam	ab76942	Rabbit	1:500
**331**	Puma	Puma	BBC3	CST	4976	Rabbit	1:50
**332**	PYGB	PYGB	PYGB	Sigma-Aldrich	SAB2900066	Rabbit	1:750
**333**	PYGM	PYGM	PYGM	Novus	H00005837-M10	Mouse	1:500
**334**	Pyk2 (phospho Y402)	Pyk2_pY402	PYK2	CST	3291	Rabbit	1:500
**335**	Pyruvate Dehydrogenase	PDHA1	PDHA1	CST	3205	Rabbit	1:200
**336**	Rab11	Rab11	RAB11A, B	CST	3539	Rabbit	1:30
**337**	Rab25	Rab25	RAB25	CST	4314	Rabbit	1:30
**338**	Rac1/Cdc42	Cdc42	CDC42	CST	4651	Rabbit	1:100
**339**	Rad23A	Rad23A	RAD23A	CST	24555	Rabbit	1:1000
**340**	Rad50	Rad50	RAD50	CST	3427	Rabbit	1:250
**341**	Rad51	Rad51	RAD51	Millipore	ABE257	Rabbit	1:1000

**342**	Raptor	Raptor	RPTOR	CST	2280	Rabbit	1:300
**343**	Rb	Rb	RB1	CST	9309	Mouse	1:150
**344**	Rb (phospho S807/811)	Rb_pS807_S811	RB1	CST	9308	Rabbit	1:1000
**345**	RBM15	RBM15	RBM15	Novus	21390002	Rabbit	1:5000
**346**	Rheb	Rheb	RHEB	R&D Systems	MAB3426	Mouse	1:75
**347**	Rictor	Rictor	RICTOR	CST	2114	Rabbit	1:100
**348**	Rictor (phospho T1135)	Rictor_pT1135	RICTOR	CST	3806	Rabbit	1:200
**349**	RIP	RIP	RIP	CST	4926	Rabbit	1:75
**350**	RIP3	RIP3	RIP3	CST	13526	Rabbit	1:500
**351**	RPA32 (phospho S4/S8)	RPA32_pS4/S8	RPA2	Bethyl	A300-245A	Rabbit	1:250
**352**	RPA32/RPA2	RPA32	RPA2	CST	2208	Rat	1:150
**353**	RRM1	RRM1	RRM1	CST	3388	Rabbit	1:100
**354**	RRM2	RRM2	RRM2	Life Technologies	PA527856	Rabbit	1:250
**355**	RSK	RSK	RPS6KA1, 2, 3	CST	9347	Rabbit	1:150
**356**	S100A4	S100A4	S100A4	CST	13018	Rabbit	1:1000
**357**	S6 (phospho S235/236)	S6_pS235_S236	RPS6	CST	2211	Rabbit	1:2500
**358**	S6 (phospho S240/244)	S6_pS240_S244	RPS6	CST	2215	Rabbit	1:1000
**359**	S6 Ribosomal Protein	S6	RPS6	CST	2317	Mouse	1:750
**360**	SCD	SCD	SCD	Santa Cruz	sc-58420	Mouse	1:20
**361**	SDHA	SDHA	SDHA	CST	11998	Rabbit	1:250
**362**	SFRP1	SFRP1	SFRP1	CST	4690	Rabbit	1:500
**363**	Shc_pY317	Shc_pY317	SHC1	CST	2431	Rabbit	01:25
**364**	SHP-2 (phospho Y542)	SHP-2_pY542	PTPN11	CST	3751	Rabbit	1:75
**365**	SHP2 / PTPN11	SHP2	PTPN11	CST	3397	Rabbit	1:250
**366**	SLC1A5	SLC1A5	SLC1A5	Sigma-Aldrich	HPA035240	Rabbit	1:150000
**367**	Slfn11	Slfn11	SLFN11	Santa Cruz	sc-374339	Mouse	1:150
**368**	Smac	Smac	DIABLO	CST	2954	Mouse	1:150
**369**	Smad1	Smad1	SMAD1	Epitomics/ Abcam	1649-1/ ab33902	Rabbit	1:500
**370**	Smad3	Smad3	SMAD3	Epitomics/ Abcam	1735-1/ ab40854	Rabbit	1:150
**371**	Smad4	Smad4	SMAD4	Santa Cruz	sc-7966	Mouse	1:30

**372**	Snail	Snail	SNAI1	CST	3895	Mouse	1:50
**373**	SOD1	SOD1	SOD1	CST	4266	Mouse	1:500
**374**	SOD2 (D9V9C)	SOD2	SOD2	CST	13194	Rabbit	1:200
**375**	Sox2	Sox2	SOX2	CST	2748	Rabbit	1:50
**376**	Src	Src	SRC	Millipore	05-184	Mouse	1:50
**377**	Src (phospho Y416)	Src_pY419	SRC	CST	2101	Rabbit	1:25
**378**	Src (phospho Y527)	Src_pY527	SRC	CST	2105	Rabbit	1:150
**379**	SRSF1/SF2	SF2	SRSF1	Invitrogen	324500	Mouse	1:75
**380**	Stat3	Stat3	STAT3	CST	4904	Rabbit	1:3000
**381**	Stat3 (phospho Y705)	Stat3_pY705	STAT3	CST	9145	Rabbit	1:100
**382**	Stat5a	Stat5a	STAT5A	Epitomics/ Abcam	1289-1/ ab32043	Rabbit	1:300
**383**	Stathmin-1	Stathmin-1	STMN1	Epitomics/ Abcam	1972-1/ ab52630	Rabbit	1:75
**384**	STING	STING	TMEM173	CST	13647	Rabbit	1:250
**385**	Syk	Syk	SYK	Santa Cruz	sc-1240	Mouse	1:500
**386**	Tau	Tau	MAPT	Millipore	05-348	Mouse	1:100
**387**	TAZ	TAZ	WWTR1	CST	4883	Rabbit	1:300
**388**	TFAM	TFAM	TFAM	CST	7495	Rabbit	1:300
**389**	Transferrin R	TFRC	TFRC	Novus	22500002	Rabbit	1:15000
**390**	TIGAR	TIGAR	TIGAR	Epitomics/ Abcam	S1711/ ab137573	Rabbit	1:100
**391**	Transglutaminase	Transglutaminase	TGM2	Lab Vision	MS-224-P1	Mouse	1:150
**392**	TRIM25	TRIM25	TRIM25	Abcam	ab167154	Rabbit	1:3000
**393**	TTF1	TTF1	NKX2-1	Epitomics/ Abcam	2044-1/ ab76013	Rabbit	1:150
**394**	Tuberin	Tuberin	TSC2	Epitomics/ Abcam	1613-1/ ab32554	Rabbit	1:2500
**395**	Tuberin/TSC2 (phospho T1462)	Tuberin_pT1462	TSC2	CST	3617	Rabbit	1:38
**396**	TUFM	TUFM	TUFM	Abcam	ab173300	Rabbit	1:38
**397**	TWEAK Receptor/FN14	FN14	TNFRSF12A	CST	4403	Rabbit	1:1000
**398**	TWIST	TWIST	TWIST1	Santa Cruz	sc-81417	Mouse	1:30
**399**	Tyro3	Tyro3	TYRO3	CST	5585	Rabbit	1:30
**400**	UBAC1	UBAC1	UBAC1	Sigma-Aldrich	HPA005651	Rabbit	1:250
**401**	Ubiquityl-Histone H2B	U-Histone-H2B	HIST1H2BB	CST	5546	Rabbit	1:500

**402**	UGT1A	UGT1A	UGT1A1, 3, 4, 5, 7, 8, 10	Santa Cruz	sc-271268	Mouse	1:75
**403**	ULK1 (phospho S757)	ULK1_pS757	ULK1	CST	6888	Rabbit	1:300
**404**	UQCRC2	UQCRC2	UQCRC2	MitoSciences / Abcam	MS304/ ab14745	Mouse	01:50
**405**	UVRAG	UVRAG	UVRAG	CST	13115	Rabbit	1:100
**406**	VASP	VASP	VASP	CST	3112	Rabbit	1:100
**407**	Vav1	Vav1	VAV1	CST	2502	Rabbit	1:500
**408**	VDAC1/Porin	Porin	VDAC1	Abcam	ab14734	Mouse	1:100
**409**	VEGF Receptor 2	VEGFR-2	KDR	CST	2479	Rabbit	1:3000
**410**	VHL/EPPK1	VHL-EPPK1	EPPK1	BD Biosciences	556347	Mouse	1:1500
**411**	Vimentin	Vimentin	VIM	Dako/Fisher	M0725/ MS-129-P	Mouse	1:250
**412**	Vinculin	Vinculin	VCL	Sigma-Aldrich	SAB4200080	Mouse	1:25000
**413**	Wee1	Wee1	WEE1	CST	4936	Rabbit	1:250
**414**	Wee1 (phospho S642)	Wee1_pS642	WEE1	CST	4910	Rabbit	1:50
**415**	WIPI1	WIPI1	WIPI1	CST	12124	Rabbit	1:150
**416**	WIPI2	WIPI2	WIPI2	CST	8567	Rabbit	1:150
**417**	XBP-1	XBP-1	XBP1	Santa Cruz	sc-32136	Goat	1:200
**418**	XIAP	XIAP	XIAP	CST	2042	Rabbit	1:100
**419**	XPA	XPA	XPA	Santa Cruz	sc-56813	Mouse	1:75
**420**	XPF	XPF	ERCC4	Abcam	ab73720	Rabbit	1:100
**421**	XPG	ERCC5	ERCC5	Proteintech Group	11331-1-AP	Rabbit	1:250
**422**	XRCC1	XRCC1	XRCC1	CST	2735	Rabbit	1:20
**423**	YAP	YAP	YAP1	Santa Cruz	sc-376830	Mouse	1:300
**424**	YAP (phospho S127)	YAP_pS127	YAP1	CST	4911	Rabbit	1:250
**425**	YB1 (phospho S102)	YB1_pS102	YBX1	CST	2900	Rabbit	1:50
**426**	ZAP-70	ZAP-70	ZAP70	CST	2705	Rabbit	1:500

Table S6. Comparisons of RPPA outcomes of *MMTV-R26* ^{Met} cells belonging to “subtype A” and “subtype B” versus those from the non-tumorigenic cells.

Subtype A (MGT4+9+11) vs. non-tumorigenic (MGT2 & 7)

Protein	logFC	AveExpr	t	P.Value	adj.P.Val
P-Met_pY1234_Y1235	3,771668	-1,0119078	10,90563	6,84E-08	5,77E-06
E-Cadherin	3,468199	1,5253849	10,61925	9,35E-08	5,77E-06
Claudin-7	1,911199	1,0822876	10,33591	1,28E-07	6,33E-06
RSK	1,524384	-0,6111489	7,299833	6,18E-06	1,53E-04
CDK1	1,448314	-0,1166139	4,725375	4,02E-04	0,00183884
ARID1A	1,446252	0,3963761	4,852469	3,20E-04	0,00155111
Bim	1,416402	0,0453894	4,942895	2,73E-04	0,00142332
53BP1	1,269014	0,5378448	5,401039	1,23E-04	7,89E-04
Rad50	1,061401	0,2447965	5,410073	1,21E-04	7,89E-04
HES1	0,981841	0,0343482	2,795423	0,0152226	0,03357122
Src_pY416	0,958743	-0,2971786	6,696275	1,52E-05	2,50E-04
ACC_pS79	0,939118	-0,11619	2,665268	0,0195073	0,04054706
Connexin-43	0,895784	0,2361452	3,028428	0,0097416	0,02382356
FASN	0,887145	-0,1825017	5,620712	8,49E-05	7,23E-04
Src_pY527	0,883245	-0,1438668	4,22756	9,98E-04	0,00347248
Rb_pS807_S811	0,870722	-0,0627291	2,38951	0,0328075	0,06157929
ACC1	0,865206	0,0651942	4,657714	4,54E-04	0,00196783
Cox2	0,828262	0,6923661	1,422211	0,1786289	0,24648788
4E-BP1_pS65	0,794498	0,2176293	6,006903	4,49E-05	5,28E-04
GCN5L2	0,78403	0,1609002	5,658902	7,96E-05	7,23E-04
Akt_pS473	0,706044	-0,29258	4,279576	9,06E-04	0,00334177
c-Jun_pS73	0,687669	0,1712913	6,835796	1,23E-05	2,17E-04
p90RSK_pT573	0,660934	-0,1175991	5,401963	1,23E-04	7,89E-04
PARP	0,639323	0,189449	6,210382	3,24E-05	4,44E-04
Akt_pT308	0,631021	-0,1776652	3,715662	0,0026124	0,00750295
HER3	0,584665	0,4124621	4,245167	9,66E-04	0,00340895
C-Raf_pS338	0,560357	-6,44E-04	5,077137	2,15E-04	0,0011679
PKC-b-II_pS660	0,557357	-0,279136	5,524983	9,97E-05	7,73E-04
IRS1	0,543443	0,197673	1,828737	0,0905815	0,1399653
VASP	0,536384	-0,1346535	3,548721	0,0035909	0,00996573
c-Myc	0,536074	0,1818384	5,782582	6,48E-05	6,96E-04
beta-Catenin	0,520031	0,2023753	3,444927	0,0043796	0,01163174
eIF4E	0,500608	-0,0795851	7,337273	5,85E-06	1,53E-04
4E-BP1	0,49076	0,0226499	2,387859	0,0329088	0,06157929
Notch1	0,475117	-0,2848443	2,481032	0,027637	0,0541772
p16_INK4a	0,460764	0,1754814	2,047757	0,061451	0,10328628
Caspase-3	0,432888	0,0986794	7,15535	7,63E-06	1,63E-04
RPA32_pS4_S8	0,422635	-0,0877175	4,922764	2,83E-04	0,00142496
NDRG1_pT346	0,421693	0,3033009	0,904163	0,3824397	0,47708386
RBM15	0,419121	0,4528309	1,104033	0,2896937	0,37859436
Paxillin	0,399942	-0,0701099	8,728997	8,82E-07	3,63E-05
PAICS	0,395387	0,2794241	2,228145	0,0442483	0,07751302
Bcl-xL	0,392312	0,1383302	4,062771	0,001357	0,00424273
14-3-3_zeta	0,388742	-0,0596922	4,658633	4,53E-04	0,00196783
Bid	0,381066	0,1070465	3,283919	0,0059639	0,01534459
HER2_pY1248	0,3781	0,0388001	2,664526	0,0195348	0,04054706
FAK	0,377805	-0,0713128	5,6286	8,38E-05	7,23E-04
Histone-H3	0,376417	0,0086542	3,004335	0,0102027	0,02470651
p38_pT180_Y182	0,363108	0,0857146	5,278316	1,52E-04	9,11E-04
Shc_pY317	0,352321	-0,2506858	4,352649	7,92E-04	0,00305704
c-Kit	0,345628	0,1667719	5,504168	1,03E-04	7,73E-04
AMPK_alpha	0,343258	-0,1949831	3,730602	0,0025393	0,00737877
PRAS40_pT246	0,333433	-0,1478686	1,602546	0,1331629	0,19578124
Smad3	0,32264	0,1278642	4,631279	4,76E-04	0,00202851
HSP70	0,316225	0,0233271	5,187279	1,78E-04	0,00102115
WIPI1	0,299004	0,2067124	1,857936	0,0860821	0,13542849
PDK1	0,288736	-0,0743783	4,052317	0,0013838	0,00427252
PAK1	0,288257	-0,0960324	1,206203	0,2493386	0,3329007
Bax	0,282279	-0,0402982	0,891662	0,3888601	0,48265551
DJ-1	0,28216	0,1007253	4,336833	8,16E-04	0,00309901
IR-b	0,271336	0,0634504	1,320554	0,2095391	0,28437448
Lck	0,270305	0,0645107	2,784147	0,0155539	0,0339984
Merlin	0,25649	0,0583102	2,001088	0,0668193	0,11002916
TUFM	0,255489	0,0967286	3,668656	0,0028567	0,00811037
Myt1	0,251577	-0,0590917	3,421506	0,0045805	0,01203606
PDK1_pS241	0,24966	0,0705303	1,828203	0,0906658	0,1399653
ATM	0,248594	0,0743071	1,061881	0,3077141	0,40002836
ER-alpha_pS118	0,247404	0,1571599	4,543131	5,59E-04	0,00222667
Bad_pS112	0,246678	-0,1849946	3,61229	0,0031806	0,00892728
Caspase-7_cleavedD19	0,233133	-0,1285137	2,305651	0,0383474	0,06814244
Bak	0,22832	-0,0479864	3,170981	0,0074085	0,01848373
Chk2_pT68	0,22803	-0,034172	2,891423	0,0126689	0,02897413
Wee1_pS642	0,223293	0,1465578	2,56915	0,0234061	0,04700253
Elk1_pS383	0,217788	-0,0541057	4,129958	0,0011969	0,00394175
Cdc2_pY15	0,208341	0,0433277	1,556484	0,1437134	0,20758604
b-Actin	0,207393	0,1279735	1,793327	0,0963213	0,14686021
EGFR_pY1173	0,201168	-0,0426149	4,551783	5,50E-04	0,00222667
STING	0,200475	0,1379571	0,41888	0,6821762	0,75222104
ATR_pS428	0,192485	0,00212	3,993173	0,0015462	0,00465732

Subtype B (MGT13) vs. non-tumorigenic (MGT2 & 7)

Protein	logFC	AveExpr	t	P.Value	adj.P.Val
P-Met_pY1234_Y1235	4,563148	-1,6674278	44,05051	1,05E-09	2,60E-07
RSK	2,114524	-0,7602672	6,313678	4,25E-04	0,00656047
Notch1	2,029212	0,2558908	10,54554	1,68E-05	5,19E-04
HES1	1,627067	0,0519271	6,696406	2,98E-04	0,0056618
Fibronectin	1,565388	0,9234731	10,57193	1,65E-05	5,19E-04
ACC_pS79	1,380298	-0,174792	3,366794	0,0122547	0,05132152
CDK1	1,366848	-0,5335007	3,823602	0,0067068	0,03524618
Rb_pS807_S811	1,304012	-0,1066745	2,532854	0,0395758	0,11217854
Akt_pS473	1,265382	-0,2390729	11,70825	8,45E-06	3,64E-04
IGFRb	1,122679	0,5041849	4,190776	0,004226	0,03070037
Bim	1,058487	-0,4815593	7,109478	2,07E-04	0,00451158
Src_pY416	1,017153	-0,5250009	18,32814	4,21E-07	5,08E-05
Akt_pT308	0,99835	-0,1866618	7,260346	1,82E-04	0,00448387
PKA-a	0,965586	0,2854551	17,31854	6,17E-07	5,08E-05
ACC1	0,852846	-0,1682892	3,899131	0,0060888	0,03379328
FASN	0,838035	-0,4375213	3,49291	0,0103442	0,0464548
LRP6_pS1490	0,671783	0,1657914	11,62867	8,84E-06	3,64E-04
p90RSK_pT573	0,607544	-0,3147928	3,325557	0,0129593	0,05334913
Shc_pY317	0,593491	-0,2402474	6,355699	4,08E-04	0,00656047
AMPK_pT172	0,585846	0,8578037	3,89801	0,0060975	0,03379328
Pdcd4	0,584895	0,116794	3,924916	0,0058923	0,03379328
PKC-b-II_pS660	0,563848	-0,4233496	5,389217	0,0010738	0,01386302
STING	0,544972	0,2327264	3,055622	0,0187941	0,06981802
MCT4	0,534748	0,5135805	0,88172	0,4076264	0,52833899
AMPK_alpha	0,481	-0,2264803	4,409915	0,003239	0,02424311
PRAS40_pT246	0,470494	-0,177067	8,623494	6,16E-05	0,00169047
IR-b	0,453404	0,0699186	1,657671	0,1420237	0,25420179
STAT5-alpha	0,451643	0,587122	2,638098	0,0339911	0,10494747
HER2_pY1248	0,445391	-0,0320792	4,41123	0,0032339	0,02424311
Tuberin	0,432484	0,1376954	4,905013	0,0018226	0,01911688
SLC1A5	0,412054	0,3457002	2,460839	0,0439391	0,11545689
Src_pY527	0,410403	-0,5794568	1,690426	0,1354647	0,24602776
Paxillin	0,406428	-0,1728096	7,041667	2,19E-04	0,00451158
Bad_pS112	0,397009	-0,1856245	4,420566	0,0031979	0,02424311
Caspase-7_cleavedD198	0,371346	-0,130765	3,639518	0,0085192	0,04125948
XRCC1	0,366656	0,0387943	3,039023	0,0192347	0,06986708
IGF1R_pY1135_Y1136	0,348902	0,1505626	3,239624	0,0145717	0,05900362
Bid	0,345436	-0,0087244	2,510036	0,0409076	0,11226863
Mcl-1	0,334732	0,3562144	2,893978	0,0235855	0,08205113
RIP	0,325051	0,1058554	1,440921	0,193453	0,30269376
ATM	0,31833	0,0386307	0,832604	0,4329695	0,55411127
COG3	0,306351	0,0145178	4,422202	0,0031917	0,02424311
14-3-3_zeta	0,305893	-0,197724	5,347493	0,0011225	0,01386302
eIF4E	0,300825	-0,2972351	4,907438	0,0018176	0,01911688
WIPI1	0,299748	0,1281728	3,925854	0,0058853	0,03379328
mTOR	0,29646	-0,0810146	4,04117	0,0050885	0,03307519
MAPK_pT202_Y204	0,294888	-0,0230378	2,702394	0,0309894	0,09801214
Chk2_pT68	0,285975	-0,0694781	3,509896	0,0101125	0,04625544
PAK1	0,283941	-0,1739056	1,541461	0,1677734	0,28383578
Wee1	0,282046	0,180511	1,947076	0,0932163	0,18604001
p21	0,275143	-0,1092629	3,873219	0,0062935	0,03379328
Myt1	0,267636	-0,1185596	3,748261	0,007392	0,03651662
FAK	0,266835	-0,2185122	3,366532	0,012259	0,05132152
YB1_pS102	0,248129	-0,0819382	3,803376	0,0068837	0,03529816
Caspase-3	0,244179	-0,0963645	4,070668	0,0049042	0,03273887
4E-BP1_pS65	0,236739	-0,2309479	1,380441	0,2105612	0,32505382
PLC-gamma2_pY759	0,23491	0,0072565	2,118022	0,0725491	0,1585807
Bak	0,215446	-0,11372	2,249705	0,0598096	0,14342683
UBAC1	0,214845	-0,092247	3,790107	0,0070025	0,03529816
Bax	0,208181	-0,146502	2,8076	0,026662	0,08780673
GATA6	0,201924	-0,0159812	2,874659	0,0242395	0,08315488
Cdc2_pY15	0,197787	-0,0161427	0,987127	0,3569608	0,49533325
PI3K-p85	0,197071	0,0344405	0,548609	0,6005859	0,70571753
GCN5L2	0,19554	-0,298087	0,966581	0,3664395	0,50283648
PARP	0,19536	-0,1694338	1,365077	0,2151167	0,32798665
Histone-H3	0,194263	-0,1686865	2,039951	0,081353	0,16967817
Stathmin	0,191596	-0,1444441	2,518617	0,0404014	0,11217854
ERCC5	0,189449	0,0877916	0,905446	0,3957795	0,52557809
beta-Catenin	0,171947	-0,083955	1,057484	0,3259429	0,46268912
EGFR_pY1173	0,169934	-0,1090561	2,744401	0,0291791	0,09360055
Elk1_pS383	0,169645	-0,1321767	2,395789	0,0483065	0,12300738
SDHA	0,16948	0,1233133	2,017755	0,0840447	0,17156231
PTEN	0,164032	0,2496951	0,94282	0,3776406	0,5125122
Connexin-43	0,158935	-0,3158979	0,379397	0,7158123	0,78931092
mTOR_pS2448	0,150508	-0,0301328	2,20943	0,0634461	0,14779565
Lck	0,150488	-0,0581287	1,887569	0,1016952	0,19935492
ATR_pS428	0,150049	-0,066832	2,208924	0,0634932	0,14779565
FOXO3a_pS318_S321	0,146765	-0,08466	2,59818	0,0360061	0,10715061
Tyro3	0,14106	-0,0157264	2,036651	0,0817478	0,16967817

DM-K9-Histone-H3	0,191106	-0,032935	2,955169	0,0112119	0,02688678
eEF2	0,187505	0,0403709	1,234271	0,2390563	0,32090713
Rictor	0,187175	-0,0121124	2,449399	0,0293286	0,05615625
eIF4G	0,186366	0,0353867	3,198586	0,0070258	0,017708
EGFR	0,182966	-0,0770142	2,536964	0,0248733	0,04954598
MSH6	0,182251	0,1297574	1,5399	0,1476837	0,21208063
MEK1_pS217_S221	0,17823	0,0065781	3,843738	0,0020492	0,00602553
XRCC1	0,173953	0,002085	1,931898	0,0755753	0,12362311
p27_pT198	0,17211	0,0723343	2,328813	0,0367341	0,06622864
Gab2	0,171561	0,0479602	0,624194	0,5433525	0,62422358
NAPSIN-A	0,170103	-0,1065433	2,9256	0,0118658	0,02764966
UBAC1	0,166895	-0,0687808	2,357465	0,0348277	0,06419738
Jagged1	0,158894	0,1041255	0,993534	0,3386726	0,43119655
TAZ	0,153525	0,1660362	2,347866	0,0354557	0,06439373
p53	0,147901	-0,0309641	1,44288	0,172831	0,24118219
Notch3	0,144246	0,0288049	2,942955	0,0114776	0,02699964
PAR	0,143159	0,7936152	0,264154	0,7958228	0,84364046
Stathmin	0,139431	-0,1300271	1,902231	0,079641	0,12857082
mTOR	0,136343	-0,1136771	1,652966	0,1223894	0,18321324
Aurora-B	0,133051	-0,0498797	1,806192	0,0941993	0,14451695
YB1_pS102	0,130054	-0,0982415	1,779569	0,0986378	0,14946961
PLC-gamma2_pY759	0,119652	-0,0105832	1,610908	0,131321	0,19539936
AMPK-a2_pS345	0,115932	-0,1005424	1,507819	0,1556308	0,22220123
Tuberin	0,115824	0,0325309	1,403352	0,1840597	0,25117539
Tyro3	0,092494	-0,0121462	1,858878	0,0859403	0,13542849
p27-Kip-1	0,091811	-0,0645913	2,350207	0,0353015	0,06439373
FOXO3a_pS318_S321	0,088969	-0,0859653	2,14559	0,0514697	0,08828487
Cyclin_D1	0,087443	-0,0020169	1,432363	0,175761	0,24389313
PEA15_pS116	0,085991	-0,0163314	0,631631	0,5386309	0,62422358
TIGAR	0,084525	0,0411889	0,976319	0,3468127	0,43929614
b-Catenin_pT41_S45	0,079037	0,0461708	1,01903	0,3268706	0,41832662
Chk1_pS296	0,071257	-0,0518607	1,16746	0,264095	0,34883135
GCLM	0,062402	-0,0037227	1,236505	0,2382525	0,32090713
Raptor	0,049775	0,0118633	0,593726	0,5629346	0,64075962
MDM2_pS166	0,04399	-0,1730021	0,553736	0,5892041	0,66758449
Akt	0,039874	-0,0891319	0,293054	0,7741296	0,82774897
DUSP4	0,037353	0,0838537	0,318001	0,7555598	0,81494878
MAPK_pT202_Y204	0,034884	-0,1252682	0,241066	0,8132833	0,85481267
Rab11	0,032688	-0,048905	0,381195	0,7092465	0,77514998
C-Raf	0,030052	-0,0492449	0,301115	0,7681125	0,82488607
Sox2	0,028709	0,2078454	0,150345	0,8828119	0,90329557
NF-kB-p65_pS536	0,027659	0,0087904	0,245802	0,8096926	0,85467557
YAP	0,026607	-0,0505988	0,45738	0,654985	0,72874462
cIAP	0,014036	-0,0504367	0,346067	0,7348567	0,79609472
Ets-1	0,006433	0,0497873	0,147502	0,8850102	0,90329557
Wee1	0,005595	0,0635073	0,032864	0,9742851	0,97428513
IGF1R_pY1135_Y1136	0,003058	0,0031501	0,042925	0,9664169	0,9703454
eIF4E_pS209	-0,0069	0,0259484	-0,10684	0,9165562	0,92403831
U-Histone-H2B	-0,01159	-0,0096519	-0,15162	0,8818259	0,90329557
JNK2	-0,01277	0,0018004	-0,1781	0,8614012	0,8939752
A-Raf	-0,01623	0,0166796	-0,14277	0,8886742	0,9033026
XPF	-0,02234	-0,1081235	-0,39114	0,7020631	0,7707093
HER3_pY1289	-0,0246	-0,0026466	-0,26741	0,7933662	0,84364046
PI3K-p110-a	-0,02523	-0,036344	-0,46261	0,6513318	0,72874462
eEF2K	-0,02755	0,178513	-0,16833	0,8689247	0,89801002
PI3K-p85	-0,02841	-0,069686	-0,13565	0,8941876	0,90518167
D-a-Tubulin	-0,03121	-0,0398156	-0,53242	0,6034589	0,68061341
SLC1A5	-0,03636	0,143932	-0,19651	0,8472655	0,88301506
RIP	-0,03699	-0,0590641	-0,21562	0,8326489	0,87145884
MMP2	-0,03721	0,055784	-0,96445	0,3525077	0,44423161
PEA15	-0,03872	0,1718814	-0,35493	0,7283627	0,79253564
PKA-a	-0,04	-0,1560579	-0,45884	0,6539663	0,72874462
LRP6_pS1490	-0,04168	-0,1509694	-0,71962	0,4845572	0,58099818
mTOR_pS2448	-0,04959	-0,1289684	-0,42059	0,6809608	0,75222104
p21	-0,05003	-0,2618167	-0,69608	0,4986958	0,58824698
JNK_pT183_pY185	-0,05417	0,0593339	-0,62554	0,5424947	0,62422358
Slfn11	-0,05446	-0,0775038	-0,82271	0,425579	0,52038616
Smad1	-0,05639	0,0367162	-0,69372	0,500129	0,58824698
Rad51	-0,06129	0,2265507	-0,6108	0,5519147	0,6311247
Glutamate-D1-2	-0,06848	0,0224619	-0,73836	0,4734825	0,57048871
c-Abl	-0,07024	-0,0653876	-1,56494	0,1417235	0,20591584
IGFBP2	-0,07332	-0,0240207	-0,64764	0,528544	0,61580364
p70-S6K1	-0,07489	0,1299386	-0,82936	0,4219433	0,51850749
IRF-1	-0,07708	0,0617817	-1,4848	0,161554	0,22802199
PREX1	-0,0774	0,2077197	-1,05361	0,3113465	0,40263137
p38_MAPK	-0,09257	0,0649005	-0,80506	0,4353339	0,52969197
Creb	-0,09465	0,0546613	-2,20983	0,0457627	0,07960132
PKC-delta_pS664	-0,10128	-0,0486037	-2,32125	0,0372539	0,06667902
ERCC5	-0,10161	-0,0637429	-0,77106	0,4545252	0,55033195
14-3-3_beta	-0,10446	-0,0199976	-2,04436	0,0618276	0,10328628
GSK-3a-b_pS21_S9	-0,12418	0,0017529	-0,68567	0,505028	0,59119387
Mnk1	-0,12863	0,1504279	-1,1942	0,2538398	0,33708831
Pdcd4	-0,13265	-0,2257078	-0,91752	0,3756589	0,47100381
VEGFR-2	-0,13295	0,0340055	-0,86012	0,4053808	0,50064526

S6_pS240_S244	0,127725	0,4220785	1,371766	0,2131229	0,32696494
ARID1A	0,115095	-0,5555488	0,353378	0,7343676	0,80617243
DM-K9-Histone-H3	0,1107	-0,1177962	1,125894	0,2978848	0,43027804
PDK1	0,106406	-0,2286699	0,953741	0,3724603	0,50827461
p38_pT180_Y182	0,09872	-0,1233594	1,03424	0,3359452	0,46880484
XPF	0,095511	-0,0517258	1,156349	0,2860453	0,41560697
Raptor	0,088684	0,0154113	0,808989	0,4455471	0,56435964
c-Kit	0,086084	-0,0356159	0,919586	0,3888417	0,51915619
PI3K-p110-a	0,082661	0,016552	0,925104	0,3861591	0,51837655
Akt	0,081209	-0,0819331	1,179535	0,2772956	0,40527812
c-Abl	0,079637	0,0173695	1,262439	0,2478299	0,37010532
PAICS	0,07913	0,0396074	0,710801	0,5005479	0,61942666
Aurora-B	0,075079	-0,1098153	0,899516	0,3987164	0,52664673
GSK-3a-b_pS21_S9	0,07313	0,1190676	0,398842	0,7020784	0,77763844
Caveolin-1	0,070314	0,2043218	0,209433	0,840166	0,88684184
YAP	0,068369	-0,039718	1,111025	0,3038096	0,4362847
IGFBP2	0,065561	0,0548377	0,461112	0,658917	0,75348374
MSI2	0,060932	0,120869	0,599495	0,5680198	0,67129616
U-Histone-H2B	0,059832	0,0240155	0,674241	0,522123	0,63473657
MEK1_pS217_S221	0,053707	-0,0937947	0,882184	0,4073923	0,52833899
Chk1_pS296	0,051428	-0,079152	0,534241	0,6099673	0,71066945
p27_pT198	0,050474	-0,0251875	0,621983	0,5539641	0,66101032
DJ-1	0,044436	-0,0755722	0,670191	0,5245494	0,63473657
p53	0,040754	-0,1158909	0,463467	0,6573101	0,75348374
C-Raf	0,03794	-0,0537902	0,312643	0,7637999	0,82744992
Stat3	0,033749	0,4534121	0,347239	0,7387735	0,80742064
C-Raf_pS338	0,028811	-0,3762361	0,414808	0,6908904	0,7721716
NAPSIN-A	0,026572	-0,2129188	0,285877	0,7833742	0,84494947
cIAP	0,026387	-0,0488455	0,417351	0,6891158	0,7721716
Bcl-xL	0,024299	-0,1228564	0,436409	0,6758845	0,76229901
CD20	0,016336	0,1297929	0,200478	0,8468943	0,89013995
GCLM	0,006495	-0,0441405	0,082951	0,9362486	0,95240706
c-Jun_pS73	0,004896	-0,302689	0,055213	0,9575349	0,96535153
Smad3	0,003638	-0,0939425	0,028646	0,9779587	0,98193417
MDM2_pS166	0,001828	-0,2026732	0,01909	0,9853102	0,98531024
TUFM	-0,0077	-0,0834473	-0,08199	0,9369835	0,95240706
MERIT40_pS29	-0,01226	0,1136221	-0,08342	0,935886	0,95240706
Wee1_pS642	-0,01489	-0,0144094	-0,11372	0,9127006	0,94325128
ER-alpha_pS118	-0,01584	-0,0209075	-0,21508	0,835933	0,88616077
AR	-0,01759	0,1283755	-0,14103	0,8918789	0,92951097
Rictor_pT1135	-0,02078	0,1220186	-0,14653	0,8876933	0,92906879
YAP_pS127	-0,02338	0,2413937	-0,0691	0,946876	0,95851789
4E-BP1	-0,03111	-0,3304412	-0,11791	0,9094996	0,94325128
HER3_pY1289	-0,03615	-0,0011075	-0,25026	0,8096846	0,86576661
JNK2	-0,03974	-0,0063912	-0,6728	0,5229878	0,63473657
PR	-0,03991	0,2736279	-0,66643	0,5268057	0,63473657
Stat3_pY705	-0,04201	0,325379	-0,33924	0,7445334	0,81013109
PKC-delta_pS664	-0,04845	7,52E-04	-0,71805	0,4963418	0,6191738
FoxM1	-0,04982	0,0565277	-0,50459	0,6295739	0,72665769
b-Catenin_pT41_S45	-0,0504	-0,0301463	-0,40772	0,6958495	0,77421098
PDK1_pS241	-0,05081	-0,1240855	-0,22416	0,8291295	0,88273697
D-a-Tubulin	-0,05104	-0,0400841	-0,60807	0,5626368	0,66813119
eIF4G	-0,05348	-0,1165538	-0,70028	0,5066953	0,62265539
TIGAR	-0,05361	-0,0403032	-0,8807	0,4081404	0,52833899
Notch3	-0,05379	-0,0941086	-0,73702	0,4854356	0,60864262
Rictor	-0,05741	-0,1662992	-0,92512	0,3861535	0,51837655
NDRG1_pT346	-0,06058	-0,0146028	-0,08866	0,9318744	0,95240706
Ets-1	-0,06377	0,0180023	-1,03536	0,3354585	0,46880484
Slfn11	-0,06517	-0,0677315	-0,79237	0,454551	0,572827
Rad50	-0,07267	-0,5211625	-0,27644	0,7903156	0,84873025
RPA32_pS4_S8	-0,07986	-0,4145376	-0,64032	0,5426597	0,65066474
p44-42-MAPK	-0,0827	0,0931581	-0,81917	0,4400946	0,56032659
eEF2	-0,08428	-0,1255586	-0,4373	0,6752698	0,76229901
CD134	-0,08434	0,1674613	-1,35055	0,2195032	0,33262135
p27-Kip-1	-0,08497	-0,1645682	-1,44029	0,193626	0,30269376
PKM2	-0,08995	0,0966772	-0,51679	0,6214707	0,72067255
A-Raf	-0,09276	-0,0118433	-0,70906	0,50156	0,61942666
G6PD	-0,09602	0,0621284	-0,87988	0,4085536	0,52833899
Cyclin_D1	-0,10112	-0,1058925	-1,48559	0,1816317	0,2990869
14-3-3_beta	-0,10458	0,0075003	-1,46237	0,1876933	0,30269376
OCT4	-0,11116	0,1129347	-2,05236	0,0798856	0,16864745
Hexokinase-II	-0,11298	0,1487511	-0,84776	0,4250306	0,5467841
eIF4E_pS209	-0,11764	-0,0196953	-1,19881	0,2701927	0,39872105
AMPK-a2_pS345	-0,11883	-0,23173	-1,54239	0,1675505	0,28383578
MSH6	-0,12202	-0,0487089	-1,25977	0,2487348	0,37010532
Creb	-0,12371	0,0671715	-2,12557	0,0717497	0,15823379
TAZ	-0,12866	0,0046104	-1,61369	0,1513017	0,26693938
WIPI2	-0,12954	0,2692593	-0,44044	0,6731039	0,76229901
cdc25C	-0,13853	0,2915575	-1,05224	0,328177	0,46319843
MMP2	-0,13915	0,0219057	-2,56676	0,0376792	0,11079485
Claudin-7	-0,14004	-0,0067117	-1,94576	0,0933966	0,18604001
IRS1	-0,14048	-0,2387615	-0,54511	0,6028599	0,70571753
N-Cadherin	-0,14183	0,0073635	-1,93337	0,0951062	0,1879298
Rab25	-0,14206	0,0114982	-1,60086	0,154112	0,26996927

GATA6	-0,1389	-0,1986784	-2,02354	0,0641846	0,10639995
Jak2	-0,14217	0,0189833	-2,3765	0,0336141	0,06242619
PAK4	-0,14445	0,0926862	-1,13604	0,2765507	0,36334052
OCT4	-0,15027	0,0565422	-3,86126	0,0019824	0,0058994
N-Cadherin	-0,1619	-0,0439391	-1,45835	0,1685958	0,23660887
P-Cadherin	-0,16595	0,1045818	-2,04382	0,0618881	0,10328628
AR	-0,1731	0,0160716	-1,84732	0,0876941	0,13709139
COG3	-0,1775	-0,2396591	-1,48881	0,1605088	0,2278487
CD20	-0,18028	-0,0020163	-2,72244	0,0174965	0,03757937
MERIT40_pS29	-0,18033	-0,0059637	-1,87899	0,0829643	0,13306605
MSI2	-0,18129	-0,0307509	-2,63475	0,0206712	0,04240027
FoxM1	-0,18286	-0,0487133	-2,49443	0,026949	0,05325125
ZAP-70	-0,20149	0,0909259	-2,62787	0,0209426	0,04240027
Rab25	-0,20263	-0,0678976	-2,90888	0,0122521	0,02828296
INPP4b	-0,20465	0,0035981	-4,54366	5,58E-04	0,00222667
ER-alpha	-0,20535	0,0807164	-4,26333	9,34E-04	0,00334396
FAK_pY397	-0,21048	0,3062533	-0,70205	0,4950855	0,58791402
FoxO3a	-0,21308	0,0217331	-3,44668	0,0043649	0,01163174
CD134	-0,21867	0,0522217	-4,13803	0,001179	0,00393535
ATM_pS1981	-0,21994	0,0095047	-4,08413	0,0013038	0,00412884
Caveolin-1	-0,2202	0,0217395	-0,70632	0,4925168	0,58768911
TTF1	-0,22137	0,0775741	-4,47425	6,34E-04	0,00248458
XBP1	-0,22244	0,0394977	-5,52061	1,00E-04	7,73E-04
Rictor_pT1135	-0,22379	-0,0240085	-1,66556	0,1198209	0,18046192
PKM2	-0,23357	-0,026473	-1,4102	0,1820713	0,24984234
cdc25C	-0,23387	0,1890208	-1,90632	0,0790684	0,12848621
Tuberin_pT1462	-0,23808	0,0478807	-3,2277	0,0066437	0,01691757
PD-L1	-0,25607	0,0026159	-6,13692	3,64E-05	4,69E-04
Hexokinase-II	-0,25661	0,0195186	-3,05028	0,0093414	0,02307335
Beclin	-0,26967	0,0575019	-5,26686	1,55E-04	9,11E-04
SDHA	-0,27331	-0,1385362	-4,27001	9,23E-04	0,00334396
B-Raf_pS445	-0,27469	0,0216048	-2,79904	0,0151178	0,03357122
MIF	-0,28066	-0,0150759	-4,2845	8,98E-04	0,00334177
G6PD	-0,28562	-0,094455	-2,45428	0,0290612	0,056079
DM-Histone-H3	-0,28801	0,0980327	-4,60032	5,04E-04	0,0021091
BRD4	-0,30036	-0,0010115	-4,09225	0,0012842	0,00411945
PR	-0,30594	0,0789249	-6,64866	1,63E-05	2,52E-04
CD4	-0,3083	0,0887386	-4,15699	0,0011381	0,00385073
WIPI2	-0,31893	0,1039822	-1,59248	0,1354101	0,19790704
B7-H4	-0,33225	0,0212483	-4,74741	3,86E-04	0,00180088
p44-42-MAPK	-0,33678	-0,104557	-2,86825	0,0132437	0,02973803
TSC1	-0,33822	-0,0948599	-1,60718	0,13214	0,19544057
SHP-2_pY542	-0,35906	-0,0189077	-4,10303	0,0012586	0,0040904
Mcl-1	-0,36634	-0,0408638	-1,86243	0,0854074	0,13542849
TFAM	-0,38584	0,2122181	-2,6807	0,0189432	0,03999125
PDHK1	-0,38987	-0,0669717	-6,22144	3,18E-05	4,44E-04
Fibronectin	-0,40037	-0,0245861	-1,03917	0,3177623	0,40878793
IGFRb	-0,40486	-0,2572525	-2,41451	0,03131	0,05948891
SOD2	-0,42029	0,0561037	-8,10914	1,99E-06	6,15E-05
PTEN	-0,44637	-0,1296311	-2,87312	0,0131207	0,02973227
LC3A-B	-0,44985	0,1000738	-2,23768	0,0434784	0,07670833
PMS2	-0,45375	0,2736831	-2,94846	0,0113571	0,02697308
MEK1	-0,45539	0,2784054	-3,46083	0,0042482	0,01153095
Atg7	-0,46796	0,0609753	-2,73123	0,0172061	0,03727985
Heregulin	-0,49019	-0,0687039	-2,46804	0,0283203	0,05507965
YAP_pS127	-0,49942	-0,0943395	-2,17194	0,049053	0,0847279
Collagen_VI	-0,50292	0,030011	-5,13438	1,95E-04	0,00109412
B-Raf	-0,51067	0,0724391	-5,4379	1,16E-04	7,89E-04
ULK1_pS757	-0,51347	0,0762755	-7,12813	7,94E-06	1,63E-04
Atg3	-0,51804	-0,0841261	-6,85944	1,18E-05	2,17E-04
Stat3_pY705	-0,54483	-0,0338044	-5,75694	6,76E-05	6,96E-04
Axl	-0,56846	-0,0268375	-8,15267	1,88E-06	6,15E-05
HSP27_pS82	-0,59208	0,1358536	-4,68489	4,32E-04	0,00194184
LDHA	-0,65569	0,079271	-4,93506	2,77E-04	0,00142332
STAT5-alpha	-0,67924	-0,0766854	-4,76584	3,74E-04	0,00177584
Gys_pS641	-0,68198	-0,0642531	-4,86307	3,14E-04	0,00155111
Glutaminase	-0,68524	-0,0335895	-4,03746	0,0014229	0,00433884
MYH11	-0,68998	0,094718	-3,28948	0,0059006	0,01534159
S6_pS240_S244	-0,6902	-0,1104895	-2,13149	0,0528086	0,08995675
TFRC	-0,69256	0,4027866	-3,52715	0,003742	0,01026971
PKC-alpha	-0,72642	-0,0970691	-5,45213	1,13E-04	7,89E-04
Granzyme-B	-0,78897	-0,0597221	-10,6371	9,17E-08	5,77E-06
p70S6K_pT389	-0,81525	-0,0130179	-4,20323	0,0010443	0,00358243
Cyclin_B1	-0,87661	0,244537	-5,39404	1,25E-04	7,89E-04
AMPK_pT172	-0,8829	-0,0045108	-6,11099	3,80E-05	4,69E-04
Gys	-0,90761	-0,0679323	-5,7821	6,49E-05	6,96E-04
Cox-IV	-0,94707	0,1957551	-5,62421	8,44E-05	7,23E-04
Myosin-IIa_pS1943	-0,99755	0,160807	-5,07158	2,18E-04	0,0011679
PLK1	-1,01791	0,1467133	-5,59318	8,89E-05	7,32E-04
MCT4	-1,02875	-0,4278113	-2,6302	0,0208504	0,04240027
Stat3	-1,03562	-0,278021	-5,65456	8,02E-05	7,23E-04
TRIM25	-1,08168	0,1025633	-5,34429	1,36E-04	8,37E-04
S6_pS235_S236	-1,21966	-0,0407079	-2,6995	0,0182779	0,03891927
PDGFR-b	-3,45067	-0,6152202	-14,695	1,90E-09	4,69E-07

ZAP-70	-0,14738	0,1672586	-1,76888	0,1209039	0,22568701
PDHK1	-0,14793	0,1395381	-1,46221	0,1877337	0,30269376
Atg3	-0,14802	0,2110792	-1,41202	0,2014673	0,31297125
XBP1	-0,14841	0,1298931	-2,51885	0,0403879	0,11217854
DM-Histone-H3	-0,15033	0,2330026	-2,06291	0,0786587	0,16835293
IRF-1	-0,15225	0,0498924	-2,39699	0,0482217	0,12300738
ATM_pS1981	-0,16042	0,0930213	-1,84521	0,1081839	0,20876115
Jak2	-0,16069	0,0485434	-2,48551	0,0423908	0,11506074
FoxO3a	-0,16263	0,0995509	-2,81837	0,0262562	0,08763892
p38_MAPK	-0,16318	0,0590571	-1,51641	0,173862	0,29016159
JNK_pT183_pY185	-0,16694	0,0252905	-1,63531	0,1466713	0,26063165
RBM15	-0,16872	0,0903623	-2,20324	0,0640248	0,14779565
Rab11	-0,17184	-0,1451814	-1,73941	0,1261882	0,23260071
Collagen_VI	-0,17461	0,3033535	-1,10641	0,3056669	0,43641459
HER3	-0,1871	-0,0724925	-2,16897	0,0673238	0,14981071
PD-L1	-0,18744	0,0995665	-3,17387	0,0159508	0,06155995
Smad1	-0,18966	-0,0055285	-3,21912	0,0149875	0,05970839
INPP4b	-0,19469	0,0618413	-2,99018	0,0205961	0,07372812
B7-H4	-0,19824	0,1663108	-1,85572	0,1065382	0,20720414
Mnk1	-0,20086	0,1533918	-1,46557	0,1868452	0,30269376
Rad51	-0,20229	0,1822849	-2,40296	0,0478038	0,12300738
MEK1	-0,20639	0,5052242	-1,19607	0,2711949	0,39872105
Tuberin_pT1462	-0,21457	0,1207483	-2,28442	0,0568454	0,13765495
PEA15_pS116	-0,21797	-0,1692776	-1,58466	0,1577332	0,27352611
B-Raf_pS445	-0,21915	0,1178505	-2,76894	0,0281732	0,09156284
CD4	-0,22355	0,2063683	-2,0594	0,0790645	0,16835293
EGFR	-0,23428	-0,3040888	-1,44886	0,1913027	0,30269376
LDHA	-0,23618	0,4319941	-1,51999	0,1729796	0,29016159
c-Myc	-0,23945	-0,2919121	-1,83696	0,1094939	0,20931118
DUSP4	-0,23995	-0,0448429	-1,69424	0,1347189	0,24602776
Beclin	-0,2442	0,139538	-3,88753	0,0061795	0,03379328
53BP1	-0,24702	-0,4465708	-1,76536	0,1215238	0,22568701
HSP70	-0,25318	-0,3041033	-2,59833	0,0359985	0,10715061
Glutamate-D1-2	-0,25518	-0,0394912	-2,01831	0,0839767	0,17156231
Granzyme-B	-0,2555	0,3769877	-2,69438	0,031348	0,09801214
Gys	-0,25676	0,4503697	-0,97707	0,3615775	0,49893661
BRD4	-0,2582	0,0962749	-2,32405	0,0536424	0,13249685
ER-alpha	-0,25858	0,1120595	-4,53533	0,0027905	0,02424311
TTF1	-0,25943	0,1196475	-4,01472	0,0052602	0,0333148
Axl	-0,26673	0,252402	-3,6237	0,0086982	0,04131634
TFAM	-0,27437	0,3617507	-1,67315	0,1388871	0,25040237
Sox2	-0,27551	0,0698959	-1,448	0,1915353	0,30269376
SHP-2_pY542	-0,28024	0,1095672	-2,47284	0,0431787	0,11545689
SOD2	-0,2808	0,2267275	-4,88818	0,0018575	0,01911688
Jagged1	-0,28263	-0,1270044	-2,2353	0,0610854	0,14507791
MIF	-0,29557	0,0525548	-4,60683	0,0025659	0,02424311
p70-S6K1	-0,29743	0,0543159	-4,58138	0,0026435	0,02424311
eEF2K	-0,32344	0,0589671	-2,17741	0,0664964	0,14931476
PREX1	-0,33267	0,1187313	-3,05014	0,0189385	0,06981802
ULK1_pS757	-0,33353	0,2888177	-3,99062	0,0054222	0,03348217
P-Cadherin	-0,33443	0,0761427	-2,94261	0,0220207	0,07770164
PEA15	-0,34867	0,0492564	-2,18148	0,0661002	0,14931476
VASP	-0,35052	-0,6562173	-2,5361	0,03939	0,11217854
S6_pS235_S236	-0,36186	0,6485896	-1,32262	0,2281608	0,34363247
Gys_pS641	-0,36883	0,2498172	-1,5819	0,1583572	0,27352611
b-Actin	-0,37532	-0,1764572	-5,26931	0,0012205	0,01435583
NF-kB-p65_pS536	-0,40735	-0,1849384	-4,54176	0,0027694	0,02424311
Merlin	-0,44466	-0,3098276	-4,07534	0,0048757	0,03273887
VEGFR-2	-0,44476	-0,0645609	-2,46791	0,0434894	0,11545689
PAK4	-0,45249	-0,0012319	-2,34393	0,0521064	0,13000281
Atg7	-0,45482	0,1900279	-1,79011	0,1172291	0,22103502
Glutaminase	-0,47371	0,2377846	-2,51829	0,0404206	0,11217854
LC3A-B	-0,47688	0,2071291	-1,44397	0,1926244	0,30269376
PMS2	-0,48922	0,378151	-2,08762	0,0758579	0,16435883
TFRC	-0,49996	0,6679799	-1,83278	0,1101638	0,20931118
B-Raf	-0,50455	0,2097444	-6,12404	5,10E-04	0,00740602
TSC1	-0,50695	-0,0779737	-1,56969	0,1611479	0,27641343
p70S6K_pT389	-0,52884	0,3247387	-1,98594	0,0880579	0,17828111
Heregulin	-0,57913	0,0224571	-2,18899	0,0653759	0,14931476
MYH11	-0,66165	0,2888351	-2,30342	0,055286	0,13520441
HSP27_pS82	-0,68487	0,2522355	-4,0859	0,0048119	0,03273887
FAK_pY397	-0,6886	0,1568537	-6,40614	3,89E-04	0,00656047
PKC-alpha	-0,71319	0,1001862	-13,2164	3,78E-06	2,34E-04
Cyclin_B1	-0,73721	0,5354757	-2,85891	0,0247867	0,0838673
E-Cadherin	-0,75573	-1,1995603	-1,49073	0,180314	0,29890978
Cox-IV	-0,80271	0,5074037	-3,0851	0,0180379	0,06854393
PLK1	-0,8331	0,494379	-3,20507	0,0152797	0,05990612
Myosin-IIa_pS1943	-0,9402	0,4484787	-3,38942	0,0118857	0,05132152
TRIM25	-0,9402	0,4484787	-3,38942	0,0118857	0,05132152
Gab2	-1,14244	-0,560432	-3,57162	0,0093168	0,04341993
p16_INK4a	-1,29666	-0,6992205	-5,94748	6,06E-04	0,00831592
Cox2	-1,35992	-0,4638683	-2,61733	0,0350245	0,10680296
PAR	-1,45606	0,0704788	-2,36477	0,0505441	0,12739176
PDGFR-b	-1,75802	1,0202718	-4,44228	0,0031161	0,02424311

Subtype A vs. MGT2

Protein	logFC	AveExpr	t	P.Value	adj.P.Val
P-Met_pY1	3,637657	-0,512787	7,504304	1,24E-05	4,37E-04
E-Cadherin	2,858419	2,07281	9,734716	1,02E-06	1,22E-04
Claudin-7	1,849309	1,334111	7,172082	1,88E-05	4,90E-04
NDRG1_pT	1,59218	0,143565	4,439983	0,001011	0,0078003
53BP1	1,390249	0,675538	4,255926	0,001372	0,0089284
HES1	1,388026	0,084085	3,031517	0,011495	0,0359412
Src_pY527	1,271446	-0,103271	5,51767	1,86E-04	0,0025465
Bim	1,1928	0,264333	3,052958	0,011065	0,0350382
RSK	0,955445	-0,315825	7,968723	7,06E-06	3,26E-04
Src_pY416	0,946306	-0,174236	4,702663	6,59E-04	0,0060253
4E-BP1	0,931205	0,004343	4,626002	7,46E-04	0,0063553
ARID1A	0,902423	0,6773	2,706231	0,020532	0,0533842
CDK1	0,841402	0,176039	2,594209	0,025065	0,0612974
Akt_pT308	0,828394	-0,134122	3,761853	0,003179	0,0153978
p16_INK4a	0,821494	0,167892	3,066624	0,010799	0,0350382
VASP	0,732469	-0,102788	3,829014	0,002831	0,0145695
Gab2	0,711829	-0,028675	2,434552	0,033271	0,0760914
IR-b	0,692957	0,020946	3,352691	0,006502	0,024048
PKC-b-II_p!	0,687287	-0,232603	5,540261	1,79E-04	0,0025465
HER3	0,684612	0,467884	3,693476	0,003579	0,0164203
Rad50	0,638681	0,455258	3,340458	0,006644	0,0241119
PDK1_pS24	0,62223	0,034216	8,196864	5,40E-06	3,26E-04
Akt_pS473	0,603119	-0,184994	2,678357	0,021578	0,0549461
c-Jun_pS73	0,585643	0,276401	4,455335	9,85E-04	0,0078003
4E-BP1_pS	0,535982	0,364639	3,84237	0,002767	0,0145413
ACC1	0,510477	0,238597	2,540506	0,027575	0,0648665
HER2_pY12	0,504194	0,063467	2,666076	0,022055	0,0555883
FASN	0,501761	-7,63E-04	4,079585	0,001845	0,0109818
C-Raf_pS33	0,500747	0,080728	3,308351	0,007032	0,0248118
PAICS	0,500277	0,310122	2,043257	0,065897	0,136744
p38_pT180	0,481667	0,109864	6,322686	5,82E-05	0,0010269
GCN5L2	0,47722	0,315373	3,78688	0,003045	0,0153479
STING	0,468437	0,114471	0,704499	0,495859	0,612386
PARP	0,461149	0,302319	3,968254	0,00223	0,0122401
eIF4E	0,451018	-0,007555	4,968384	4,31E-04	0,004632
PDK1	0,445608	-0,066556	7,13043	1,98E-05	4,90E-04
Caspase-3	0,438372	0,152172	5,332925	2,45E-04	0,0031884
14-3-3_zet	0,423332	-0,017049	3,691773	0,00359	0,0164203
Paxillin	0,41394	-0,022313	6,874556	2,76E-05	6,21E-04
FAK	0,407971	-0,029242	5,123623	3,38E-04	0,004157
Histone-H3	0,385838	0,054322	2,196682	0,050528	0,1114322
p90RSK_pT	0,378022	0,017034	3,531836	0,004745	0,01947
Bax	0,368331	-0,020412	0,826717	0,426078	0,5396982
Bcl-xL	0,364332	0,192799	2,719429	0,020055	0,0526977
Jagged1	0,35794	0,087936	1,749995	0,1081	0,1964232
c-Myc	0,357068	0,281863	3,662234	0,003779	0,016971
Shc_pY317	0,338664	-0,203854	3,023125	0,011668	0,0360265
Caveolin-1	0,334515	-0,106836	0,950612	0,362349	0,4775705
RBM15	0,334174	0,521032	0,624377	0,545193	0,6474162
PRAS40_pT	0,323953	-0,104174	1,103932	0,293333	0,4188231
WIPI1	0,304193	0,243406	1,358826	0,201582	0,317138
TUFM	0,300465	0,120711	3,217371	0,008261	0,0283398
beta-Caten	0,289844	0,309686	1,608168	0,136277	0,2404317
ER-alpha_p	0,286177	0,181252	4,07261	0,001867	0,0109818
c-Kit	0,279571	0,222288	4,388641	0,0011	0,0079908
HSP70	0,27151	0,071262	3,377812	0,00622	0,0240053
DJ-1	0,265733	0,139229	2,994441	0,01228	0,0369909
RPA32_pS4	0,261734	-0,005264	2,862701	0,015532	0,044098
PLC-gamma	0,260835	-0,021192	3,350843	0,006523	0,024048
ACC_pS79	0,251971	0,126956	0,670382	0,516529	0,6254047
MSH6	0,246726	0,140975	1,52971	0,154501	0,2651031
b-Actin	0,245096	0,147224	1,529492	0,154554	0,2651031
AMPK_alph	0,241763	-0,133322	2,038531	0,066434	0,136744
Bid	0,241507	0,180387	1,811547	0,097594	0,1824368
eIF4G	0,239594	0,049167	3,538797	0,004687	0,01947
Bad_pS112	0,238065	-0,152378	2,575245	0,025924	0,0627778

Subtype B vs. MGT 2

Protein	logFC	AveExpr	t	P.Value	adj.P.Val
P-Met_pY1	4,429137	-0,83157	57,94388	2,68E-10	6,62E-08
HES1	2,033252	0,168379	33,24106	1,09E-08	9,01E-07
Notch1	1,710942	0,731063	31,59293	1,54E-08	9,49E-07
Fibronectin	1,599821	1,178052	9,284537	4,58E-05	4,52E-04
IGFRb	1,571705	0,517034	24,26581	8,87E-08	3,13E-06
MCT4	1,567953	0,191969	17,06514	9,06E-07	1,97E-05
RSK	1,545585	-0,170202	29,36662	2,50E-08	1,23E-06
Akt_pT308	1,195723	-0,094465	13,88509	3,49E-06	5,74E-05
Akt_pS473	1,162458	0,01902	12,2847	7,70E-06	1,06E-04
NDRG1_pT	1,109908	-0,493183	19,43433	3,85E-07	1,06E-05
Src_pY416	1,004716	-0,345657	16,91783	9,59E-07	1,97E-05
PKA-a	0,945893	0,458861	16,1482	1,30E-06	2,47E-05
IR-b	0,875025	-0,021004	6,686059	3,40E-04	0,0020021
Bim	0,834885	-0,210663	10,30311	2,37E-05	2,93E-04
STING	0,812935	0,218965	8,125268	1,05E-04	9,25E-04
Src_pY527	0,798604	-0,664382	7,787181	1,36E-04	0,0010837
CDK1	0,759937	-0,056419	12,81882	5,85E-06	9,03E-05
LRP6_pS14	0,702505	0,268665	11,88844	9,51E-06	1,24E-04
PKC-b-II_p!	0,693778	-0,378662	9,59041	3,73E-05	4,39E-04
ACC_pS79	0,693151	0,336689	7,578502	1,60E-04	0,0012387
AMPK_pT1	0,684977	0,918582	4,163988	0,004651	0,0149183
SLC1A5	0,662634	0,316106	7,351803	1,93E-04	0,0013138
Caveolin-1	0,625031	-0,005511	6,850821	2,95E-04	0,0018659
Shc_pY317	0,579833	-0,133043	5,338815	0,001242	0,0061373
HER2_pY12	0,571485	-0,006164	8,016517	1,14E-04	9,50E-04
YAP_pS127	0,550479	0,007843	9,450377	4,10E-05	4,52E-04
ERCC5	0,506844	-0,006689	4,430638	0,003387	0,0124122
ACC1	0,498117	0,119805	6,114026	5,75E-04	0,0032184
IGF1R_pY1	0,494727	0,152044	7,286968	2,04E-04	0,0013228
PRAS40_pT	0,461013	-0,092619	7,99944	1,15E-04	9,50E-04
Tuberin	0,455819	0,202501	5,167735	0,001488	0,0068051
FASN	0,452651	-0,139704	5,22551	0,001399	0,0065509
MAPK_pT2	0,433972	-0,028119	5,953306	6,71E-04	0,0035067
Rb_pS807_	0,424155	0,468813	7,429753	1,81E-04	0,0013065
Paxillin	0,420426	-0,108736	6,765916	0,000317	0,0019426
4E-BP1	0,409332	-0,511953	6,093394	5,86E-04	0,0032184
Bad_pS112	0,388397	-0,114121	3,819178	0,007119	0,0204478
AMPK_alph	0,379505	-0,103425	3,568891	0,009808	0,0259636
PLC-gamma	0,376092	-0,008946	4,423101	0,003417	0,0124122
GSK-3a-b_p	0,371556	0,012234	5,765962	8,05E-04	0,0040593
Pdcd4	0,358724	0,30753	4,945374	0,001892	0,0080555
p21	0,354248	-0,093737	6,182004	5,39E-04	0,0030976
COG3	0,347162	0,050711	5,221219	0,001406	0,0065509
14-3-3_zet	0,340484	-0,159122	5,936645	6,81E-04	0,0035067
p90RSK_pT	0,324632	-0,097478	3,609566	0,009304	0,0255765
PDK1_pS24	0,321764	-0,281823	4,349438	0,003726	0,0129631
WIPI1	0,304937	0,177482	4,349768	0,003725	0,0129631
FAK	0,297001	-0,184835	5,008786	0,001765	0,007649
Bax	0,294233	-0,145235	5,048647	0,001691	0,0074564
Caspase-7_	0,293288	-0,035883	2,760869	0,029328	0,0619152
WIPI2	0,265893	0,088879	1,838137	0,110552	0,1870289
PDK1	0,263278	-0,273178	3,876257	0,006627	0,0197203
GATA6	0,252854	-0,001738	3,563194	0,009881	0,0259636
eIF4E	0,251234	-0,225829	4,329857	0,003814	0,0130825
Caspase-3	0,249663	-0,056699	3,822671	0,007088	0,0204478
YB1_pS102	0,247645	-0,039208	3,393642	0,012343	0,030293
UBAC1	0,237905	-0,064641	4,115195	0,004934	0,0154269
p38_pT180	0,217278	-0,15386	2,938324	0,022889	0,0504783
Myt1	0,209453	-0,049406	3,66189	0,008698	0,024413
XRCC1	0,207335	0,165378	2,451464	0,045538	0,086523
Bid	0,205877	0,106317	2,356773	0,052183	0,0969118
mTOR	0,205357	0,006248	3,857133	0,006787	0,0199584
Rictor_pT1	0,204619	0,028295	3,573809	0,009746	0,0259636
Histone-H3	0,203684	-0,139153	1,838476	0,110497	0,1870289
Chk2_pT68	0,203435	0,012562	3,233	0,015298	0,0363328
HER3_pY12	0,191942	-0,098544	3,108271	0,018117	0,0414349

p53	0,234861	-0,028158	1,717783	0,113997	0,2055273
Elk1_pS38	0,223288	-0,027692	3,153167	0,009258	0,0304905
EGFR	0,218885	-0,060514	3,053962	0,011045	0,0350382
ERCC5	0,21579	-0,13424	2,001351	0,070806	0,1432118
SLC1A5	0,214218	0,093795	0,925357	0,37476	0,4872195
TSC1	0,209308	-0,236983	1,440225	0,177827	0,2889694
Smad3	0,208068	0,189307	3,351871	0,006511	0,024048
Lck	0,206677	0,110104	1,586843	0,141034	0,2470589
Merlin	0,203843	0,100168	1,149809	0,27476	0,4057239
HER3_pY1	0,203492	-0,047216	2,946901	0,013366	0,0393039
Bak	0,193494	-0,012915	1,976923	0,073823	0,1461908
Myt1	0,193394	-0,016846	2,059608	0,06407	0,1341125
NAPSIN-A	0,193247	-0,08934	2,482779	0,030548	0,0705178
UBAC1	0,189955	-0,051966	1,969361	0,074781	0,1465416
Connexin-4	0,18699	0,477773	0,749889	0,469156	0,5852598
EGFR_pY11	0,182097	-0,013826	3,197102	0,008563	0,028975
Aurora-B	0,17813	-0,041328	1,975649	0,073983	0,1461908
PAK4	0,174688	0,016478	1,855111	0,09073	0,1723861
GSK-3a-b_	0,174242	-0,068138	0,819201	0,430172	0,5421047
MAPK_pT2	0,173967	-0,146165	0,912501	0,381192	0,4929548
ATR_pS428	0,173213	0,029853	2,740563	0,019313	0,0515209
Notch3	0,172453	0,041833	2,738095	0,019399	0,0515209
VEGFR-2	0,161657	-0,036295	0,967953	0,353999	0,4751501
MEK1_pS2	0,160906	0,032162	2,643838	0,022946	0,0572498
b-Catenin_	0,158045	0,041754	1,869614	0,088544	0,1695382
Notch1	0,156847	-0,167172	0,700098	0,498497	0,6125812
Caspase-7_	0,155075	-0,084976	1,138371	0,279303	0,4057239
TAZ	0,151172	0,185789	1,807623	0,098235	0,1824368
IGF1R_pY1	0,148883	-0,022979	2,229583	0,047709	0,106625
Chk2_pT68	0,14549	0,009538	1,474399	0,168586	0,2794674
Rictor	0,143098	0,019462	1,386335	0,193273	0,3079892
Tuberin	0,139159	0,042867	1,276209	0,228333	0,3481377
Cyclin_D1	0,129665	0,001313	1,650296	0,127294	0,2261986
TIGAR	0,129574	0,043638	1,09703	0,296209	0,4188231
YB1_pS102	0,12957	-0,081783	1,285143	0,225307	0,3461398
Glutamate-	0,127577	-0,021805	1,417977	0,184073	0,2971628
IRS1	0,116085	0,34378	0,316469	0,757609	0,8075234
AMPK-a2_	0,11558	-0,085886	1,095769	0,296737	0,4188231
PEA15	0,11237	0,139538	0,833019	0,422664	0,5396982
LC3A-B	0,109187	-0,058193	1,065715	0,309528	0,4319408
PEA15_pS1	0,101697	-0,008363	0,556717	0,588938	0,686168
p27_pT198	0,101498	0,106837	1,053753	0,314734	0,4355815
eEF2K	0,100409	0,15178	0,451722	0,660302	0,7379841
FOXO3a_p	0,094894	-0,075844	1,823714	0,095631	0,1803112
JNK_pT183	0,093291	0,025704	0,959186	0,358203	0,4756777
p27-Kip-1	0,090625	-0,052819	1,913175	0,082268	0,158752
Akt	0,089004	-0,093046	0,474568	0,644435	0,7235247
WIPI2	0,076508	-0,008059	0,424551	0,6794	0,7491602
YAP_pS127	0,074437	-0,261542	0,418235	0,683875	0,7507427
DM-K9-His	0,070306	0,013084	1,135106	0,28061	0,4057239
Stathmin	0,063385	-0,09865	0,67448	0,514019	0,6254047
Mnk1	0,061391	0,099689	0,508216	0,621402	0,7124215
p38_MAPK	0,056059	0,026224	0,387848	0,705576	0,7685687
PKM2	0,046362	-0,106769	0,241959	0,813292	0,8548214
mTOR	0,045241	-0,079951	0,426884	0,677751	0,7491602
Wee1_pS6	0,045056	0,207071	0,643198	0,533358	0,6395114
PREX1	0,044776	0,175763	0,55076	0,592877	0,6875148
IGFRb	0,044164	-0,389855	0,427417	0,677375	0,7491602
MDM2_pS	0,03625	-0,166058	0,330196	0,747494	0,8062492
p21	0,029076	-0,282497	0,317732	0,756676	0,8075234
cIAP	0,025597	-0,050772	0,50585	0,623008	0,7124215
Tyro3	0,016713	0,013275	0,306917	0,764675	0,8075234
XRCC1	0,014632	0,052949	0,148387	0,884739	0,9143541
U-Histone-	0,010909	-0,015202	0,117134	0,908879	0,9315062
MCT4	0,004452	-0,74516	0,017185	0,986599	0,992555
Rictor_pT1	0,001608	-0,09316	0,01033	0,991944	0,992555
Rad51	-0,00124	0,207918	-0,00955	0,992555	0,992555
Rb_pS807_	-0,00913	0,206846	-0,03008	0,976542	0,9885487
LRP6_pS14	-0,01096	-0,161801	-0,1418	0,889819	0,915772

PKM2	0,189979	-0,030715	2,921563	0,023427	0,0512087
RIP	0,187817	0,216472	1,363352	0,216825	0,3113704
Gys	0,185007	0,229644	3,15264	0,017055	0,0393704
PAICS	0,18402	0,011217	1,71356	0,132265	0,21214
Bak	0,18062	-0,062856	1,652588	0,144328	0,2270633
Elk1_pS38	0,175146	-0,105295	2,191381	0,066274	0,1186205
STAT5-alph	0,173442	0,775827	3,072549	0,019024	0,0431092
Stat3	0,173035	0,403483	3,030139	0,020164	0,0452767
Mcl-1	0,169579	0,479658	2,567735	0,038559	0,0767051
FOXO3a_p	0,15269	-0,06187	2,656238	0,033999	0,0694039
Stat3_pY70	0,15226	0,240467	2,857044	0,025629	0,0550477
EGFR_pY11	0,150863	-0,072296	2,273822	0,058821	0,1068297
S6_pS240_	0,1319	0,442304	1,231873	0,259488	0,3580641
ATR_pS428	0,130777	-0,033401	1,752501	0,125071	0,2059507
Akt	0,130338	-0,087664	2,386733	0,049979	0,0935216
p53	0,127715	-0,143689	1,597836	0,156039	0,2423994
Aurora-B	0,120157	-0,114976	1,811734	0,114845	0,1916666
CD20	0,11635	0,092588	1,985751	0,089289	0,1553125
Stathmin	0,115549	-0,081181	1,728994	0,129368	0,2102227
mTOR_pS2	0,114578	0,01004	1,580779	0,159865	0,2467916
Hexokinase	0,094923	0,04622	1,717849	0,131454	0,21214
SDHA	0,093427	0,182789	1,170325	0,281823	0,3762714
Lck	0,08686	-0,00688	1,189175	0,274816	0,370927
U-Histone-	0,082333	0,025272	1,155404	0,287475	0,3817538
LC3A-B	0,082153	-0,098236	1,329239	0,227257	0,3226006
MEK1	0,069103	0,359646	0,978753	0,361722	0,4600599
Tyro3	0,065279	0,038768	1,218976	0,264039	0,3603457
p44-42-MA	0,06256	0,020878	1,091774	0,312642	0,4085853
Wee1	0,057608	0,318637	0,960062	0,370391	0,4667683
S6_pS235_	0,051162	0,42135	0,330397	0,751193	0,8246435
NAPSIN-A	0,049716	-0,217621	0,466318	0,655773	0,7430087
MERIT40_	0,048588	0,087179	0,412583	0,692798	0,7813746
TSC1	0,040575	-0,38389	0,717029	0,497592	0,5995378
c-Abl	0,040335	0,046742	0,63463	0,546726	0,6523741
Collagen_V	0,040136	0,187521	0,356881	0,732159	0,8120777
cIAP	0,037947	-0,048946	0,566057	0,589807	0,6904381
TUFM	0,037279	-0,102757	0,355465	0,733171	0,8120777
MEK1_pS2	0,036383	-0,077658	0,55098	0,599535	0,6985145
b-Catenin_	0,02861	-0,070389	0,263338	0,800207	0,8519445
DJ-1	0,028009	-0,061384	0,382096	0,714224	0,8018785
ER-alpha_	0,022935	-0,039132	0,296226	0,776031	0,8336778
Gys_pS641	0,021184	0,030585	0,369732	0,722994	0,8080525
c-Kit	0,020027	0,005564	0,295583	0,776501	0,8336778
PARP	0,017185	-0,064674	0,149695	0,885413	0,904904
YAP	0,013803	-0,006171	0,246721	0,812515	0,8576546
PR	0,010656	0,24656	0,198898	0,848245	0,876638
eIF4G	-2,47E-04	-0,147012	-0,00395	0,99696	0,99696
Bcl-xL	-0,00368	-0,107499	-0,06513	0,949972	0,9577273
MDM2_pS	-0,00591	-0,199264	-0,05304	0,959248	0,9631474
PDHK1	-0,00776	0,058109	-0,12715	0,902555	0,9174122
TIGAR	-0,00856	-0,067512	-0,14815	0,886586	0,904904
DM-K9-His	-0,0101	-0,050499	-0,15294	0,882953	0,904904
Mnk1	-0,01085	0,042951	-0,11676	0,910477	0,9216714
p38_MAPK	-0,01454	-0,028369	-0,20919	0,840521	0,8723052
XPF	-0,01589	0,009206	-0,2963	0,775978	0,8336778
PAK1	-0,01791	-0,004488	-0,30113	0,77245	0,8336778
JNK_pT183	-0,01948	-0,062312	-0,34436	0,74113	0,8172287
p27_pT198	-0,02014	0,01171	-0,25504	0,806344	0,8547941
FoxM1	-0,02167	0,036726	-0,24184	0,816141	0,8578158
4E-BP1_pS	-0,02178	-0,086957	-0,2319	0,823542	0,8619269
LDHA	-0,02473	0,306928	-0,22367	0,829685	0,8646926
TFAM	-0,02557	0,215198	-0,29125	0,779674	0,8336778
Notch3	-0,02558	-0,114612	-0,32481	0,755233	0,8254095
Atg7	-0,02732	-0,058942	-0,499	0,633749	0,734911
G6PD	-0,02819	0,018532	-0,47475	0,650055	0,7430087
Rab25	-0,02846	-0,058297	-0,46709	0,655251	0,7430087
C-Raf_pS33	-0,0308	-0,347453	-0,4849	0,643199	0,7423844
B7-H4	-0,03884	0,068568	-0,70454	0,504849	0,6053288
PI3K-p110-	-0,04162	0,080436	-0,76123	0,472466	0,5748723

PAK1	-0,0136	-0,004866	-0,04448	0,965322	0,9812126
Rab11	-0,01981	-0,035245	-0,1721	0,866507	0,9030688
P-Cadherin	-0,02388	0,057863	-0,30645	0,765022	0,8075234
GCLM	-0,02593	0,020193	-0,50009	0,626924	0,713596
p70-S6K1	-0,02627	0,111673	-0,21259	0,835559	0,8745042
MMP2	-0,02702	0,049248	-0,55704	0,588724	0,686168
YAP	-0,02796	-0,037328	-0,37465	0,715085	0,7746759
JNK2	-0,03743	0,004678	-0,38679	0,706336	0,7685687
Atg7	-0,04046	-0,075657	-0,30686	0,764719	0,8075234
Chk1_pS29	-0,04384	-0,021965	-0,73728	0,476481	0,5914106
Hexokinase	-0,0487	-0,050583	-0,80062	0,440407	0,5521855
Ets-1	-0,04892	0,060662	-0,97684	0,349774	0,4746931
Creb	-0,05367	0,035296	-1,02208	0,328831	0,4487363
Cox2	-0,05874	0,957895	-0,08284	0,935477	0,9548053
PKA-a	-0,05969	-0,157512	-0,4941	0,631013	0,7149549
FAK_pY397	-0,06338	0,253015	-0,15167	0,882213	0,9143541
PMS2	-0,07143	0,147057	-0,59999	0,560745	0,6626989
N-Cadherin	-0,07154	-0,080749	-0,47941	0,641096	0,7230625
Smad1	-0,07287	0,032616	-0,65233	0,527671	0,6357796
CD20	-0,08026	-0,042893	-1,05163	0,315664	0,4355815
NF-kB-p65_	-0,08295	0,032383	-0,56537	0,583242	0,6860037
mTOR_pS2	-0,08552	-0,128678	-0,52021	0,61329	0,7078633
Slfn11	-0,08637	-0,078558	-0,96595	0,354957	0,4751501
GATA6	-0,08797	-0,225422	-0,96402	0,355882	0,4751501
Rab25	-0,08902	-0,114058	-1,14255	0,277638	0,4057239
Jak2	-0,09052	-0,008305	-1,16934	0,267136	0,3974848
IRF-1	-0,09924	0,056109	-1,47526	0,168358	0,2794674
D-a-Tubulin	-0,10018	-0,031204	-1,40656	0,187348	0,3004864
eEF2	-0,10324	0,116836	-0,63082	0,541127	0,6456925
Tuberin_pT	-0,10398	-0,006469	-1,31947	0,213982	0,3324126
ATM_pS19	-0,10456	-0,039159	-2,17966	0,052048	0,1137689
Raptor	-0,10909	0,047014	-1,25124	0,236964	0,3590808
c-Abl	-0,10954	-0,067083	-1,96493	0,075347	0,1465416
ZAP-70	-0,10962	0,048859	-1,13442	0,280886	0,4057239
Cdc2_pY15	-0,11392	0,128145	-1,08775	0,300112	0,4211795
MERIT40_p	-0,11948	-0,039726	-1,17769	0,263926	0,3950895
OCT4	-0,12349	0,032758	-2,62763	0,023618	0,0583365
eIF4E_pS2(	-0,12707	0,046931	-2,2271	0,047917	0,106625
XPF	-0,13373	-0,090681	-2,36272	0,037769	0,0855867
COG3	-0,13669	-0,269422	-0,82924	0,424707	0,5396982
TFAM	-0,13704	0,118415	-0,8328	0,422781	0,5396982
cdc25C	-0,13826	0,142199	-0,92531	0,374784	0,4872195
BRD4	-0,1446	-0,06714	-2,08815	0,060995	0,1289559
PI3K-p110-	-0,14952	-0,016923	-3,41611	0,005814	0,0231627
FoxM1	-0,15471	-0,076849	-1,74971	0,108152	0,1964232
C-Raf	-0,15709	-0,011436	-1,46843	0,170171	0,2802145
PKC-delta_	-0,15902	-0,050856	-3,52424	0,004808	0,01947
14-3-3_bet	-0,17231	-0,020808	-2,84679	0,01598	0,0448519
B7-H4	-0,17286	-0,049555	-2,91179	0,01423	0,0413515
RIP	-0,17423	-0,038769	-0,94825	0,363495	0,4775705
MEK1	-0,1799	0,170994	-1,37431	0,196868	0,3117081
DUSP4	-0,18444	0,128881	-1,47938	0,167273	0,2794674
INPP4b	-0,19089	-0,024663	-3,62402	0,004039	0,0178147
p44-42-MA	-0,19152	-0,17336	-1,28421	0,225621	0,3461398
SHP-2_pY5	-0,19254	-0,09438	-2,0873	0,061084	0,1289559
CD4	-0,20334	0,03085	-2,55706	0,026776	0,063593
CD134	-0,20466	0,02215	-2,95002	0,013292	0,0393039
MYH11	-0,20505	-0,080303	-1,09766	0,295948	0,4188231
DM-Histon	-0,21518	0,048536	-2,76747	0,018408	0,0505211
ER-alpha	-0,21616	0,056836	-3,38394	0,006153	0,0240053
MIF	-0,21648	-0,062072	-2,56651	0,02633	0,0631408
G6PD	-0,21778	-0,142741	-1,51267	0,158728	0,2703856
A-Raf	-0,21779	0,051285	-1,71144	0,115192	0,2061771
Wee1	-0,21884	0,105018	-1,02382	0,328047	0,4487363
PD-L1	-0,21999	-0,036177	-4,263	0,001355	0,0089284
IGFBP2	-0,2265	-0,005399	-1,78229	0,102466	0,1888741
Beclin	-0,23185	0,016681	-3,53411	0,004726	0,01947
FoxO3a	-0,23736	-6,74E-04	-2,86391	0,015499	0,044098
XBP1	-0,24793	0,016132	-4,894	4,85E-04	0,0049924

cdc25C	-0,04292	0,229565	-0,47012	0,65319	0,7430087
ATM_pS19	-0,04503	0,019368	-0,75503	0,475937	0,5762568
N-Cadherin	-0,05146	-0,053096	-0,97553	0,363205	0,4600599
ZAP-70	-0,05551	0,105245	-0,78236	0,460763	0,5634078
MSH6	-0,05754	-0,095416	-0,59024	0,574397	0,6779627
beta-Caten	-0,05824	0,037597	-0,58706	0,576405	0,6779627
Cyclin_D1	-0,0589	-0,140117	-0,90985	0,394464	0,4920839
Glutamate-	-0,05912	-0,161659	-1,01008	0,347548	0,444789
Chk1_pS29	-0,06366	-0,024299	-0,88828	0,405158	0,5018846
JNK2	-0,0644	-0,003338	-1,11781	0,302133	0,3990735
Raptor	-0,07018	0,094162	-1,1847	0,276466	0,371125
CD134	-0,07033	0,1474	-1,07888	0,317957	0,413344
DM-Histon	-0,07749	0,178097	-1,21892	0,264059	0,3603457
Tuberin_pT	-0,08047	0,030326	-1,523	0,173486	0,2628894
GCLM	-0,08184	-0,007694	-1,31115	0,232966	0,3288146
Creb	-0,08273	0,029571	-1,46449	0,188359	0,2819681
Jagged1	-0,08358	-0,255073	-1,56374	0,163774	0,2498952
OCT4	-0,08438	0,083166	-1,61517	0,152237	0,2379901
MSI2	-0,08455	0,189506	-1,42272	0,199681	0,2918411
p27-Kip-1	-0,08616	-0,17866	-1,44399	0,19384	0,2864007
HER3	-0,08715	-0,144546	-1,5632	0,163899	0,2498952
IGFBP2	-0,08762	0,127348	-0,93491	0,382307	0,4793395
Slfn11	-0,09708	-0,066141	-1,05563	0,327722	0,4216005
c-Jun_pS73	-0,09713	-0,261039	-1,35224	0,220177	0,3143565
Rictor	-0,10149	-0,15851	-1,69216	0,136386	0,2173371
BRD4	-0,10243	-0,010294	-1,44056	0,194772	0,2864007
TFRC	-0,10351	0,423692	-0,59613	0,570677	0,6776791
PKC-delta_	-0,10618	0,01554	-1,94196	0,095138	0,1631877
PMS2	-0,1069	0,14136	-1,28062	0,242886	0,3389432
Jak2	-0,10903	3,33E-04	-1,77367	0,12132	0,2011138
p70S6K_pT	-0,10932	0,066268	-0,88584	0,406384	0,5018846
Smad3	-0,11093	-0,04749	-1,06217	0,324952	0,4202255
GCN5L2	-0,11127	-0,141842	-1,10175	0,30858	0,4054211
SHP-2_pY5	-0,11372	-0,005081	-1,81284	0,114661	0,1916666
CD4	-0,11859	0,126065	-1,40431	0,204861	0,2976512
Ets-1	-0,11913	0,029213	-2,19915	0,065532	0,118149
AMPK-a2_p	-0,11918	-0,25196	-1,38655	0,209974	0,3032958
D-a-Tubulin	-0,12001	-0,021247	-1,47906	0,18455	0,2779508
PTEN	-0,1206	0,391667	-2,1152	0,07401	0,1315135
Cdc2_pY15	-0,12447	0,146667	-2,01365	0,08575	0,1502142
53BP1	-0,12579	-0,537412	-0,87951	0,409567	0,503299
MMP2	-0,12896	-0,006025	-2,28913	0,057534	0,1052666
TAZ	-0,13101	-0,016504	-1,67688	0,139403	0,2207208
PAK4	-0,13335	-0,206457	-2,56304	0,038818	0,0767051
Rad51	-0,14224	0,123587	-2,51801	0,041397	0,0798835
Granzyme-	-0,14226	0,287889	-1,94506	0,094712	0,1631877
C-Raf	-0,1492	0,027571	-2,73584	0,03038	0,0635929
VEGFR-2	-0,15015	-0,258649	-2,51138	0,041792	0,0800201
PD-L1	-0,15136	0,053002	-2,54202	0,04	0,0777962
VASP	-0,15443	-0,79474	-1,73271	0,128679	0,2102227
Glutaminas	-0,16944	0,03487	-2,42971	0,046984	0,0885875
14-3-3_bet	-0,17243	0,016716	-2,63356	0,035111	0,0705078
XBP1	-0,17389	0,114646	-2,80976	0,027382	0,0583048
IRF-1	-0,17441	0,032658	-2,67768	0,032982	0,0678885
MYH11	-0,17671	-0,018565	-3,17057	0,016645	0,0387865
INPP4b	-0,18093	0,022965	-3,00863	0,02077	0,046217
FoxO3a	-0,18691	0,081383	-3,20605	0,015864	0,0373192
Axl	-0,18778	0,175097	-3,47248	0,011124	0,0283261
P-Cadherin	-0,19236	-0,038015	-3,31283	0,013744	0,0332818
Wee1_pS6	-0,19312	0,054334	-3,72218	0,008052	0,0228591
eEF2K	-0,19548	-0,047663	-1,28135	0,242646	0,3389432
PEA15	-0,19758	-0,070949	-1,24298	0,255622	0,354712
EGFR	-0,19836	-0,358619	-1,20859	0,267753	0,363379
Atg3	-0,19938	0,20625	-2,87195	0,025102	0,0543879
Claudin-7	-0,20193	-0,300118	-3,44577	0,011522	0,0290396
PEA15_pS1	-0,20226	-0,212926	-1,44046	0,194799	0,2864007
Smad1	-0,20614	-0,031447	-3,39845	0,012265	0,030293
Beclin	-0,20638	0,082546	-3,28545	0,014257	0,0341886
AR	-0,20926	0,202032	-4,06792	0,005227	0,0161384

PDHK1	-0,24969	-0,141532	-4,83918	5,29E-04	0,0052289
PR	-0,25538	0,031221	-4,62549	7,46E-04	0,0063553
Sox2	-0,27523	0,266721	-1,20916	0,252109	0,3797007
TTF1	-0,27763	0,059937	-4,58585	7,96E-04	0,0065523
Collagen_V	-0,28817	-0,072339	-3,15669	0,009201	0,0304905
Gys_pS641	-0,29197	-0,221008	-4,19661	0,001515	0,0093546
TFRC	-0,29611	0,24353	-1,45362	0,174154	0,2848743
MSI2	-0,32677	-0,02712	-5,08369	3,60E-04	0,004157
B-Raf_pS44	-0,33541	-0,001932	-2,50787	0,029219	0,0680857
SDHA	-0,34936	-0,159111	-4,4032	0,001074	0,0079908
Stat3_pY7(	-0,35056	-0,137707	-3,70164	0,003529	0,0164203
Pdcd4	-0,35882	-0,201283	-2,09119	0,060675	0,1289559
HSP27_pS8	-0,36439	0,019929	-2,68632	0,021274	0,0547365
AR	-0,36478	0,029133	-3,98232	0,002177	0,0122401
Fibronectir	-0,36594	-0,081243	-0,67256	0,515194	0,6254047
Glutamina:	-0,38097	-0,175164	-1,99715	0,071316	0,1432118
ATM	-0,3847	0,220743	-2,75482	0,018829	0,0511061
SOD2	-0,39075	-0,00217	-5,75394	1,31E-04	0,0021542
p70S6K_pT	-0,39572	-0,191915	-2,10826	0,058912	0,1276423
B-Raf	-0,39896	-0,012151	-3,33268	0,006736	0,0241119
ULK1_pS75	-0,42069	-0,005227	-4,72419	6,36E-04	0,0060253
LDHA	-0,44425	-0,041709	-2,82167	0,016712	0,0463804
Cyclin_B1	-0,44665	0,056019	-4,26203	0,001358	0,0089284
Gys	-0,46584	-0,262499	-6,48289	4,67E-05	9,61E-04
Axl	-0,48951	-0,112747	-5,70622	1,40E-04	0,0021659
Cox-IV	-0,51711	-0,001632	-4,23952	0,00141	0,0089284
Mcl-1	-0,5315	-0,056949	-2,00174	0,070759	0,1432118
Myosin-IIa_	-0,56759	-0,042934	-3,00941	0,011957	0,0364621
Atg3	-0,5694	-0,139995	-7,22159	1,77E-05	4,90E-04
PLK1	-0,58795	-0,05959	-3,76235	0,003177	0,0153978
PI3K-p85	-0,63576	0,037165	-9,3692	1,48E-06	1,22E-04
TRIM25	-0,65172	-0,111768	-3,25323	0,007752	0,0269689
Granzyme-	-0,67573	-0,179623	-7,87182	7,92E-06	3,26E-04
S6_pS240_	-0,68602	-0,198127	-1,49949	0,162064	0,2741772
PTEN	-0,731	-0,134067	-4,24212	0,001404	0,0089284
PKC-alpha	-0,73215	-0,187465	-3,91269	0,002452	0,0131676
AMPK_pT1	-0,78377	-0,133669	-3,97227	0,002215	0,0122401
S6_pS235_	-0,80664	-0,269325	-1,32602	0,211876	0,3312243
Stat3	-0,89634	-0,433704	-3,59133	0,004276	0,0185294
PAR	-0,90248	1,00175	-1,54287	0,151303	0,2631822
Heregulin	-0,93558	-0,049425	-5,06575	3,70E-04	0,004157
STAT5-alph	-0,95745	-0,111603	-6,36315	5,50E-05	0,0010269
PDGFR-b	-2,77677	-1,172098	-30,5446	6,21E-12	1,53E-09

PREX1	-0,2105	0,01283	-2,29758	0,056836	0,1047657
Rab11	-0,22434	-0,153641	-2,10942	0,074633	0,1316734
MIF	-0,23139	-0,023785	-4,21668	0,004365	0,0141847
eIF4E_pS2(	-0,23782	0,008209	-4,27932	0,004049	0,0133362
ULK1_pS75	-0,24075	0,194529	-3,39095	0,012387	0,030293
RPA32_pS4	-0,24076	-0,363868	-2,55644	0,039186	0,0768163
p70-S6K1	-0,24881	-0,016118	-3,88946	0,006518	0,0196337
SOD2	-0,25126	0,166774	-4,31333	0,003889	0,0131586
RBM15	-0,25367	0,095418	-4,00354	0,005657	0,0172507
ER-alpha	-0,2694	0,072059	-4,38667	0,003566	0,0127663
B-Raf_pS44	-0,27987	0,104569	-3,51451	0,010528	0,0273731
A-Raf	-0,29433	0,052879	-4,72652	0,002412	0,0099295
HSP70	-0,29789	-0,329619	-2,71675	0,03121	0,0647802
Cyclin_B1	-0,30724	0,237113	-4,47017	0,003235	0,0122912
ATM	-0,31496	0,346519	-2,63756	0,034912	0,0705078
TTF1	-0,31568	0,097674	-5,23531	0,001385	0,0065509
b-Actin	-0,33762	-0,255878	-4,66094	0,002598	0,0104183
Cox-IV	-0,37275	0,197812	-4,62509	0,002706	0,0106087
eEF2	-0,37502	-0,023708	-3,48264	0,010977	0,028242
B-Raf	-0,39284	0,078568	-7,40208	1,85E-04	0,0013065
PLK1	-0,40314	0,179577	-5,07954	0,001635	0,0073431
PI3K-p85	-0,41028	0,311163	-5,98445	6,51E-04	0,0034941
c-Myc	-0,41846	-0,261359	-4,65506	0,002615	0,0104183
ARID1A	-0,42873	-0,318287	-4,87571	0,002042	0,0085497
HSP27_pS8	-0,45719	0,043754	-4,29815	0,00396	0,0132169
DUSP4	-0,46174	0,002738	-8,67448	7,00E-05	6,40E-04
Rad50	-0,49539	-0,364531	-4,59453	0,002802	0,0108141
Merlin	-0,4973	-0,364996	-4,13384	0,004824	0,0152748
Myosin-IIa_	-0,51024	0,115317	-3,59979	0,009423	0,0255765
TRIM25	-0,51024	0,115317	-3,59979	0,009423	0,0255765
NF-kB-p65_	-0,51796	-0,210527	-8,6935	6,90E-05	6,40E-04
FAK_pY397	-0,54151	-0,02003	-7,45072	1,78E-04	0,0013065
Connexin-4	-0,54986	-0,005134	-6,74757	3,22E-04	0,0019426
IRS1	-0,56784	-0,091901	-9,31209	4,49E-05	4,52E-04
Sox2	-0,57945	0,144242	-7,32754	1,97E-04	0,0013138
Gab2	-0,60218	-0,972387	-9,2849	4,58E-05	4,52E-04
PKC-alpha	-0,71892	-0,019783	-12,4502	7,07E-06	1,03E-04
p16_INK4a	-0,93593	-1,065797	-14,4963	2,63E-06	4,65E-05
Heregulin	-1,02453	0,101335	-18,0143	6,35E-07	1,57E-05
PDGFR-b	-1,08412	0,449338	-19,4359	3,85E-07	1,06E-05
E-Cadherin	-1,36551	-1,085202	-4,44167	0,003344	0,0124122
Cox2	-2,24692	-0,342198	-34,5506	8,46E-09	9,01E-07
PAR	-2,5017	0,239122	-25,1183	7,06E-08	2,90E-06

Subtype A vs. MGT7

Protein	logFC	AveExpr	t	P.Value	adj.P.Val
E-Cadherin	4,077979	1,851072	13,59682	3,49E-08	1,73E-06
P-Met_pY1	3,905679	-0,561518	8,062376	6,39E-06	8,49E-05
RSK	2,093323	-0,522712	17,44287	2,58E-09	2,12E-07
CDK1	2,055225	-0,044656	6,337862	5,75E-05	3,23E-04
ARID1A	1,990081	0,479544	5,962624	9,74E-05	4,63E-04
Claudin-7	1,973089	1,311605	7,625586	1,08E-05	1,03E-04
Rb_pS807_	1,75058	-0,113102	5,758942	1,31E-04	5,87E-04
Cox2	1,715259	0,635351	2,416961	0,034359	0,0606194
Bim	1,640005	0,183023	4,214912	0,001475	0,0044977
ACC_pS79	1,626265	-0,122915	4,328442	0,001221	0,0039119
Connexin-4	1,604578	0,22003	6,424789	5,10E-05	3,02E-04
Rad50	1,484122	0,301541	7,84141	8,30E-06	8,92E-05
FASN	1,27253	-0,140903	10,44204	5,14E-07	1,50E-05
ACC1	1,219935	0,109605	6,171473	7,24E-05	0,000389
PAR	1,188793	0,62152	2,029457	0,067524	0,1030536
53BP1	1,147778	0,719624	3,536219	0,004722	0,01178
GCN5L2	1,090841	0,203806	9,041021	2,13E-06	4,04E-05
4E-BP1_pS	1,053014	0,270633	8,000785	6,87E-06	8,49E-05
Src_pY416	0,971179	-0,178759	4,825654	5,44E-04	0,0019745
IRS1	0,9708	0,188377	2,63659	0,023276	0,0438869
p90RSK_pT	0,943846	-0,085843	8,897744	2,49E-06	4,39E-05
ATM	0,881887	-0,009546	6,501237	4,59E-05	2,91E-04
PARP	0,817498	0,237528	7,295409	1,62E-05	1,29E-04
Akt_pS473	0,808969	-0,222421	3,568563	0,004462	0,0114804
Notch1	0,793386	-0,282907	3,537211	0,004713	0,01178
c-Jun_pS73	0,789696	0,239301	6,09568	8,06E-05	4,06E-04
beta-Caten	0,750219	0,225981	4,315244	0,001248	0,0039119
c-Myc	0,71508	0,21677	7,349606	1,52E-05	1,25E-04
C-Raf_pS33	0,619967	0,059052	4,13766	0,001679	0,0050585
PAK1	0,590111	-0,114631	1,930564	0,079928	0,1189295
RPA32_pS4	0,583537	-0,063773	6,43274	5,04E-05	3,02E-04
PI3K-p85	0,578939	-0,183689	8,546006	3,67E-06	5,90E-05
HES1	0,575656	0,231788	1,256218	0,23527	0,3026657
eIF4E	0,550199	-0,025588	6,108774	7,91E-05	4,06E-04
Cdc2_pY15	0,5306	0,01096	4,900449	4,83E-04	0,0018066
Bid	0,520625	0,129638	3,549615	0,004612	0,0117445
RBM15	0,504069	0,490142	0,941461	0,366848	0,4441738
Src_pY527	0,495044	0,037893	2,216448	0,048859	0,0807804
HER3	0,484719	0,504228	2,581701	0,025662	0,0473706
eEF2	0,478251	0,01111	2,996859	0,01225	0,0254274
AMPK_alpl	0,444753	-0,170229	4,313517	0,001251	0,0039119
Smad3	0,437212	0,147645	5,818215	1,20E-04	5,59E-04
Akt_pT308	0,433648	-0,06235	1,968314	0,074962	0,1129002
PKC-b-II_p	0,427428	-0,185356	3,442722	0,005562	0,0134699
Caspase-3	0,427404	0,154166	5,252264	2,79E-04	0,0011333
Bcl-xL	0,420292	0,182625	3,147138	0,009378	0,020498
c-Kit	0,411685	0,198267	5,25049	2,80E-04	0,0011333
Wee1_pS6	0,40153	0,142258	4,645249	7,26E-04	0,0023914
Paxillin	0,385944	-0,017222	6,852062	2,87E-05	2,07E-04
Histone-H3	0,366995	0,057748	2,099147	0,059892	0,0954415
Shc_pY317	0,365979	-0,208821	3,786027	0,003059	0,0084894
HSP70	0,360941	0,055002	4,667178	7,01E-04	0,0023714
14-3-3_zet	0,354151	-0,00447	3,090472	0,010371	0,0222748
FAK	0,34764	-0,018272	3,843346	0,002771	0,0077789
PRAS40_pT	0,342914	-0,107622	1,167702	0,267813	0,3392298
VASP	0,3403	-0,031485	1,781609	0,102633	0,1491199
Lck	0,333933	0,086966	2,531525	0,028053	0,0504822
XRCC1	0,333274	-0,004986	3,395825	0,006041	0,0142101
Sox2	0,332648	0,156197	1,460706	0,172288	0,2325423
DM-K9-His	0,311906	-0,030843	4,64969	7,21E-04	0,0023914
Caspase-7_	0,31119	-0,113361	2,543016	0,027486	0,0499203
Chk2_pT68	0,310569	-0,020476	3,082131	0,010526	0,0224127
Myt1	0,30976	-0,038003	3,199444	0,008547	0,019191
Merlin	0,309137	0,081024	1,768057	0,104967	0,1516185
DJ-1	0,298587	0,133255	3,448291	0,005508	0,0134699
WIPI1	0,293815	0,245293	1,298115	0,221019	0,288845

subtype B vs. MGT 7

Protein	logFC	AveExpr	t	P.Value	adj.P.Val
P-Met_pY1	4,697158	-0,938778	74,92326	1,46E-10	3,62E-08
RSK	2,683463	-0,625353	54,86156	1,05E-09	1,29E-07
Notch1	2,347481	0,476448	45,8311	3,26E-09	2,03E-07
Rb_pS807_	2,18387	-0,235072	39,93065	7,76E-09	3,83E-07
ACC_pS79	2,067445	-0,213028	23,94125	1,93E-07	4,51E-06
CDK1	1,97376	-0,541948	38,45157	9,84E-09	4,05E-07
Fibronectir	1,530955	1,205599	9,730992	4,91E-05	2,53E-04
Akt_pS473	1,368307	-0,06332	13,46006	6,88E-06	6,30E-05
Bim	1,28209	-0,389545	24,19337	1,81E-07	4,51E-06
FASN	1,22342	-0,448012	14,95374	3,60E-06	3,58E-05
HES1	1,220882	0,493327	21,1277	4,21E-07	6,94E-06
ACC1	1,207576	-0,163979	21,16275	4,17E-07	6,94E-06
Src_pY416	1,029589	-0,355606	18,66111	9,13E-07	1,11E-05
PKA-a	0,985279	0,443107	19,2266	7,59E-07	1,04E-05
ATM	0,951623	-0,160116	8,725685	9,39E-05	4,37E-04
p90RSK_pT	0,890456	-0,323807	10,28256	3,53E-05	2,03E-04
Connexin-4	0,867729	-0,57217	10,61205	2,92E-05	1,76E-04
Pdcd4	0,811065	0,126594	11,36853	1,93E-05	1,22E-04
PI3K-p85	0,804418	-0,174715	12,15262	1,29E-05	9,34E-05
Akt_pT308	0,800977	0,063433	9,42914	5,93E-05	2,93E-04
STAT5-alph	0,729843	0,553266	12,95944	8,68E-06	6,70E-05
IGFRb	0,673653	0,876255	13,66633	6,27E-06	5,96E-05
ARID1A	0,658924	-0,75335	7,53846	2,20E-04	8,26E-04
LRP6_pS14	0,641061	0,293243	11,98267	1,40E-05	9,88E-05
Shc_pY317	0,607148	-0,143969	9,197737	6,87E-05	3,33E-04
PAK1	0,585795	-0,245972	11,11462	2,21E-05	1,36E-04
AMPK_alpl	0,582495	-0,184621	10,14781	3,82E-05	2,15E-04
XRCC1	0,525977	0,037921	6,388376	5,63E-04	0,0018298
Cdc2_pY15	0,520045	-0,11114	7,186166	2,90E-04	0,0010225
Wee1	0,506484	0,139087	8,132213	1,42E-04	6,15E-04
GCN5L2	0,502351	-0,38729	5,969369	8,19E-04	0,0023802
Mcl-1	0,499886	0,347536	7,515356	2,24E-04	8,26E-04
4E-BP1_pS	0,495256	-0,29377	8,153183	1,40E-04	6,15E-04
AMPK_pT1	0,486715	0,997887	4,799688	0,002606	0,0060716
Bid	0,484994	-0,00533	3,802381	0,00811	0,0147343
PRAS40_pT	0,479974	-0,100203	8,915874	8,26E-05	3,93E-04
RIP	0,462285	0,106685	1,844754	0,112134	0,1465453
Caspase-7_	0,449403	-0,098329	8,343125	1,22E-04	5,59E-04
PTEN	0,448663	0,163963	9,436692	5,90E-05	2,93E-04
PKC-b-II_p	0,433918	-0,274718	6,139734	7,02E-04	0,0021397
Tuberin	0,409148	0,22117	4,123747	0,005535	0,0110677
Bad_pS112	0,405622	-0,121011	5,186137	0,001744	0,0045816
beta-Caten	0,402134	-0,146554	6,042147	7,66E-04	0,0023086
Paxillin	0,39243	-0,097537	7,902622	1,68E-04	6,86E-04
mTOR	0,387562	-0,066634	7,952201	1,62E-04	6,77E-04
PARP	0,373534	-0,207213	3,540071	0,011208	0,0197741
Chk2_pT68	0,368514	-0,05347	5,439334	0,001355	0,0035975
eIF4E	0,350416	-0,265501	6,760994	4,10E-04	0,0013834
Rad50	0,350054	-0,702708	3,519606	0,0115	0,0201447
Myt1	0,325818	-0,095952	5,030162	0,002046	0,0050525
HER2_pY12	0,319297	0,094711	4,232412	0,004881	0,0101314
WIPI1	0,294558	0,181634	3,502838	0,011745	0,0204291
IRS1	0,286879	-0,433787	3,969903	0,006632	0,0130014
STING	0,27701	0,433335	2,789512	0,029918	0,0461539
14-3-3_zet	0,271302	-0,131449	5,086183	0,001931	0,0049116
Stathmin	0,267642	-0,142018	4,610754	0,003195	0,0072399
COG3	0,26554	0,083359	3,802082	0,008113	0,0147343
Bak	0,250272	-0,090717	3,83869	0,007761	0,0143458
YB1_pS102	0,248613	-0,039595	3,901744	0,007194	0,0135278
Raptor	0,247552	-0,032933	4,238046	0,00485	0,0101314
SDHA	0,245534	0,121946	3,433324	0,012824	0,0216948
Caspase-3	0,238695	-0,052312	3,961999	0,006695	0,0130208
FAK	0,23667	-0,160703	2,734696	0,032237	0,0485526
DM-K9-His	0,231501	-0,147139	3,087097	0,020079	0,0324157
C-Raf	0,225082	-0,122143	4,418151	0,003954	0,0085667
IGFBP2	0,218739	0,004805	1,89843	0,103935	0,1372836

PAICS	0,290497	0,348264	1,187647	0,260186	0,3312678
Bak	0,263146	-0,025579	3,326898	0,006821	0,0157461
DUSP4	0,259141	0,04823	2,047837	0,065427	0,1010029
Bad_pS112	0,25529	-0,15551	3,110719	0,010004	0,021676
HER2_pY12	0,252006	0,109319	1,324687	0,212352	0,2813903
p38_pT180	0,244549	0,152977	3,217093	0,008283	0,0187706
p27_pT198	0,242723	0,08116	2,729391	0,019731	0,0386796
Rictor	0,231252	0,003434	2,248235	0,04622	0,077663
Wee1	0,230033	0,023405	1,07217	0,306787	0,3827096
mTOR	0,227446	-0,113079	2,147609	0,055074	0,0883326
EGFR_pY11	0,220239	-0,020761	4,315428	0,001247	0,0039119
C-Raf	0,217194	-0,079488	2,02889	0,06759	0,1030536
Stathmin	0,215478	-0,126303	2,337273	0,039539	0,0682952
Elk1_pS383	0,212288	-0,025692	3,610465	0,004147	0,0107835
ATR_pS428	0,211757	0,022845	3,414579	0,005845	0,0138809
TUFM	0,210513	0,137065	2,61265	0,024288	0,0454489
Raptor	0,208643	-0,010756	2,375235	0,036984	0,0647868
ER-alpha_p	0,208631	0,195351	2,953036	0,013245	0,0270372
Bax	0,196227	0,010879	0,440265	0,66834	0,7219861
MEK1_pS2	0,195554	0,025863	3,328584	0,006801	0,0157461
Chk1_pS29	0,18635	-0,063817	2,978386	0,01266	0,0260589
A-Raf	0,185335	-0,02201	1,464524	0,171263	0,2324288
b-Actin	0,169691	0,160934	1,054955	0,314248	0,3900466
Tyro3	0,168276	-0,014282	3,054048	0,011065	0,0233592
TAZ	0,155878	0,184933	1,815747	0,096962	0,1425579
GCLM	0,150735	-0,011929	3,187728	0,008726	0,0194173
EGFR	0,147047	-0,047453	1,591412	0,140054	0,1943441
NAPSIN-A	0,146958	-0,080924	1,935652	0,079242	0,1186224
UBAC1	0,143835	-0,04358	1,511115	0,159169	0,2172084
NF-kB-p65_	0,138268	-0,007839	0,931245	0,371876	0,4480651
eIF4G	0,133138	0,068523	1,787907	0,101565	0,1484409
PDK1	0,131864	-0,009512	2,086559	0,061208	0,0959359
YB1_pS102	0,130538	-0,081959	1,322557	0,213036	0,2813903
MSH6	0,117776	0,164421	0,748335	0,470083	0,5425722
AMPK-a2_p	0,116284	-0,086014	1,096294	0,296559	0,3737254
Notch3	0,116038	0,05209	2,258598	0,04539	0,0767905
eIF4E_pS20	0,113283	0,00323	1,683749	0,12059	0,1708614
RIP	0,100239	-0,088672	0,438797	0,669372	0,7219861
p16_INK4a	0,100033	0,299067	0,373217	0,716137	0,7657393
PI3K-p110-	0,09905	-0,062117	2,304663	0,04187	0,0718194
Pdcd4	0,093523	-0,283527	0,544331	0,597163	0,658033
p27-Kip-1	0,092997	-0,05325	1,861886	0,089752	0,1327472
Tuberin	0,092488	0,051353	0,816781	0,431528	0,5075587
XPF	0,08906	-0,131189	1,587525	0,14093	0,1944682
Aurora-B	0,087973	-0,024936	0,885671	0,394897	0,4689401
Rab11	0,085186	-0,054335	0,738243	0,475947	0,5467861
FOXO3a_p!	0,083044	-0,073689	1,614393	0,134967	0,1883432
YAP	0,081174	-0,057171	1,089494	0,299416	0,3754096
IGFBP2	0,079857	-0,061101	0,593631	0,564863	0,6341875
PEA15_pS1	0,070285	-0,002652	0,382432	0,709486	0,7619261
Ets-1	0,06179	0,040532	1,220786	0,247895	0,3172537
p53	0,06094	0,003464	0,455481	0,657695	0,7156414
MDM2_pS	0,05173	-0,168872	0,477976	0,642101	0,7017648
4E-BP1	0,050315	0,164505	0,250601	0,80678	0,8408209
Cyclin_D1	0,045221	0,016667	0,563676	0,584375	0,6472668
TIGAR	0,039477	0,060019	0,334432	0,744394	0,7824057
D-a-Tubulir	0,037758	-0,056284	0,627801	0,543051	0,6152922
AR	0,018574	-0,040568	0,197774	0,846858	0,8727302
JNK2	0,011897	-0,004291	0,121746	0,905314	0,9240183
cIAP	0,002476	-0,046568	0,050399	0,960715	0,96587
b-Catenin_	2,89E-05	0,070485	3,62E-04	0,999718	0,9997176
Akt	-0,00926	-0,07518	-0,0488	0,96196	0,96587
mTOR_pS2	-0,01366	-0,141743	-0,08319	0,935205	0,9467033
PKA-a	-0,0203	-0,164673	-0,16907	0,868835	0,8904658
PLC-gamma	-0,02153	0,030147	-0,33716	0,742393	0,7824057
Slfn11	-0,02255	-0,09016	-0,25396	0,804249	0,8408209
c-Abl	-0,03094	-0,081375	-0,58467	0,570663	0,6377998
U-Histone-	-0,03409	-0,00702	-0,3345	0,744342	0,7824057
MSI2	-0,03581	-0,080021	-0,57022	0,580082	0,645407

Tyro3	0,216841	-0,021857	4,213207	0,00499	0,0102712
Lck	0,214116	-0,057782	2,749367	0,031598	0,0478822
PI3K-p110-	0,206945	-0,018991	4,176665	0,005205	0,0105379
XPF	0,206908	-0,079911	4,263259	0,004712	0,0100325
eEF2	0,20647	-0,256304	2,235929	0,064486	0,0899894
MSI2	0,206411	0,073123	3,944605	0,006835	0,0130871
IGF1R_pY1	0,203077	0,268704	3,15669	0,018323	0,0301385
p21	0,196038	-0,030453	3,725141	0,008911	0,0159489
UBAC1	0,191785	-0,046192	3,92717	0,006979	0,0132594
EGFR_pY11	0,189005	-0,087553	3,705302	0,00913	0,0162243
mTOR_pS2	0,186438	-0,018703	2,700916	0,033761	0,0502354
Histone-H3	0,184841	-0,131616	1,711879	0,135249	0,1695762
AR	0,174091	0,04869	3,060825	0,020789	0,0333433
ATR_pS428	0,169321	-0,048818	2,39691	0,051438	0,0734402
Chk1_pS29	0,166521	-0,116373	2,190569	0,068747	0,0953957
Elk1_pS383	0,164145	-0,100894	3,258162	0,016054	0,0267929
Wee1_pS6	0,163351	-0,088256	1,790287	0,121102	0,1557927
SLC1A5	0,161474	0,51657	2,657443	0,035836	0,0526878
MAPK_pT2	0,155805	0,083148	1,900907	0,103572	0,1372836
c-Kit	0,15214	-0,047281	1,584196	0,161743	0,1997521
GATA6	0,150994	0,039006	3,044622	0,02124	0,0338469
FOXO3a_p!	0,140839	-0,05713	2,660797	0,035671	0,0526878
S6_pS240_	0,12355	0,445644	1,144789	0,293792	0,3406887
YAP	0,122936	-0,049824	2,363145	0,053928	0,0765528
Bax	0,122129	-0,076393	2,416638	0,050038	0,0718576
p27_pT198	0,121086	-0,04478	2,287947	0,059933	0,0841099
c-Abl	0,118939	0,015301	2,172045	0,070568	0,0973764
Smad3	0,11821	-0,139148	0,958144	0,373195	0,4267554
A-Raf	0,108797	-0,10837	1,961889	0,095005	0,1275342
c-Jun_pS73	0,106923	-0,34266	1,761632	0,126097	0,1605466
GCLM	0,094828	-0,078361	1,893753	0,104626	0,1374601
PLC-gamma	0,093728	0,104	1,902321	0,103365	0,1372836
C-Raf_pS33	0,08842	-0,395141	1,749967	0,128188	0,1623709
RPA32_pS4	0,08104	-0,492589	0,878326	0,411911	0,4667061
MEK1_pS2	0,071031	-0,091517	1,206356	0,27089	0,3171079
DJ-1	0,060863	-0,074525	0,935965	0,383663	0,4367036
Bcl-xL	0,052279	-0,129883	1,046435	0,333722	0,3833918
U-Histone-	0,037331	0,043273	0,397148	0,704325	0,7531093
Akt	0,032079	-0,04836	0,486619	0,642945	0,6965234
IR-b	0,031783	0,316294	0,225578	0,828649	0,8457697
Aurora-B	0,030001	-0,078913	0,331881	0,750711	0,7919369
Sox2	0,028433	-0,098909	0,367761	0,725056	0,7686211
Src_pY527	0,022202	-0,353822	0,418916	0,689144	0,7400808
D-a-Tubulir	0,017926	-0,076422	0,328062	0,753462	0,7919369
cIAP	0,014827	-0,039698	0,238545	0,819006	0,8393962
MDM2_pS	0,009568	-0,205456	0,088181	0,932461	0,9400729
PKC-delta_	0,009284	-0,030647	0,158939	0,878675	0,8894786
NAPSIN-A	0,003427	-0,199106	0,033002	0,974691	0,9746906
eIF4E_pS20	0,002538	-0,087934	0,0336	0,974232	0,9746906
Ets-1	-0,00842	-0,015073	-0,16254	0,875957	0,8894786
Rictor	-0,01333	-0,193772	-0,24154	0,816783	0,8393962
JNK2	-0,01507	-0,023068	-0,25977	0,803298	0,8336747
DUSP4	-0,01817	-0,174693	-0,30705	0,768672	0,8044994
p38_pT180	-0,01984	-0,059012	-0,276	0,791352	0,824742
PAICS	-0,02576	0,095129	-0,24407	0,814904	0,8393962
Slfn11	-0,03327	-0,091667	-0,36891	0,72424	0,7686211
14-3-3_bet	-0,03672	-0,03757	-0,60925	0,563605	0,6159749
p53	-0,04621	-0,07412	-0,70626	0,505217	0,5595896
PDK1	-0,05047	-0,14768	-0,74929	0,480636	0,5371812
TUFM	-0,05267	-0,066776	-0,69002	0,514708	0,5675577
ER-alpha_p	-0,05461	-0,008113	-0,71014	0,502968	0,5595896
c-Myc	-0,06045	-0,404563	-0,68072	0,520194	0,5710574
MERIT40_p	-0,07311	0,13586	-0,5356	0,610552	0,6643448
FoxM1	-0,07797	0,059247	-0,85536	0,42359	0,4777478
Claudin-7	-0,07815	-0,34963	-1,25117	0,255196	0,3001594
Notch3	-0,08199	-0,092046	-1,52618	0,175339	0,2133429
CD20	-0,08368	0,172599	-1,58464	0,161643	0,1997521
RBM15	-0,08377	0,02746	-1,53398	0,173451	0,2120923
p27-Kip-1	-0,08378	-0,179609	-1,35737	0,221134	0,2625964

14-3-3_bet	-0,0366	-0,045484	-0,61735	0,549669	0,6199463
Smad1	-0,0399	0,026621	-0,35583	0,728754	0,775872
Jagged1	-0,04015	0,160316	-0,19628	0,847997	0,8727302
PKC-delta_	-0,04355	-0,07185	-0,89381	0,390713	0,4684759
Heregulin	-0,04479	-0,211387	-0,24282	0,812649	0,8433794
MMP2	-0,0474	0,052953	-0,98348	0,346679	0,426019
IRF-1	-0,05491	0,04805	-0,81101	0,434694	0,5088597
STING	-0,06749	0,211912	-0,10141	0,921061	0,9362228
LRP6_pS14	-0,0724	-0,15063	-0,94618	0,36454	0,4441738
MAPK_pT2	-0,1042	-0,095589	-0,54094	0,599423	0,658033
Rad51	-0,12134	0,229754	-0,88946	0,392944	0,4688757
PDK1_pS24	-0,12291	0,169696	-1,70236	0,116972	0,1679774
p70-S6K1	-0,12351	0,129352	-1,00516	0,336594	0,4156942
p21	-0,12913	-0,253731	-1,41581	0,18474	0,246653
Creb	-0,13563	0,050198	-2,6463	0,022878	0,0434674
IGF1R_pY1	-0,14277	0,030049	-2,1569	0,054193	0,0874881
IR-b	-0,15029	0,174263	-0,7185	0,487553	0,5575264
eEF2K	-0,15551	0,19831	-0,71006	0,492563	0,5606592
PTEN	-0,16174	-0,237569	-0,94207	0,366548	0,4441738
TTF1	-0,16512	0,039481	-2,9145	0,014186	0,0284879
OCT4	-0,17705	0,042496	-3,68896	0,003618	0,00961
FoxO3a	-0,1888	-0,009503	-2,29361	0,04269	0,0727205
PEA15	-0,1898	0,194478	-1,43683	0,178817	0,2400428
GATA6	-0,18983	-0,206902	-2,21413	0,049057	0,0807804
Jak2	-0,19383	0,01048	-2,52848	0,028205	0,0504822
ER-alpha	-0,19453	0,052902	-3,15482	0,009251	0,0204008
XBP1	-0,19696	0,006864	-4,28252	0,001317	0,0040676
SDHA	-0,19726	-0,186767	-2,58089	0,025699	0,0473706
PREX1	-0,19958	0,220191	-2,42653	0,033783	0,0600322
Mcl-1	-0,20119	-0,117005	-0,75625	0,465519	0,5398267
JNK_pT183	-0,20163	0,079326	-2,08504	0,061368	0,0959359
FoxM1	-0,21101	-0,066612	-2,36236	0,037832	0,065806
B-Raf_pS44	-0,21397	-0,024011	-1,6566	0,126047	0,176895
COG3	-0,21831	-0,254581	-1,31567	0,215261	0,282817
INPP4b	-0,2184	-0,019661	-3,74148	0,003304	0,0090666
CD134	-0,23267	0,027245	-3,45868	0,005409	0,0133595
MERIT40_p	-0,24118	-0,017598	-2,18225	0,051857	0,0842679
p38_MAPK	-0,24121	0,080272	-1,68139	0,121056	0,1708614
N-Cadherin	-0,25227	-0,047889	-1,68977	0,119409	0,1704862
HER3_pY12	-0,2527	0,035728	-3,6702	0,003738	0,0098224
Glutamate-	-0,26454	0,049489	-2,943	0,013484	0,0272993
CD20	-0,28029	-0,006524	-3,7106	0,003485	0,0094595
SLC1A5	-0,28694	0,184915	-1,26037	0,233826	0,3023821
PD-L1	-0,29215	-0,023057	-5,77988	1,27E-04	5,80E-04
ZAP-70	-0,29337	0,082267	-3,03637	0,011418	0,0239012
Beclin	-0,30749	0,030434	-4,99438	4,16E-04	0,0015821
P-Cadherin	-0,30802	0,109523	-3,43219	0,005666	0,0135884
Rab25	-0,31623	-0,072747	-4,07211	0,001876	0,005516
Mnk1	-0,31864	0,168786	-2,7471	0,019118	0,037778
cdc25C	-0,32948	0,176967	-2,05459	0,064672	0,1004658
ATM_pS19	-0,33532	0,002799	-7,52054	1,23E-05	1,12E-04
MIF	-0,34484	-0,038735	-4,13008	0,001701	0,0050618
G6PD	-0,35346	-0,118073	-2,2265	0,048009	0,080123
PR	-0,35651	0,049608	-6,40147	5,26E-05	3,02E-04
FAK_pY397	-0,35757	0,306504	-0,85553	0,410652	0,4853166
DM-Histon	-0,36085	0,075023	-4,6957	6,69E-04	0,0023286
Gab2	-0,36871	0,167786	-1,26068	0,233719	0,3023821
Tuberin_pT	-0,37218	0,042294	-4,68225	6,84E-04	0,0023466
STAT5-alph	-0,40104	-0,212767	-2,65449	0,022546	0,0431703
CD4	-0,41325	0,069014	-5,17645	3,14E-04	0,0012104
ERCC5	-0,419	-0,018824	-3,96449	0,002252	0,0065448
GSK-3a-b_p	-0,42261	0,040381	-1,99485	0,071644	0,1085653
VEGFR-2	-0,42756	0,070836	-2,55996	0,026672	0,0488002
Fibronectin	-0,4348	-0,068722	-0,80195	0,439698	0,5122902
Rictor_pT1	-0,44919	-0,011196	-2,89756	0,014621	0,0291246
SOD2	-0,44982	0,008572	-6,68145	3,60E-05	2,41E-04
BRD4	-0,45613	-0,010498	-6,96157	2,49E-05	1,86E-04
PAK4	-0,46359	0,132529	-4,87975	4,99E-04	0,0018389
Hexokinase	-0,46452	0,025019	-7,4514	1,34E-05	1,18E-04

PR	-0,09048	0,287013	-1,77088	0,124464	0,1592882
Atg3	-0,09665	0,16516	-0,84611	0,428357	0,4809286
CD134	-0,09835	0,158608	-1,7218	0,133374	0,1680785
TIGAR	-0,09866	-0,031474	-1,83394	0,113861	0,1472443
Stat3	-0,10554	0,514912	-2,07753	0,080657	0,1100685
eIF4G	-0,1067	-0,10443	-1,39794	0,209231	0,2508744
AMPK-a2_p	-0,11848	-0,252242	-1,3651	0,21882	0,2611044
Rab11	-0,11934	-0,195639	-1,11099	0,307043	0,3543911
XBP1	-0,12292	0,094257	-2,48741	0,045337	0,0654866
TAZ	-0,12631	-0,018387	-1,53397	0,173452	0,2120923
ERCC5	-0,12795	0,247226	-1,15828	0,28864	0,3362926
b-Catenin_	-0,12941	-0,007183	-1,28495	0,243892	0,2882362
IRF-1	-0,13009	0,014927	-2,03833	0,085258	0,1157078
Heregulin	-0,13374	-0,254981	-2,65088	0,036161	0,0528507
OCT4	-0,13794	0,10459	-2,72506	0,032665	0,0488979
FoxO3a	-0,13835	0,061959	-2,61028	0,03824	0,0555612
Cyclin_D1	-0,14334	-0,106339	-2,13945	0,073894	0,1013985
E-Cadherin	-0,14595	-1,573026	-0,44404	0,671813	0,7246191
MMP2	-0,14934	0,002127	-2,8531	0,027448	0,0426387
B-Raf_pS44	-0,15844	0,055994	-2,77263	0,030613	0,0466748
G6PD	-0,16386	0,072803	-1,40337	0,207682	0,2502312
Creb	-0,16469	0,062356	-3,28144	0,015578	0,0261749
Smad1	-0,17317	-0,044635	-2,91578	0,025225	0,0396852
MSH6	-0,18649	-0,043836	-2,31558	0,057649	0,0813681
TTF1	-0,20318	0,052673	-4,3103	0,004466	0,0095913
INPP4b	-0,20844	0,033968	-3,0191	0,021972	0,0347891
HSP70	-0,20846	-0,365392	-1,98403	0,092073	0,1242738
Jak2	-0,21235	0,04166	-3,77507	0,008384	0,015115
DM-Histon	-0,22316	0,236368	-3,83638	0,007783	0,0143458
PD-L1	-0,22352	0,081866	-4,11713	0,005577	0,0110677
GSK-3a-b_p	-0,22529	0,250974	-4,24644	0,004803	0,0101314
p44-42-MA	-0,22796	0,137085	-4,1135	0,005601	0,0110677
N-Cadherin	-0,23219	0,019196	-4,78009	0,002661	0,0061419
PEA15_pS1	-0,23367	-0,200361	-1,60621	0,156849	0,1956648
cdc25C	-0,23414	0,306053	-1,84101	0,112729	0,1465474
Stat3_pY70	-0,23629	0,395886	-5,00692	0,002095	0,0051244
ZAP-70	-0,23925	0,178742	-3,47374	0,012184	0,0208984
Rictor_pT1	-0,24618	0,208616	-5,06932	0,001965	0,0049116
ER-alpha	-0,24776	0,063404	-4,6993	0,002902	0,0066368
Rab25	-0,25567	0,032587	-4,47119	0,003726	0,0081454
Rad51	-0,26234	0,171627	-3,13177	0,018932	0,0307647
HER3_pY12	-0,26425	0,083933	-4,52409	0,003514	0,0078198
EGFR	-0,2702	-0,329884	-1,42435	0,201796	0,2443318
ATM_pS19	-0,2758	0,111675	-5,53879	0,001229	0,0033368
Beclin	-0,28202	0,112803	-5,74327	0,001011	0,0028377
HER3	-0,28705	-0,064588	-4,18439	0,005159	0,0105306
PDHK1	-0,2881	0,170248	-4,58055	0,003302	0,0074152
NF-kB-p65_	-0,29675	-0,299014	-4,50104	0,003605	0,0079502
SOD2	-0,31034	0,190406	-5,81696	9,43E-04	0,0027094
p38_MAPK	-0,31181	0,090537	-5,08912	0,001925	0,0049116
JNK_pT183	-0,3144	0,055657	-6,22359	6,51E-04	0,0020354
Hexokinase	-0,32089	0,212545	-5,7514	0,001003	0,0028377
CD4	-0,3285	0,210027	-3,89766	0,007229	0,0135278
Axl	-0,34568	0,238258	-6,02639	7,77E-04	0,0023137
p70-S6K1	-0,34604	0,022775	-6,00728	7,91E-04	0,0023264
Tuberin_pT	-0,34867	0,137605	-6,78324	4,02E-04	0,0013797
B7-H4	-0,35764	0,196086	-7,28396	2,68E-04	9,60E-04
MIF	-0,35975	0,027556	-7,53573	2,21E-04	8,26E-04
53BP1	-0,36826	-0,440424	-2,78543	0,030084	0,0461539
Granzyme-	-0,36875	0,378485	-4,9506	0,002222	0,0053806
PKM2	-0,36988	0,193229	-5,66983	0,001084	0,0030082
Collagen_V	-0,38936	0,359319	-3,44023	0,012712	0,0216539
Mnk1	-0,39088	0,194965	-5,10519	0,001894	0,0049116
Merlin	-0,39201	-0,407113	-3,48727	0,011977	0,0206882
PAR	-0,41043	-0,597385	-3,94903	0,006799	0,0130871
b-Actin	-0,41302	-0,225716	-5,63988	0,001115	0,0030611
BRD4	-0,41397	0,114319	-6,88796	3,69E-04	0,001283
PDK1_pS24	-0,42338	0,016233	-6,74715	4,14E-04	0,0013834
ULK1_pS75	-0,42631	0,268754	-6,28088	6,19E-04	0,0019593

Atg3	-0,46667	-0,158672	-4,70623	6,58E-04	0,0023222
p44-42-MA	-0,48204	-0,120539	-3,22509	0,008167	0,0186778
B7-H4	-0,49165	0,008408	-8,41028	4,28E-06	6,21E-05
PKM2	-0,5135	-0,004977	-2,67252	0,021835	0,0421338
SHP-2_pY5	-0,52558	-0,033828	-5,68291	1,46E-04	6,45E-04
PDHK1	-0,53004	-0,09056	-9,83574	9,32E-07	2,09E-05
ULK1_pS75	-0,60626	0,028512	-6,83676	2,93E-05	2,07E-04
B-Raf	-0,62237	0,028469	-5,20918	2,98E-04	0,001188
TFAM	-0,63463	0,208887	-3,86464	0,002672	0,0076739
Axl	-0,64741	-0,084037	-7,38521	1,45E-05	1,24E-04
S6_pS240_	-0,69437	-0,196608	-1,51629	0,157871	0,2166337
WIPI2	-0,71436	0,135735	-3,69479	0,003582	0,00961
Collagen_V	-0,71767	0,005751	-7,86435	8,08E-06	8,92E-05
PKC-alpha	-0,72069	-0,189548	-3,85003	0,00274	0,0077785
MEK1	-0,73088	0,271173	-5,64225	1,55E-04	6,73E-04
Stat3_pY70	-0,7391	-0,067062	-7,84177	8,30E-06	8,92E-05
NDRG1_pT	-0,74879	0,569197	-2,08564	0,061304	0,0959359
Caveolin-1	-0,77492	0,094879	-2,19405	0,050804	0,0831027
HSP27_pS8	-0,81976	0,102724	-6,4187	5,14E-05	3,02E-04
PMS2	-0,83606	0,28608	-7,01512	2,32E-05	1,79E-04
IGFRb	-0,85389	-0,226573	-8,50891	3,82E-06	5,90E-05
LDHA	-0,86714	0,035181	-6,00222	9,21E-05	4,46E-04
TSC1	-0,88574	-0,037883	-6,09638	8,05E-05	4,06E-04
Atg7	-0,89547	0,079798	-6,81611	3,01E-05	2,07E-04
Granzyme-	-0,90222	-0,138443	-10,3752	5,48E-07	1,50E-05
AMPK_pT1	-0,98203	-0,097621	-5,5402	1,81E-04	7,69E-04
Glutaminas	-0,9895	-0,064521	-5,18491	3,10E-04	0,0012104
LC3A-B	-1,00888	0,145092	-9,86349	9,06E-07	2,09E-05
Gys_pS641	-1,07199	-0,079185	-15,6395	8,13E-09	5,02E-07
YAP_pS127	-1,07328	-0,052866	-6,0415	8,70E-05	4,30E-04
TFRC	-1,08901	0,387694	-5,3443	2,43E-04	0,0010155
Stat3	-1,17491	-0,383054	-4,7085	6,56E-04	0,0023222
MYH11	-1,17492	0,096037	-6,28033	6,22E-05	3,42E-04
p70S6K_pT	-1,23478	-0,039359	-6,59755	4,03E-05	2,62E-04
Cyclin_B1	-1,30657	0,212369	-12,9819	5,63E-08	2,32E-06
Gys	-1,34938	-0,101855	-18,948	1,07E-09	1,33E-07
Cox-IV	-1,37704	0,154718	-11,6248	1,74E-07	6,15E-06
Myosin-IIa_	-1,42752	0,113416	-7,65622	1,04E-05	1,03E-04
PLK1	-1,44787	0,096759	-9,42633	1,42E-06	2,91E-05
TRIM25	-1,51165	0,044582	-7,62208	1,08E-05	1,03E-04
S6_pS235_	-1,63268	-0,119137	-2,68167	0,021482	0,041779
MCT4	-2,06196	-0,369449	-8,01736	6,74E-06	8,49E-05
PDGFR-b	-4,12457	-0,927044	-45,2463	9,13E-14	2,25E-11

SHP-2_pY5	-0,44676	0,128133	-7,35017	2,55E-04	9,25E-04
LDHA	-0,44762	0,476085	-8,05559	1,50E-04	6,38E-04
Glutamate-	-0,45124	-0,004813	-8,2162	1,34E-04	6,00E-04
eEF2K	-0,4514	0,054703	-3,15247	0,018425	0,0301385
PREX1	-0,45485	0,110573	-4,87965	0,002394	0,0056852
4E-BP1	-0,47156	-0,159597	-7,88824	1,69E-04	6,86E-04
Cox2	-0,47292	-1,051796	-7,54332	2,19E-04	8,26E-04
P-Cadherin	-0,4765	0,075639	-5,44259	0,00135	0,0035975
Jagged1	-0,48167	-0,095837	-9,99258	4,19E-05	2,20E-04
MEK1	-0,48188	0,58004	-7,78747	1,83E-04	7,27E-04
Caveolin-1	-0,4844	0,438262	-4,86887	0,002421	0,0056956
MCT4	-0,49846	1,018534	-6,5153	5,04E-04	0,0016613
PEA15	-0,49975	0,049919	-3,19957	0,017324	0,028719
TFAM	-0,52316	0,414235	-6,14579	6,98E-04	0,0021397
WIPI2	-0,52497	0,405226	-2,88814	0,026181	0,0409279
VASP	-0,5466	-0,637872	-6,32922	5,93E-04	0,001902
YAP_pS127	-0,59724	0,466929	-11,6211	1,69E-05	1,16E-04
B-Raf	-0,61625	0,167933	-13,0013	8,51E-06	6,70E-05
Gys	-0,69854	0,583061	-13,0449	8,34E-06	6,70E-05
PKC-alpha	-0,70746	-0,024366	-13,1365	7,99E-06	6,70E-05
VEGFR-2	-0,73937	-0,022961	-13,2104	7,72E-06	6,70E-05
Gys_pS641	-0,75884	0,342594	-14,9396	3,62E-06	3,58E-05
PAK4	-0,77163	0,048855	-14,9624	3,59E-06	3,58E-05
S6_pS235_	-0,77487	0,751764	-4,92621	0,002279	0,0054662
Glutaminas	-0,77798	0,278284	-11,5562	1,75E-05	1,17E-04
FAK_pY397	-0,8357	0,097646	-12,5867	1,04E-05	7,77E-05
PMS2	-0,87153	0,44721	-10,5314	3,06E-05	1,80E-04
Atg7	-0,88232	0,28306	-18,5744	9,40E-07	1,11E-05
TFRC	-0,89641	0,740853	-5,06731	0,001969	0,0049116
HSP27_pS8	-0,91256	0,225903	-11,3629	1,93E-05	1,22E-04
p70S6K_pT	-0,94837	0,401891	-7,75291	1,87E-04	7,34E-04
LC3A-B	-1,03592	0,348992	-17,8253	1,21E-06	1,36E-05
TSC1	-1,05448	0,054131	-20,1759	5,62E-07	8,67E-06
MYH11	-1,14658	0,369384	-21,5179	3,76E-07	6,94E-06
Cyclin_B1	-1,16717	0,581082	-23,7859	2,01E-07	4,51E-06
NDRG1_pT	-1,23107	0,443207	-22,2046	3,09E-07	6,36E-06
Cox-IV	-1,23267	0,541782	-18,8318	8,63E-07	1,11E-05
PLK1	-1,26306	0,523547	-19,7823	6,35E-07	9,23E-06
Myosin-IIa_	-1,37016	0,459287	-10,0217	4,12E-05	2,20E-04
TRIM25	-1,37016	0,459287	-10,0217	4,12E-05	2,20E-04
p16_INK4a	-1,65739	-0,777213	-27,0609	8,95E-08	2,76E-06
Gab2	-1,68271	-0,540172	-27,826	7,52E-08	2,65E-06
PDGFR-b	-2,43191	0,988457	-45,7566	3,29E-09	2,03E-07

Table S7. Changes in protein expression and/or phosphorylation levels occurring in *MMTV-R26* ^{Met} MGT4 cells when exposed to single or combined treatment targeting BCL-XL (A1155463) and WEE1 (Adavosertib).

MGT4 + A11 + ADAV vs. MGT4 + A11

Protein	logFC	AveExpr	t	P.Value	adj.P.Val	B
PAR	2,628347	-0,447016	37,96932	9,93E-14	4,28E-11	22,00919
Histone-H3	1,565677	-0,22751	20,15493	1,59E-10	2,29E-08	14,67206
CDK1_pT14	1,240134	-0,263332	23,3577	2,90E-11	6,24E-09	16,42536
Caspase-7-cleaved-	1,200468	0,044123	18,16382	5,27E-10	3,78E-08	13,43042
p53	1,089415	0,265969	12,73397	2,90E-08	1,14E-06	9,21298
Caspase-3-cleaved	1,049189	-0,157487	12,46308	3,68E-08	1,32E-06	8,960888
DM-Histone-H3	0,923131	0,044083	13,51466	1,50E-08	6,45E-07	9,913311
JNK2	0,794061	0,368288	10,1844	3,32E-07	8,30E-06	6,627543
H2AX_pS140	0,79091	-0,061435	16,25579	1,87E-09	1,15E-07	12,10571
KAP1	0,786688	-0,155818	3,727147	0,002957	0,0144311	-2,94911
Vinculin	0,759792	-0,081115	2,868058	0,014318	0,0484051	-4,54142
p38-MAPK-_pT180_Y182	0,699031	0,014931	11,65955	7,66E-08	2,36E-06	8,18345
H2AX_pS139	0,627217	-0,076421	10,14326	3,47E-07	8,30E-06	6,581517
RBM15	0,586131	0,009748	6,343481	3,95E-05	4,86E-04	1,557214
Caveolin-1	0,497422	-0,149569	3,132231	0,008791	0,0347618	-4,05453
S6_pS235_S236	0,492271	0,525909	2,696065	0,019659	0,0596703	-4,85442
Cyclophilin-F	0,461977	-0,349202	3,065413	0,009945	0,0375981	-4,17819
SOD1	0,44144	-0,066911	7,220316	1,14E-05	1,97E-04	2,86881
ER-a_pS118	0,422159	-0,029565	4,553872	6,86E-04	0,0043459	-1,43986
CREB_pS133	0,416132	0,0246	5,047487	2,98E-04	0,0021796	-0,57152
p90RSK_pT573	0,409055	0,127706	2,299995	0,040483	0,1020363	-5,55473
MAPK_pT202_Y204	0,372642	0,259927	3,404308	0,005328	0,0244283	-3,54909
AMPKa_pT172	0,369015	0,069402	6,31148	4,14E-05	4,95E-04	1,507402
Bcl2	0,36725	0,005942	3,090251	0,0095	0,0363294	-4,13225
S6	0,350749	-0,221281	2,27055	0,042686	0,1063461	-5,60527
TRAP1	0,348811	-0,205144	3,026304	0,01069	0,0390462	-4,25044
MLH1	0,344489	-0,022308	4,364849	9,51E-04	0,0056929	-1,77969
Claudin-7	0,334078	-0,262171	3,942495	0,002006	0,0107897	-2,55099
Caspase-8	0,325171	-0,049334	2,832361	0,015293	0,0503401	-4,60671
c-Jun_pS73	0,324502	-0,075111	4,255394	0,001152	0,0067998	-1,97811
C-Raf_pS338	0,313519	-0,094016	3,878419	0,002251	0,0116171	-2,66918
ATP5A	0,288493	-0,041445	3,192491	0,007866	0,0326002	-3,94279
Myosin-IIa	0,276154	0,125587	1,109796	0,289133	0,4311983	-7,28761
MTCO1	0,273638	0,022592	1,95599	0,07449	0,1583829	-6,12722
MDM2_pS166	0,271517	0,002366	3,674281	0,003254	0,0155852	-3,04718
Tau	0,268593	0,023815	3,868508	0,002291	0,0116171	-2,68748
GRB7	0,257698	-0,033547	3,184705	0,00798	0,0327566	-3,95724
MIG6	0,254672	-0,034288	4,738494	5,00E-04	0,0033696	-1,1117
Bad_pS112	0,252335	0,10143	5,30182	1,97E-04	0,0016013	-0,13596
Wee1_pS642	0,250104	0,077241	4,470019	7,92E-04	0,0049491	-1,59016
SF2	0,248546	-0,07376	2,060932	0,061995	0,1406317	-5,95706
Mitofusin-1	0,242395	0,041273	1,385346	0,191507	0,3275377	-6,95901
VHL-EPPK1	0,24024	-0,032711	1,062236	0,309329	0,4488914	-7,33848
IRF-1	0,235718	0,017763	2,498176	0,028256	0,0787308	-5,20861
EGFR	0,232973	0,023382	3,722864	0,00298	0,0144311	-2,95705
Ambra1_pS52	0,22697	0,096777	2,945236	0,012417	0,0438658	-4,3998
Annexin-VII	0,224964	-0,018589	2,973672	0,011782	0,0423152	-4,34747
SOD2	0,220183	0,043969	2,537959	0,026275	0,0749969	-5,13801
cdc25C	0,219803	0,066872	3,056027	0,010119	0,0375981	-4,19554
Caspase-8-cleaved	0,212609	-0,011594	3,900048	0,002165	0,0113787	-2,62926
Fibronectin	0,212218	0,223085	3,165356	0,00827	0,0333121	-3,99313
ADAR1	0,207581	-0,023956	2,583851	0,024158	0,0708299	-5,05617
PAX8	0,206482	0,134307	2,679777	0,020257	0,0610549	-4,88384
eIF4E_pS209	0,204506	0,048507	4,403309	8,89E-04	0,0053993	-1,71025
GATA3	0,204245	-0,050683	2,249395	0,04434	0,1098298	-5,64141
DNA-Ligase-IV	0,202696	0,096328	1,72486	0,11055	0,2127097	-6,48506
Cyclin-B1	0,199964	-5,99E-04	2,47646	0,029398	0,0801925	-5,247
EPHA2	0,197891	-0,649423	4,186227	0,001301	0,0075762	-2,10406
Stathmin-1	0,186112	-0,093131	3,249935	0,007076	0,0307184	-3,83613
p70-S6K_pT389	0,185756	0,269091	1,486528	0,163293	0,2849368	-6,82504
SETD2	0,183254	-0,035564	2,919092	0,013031	0,0456613	-4,44785
Collagen-VI	0,18283	0,063135	2,602209	0,023358	0,0694306	-5,02332
PKC-b-II_pS660	0,182643	0,022917	1,629754	0,129467	0,2374481	-6,62459
ATM	0,182023	0,084602	1,735465	0,108601	0,2117958	-6,4692
14-3-3-beta	0,177872	0,022584	2,057712	0,062348	0,1406908	-5,96235
DUSP4	0,17682	-0,042366	1,63932	0,127443	0,2347347	-6,61078
RIP3	0,169619	-0,015111	2,76372	0,017357	0,0548696	-4,73178
D-a-Tubulin	0,165056	0,01165	2,771548	0,017108	0,0548696	-4,71755
Aurora-A	0,164932	0,026589	2,274496	0,042385	0,106208	-5,59851
N-Ras	0,160593	-0,002013	2,913742	0,01316	0,0457428	-4,45767

MGT4 + A11 + ADAV vs. MGT4 + ADAV

Protein	logFC	AveExpr	t	P.Value	adj.P.Val	B
PAR	1,77538	-0,447016	25,64729	9,77E-12	4,21E-09	17,41064
Histone-H3	1,24939	-0,22751	16,08338	2,11E-09	4,54E-07	12,17029
Caspase-3-	0,923867	-0,157487	10,97441	1,48E-07	1,60E-05	7,791781
Caspase-7-	0,852249	0,044123	12,89506	2,52E-08	3,63E-06	9,630045
DM-Histon	0,679048	0,044083	9,941275	4,30E-07	3,70E-05	6,681096
KAP1	0,640602	-0,155818	3,035028	0,010519	0,1237839	-3,82686
HES1	0,614045	-0,136402	2,333775	0,038089	0,2534579	-5,08557
Histone-H3	0,476422	0,032165	6,248991	4,54E-05	0,002023	1,790387
H2AX_pS14	0,413435	-0,061435	8,497446	2,22E-06	1,60E-04	4,957271
H2AX_pS13	0,399667	-0,076421	6,46336	3,31E-05	0,0017839	2,120802
ER-a_pS11	0,368771	-0,029565	3,97797	0,001883	0,0450839	-2,08416
Aurora-AB	0,359293	0,130098	4,511615	7,37E-04	0,0244475	-1,11808
ERRalpha	0,348421	-0,060608	6,226119	4,69E-05	0,002023	1,75476
CREB_pS13	0,316702	0,0246	3,841448	0,002405	0,0518363	-2,33502
Bcl2	0,303528	0,005942	2,554058	0,025512	0,1982695	-4,69945
Cdc6	0,285005	0,183566	4,31231	0,001042	0,0293093	-1,47578
Caveolin-1	0,265426	-0,149569	1,671369	0,120864	0,4616099	-6,15086
Stathmin-1	0,25082	-0,093131	4,37989	9,26E-04	0,0285206	-1,35404
RBM15	0,242631	0,009748	2,625912	0,022364	0,1939187	-4,57124
MLH1	0,241882	-0,022308	3,064759	0,009957	0,1226175	-3,77209
Cyclophilin	0,227372	-0,349202	1,508709	0,157604	0,5031641	-6,38106
Sox2	0,223536	0,345513	3,856503	0,002341	0,0518363	-2,3073
SOD1	0,222717	-0,066911	3,642829	0,003446	0,0550062	-2,70177
Cox2	0,219464	0,165493	1,294413	0,220215	0,553027	-6,65926
c-Myc	0,211255	-0,068852	1,897212	0,082467	0,3664253	-5,80799
SOD2	0,195935	0,043969	2,258454	0,043624	0,2645265	-5,21476
PARP	0,194419	-0,075124	3,713172	0,003033	0,0522848	-2,57168
Caspase-8	0,193593	-0,049334	1,686268	0,117908	0,4616099	-6,12903
Fibronectir	0,193584	0,223085	2,887415	0,013816	0,1452348	-4,0977
S6	0,190408	-0,221281	1,232593	0,241658	0,5808818	-6,73365
PAX8	0,182963	0,134307	2,374533	0,035381	0,2450007	-5,01501
TRAP1	0,178947	-0,205144	1,552559	0,146854	0,4944847	-6,32052
ATP5A	0,175825	-0,041445	1,945699	0,075833	0,3514424	-5,73128
MITF	0,175276	0,104632	3,294253	0,006522	0,0878406	-3,34778
E-Cadherin	0,172151	-0,718665	0,390062	0,703421	0,9060647	-7,42456
Claudin-7	0,166088	-0,262171	1,960017	0,07397	0,3465357	-5,70844
MTCO1	0,161766	0,022592	1,156321	0,270364	0,6298742	-6,82152
GRB7	0,15997	-0,033547	1,97695	0,071822	0,3439474	-5,68132
Caspase-8-	0,159073	-0,011594	2,918004	0,013057	0,1442984	-4,04174
S100A4	0,15705	0,323293	2,0956	0,058317	0,3105905	-5,48809
Mcl-1	0,151739	0,126503	1,999326	0,069071	0,3382926	-5,6453
eEF2K	0,149796	0,045657	1,455442	0,171561	0,5186339	-6,45301
IRF-1	0,149114	0,017763	1,580331	0,140378	0,4944847	-6,28158
DNA_POLG	0,141829	0,048448	1,331618	0,208071	0,5484457	-6,61318
C-Raf_pS33	0,141795	-0,094016	1,754088	0,105252	0,4161802	-6,0282
EGFR	0,141214	0,023382	2,256576	0,043772	0,2645265	-5,21796
Bcl-xL	0,139267	-0,052409	3,339261	0,006004	0,0834711	-3,26437
GATA3	0,138063	-0,050683	1,520525	0,154643	0,502496	-6,36486
Mitofusin-1	0,136821	0,041273	0,781969	0,449599	0,8074041	-7,18398
SDHA	0,132624	-0,103483	1,987231	0,070546	0,3416321	-5,66479
Bid	0,127081	0,009543	1,777647	0,101149	0,4074308	-5,99263
b-Actin	0,124978	-0,123301	1,32571	0,209962	0,5484457	-6,62056
Annexin-I	0,12349	-0,035592	1,300454	0,218205	0,553027	-6,65185
ACC_pS79	0,119903	-0,237276	1,313618	0,213876	0,5519799	-6,6356
Cox-IV	0,119143	-0,046332	0,595235	0,562898	0,8406864	-7,31756
Annexin-VI	0,118431	-0,018589	1,565464	0,143813	0,4944847	-6,30248
SF2	0,115018	-0,07376	0,953722	0,359303	0,7221004	-7,03256
IGFBP2	0,10919	-0,058578	1,465885	0,168745	0,5158084	-6,43905
Transglutar	0,108853	0,012303	0,825141	0,425615	0,787296	-7,14842
Tyro3	0,108323	0,007668	1,881758	0,084691	0,3724655	-5,83223
Heregulin	0,107308	0,529459	1,337036	0,206349	0,5484457	-6,60639
DM-K9-His	0,105642	0,012197	1,29297	0,220698	0,553027	-6,66103
Cyclin-B1	0,103239	-5,99E-04	1,278566	0,22556	0,5561111	-6,67859
Coup-TFII	0,102543	0,178384	1,444853	0,174457	0,5186339	-6,46711
PARG	0,101702	2,89E-04	1,46844	0,168062	0,5158084	-6,43562
Collagen-V	0,100183	0,063135	1,425896	0,179744	0,5199318	-6,49215
YAP	0,098425	0,05181	1,853687	0,08887	0,3783138	-5,87597
XRCC1	0,097395	-0,039169	1,241531	0,238459	0,5773912	-6,72306
Aurora-A	0,097341	0,026589	1,342384	0,20466	0,5484457	-6,59966
TFAM	0,096845	0,020213	1,434267	0,177393	0,5186339	-6,48112

Notch1-cleaved	0,158484	-0,006006	3,237015	0,007247	0,0307184	-3,86013
Bid	0,156599	0,009543	2,19056	0,049264	0,1186182	-5,74123
DNMT1	0,154231	-0,244014	2,403719	0,033557	0,0886222	-5,3748
Notch3	0,153731	-0,012206	3,150047	0,008507	0,0339491	-4,02151
PARG	0,149382	2,89E-04	2,156876	0,052311	0,1232036	-5,79787
CDT1	0,148304	0,052388	2,370184	0,035661	0,0920362	-5,43329
c-Myc	0,147521	-0,068852	1,324838	0,210242	0,3481555	-7,03587
YB1_pS102	0,145909	-0,035683	2,504083	0,027953	0,0787308	-5,19815
Src	0,145128	-0,207681	2,113457	0,056503	0,1309293	-5,87032
YAP	0,144603	0,05181	2,723384	0,018695	0,0575552	-4,80498
Cdc42	0,143783	0,025328	3,121586	0,008966	0,0351291	-4,07425
Transglutaminase	0,143116	0,012303	1,084873	0,299588	0,4421999	-7,3145
CD49b	0,14114	-0,01632	3,030203	0,010613	0,0390462	-4,24324
COG3	0,140287	0,05772	2,201553	0,048306	0,1169655	-5,72266
ACC_pS79	0,137803	-0,237276	1,509724	0,157347	0,2756776	-6,79341
ACVRL1	0,136731	-2,20E-04	2,577992	0,024419	0,0710245	-5,06665
Syk	0,135082	0,002397	2,203825	0,04811	0,1169655	-5,71882
Annexin-I	0,130742	-0,035592	1,37682	0,19406	0,3305924	-6,96999
MSI2	0,130695	-0,049706	1,753761	0,10531	0,2072541	-6,44171
Enolase-1	0,130538	-0,337237	1,671938	0,12075	0,2272622	-6,5633
Rb	0,129048	0,015017	2,10733	0,057119	0,1316497	-5,88049
MERIT40_pS29	0,127443	-0,058898	1,981759	0,071222	0,1543987	-6,08584
PR	0,124794	0,032851	1,604207	0,135011	0,2455262	-6,66122
FOXM1	0,120926	0,001348	2,420301	0,032562	0,0871685	-5,34578
S6_pS240_S244	0,116998	0,524502	0,984701	0,344479	0,4821139	-7,41742
PYGM	0,115132	-0,031874	1,512618	0,156619	0,2755208	-6,78944
p21	0,115076	0,016374	1,64396	0,126471	0,233945	-6,60406
ATR_pS428	0,113369	-0,007961	1,726828	0,110186	0,2127097	-6,48212
CA9	0,112877	0,007759	1,93114	0,077772	0,1643119	-6,16685
CD4	0,11198	0,001958	0,807017	0,435579	0,5758734	-7,57877
CtIP	0,111624	-0,049514	2,168387	0,05125	0,1220383	-5,77856
XBP-1	0,111304	0,073656	2,002886	0,068643	0,1517183	-6,05172
GATA6	0,11099	0,007955	1,729934	0,109613	0,2127097	-6,47748
MRAP	0,11001	0,030253	1,90027	0,082033	0,171633	-6,21572
VHL	0,10966	0,077473	1,514189	0,156224	0,2755208	-6,78729
DAPK1_pS308	0,108951	0,013646	1,995388	0,069548	0,1521588	-6,06385
BiP-GRP78	0,106921	0,01833	1,916699	0,079739	0,1676473	-6,18977
Rheb	0,106119	-0,006062	2,004555	0,068443	0,1517183	-6,04902
4E-BP1	0,105809	-0,121582	1,78429	0,100018	0,2014382	-6,39547
Chk1_pS296	0,104451	0,030022	1,066203	0,307605	0,4488914	-7,33431
HER3	0,099278	-0,121268	2,08075	0,059867	0,1365218	-5,92445
PRAS40	0,096575	-0,009817	1,849889	0,08945	0,1844629	-6,29457
Complex-II-Subunit	0,093413	-0,044895	1,761166	0,104004	0,206595	-6,43054
Rad50	0,09257	-0,199697	1,149163	0,273189	0,4160578	-7,24415
DNA_POLG	0,091061	0,048448	0,854964	0,409549	0,553341	-7,538
IGFBP2	0,090832	-0,058578	1,219425	0,246434	0,3849555	-7,16359
PD-L1	0,089645	0,04206	0,910622	0,380672	0,5241836	-7,48808
NAPSIN-A	0,089171	-0,101967	1,385731	0,191392	0,3275377	-6,95851
Chk2_pT68	0,087271	-0,002757	1,324465	0,210362	0,3481555	-7,03633
14-3-3-zeta	0,086245	-0,152677	1,296713	0,219448	0,3583302	-7,07073
Aurora-B	0,085383	0,031936	1,371811	0,195573	0,3318584	-6,97642
CIITA	0,084159	-0,028549	1,312669	0,214186	0,3523437	-7,05102
BAP1	0,08283	0,003482	1,65086	0,125039	0,233297	-6,59405
Cyclin-D1	0,081793	0,034803	1,015438	0,330212	0,4728286	-7,38673
VEGF-164	0,081308	0,03077	1,646642	0,125913	0,2339157	-6,60017
IL-6	0,077952	-0,01241	1,323006	0,210832	0,3481555	-7,03815
CD31	0,077369	0,01401	1,611638	0,133377	0,2435831	-6,65061
PDH	0,074612	0,038497	1,3871	0,190985	0,3275377	-6,95674
HSP27	0,07056	-0,024798	1,145432	0,27467	0,4168409	-7,24832
MIF	0,06894	-0,020019	1,455836	0,171454	0,2967739	-6,86638
Bim	0,06879	-0,479038	0,546765	0,594702	0,7184027	-7,76222
DM-K9-Histone-H3	0,065462	0,012197	0,801204	0,438807	0,5783662	-7,58357
PKC-a-b-II_pT638_T641	0,06133	0,193816	1,012725	0,331454	0,473035	-7,38947
MEK2	0,061318	-0,028089	1,201854	0,252923	0,3893209	-7,18409
HSP70	0,061097	-0,104212	1,115365	0,286835	0,4307522	-7,28154
Calnexin	0,060677	-0,064072	1,069006	0,306391	0,4488914	-7,33135
SOX17	0,060094	-0,001645	1,23338	0,241375	0,382473	-7,14715
GCLM	0,058653	0,022262	1,009472	0,332947	0,4735979	-7,39275
Bak	0,058551	-0,029684	1,035187	0,321276	0,4646641	-7,36659
FASN	0,055383	-0,105082	0,906937	0,382539	0,5250773	-7,49147
Hexokinase-I	0,055313	0,008558	1,064861	0,308187	0,4488914	-7,33572
PAI-1	0,055135	-0,036353	0,850796	0,41177	0,5546027	-7,54163
Patched	0,053392	-0,044418	0,814183	0,431622	0,5723966	-7,57281
TFAM	0,053257	0,020213	0,788736	0,445783	0,5857693	-7,59376
Smad4	0,052687	-0,014953	0,936875	0,367553	0,5081363	-7,46358
CDKN2A	0,051102	0,017704	0,593121	0,564265	0,6988459	-7,73437
AceCS1	0,05108	0,175013	0,574185	0,576596	0,7040022	-7,746

Lck	0,095801	-0,0272	1,382813	0,192263	0,5355361	-6,5482
CD44	0,091911	-0,048103	1,116863	0,28622	0,6492664	-6,86522
DUSP4	0,090852	-0,042366	0,8423	0,416321	0,7788793	-7,13381
GATA6	0,08737	0,007955	1,361785	0,198631	0,5484457	-6,5751
Vinculin	0,081391	-0,081115	0,307234	0,764006	0,9216856	-7,45546
COG3	0,079435	0,05772	1,246599	0,236659	0,5762717	-6,71704
Chk1_pS29	0,079142	0,030022	0,807856	0,435114	0,7946369	-7,16286
Hexokinase	0,077651	-0,321777	0,604989	0,556611	0,8406864	-7,31142
Ambra1_pS	0,076344	0,096777	0,990665	0,341677	0,7012504	-6,99661
Cdc42	0,075523	0,025328	1,639634	0,127377	0,4628801	-6,19695
CD171	0,074913	-0,179758	0,339746	0,740003	0,9216856	-7,44419
CD86	0,074342	-0,059701	1,297426	0,219211	0,553027	-6,65557
Enolase-1	0,074317	-0,337237	0,951851	0,360212	0,7221004	-7,03435
Notch1-cle	0,074168	-0,006006	1,514867	0,156054	0,502496	-6,37263
YAP_pS127	0,073161	0,902732	0,610637	0,552989	0,8406864	-7,30782
RPA32	0,071498	-0,242082	1,134492	0,27905	0,6431574	-6,84585
DNA-Ligase	0,071404	0,096328	0,607623	0,554921	0,8406864	-7,30974
c-Jun_pS73	0,070647	-0,075111	0,926444	0,372726	0,7369042	-7,05836
D-a-Tubulir	0,070607	0,01165	1,185596	0,259046	0,6101032	-6,78831
4E-BP1	0,06702	-0,121582	1,130174	0,280793	0,643733	-6,85062
FN14	0,067017	0,021789	0,89254	0,389895	0,7549078	-7,08951
U-Histone-	0,066739	0,011354	0,599676	0,560031	0,8406864	-7,31477
SETD2	0,065918	-0,035564	1,050022	0,314682	0,676246	-6,93644
Grp75	0,064968	-0,171403	1,010149	0,332636	0,6925892	-6,97718
SOX17	0,064347	-0,001645	1,320654	0,211591	0,5493724	-6,62686
RIP3	0,063802	-0,015111	1,039567	0,319318	0,676246	-6,94725
Snail	0,06212	0,389254	0,840201	0,41745	0,7788793	-7,13561
CDKN2A	0,061682	0,017704	0,715931	0,487923	0,838916	-7,23503
Tuberin_p1	0,061148	0,059566	0,778142	0,451765	0,8079288	-7,18705
MERIT40_p	0,05818	-0,058898	0,904712	0,383669	0,7529167	-7,07844
Aurora-B	0,057969	0,031936	0,931361	0,370281	0,7354429	-7,05376
p27-Kip1	0,057885	0,01314	0,643284	0,53231	0,8406864	-7,2864
PREX1	0,05325	0,008897	0,796223	0,441585	0,7988712	-7,17243
VHL-EPPK1	0,052152	-0,032711	0,230596	0,821568	0,9334929	-7,47761
ADAR1	0,050936	-0,023956	0,634024	0,53813	0,8406864	-7,29258
AR	0,050638	0,027125	0,903435	0,384319	0,7529167	-7,07961
EPHA2	0,049966	-0,649423	1,05699	0,31162	0,6761695	-6,92918
Rad50	0,049587	-0,199697	0,615577	0,549831	0,8406864	-7,30464
N-Ras	0,048613	-0,002013	0,88201	0,395336	0,7549078	-7,09898
Wee1_pS6	0,048258	0,077241	0,862498	0,405556	0,7632947	-7,11627
cdc25C	0,047852	0,066872	0,665306	0,518614	0,8406864	-7,27136
IR-b	0,047754	-0,023481	0,437321	0,669754	0,8984516	-7,40372
G6PD	0,046291	-0,105384	0,479332	0,64044	0,8984516	-7,38327
Hexokinase	0,046108	0,008558	0,887644	0,392419	0,7549078	-7,09393
BiP-GRP78	0,045527	0,01833	0,816135	0,430547	0,7896418	-7,15598
SLC1A5	0,044073	-0,020076	0,337309	0,741792	0,9216856	-7,44507
Tau	0,043839	0,023815	0,631406	0,539782	0,8406864	-7,29431
SCD	0,042921	0,046091	0,293097	0,774525	0,9216856	-7,46002
CD4	0,04268	0,001958	0,307589	0,763742	0,9216856	-7,45534
Hif-1-alpha	0,041821	0,203091	0,392344	0,70178	0,9060647	-7,42361
HSP70	0,041687	-0,104212	0,761019	0,461544	0,8134175	-7,20062
PRAS40	0,041436	-0,009817	0,793706	0,442994	0,7988712	-7,17448
Bim	0,040894	-0,479038	0,325037	0,750828	0,9216856	-7,44943
Rb	0,039722	0,015017	0,648649	0,528954	0,8406864	-7,28278
PD-1	0,038567	0,012291	0,678756	0,510351	0,8406864	-7,26195
Complex-II	0,038169	-0,044895	0,719624	0,485728	0,838916	-7,23228
GCLM	0,037951	0,022262	0,653179	0,52613	0,8406864	-7,2797
Syk	0,03754	0,002397	0,612455	0,551825	0,8406864	-7,30665
P-Cadherin	0,037431	0,004036	0,400759	0,69574	0,9060647	-7,42005
CD49b	0,037159	-0,01632	0,797779	0,440716	0,7988712	-7,17116
HSP27	0,036715	-0,024798	0,596016	0,562393	0,8406864	-7,31707
Cyclin-E1	0,036164	0,131132	0,413665	0,686518	0,9058509	-7,41444
Myosin-IIa	0,034994	0,125587	0,140632	0,890526	0,9453616	-7,49561
STING	0,034502	0,244125	0,152961	0,881006	0,9446507	-7,49366
Cyclin-D3	0,034441	0,043783	0,46426	0,650887	0,8984516	-7,39081
FOXM1	0,033707	0,001348	0,674636	0,512873	0,8406864	-7,26485
Merlin	0,032685	-0,057375	0,380642	0,710215	0,9083162	-7,42844
Notch3	0,030738	-0,012206	0,62983	0,540778	0,8406864	-7,29535
MEK1	0,030651	-0,124464	0,435224	0,671233	0,8984516	-7,4047
RRM1	0,030246	-0,036564	0,552337	0,590999	0,8634592	-7,34345
BAP1	0,029835	0,003482	0,594628	0,56329	0,8406864	-7,31794
PD-L1	0,029369	0,04206	0,298333	0,770623	0,9216856	-7,45835
CDT1	0,028754	0,052388	0,459549	0,654169	0,8984516	-7,39312
CD29	0,028668	0,008186	0,539145	0,599785	0,8733357	-7,35104
LRP6_pS14	0,027337	0,099524	0,369061	0,718602	0,9115925	-7,43307
CA9	0,027105	0,007759	0,463726	0,651259	0,8984516	-7,39108
ERCC5	0,026896	0,04876	0,306631	0,764453	0,9216856	-7,45566

c-Abl	0,048813	0,06195	0,666521	0,517864	0,6584053	-7,68593
AR	0,047109	0,027125	0,840475	0,417303	0,5603042	-7,55054
PI3K-p110-b	0,044352	-0,043716	0,696791	0,499395	0,640593	-7,66442
DAPK2	0,041487	0,006769	0,828256	0,423917	0,5639149	-7,56097
MelanA	0,040197	-0,07913	0,498708	0,627127	0,7364894	-7,78881
E2F1	0,039477	-0,015219	0,67728	0,511254	0,6519245	-7,67838
N-Cadherin	0,039105	0,036274	0,749226	0,468355	0,6080152	-7,6251
SDHA	0,037689	-0,103483	0,564726	0,582808	0,709577	-7,75168
UQCRC2	0,036529	-0,059451	0,777295	0,452246	0,5906607	-7,60299
WIPI2	0,03641	-0,02897	0,276899	0,786636	0,857131	-7,88071
HLA-DR-DP-DQ-DX	0,035343	-0,01581	0,62175	0,5459	0,6870819	-7,7161
PD-1	0,034964	0,012291	0,615337	0,549984	0,6870819	-7,72026
IRS2	0,03452	0,497198	0,366643	0,720358	0,8191933	-7,84971
Jagged1	0,034389	-0,002552	0,762569	0,460653	0,5998229	-7,61468
BRD4	0,034241	-0,067661	0,390973	0,702766	0,8034269	-7,83985
14-3-3-epsilon	0,033517	0,009189	0,537422	0,600937	0,7234746	-7,76757
YAP_pS127	0,03327	0,902732	0,277689	0,786044	0,857131	-7,88048
Cox-IV	0,03326	-0,046332	0,166166	0,870832	0,9148983	-7,90717
XPA	0,03231	0,037377	0,531151	0,605142	0,7256483	-7,77111
Bim_1	0,031572	-0,20752	0,385155	0,706957	0,8060805	-7,84226
CD134	0,030915	-5,21E-04	0,462164	0,652347	0,7598955	-7,80746
Cdc6	0,029744	0,183566	0,450054	0,660806	0,7676748	-7,81334
Gli1	0,026904	0,045725	0,472737	0,645001	0,7533757	-7,8022
PDHK1	0,026736	-0,003486	0,43086	0,674315	0,7812626	-7,82235
Histone-H3_pS10	0,025865	0,032165	0,339264	0,740356	0,8288143	-7,86007
XRCC1	0,025248	-0,039169	0,321849	0,753182	0,8388149	-7,86625
CD38	0,025135	-0,02424	0,30849	0,763073	0,8454618	-7,87077
MEK1_p_S217_S221	0,022659	0,022895	0,349353	0,732963	0,8248228	-7,85634
Grp75	0,019198	-0,171403	0,298493	0,770504	0,8515054	-7,87403
TFRC	0,018874	-0,023369	0,199007	0,845636	0,90023	-7,90069
Enolase-2	0,018453	-7,87E-04	0,278176	0,785679	0,857131	-7,88033
SFRP1	0,018111	0,054441	0,343537	0,737222	0,8274549	-7,85851
S100A4	0,017364	0,323293	0,231702	0,820728	0,8821289	-7,89309
Myt1	0,01455	-0,055936	0,242365	0,812647	0,8756272	-7,89037
AMPK-a2_pS345	0,012675	-0,111954	0,111822	0,912839	0,9367472	-7,91533
Ets-1	0,010683	-0,022047	0,226552	0,824631	0,8841192	-7,89436
P-Cadherin	0,008282	0,004036	0,088668	0,930829	0,9484329	-7,91785
TAZ	0,004935	0,101754	0,093992	0,926689	0,946452	-7,91732
UGT1A	0,004202	0,268485	0,061073	0,952321	0,963498	-7,92008
TUFM	0,002065	0,039828	0,025695	0,979929	0,9844974	-7,92174
CD29	0,001455	0,008186	0,027363	0,978626	0,9844974	-7,9217
Granzyme-B	-1,43E-05	0,194614	-1,67E-04	0,99987	0,9998699	-7,9221
CD44	-0,00108	-0,048103	-0,01308	0,989782	0,9920842	-7,92201
PREX1	-0,00274	0,008897	-0,04091	0,968053	0,9771211	-7,9212
CD20	-0,00443	-0,016963	-0,06167	0,951859	0,963498	-7,92004
Creb	-0,00689	-0,033283	-0,11874	0,907476	0,9366229	-7,91447
Rab11	-0,00727	-0,004335	-0,11758	0,908372	0,9366229	-7,91462
NDUFB4	-0,00742	-0,047676	-0,0856	0,933217	0,9486234	-7,91813
Glutaminase	-0,00773	0,32211	-0,10419	0,918765	0,9405882	-7,91623
LC3A-B	-0,00793	-0,040287	-0,15307	0,880923	0,9215483	-7,90943
RIP	-0,00839	0,030057	-0,12257	0,904506	0,9366229	-7,91397
p27_pT157	-0,00864	0,027337	-0,16407	0,872444	0,9148983	-7,90754
Puma	-0,01034	-0,083658	-0,12816	0,900173	0,9348782	-7,91321
MMP2	-0,01059	-0,02074	-0,18605	0,855555	0,9050349	-7,90338
CD45	-0,01091	-0,03593	-0,13195	0,897239	0,934082	-7,91268
Notch1	-0,01174	-0,054826	-0,1945	0,849086	0,9013696	-7,90165
HSP60	-0,01259	-0,120894	-0,19863	0,845924	0,90023	-7,90077
NQO1	-0,01405	-0,013101	-0,11318	0,911783	0,9367472	-7,91517
Glutamate-D1-2	-0,01573	0,097722	-0,357	0,727381	0,8206834	-7,85345
U-Histone-H2B	-0,01654	0,011354	-0,14862	0,884354	0,9228969	-7,91015
PAK1	-0,01795	0,121987	-0,31199	0,760475	0,8447543	-7,8696
Lck	-0,0183	-0,0272	-0,26408	0,796261	0,8622829	-7,88444
TRIP13	-0,01846	-0,047949	-0,27571	0,787526	0,857131	-7,88107
ERCC5	-0,01861	0,04876	-0,21214	0,835612	0,8936689	-7,89778
FOXO3	-0,01893	0,039835	-0,18357	0,857459	0,9050349	-7,90388
LRP6_pS1490	-0,02046	0,099524	-0,27617	0,787181	0,857131	-7,88093
ATM_pS1981	-0,022	-0,044366	-0,26952	0,792169	0,860012	-7,88288
c-Kit	-0,02353	-0,017567	-0,3334	0,744666	0,8314791	-7,86219
SCD	-0,02662	0,046091	-0,18177	0,858838	0,9050349	-7,90423
Porin	-0,02718	-0,05308	-0,583	0,570837	0,7018063	-7,74063
RPA32_pS4_S8	-0,02793	0,003	-0,42086	0,6814	0,7857182	-7,82689
INPP4b	-0,02835	-0,042678	-0,57718	0,574635	0,7036018	-7,74418
VASP	-0,02857	0,087681	-0,42029	0,681807	0,7857182	-7,82715
Mnk1	-0,02874	0,189059	-0,35786	0,726748	0,8206834	-7,85312
p38-a	-0,03069	-0,037713	-0,59052	0,565952	0,6989258	-7,73599
ENY2	-0,03274	-0,11689	-0,54623	0,595057	0,7184027	-7,76252
Beclin	-0,03315	0,065552	-0,72247	0,484039	0,6246131	-7,64549

Mnk1	0,026279	0,189059	0,327209	0,749226	0,9216856	-7,44867
PR	0,024936	0,032851	0,320548	0,754143	0,9216856	-7,45098
XBP-1	0,024663	0,073656	0,4438	0,665194	0,8984516	-7,40069
HER3	0,024184	-0,121268	0,506872	0,621558	0,8900047	-7,36888
MEK1_p_S	0,024074	0,022895	0,371172	0,71707	0,9115925	-7,43224
PDH	0,023612	0,038497	0,438967	0,668594	0,8984516	-7,40295
Chk1	0,022605	-0,197309	0,395465	0,699537	0,9060647	-7,42229
ATR_pS428	0,022486	-0,007961	0,342503	0,73798	0,9216856	-7,44318
NAPSIN-A	0,021669	-0,101967	0,336743	0,742208	0,9216856	-7,44528
MRAP	0,019173	0,030253	0,33119	0,746293	0,9216856	-7,44726
A-Raf_pS29	0,018922	0,023098	0,284755	0,780755	0,9216856	-7,4626
NRF2	0,018762	-0,109994	0,230723	0,821471	0,9334929	-7,47758
eIF4E	0,017942	0,084361	0,279318	0,784823	0,9216856	-7,46425
14-3-3-beta	0,01776	0,022584	0,205453	0,840711	0,9334929	-7,48351
PDHK1	0,017523	-0,003486	0,282392	0,782522	0,9216856	-7,46332
PYGM	0,015961	-0,031874	0,209693	0,837474	0,9334929	-7,48256
BRD4	0,015721	-0,067661	0,179509	0,860576	0,9390078	-7,48889
N-Cadherin	0,013995	0,036274	0,268145	0,793206	0,9236947	-7,46754
MIG6	0,013683	-0,034288	0,254587	0,803413	0,9283403	-7,47135
c-Abl	0,013308	0,06195	0,181709	0,858887	0,9390078	-7,48847
Src	0,012614	-0,207681	0,183699	0,85736	0,9390078	-7,48807
VHL	0,012525	0,077473	0,172944	0,865619	0,9407674	-7,49014
DAPK1_pS3	0,012352	0,013646	0,226228	0,824885	0,9334929	-7,47868
INPP4b	0,012192	-0,042678	0,248189	0,808243	0,9314245	-7,47308
IRS1	0,011322	0,130579	0,149376	0,883773	0,9446507	-7,49424
MIF	0,010669	-0,020019	0,225312	0,825581	0,9334929	-7,4789
Chk2	0,009345	0,017318	0,079352	0,938079	0,9695736	-7,50289
Jagged1	0,009333	-0,002552	0,206953	0,839566	0,9334929	-7,48318
Bak	0,008164	-0,029684	0,144345	0,887658	0,9446507	-7,49504
UQCRC2	0,007903	-0,059451	0,168173	0,869287	0,9407674	-7,49102
MelanA	0,007046	-0,07913	0,087413	0,931806	0,9677306	-7,50216
Calnexin	0,006476	-0,064072	0,114087	0,911082	0,9634726	-7,49926
Rab11	0,006038	-0,004335	0,097628	0,923862	0,9664673	-7,50114
IL-6	0,002941	-0,01241	0,04991	0,961027	0,9815226	-7,50495
4E-BP1_pS	0,002867	0,087254	0,06086	0,952486	0,9797651	-7,50429
Ets-1	0,002202	-0,022047	0,0467	0,963531	0,9817537	-7,50511
HLA-DR-DP	0,002019	-0,01581	0,035511	0,972264	0,98338	-7,50561
Glutaminas	0,001874	0,32211	0,02525	0,980277	0,9848467	-7,50595
CtIP	0,001697	-0,049514	0,032963	0,974253	0,98338	-7,5057
c-Abl_pY41	0,001207	0,027149	0,015088	0,988214	0,9905119	-7,50617
PEA-15	-9,00E-04	0,046253	-0,01204	0,990591	0,9905914	-7,50621
TUFM	-0,00207	0,039828	-0,02577	0,979869	0,9848467	-7,50593
MMP14	-0,00221	-0,048973	-0,03419	0,973293	0,98338	-7,50566
Bad_pS112	-0,00291	0,10143	-0,06122	0,952205	0,9797651	-7,50427
Bim_1	-0,00343	-0,20752	-0,04185	0,967317	0,983287	-7,50535
p21	-0,00365	0,016374	-0,05213	0,959296	0,9815226	-7,50482
PEA-15_pS	-0,00498	-0,061818	-0,05254	0,958974	0,9815226	-7,5048
MMP2	-0,00527	-0,02074	-0,09262	0,927756	0,9677306	-7,50166
MTSS1	-0,0055	0,031705	-0,08813	0,931247	0,9677306	-7,50209
GCN5L2	-0,00582	-0,152105	-0,11148	0,913107	0,9634726	-7,49958
Notch1	-0,006	-0,054826	-0,09932	0,922543	0,9664673	-7,50096
CD134	-0,00669	-5,21E-04	-0,10006	0,921969	0,9664673	-7,50088
DAPK2	-0,00723	0,006769	-0,14434	0,887665	0,9446507	-7,49504
Atg3	-0,00855	0,143336	-0,18255	0,858238	0,9390078	-7,4883
VAV1	-0,00942	-0,053778	-0,08274	0,935443	0,9691734	-7,50259
TRIP13	-0,01005	-0,047949	-0,15009	0,883224	0,9446507	-7,49413
UVRAG	-0,01046	0,098398	-0,16605	0,870919	0,9407674	-7,4914
TAZ	-0,01086	0,101754	-0,20688	0,83962	0,9334929	-7,48319
Connexin-4	-0,01109	-0,052007	-0,20308	0,842526	0,9334929	-7,48403
Chk2_pT68	-0,01126	-0,002757	-0,17091	0,86718	0,9407674	-7,49052
PCNA	-0,01142	-0,015273	-0,22479	0,825977	0,9334929	-7,47903
HSP60	-0,01235	-0,120894	-0,1948	0,848858	0,9380966	-7,48581
14-3-3-eps	-0,01304	0,009189	-0,20904	0,83797	0,9334929	-7,48271
PLK1	-0,01322	-0,086862	-0,10995	0,914293	0,9634726	-7,49976
cdc2_pY15	-0,01332	-0,23913	-0,18235	0,858397	0,9390078	-7,48834
TTF1	-0,01375	-0,024739	-0,21028	0,83703	0,9334929	-7,48243
LDHA	-0,01514	-0,094454	-0,15648	0,878295	0,9446507	-7,49307
PAI-1	-0,01557	-0,036353	-0,24027	0,814234	0,9334929	-7,47516
PI3K-p85	-0,01773	0,139813	-0,29183	0,775467	0,9216856	-7,46041
Smad1	-0,01782	0,042839	-0,29273	0,7748	0,9216856	-7,46013
MSH6	-0,018	-0,01407	-0,26778	0,793476	0,9236947	-7,46764
LAD1	-0,01827	-0,060637	-0,28425	0,781131	0,9216856	-7,46276
ENY2	-0,01914	-0,11689	-0,31929	0,755076	0,9216856	-7,45141
Rheb	-0,0195	-0,006062	-0,36834	0,719122	0,9115925	-7,43336
E2F1	-0,02116	-0,015219	-0,3631	0,722936	0,9137405	-7,43541
Axl	-0,02181	-0,091902	-0,20745	0,839182	0,9334929	-7,48307
Smac	-0,02201	-0,021718	-0,26562	0,795106	0,9236947	-7,46826

ERCC1	-0,03329	-0,05099	-0,60719	0,555201	0,6906695	-7,72549
MSH6	-0,03372	-0,01407	-0,5017	0,62508	0,7364894	-7,78722
XPF	-0,03464	0,014147	-0,65202	0,52685	0,6659012	-7,69591
PEA-15	-0,03548	0,046253	-0,47475	0,643608	0,7533757	-7,80119
MEK1	-0,03586	-0,124464	-0,50914	0,620013	0,7361582	-7,78323
A-Raf_pS299	-0,03724	0,023098	-0,56041	0,585652	0,711031	-7,75424
TRIM25	-0,03884	-0,075383	-0,29147	0,775736	0,8550956	-7,87626
eIF4E	-0,03966	0,084361	-0,61738	0,548679	0,6870819	-7,71894
4E-BP1_pS65	-0,0405	0,087254	-0,85972	0,407028	0,5516632	-7,53385
NRF2	-0,04068	-0,109994	-0,50021	0,626098	0,7364894	-7,78801
PDHA1	-0,04123	-0,038326	-0,58192	0,571541	0,7018063	-7,74129
FAK	-0,046	-0,154643	-0,60585	0,556061	0,6906695	-7,72635
Atg3	-0,04635	0,143336	-0,98955	0,3422	0,4821139	-7,41263
Chk2	-0,04795	0,017318	-0,40719	0,691138	0,7922348	-7,83293
G6PD	-0,0489	-0,105384	-0,50634	0,62192	0,7363942	-7,78474
Bax	-0,04902	-0,033866	-0,94164	0,365208	0,5077567	-7,45908
VEGFR-2	-0,05228	0,135944	-0,51965	0,612894	0,729716	-7,77751
CD86	-0,05365	-0,059701	-0,93629	0,367839	0,5081363	-7,46413
UVRAG	-0,05463	0,098398	-0,86756	0,402887	0,547774	-7,52695
C-Raf	-0,05464	-0,023119	-0,9846	0,344527	0,4821139	-7,41752
Gli3	-0,05536	2,76E-05	-0,89343	0,389435	0,5328465	-7,50379
Axl	-0,05542	-0,091902	-0,52721	0,607794	0,7256483	-7,77332
FoxO3a_pS318_S321	-0,05552	0,075277	-0,87614	0,398394	0,5433789	-7,51934
p27-Kip1	-0,05562	0,01314	-0,61814	0,548194	0,6870819	-7,71845
b-Catenin_pT41_S45	-0,05894	-0,033569	-1,02025	0,328017	0,4712516	-7,38185
FN14	-0,06271	0,021789	-0,83517	0,420168	0,5623988	-7,55509
Smac	-0,06479	-0,021718	-0,78191	0,449629	0,5890278	-7,59928
PKC-delta_pS664	-0,06491	-0,045451	-1,1406	0,276599	0,4182959	-7,25371
MTSS1	-0,06642	0,031705	-1,06452	0,308337	0,4488914	-7,33608
RRM1	-0,06676	-0,036564	-1,2192	0,246514	0,3849555	-7,16385
PEA-15_pS116	-0,06865	-0,061818	-0,72444	0,482873	0,6246131	-7,64401
oct-04	-0,06945	-0,096692	-0,24856	0,807962	0,8727614	-7,88873
HER2	-0,07422	-0,099699	-0,52788	0,607338	0,7256483	-7,77294
PI3K-p85	-0,07433	0,139813	-1,22322	0,245051	0,3849555	-7,15914
TTF1	-0,07948	-0,024739	-1,21583	0,247749	0,3854873	-7,1678
PLK1	-0,07955	-0,086862	-0,66161	0,520897	0,6603134	-7,68933
Rictor_pT1135	-0,08016	0,247853	-1,47281	0,166898	0,2900529	-6,84359
Chk1_pS345	-0,08126	0,094896	-1,22724	0,24359	0,3845689	-7,1544
TIGAR	-0,08135	-0,036293	-1,24129	0,238546	0,3799484	-7,13777
GSK-3a-b_pS21_S9	-0,08229	0,350958	-1,03118	0,323074	0,4657027	-7,3707
PDK1_pS241	-0,08381	0,104414	-1,52827	0,152728	0,2742739	-6,76789
LAD1	-0,08522	-0,060637	-1,32578	0,209938	0,3481555	-7,03468
GCLC	-0,0873	-0,02283	-1,21022	0,249816	0,3873051	-7,17436
Smad3	-0,08751	-0,00225	-1,26743	0,229378	0,3702701	-7,10643
PHGDH	-0,08832	-0,245847	-0,68661	0,505559	0,6465758	-7,67175
CD171	-0,09058	-0,179758	-0,41079	0,688567	0,7913926	-7,83136
PCNA	-0,09148	-0,015273	-1,80117	0,097195	0,1970097	-6,3697
Elk1_pS383	-0,09276	0,002093	-1,08983	0,297484	0,4406034	-7,30918
UBQLN4	-0,09283	-0,037809	-1,09481	0,295384	0,4390016	-7,30383
B-Raf_pS445	-0,09538	0,108475	-2,15781	0,052225	0,1232036	-5,79631
TSC1	-0,09703	0,030918	-1,76109	0,104017	0,206595	-6,43065
Tuberin_pT1462	-0,1018	0,059566	-1,29542	0,21988	0,3583302	-7,07232
c-Abl_pY412	-0,10324	0,027149	-1,29055	0,221507	0,3589076	-7,07828
Lasu1	-0,10349	-0,040635	-1,97293	0,072326	0,1558632	-6,10004
Rab25	-0,10376	0,029024	-1,16243	0,267973	0,4095612	-7,22923
BMK1-Erk5_pT218_Y220	-0,10552	0,018014	-1,80016	0,097362	0,1970097	-6,37124
B-Raf	-0,10806	0,001943	-1,25705	0,232982	0,3746839	-7,11892
ATRX	-0,10841	0,050761	-0,98604	0,34385	0,4821139	-7,41611
Cyclin-E1	-0,10864	0,131132	-1,24274	0,238029	0,3799484	-7,13604
PRAS40_pT246	-0,10991	0,125751	-2,13437	0,054446	0,1273271	-5,8355
MR1	-0,11097	0,031104	-0,82882	0,423612	0,5639149	-7,5605
CSK	-0,11147	0,00617	-1,82065	0,094028	0,1929812	-6,33979
Myosin-IIa_pS1943	-0,11326	-0,007141	-0,96548	0,353625	0,4932439	-7,43621
DRP1	-0,11511	0,073839	-2,38655	0,034619	0,0904295	-5,40478
LDHA	-0,11512	-0,094454	-1,18991	0,257411	0,3948196	-7,19789
B7-H3	-0,11647	-0,017587	-1,66719	0,121704	0,2280618	-6,57025
DJ1	-0,11723	-0,237303	-1,20642	0,251223	0,38809	-7,17878
BCL2A1	-0,11938	-0,035601	-1,24029	0,2389	0,3799484	-7,13895
Pdcd4	-0,12498	0,463311	-1,2941	0,220319	0,3583302	-7,07393
ULK1_pS757	-0,12592	0,319938	-2,34976	0,037004	0,0942433	-5,46876
Hexokinase-II	-0,1285	-0,321777	-1,00113	0,3368	0,4775027	-7,40112
PMS2	-0,1285	-0,057549	-1,52496	0,153543	0,2745932	-6,77246
eEF2	-0,12857	0,086276	-1,98123	0,071288	0,1543987	-6,0867
p44-42-MAPK	-0,12865	-0,015364	-1,11057	0,288815	0,4311983	-7,28678
PKCa	-0,12908	0,324233	-2,46911	0,029794	0,0805153	-5,25997
MERIT40	-0,12921	-0,004042	-1,35436	0,200922	0,3382707	-6,99868
PKM2	-0,1303	-0,316375	-2,0262	0,065897	0,1479243	-6,01385

VEGF-164	-0,0225	0,03077	-0,45574	0,656828	0,8984516	-7,39498
PKC-delta_	-0,0231	-0,045451	-0,40587	0,692084	0,9060647	-7,41785
CD38	-0,02375	-0,02424	-0,29152	0,775699	0,9216856	-7,46051
Elk1_pS383	-0,02471	0,002093	-0,29035	0,776576	0,9216856	-7,46088
Myt1	-0,02563	-0,055936	-0,42693	0,677093	0,8993053	-7,4085
YB1_pS102	-0,02588	-0,035683	-0,44413	0,664964	0,8984516	-7,40053
ARID1A	-0,02634	-0,041475	-0,23253	0,820103	0,9334929	-7,47713
Glutamate-	-0,02647	0,097722	-0,60081	0,559302	0,8406864	-7,31406
IGFRb	-0,02756	0,161739	-0,38599	0,706352	0,9060647	-7,42624
CD20	-0,02824	-0,016963	-0,39313	0,701211	0,9060647	-7,42327
CD31	-0,02923	0,01401	-0,60879	0,554171	0,8406864	-7,30899
AMPK-a2_	-0,02925	-0,111954	-0,25807	0,80079	0,9277965	-7,47039
Stat3	-0,0301	0,455546	-0,26584	0,79494	0,9236947	-7,4682
A-Raf	-0,03046	-0,001133	-0,48284	0,63802	0,8984516	-7,38148
PI3K-p110-	-0,03069	-0,043716	-0,48218	0,638473	0,8984516	-7,38181
XPA	-0,03157	0,037377	-0,51895	0,613365	0,8841479	-7,36233
FASN	-0,03189	-0,105082	-0,52224	0,611144	0,8839029	-7,36052
c-Kit	-0,0321	-0,017767	-0,45476	0,65751	0,8984516	-7,39545
DNMT1	-0,03266	-0,244014	-0,50902	0,620094	0,8900047	-7,36772
FGF-basic	-0,03273	0,070481	-0,46194	0,652506	0,8984516	-7,39196
PDK1_pS24	-0,03406	0,104414	-0,62099	0,546383	0,8406864	-7,30114
b-Catenin_	-0,03481	-0,033569	-0,60254	0,558185	0,8406864	-7,31296
Creb	-0,03563	-0,033283	-0,61429	0,550651	0,8406864	-7,30547
Smad4	-0,03589	-0,014953	-0,6382	0,535498	0,8406864	-7,2898
Atg7	-0,03594	-0,03646	-0,41261	0,68727	0,9058509	-7,41491
Puma	-0,03601	-0,083658	-0,44623	0,663488	0,8984516	-7,39954
SFRP1	-0,03692	0,054441	-0,7004	0,497222	0,840403	-7,24644
TRIM25	-0,03733	-0,075383	-0,28018	0,784178	0,9216856	-7,46399
PHGDH	-0,03738	-0,245847	-0,2906	0,776386	0,9216856	-7,4608
XIAP	-0,0377	0,151229	-0,57579	0,575542	0,8467108	-7,32952
PKC-a-b-II_	-0,03815	0,193816	-0,63003	0,54065	0,8406864	-7,29522
RRM2	-0,03856	0,068136	-0,42793	0,676386	0,8993053	-7,40805
14-3-3-zeta	-0,03932	-0,152677	-0,59116	0,565537	0,8406864	-7,3201
Cyclin-D1	-0,03934	0,034803	-0,48836	0,63422	0,8984516	-7,37864
Melanoma	-0,04041	-0,02963	-0,61095	0,552792	0,8406864	-7,30762
Jak2	-0,04052	0,117213	-0,46591	0,64974	0,8984516	-7,39
Smad3	-0,04057	-0,00225	-0,58763	0,567826	0,8410075	-7,32228
Rad51	-0,04059	0,041121	-0,38651	0,705979	0,9060647	-7,42603
PDHA1	-0,04079	-0,038326	-0,5757	0,575606	0,8467108	-7,32958
ATR	-0,04326	-0,111535	-0,42547	0,67813	0,8993053	-7,40916
Patched	-0,04336	-0,044418	-0,66118	0,521163	0,8406864	-7,27421
DDB-1	-0,04551	-0,14144	-0,5306	0,60551	0,8787029	-7,35587
HLA-DQA1	-0,04576	-0,058912	-0,44999	0,660853	0,8984516	-7,39775
DJ1	-0,04596	-0,237303	-0,47295	0,644852	0,8984516	-7,38649
CD45	-0,04603	-0,03593	-0,55679	0,588051	0,8620749	-7,34085
TIGAR	-0,04622	-0,036293	-0,70536	0,49424	0,838916	-7,24282
CIITA	-0,04667	-0,028549	-0,72799	0,480781	0,838916	-7,22602
WIPI1	-0,04676	0,170801	-0,81812	0,429454	0,7896418	-7,15431
NQO1	-0,04801	-0,013101	-0,38672	0,705829	0,9060647	-7,42595
MSI2	-0,0481	-0,049706	-0,64548	0,530934	0,8406864	-7,28492
HER2	-0,04935	-0,099699	-0,351	0,731758	0,9194979	-7,44003
HMHA1	-0,04968	-0,095438	-0,62103	0,54636	0,8406864	-7,30111
MSH2	-0,05021	-0,305486	-1,08214	0,30075	0,6647354	-6,90266
AMPKa_pT	-0,05151	0,069402	-0,88103	0,395845	0,7549078	-7,09986
p38-a	-0,05154	-0,037713	-0,99185	0,341123	0,7012504	-6,99544
RIP	-0,0529	0,030057	-0,773	0,45469	0,8097984	-7,19116
ATM_pS19	-0,05387	-0,044366	-0,65984	0,521991	0,8406864	-7,27514
p44-42-MA	-0,05544	-0,015364	-0,47859	0,640954	0,8984516	-7,38365
CD26	-0,05677	-0,017648	-1,023	0,326769	0,6836772	-6,96419
Pdcd4	-0,05707	0,463311	-0,59097	0,565659	0,8406864	-7,32021
C-Raf	-0,05729	-0,023119	-1,03231	0,322566	0,6781759	-6,9547
EGFR_pY11	-0,05841	0,002371	-0,86987	0,401674	0,7595346	-7,10978
p27_pT157	-0,05885	0,027337	-1,11707	0,286136	0,6492664	-6,865
UBAC1	-0,06053	-0,032503	-1,06014	0,310244	0,6761695	-6,92589
BMK1-Erk5	-0,06084	0,018014	-1,03786	0,320079	0,676246	-6,949
DUSP6	-0,06199	0,129979	-1,041	0,31868	0,676246	-6,94577
NDUFB4	-0,06276	-0,047676	-0,72411	0,483069	0,838916	-7,22893
Beclin	-0,0634	0,065552	-1,3817	0,192594	0,5355361	-6,54963
MR1	-0,06499	0,031104	-0,48545	0,636224	0,8984516	-7,38014
TFRC	-0,06518	-0,023369	-0,68724	0,505179	0,8406864	-7,25592
eIF4E_pS2(	-0,06587	0,048507	-1,41837	0,18188	0,5226028	-6,50203
FAK	-0,06604	-0,154643	-0,86964	0,401796	0,7595346	-7,10999
UGT1A	-0,067	0,268485	-0,9738	0,349646	0,7108366	-7,01317
MERIT40	-0,06756	-0,004042	-0,70813	0,492579	0,838916	-7,24079
Stat3_pY7(	-0,06769	0,23668	-0,75956	0,462383	0,8134175	-7,20176
AceCS1	-0,06829	0,175013	-0,76761	0,457764	0,8119186	-7,19543
Atg4B	-0,06833	0,127254	-1,27786	0,225799	0,5561111	-6,67945

JAB1	-0,13096	0,007015	-1,74349	0,107147	0,20991	-6,45717
Melanoma-gp100	-0,13262	-0,02963	-2,00519	0,068366	0,1517183	-6,04798
UBAC1	-0,13402	-0,032503	-2,34725	0,037173	0,0942433	-5,47311
IGFRb	-0,13476	0,161739	-1,88747	0,083862	0,1746119	-6,23586
AMPKa	-0,13744	0,044799	-2,49132	0,028612	0,0790486	-5,22074
FGF-basic	-0,1388	0,070481	-1,95918	0,074079	0,1583829	-6,12212
Bcl-xL	-0,1428	-0,052409	-3,42397	0,005139	0,0238166	-3,51251
mTOR_pS2448	-0,14429	0,087497	-1,99768	0,06927	0,1521588	-6,06015
EGFR_pY1173	-0,14614	0,002371	-2,17636	0,050528	0,1209862	-5,76516
NDRG1_pT346	-0,14764	0,175484	-1,68748	0,117672	0,223421	-6,54049
Atg7	-0,14892	-0,03646	-1,70957	0,113414	0,2162903	-6,50781
IR-b	-0,14912	-0,023481	-1,3656	0,197461	0,3337486	-6,98436
eIF4G	-0,15161	0,075302	-2,36542	0,03597	0,0922811	-5,44157
ACSL1	-0,15219	0,059466	-2,85931	0,014551	0,0486177	-4,55744
PAK4	-0,15378	0,010678	-3,08881	0,009525	0,0363294	-4,13492
GSK-3a-b	-0,15507	0,145096	-2,83211	0,015301	0,0503401	-4,60717
HLA-DQA1	-0,15593	-0,058912	-1,53343	0,151463	0,2731411	-6,76075
STING	-0,15757	0,244125	-0,69858	0,498317	0,640593	-7,66313
E-Cadherin	-0,15879	-0,718665	-0,35979	0,725345	0,8206834	-7,85238
p27_pT198	-0,1621	-0,028398	-1,80739	0,096174	0,1964504	-6,36017
c-IAP2	-0,16608	-0,079066	-2,51557	0,027372	0,0776151	-5,17778
b-Catenin	-0,16648	-0,058081	-2,96857	0,011893	0,0423626	-4,35686
PI3K-p110-a	-0,1675	0,200034	-2,40674	0,033374	0,0886222	-5,36952
PTPN12	-0,16914	0,448663	-2,9857	0,011523	0,0417335	-4,32532
HMHA1	-0,17056	-0,095438	-2,13223	0,054653	0,1273271	-5,83907
53BP1	-0,17105	0,410082	-1,95516	0,074598	0,1583829	-6,12855
ARID1A	-0,1716	-0,041475	-1,51473	0,15609	0,2755208	-6,78655
WIPI1	-0,17492	0,170801	-3,0604	0,010038	0,0375981	-4,18746
Atg5	-0,17764	0,237612	-2,24504	0,044687	0,1100586	-5,64884
Mitofusin-2	-0,17782	0,07772	-1,8598	0,087944	0,18223	-6,27915
Atg4B	-0,18092	0,127254	-3,38343	0,005536	0,0251152	-3,58793
CD26	-0,18096	-0,017648	-3,26078	0,006936	0,0305055	-3,81598
IDO	-0,18098	-0,045778	-1,56092	0,144877	0,2623608	-6,72244
Raptor	-0,18612	0,029375	-2,88077	0,013986	0,0478425	-4,51815
Paxillin	-0,18614	-0,099849	-2,60589	0,023201	0,0694306	-5,01673
Merlin	-0,18912	-0,057375	-2,20243	0,04823	0,1169655	-5,72118
Gys	-0,19124	0,329948	-2,46735	0,02989	0,0805153	-5,26306
Erk5	-0,19318	-0,046955	-2,55467	0,025484	0,0732234	-5,10826
PYGB	-0,19334	0,004676	-3,3156	0,006271	0,0281526	-3,71409
RSK	-0,19347	-0,552241	-3,24239	0,007175	0,0307184	-3,85016
Slfn11	-0,1971	-0,015227	-1,34841	0,202773	0,3400593	-7,00623
A-Raf	-0,20048	-0,001133	-3,17752	0,008087	0,0328805	-3,97057
SHP2	-0,2016	-0,044912	-2,76312	0,017376	0,0548696	-4,73287
HER3_pY1289	-0,20666	3,22E-04	-3,93653	0,002028	0,0107897	-2,56198
HSP27_pS82	-0,21049	-0,021664	-1,51486	0,156057	0,2755208	-6,78637
B7-H4	-0,21369	-0,039993	-2,49705	0,028314	0,0787308	-5,21059
4E-BP1_pT37_T46	-0,2144	-0,149083	-2,75225	0,017727	0,0553663	-4,75262
NF-kB-p65_pS536	-0,21507	0,118054	-4,07391	0,001587	0,0089997	-2,30944
GSK-3B	-0,21579	0,32798	-2,09856	0,058013	0,1329982	-5,89503
Aurora-ABC_pT288_pT23	-0,21695	0,130098	-2,72428	0,018665	0,0575552	-4,80337
IRS1	-0,21722	0,130579	-2,8659	0,014376	0,0484051	-4,54537
MSH2	-0,21838	-0,305486	-4,70709	5,28E-04	0,0034993	-1,16725
SLC1A5	-0,21948	-0,020076	-1,67974	0,119195	0,2253199	-6,55186
ACC1	-0,21957	-0,14479	-4,01458	0,001764	0,0097457	-2,41834
Tuberin	-0,22095	0,001667	-2,78026	0,016836	0,054558	-4,7017
ER-a	-0,22121	-0,077341	-2,58447	0,024131	0,0708299	-5,05508
DDR1	-0,22555	-0,018116	-3,87368	0,00227	0,0116171	-2,67794
Gys_pS641	-0,23075	0,273752	-2,80554	0,016069	0,052468	-4,65566
Rb_pS807_S811	-0,23533	0,274772	-4,59662	6,37E-04	0,0040988	-1,36353
IGF1R_pY1135_Y1136	-0,23661	-0,141052	-3,83021	0,002455	0,0123014	-2,75826
EMA	-0,24261	0,046986	-1,11883	0,285412	0,430114	-7,27775
ATR	-0,24415	-0,111535	-2,40103	0,033722	0,0886222	-5,3795
Gab2	-0,24626	0,045631	-2,8928	0,013679	0,047166	-4,4961
Akt_pT308	-0,25003	0,127704	-3,61772	0,003607	0,0168977	-3,15222
PARP	-0,25049	-0,075124	-4,78407	4,63E-04	0,0031692	-1,0313
ASNS	-0,25176	-0,022748	-3,78878	0,002645	0,0131036	-2,83492
Akt_pS473	-0,25672	0,399518	-2,57498	0,024554	0,0710245	-5,07203
ZAP-70	-0,26283	-0,001713	-2,37703	0,035222	0,0914502	-5,42138
MCT4	-0,27109	0,021705	-4,12357	0,001453	0,0083507	-2,21851
DDB-1	-0,28114	-0,14144	-3,27753	0,006726	0,0298838	-3,78485
Akt2	-0,28563	0,225659	-4,60106	6,32E-04	0,0040988	-1,35562
XIAP	-0,292	0,151229	-4,4592	8,07E-04	0,0049705	-1,6096
PDK1	-0,29333	-0,003713	-3,23528	0,00727	0,0307184	-3,86335
Smad1	-0,29718	0,042839	-4,88157	3,93E-04	0,0028245	-0,86011
Cox2	-0,29779	0,165493	-1,75637	0,104848	0,2072541	-6,43778
Cyclin-D3	-0,30003	0,043783	-4,04432	0,001673	0,0093629	-2,36373
Rad23A	-0,30217	-0,014213	-1,71281	0,112802	0,2160781	-6,503

Paxillin	-0,07021	-0,099849	-0,9829	0,345331	0,7053908	-7,00427
JAB1	-0,07169	0,007015	-0,95434	0,359005	0,7221004	-7,03197
MAPK_pT2	-0,07337	0,259927	-0,67024	0,515574	0,8406864	-7,26793
Tuberin	-0,07413	0,001667	-0,93279	0,369572	0,7354429	-7,05241
ACC1	-0,07419	-0,14479	-1,35655	0,200244	0,5484457	-6,58176
B7-H3	-0,07435	-0,017587	-1,06426	0,308448	0,6761695	-6,92157
p27_pT198	-0,07463	-0,028398	-0,8321	0,421831	0,7836602	-7,14253
CSK	-0,07506	0,00617	-1,22592	0,24407	0,5811843	-6,74151
UBQLN4	-0,07516	-0,037809	-0,8865	0,393009	0,7549078	-7,09496
p38-MAPK	-0,07553	0,104174	-1,27125	0,228063	0,5584958	-6,68746
eIF4G	-0,07654	0,075302	-1,19416	0,255807	0,6057853	-6,77848
PAK1	-0,07698	0,121987	-1,33781	0,206103	0,5484457	-6,60541
Rab25	-0,0791	0,029024	-0,88615	0,393191	0,7549078	-7,09527
PI3K-p110-	-0,07947	0,200034	-1,14186	0,276096	0,6397701	-6,83768
CDK1_pT14	-0,08039	-0,263332	-1,51417	0,156228	0,502496	-6,37358
b-Catenin	-0,0806	-0,058081	-1,43733	0,176541	0,5186339	-6,47708
Gli1	-0,08148	0,045725	-1,43177	0,178092	0,5186339	-6,48442
DDR1	-0,08456	-0,018116	-1,45234	0,172404	0,5186339	-6,45715
LC3A-B	-0,08521	-0,040287	-1,64471	0,126315	0,4628801	-6,18961
Enolase-2	-0,0863	-7,87E-04	-1,30098	0,218032	0,553027	-6,6512
p38-MAPK-	-0,08633	0,014931	-1,43997	0,175807	0,5186339	-6,47358
Porin	-0,08667	-0,05308	-1,85912	0,088046	0,3783138	-5,86753
TSC1	-0,09035	0,030918	-1,63988	0,127326	0,4628801	-6,1966
eEF2	-0,09043	0,086276	-1,39348	0,189097	0,535152	-6,53444
MEK2	-0,09143	-0,028089	-1,79209	0,098705	0,4013369	-5,97068
PMS2	-0,09192	-0,057549	-1,09079	0,29708	0,6600066	-6,89343
Gli3	-0,09223	2,76E-05	-1,48857	0,162762	0,509804	-6,40847
PAICS	-0,09402	-0,15572	-1,55957	0,145196	0,4944847	-6,31074
HSP27_pS8	-0,09473	-0,021664	-0,68177	0,508509	0,8406864	-7,25982
Wee1	-0,09497	-0,01496	-0,7051	0,494396	0,838916	-7,24301
NDRG1_pT	-0,09594	0,175484	-1,09657	0,294646	0,6600066	-6,88722
Lasu1	-0,09597	-0,040635	-1,82963	0,0926	0,3801027	-5,91318
PYGB	-0,09771	0,004676	-1,67562	0,120014	0,4616099	-6,14464
Bax	-0,09977	-0,033866	-1,91636	0,079786	0,362617	-5,77782
oct-04	-0,09997	-0,096692	-0,35778	0,726812	0,9159527	-7,43746
Granzyme-	-0,10128	0,194614	-1,17624	0,262624	0,615167	-6,799
AMPKa	-0,10175	0,044799	-1,84434	0,090303	0,3783138	-5,89046
SHP2	-0,10227	-0,044912	-1,40168	0,186694	0,5328811	-6,52381
IRS2	-0,10313	0,497198	-1,09538	0,295144	0,6600066	-6,8885
ASNS	-0,10411	-0,022748	-1,56679	0,143505	0,4944847	-6,30062
Rictor_pT1	-0,10416	0,247853	-1,91373	0,08015	0,362617	-5,78198
FoxO3a_pS	-0,10503	0,075277	-1,65761	0,12365	0,4628801	-6,17091
BCL2A1	-0,10513	-0,035601	-1,09228	0,296452	0,6600066	-6,89183
VEGFR-2	-0,10621	0,135944	-1,05567	0,312199	0,6761695	-6,93056
XPF	-0,10753	0,014147	-2,02401	0,06615	0,3277096	-5,60532
Gys	-0,10778	0,329948	-1,39052	0,189973	0,535152	-6,53827
Chk1_pS34	-0,1098	0,094896	-1,65826	0,123518	0,4628801	-6,16997
DRP1	-0,11062	0,073839	-2,29345	0,040963	0,2635093	-5,15494
PKA-a	-0,11139	-0,147499	-1,34574	0,203606	0,5484457	-6,59543
Myosin-IIa_	-0,11771	-0,007141	-1,00337	0,335763	0,6957393	-6,98398
GSK-3a-b	-0,11995	0,145096	-2,19071	0,04925	0,2756727	-5,32951
ERCC1	-0,12051	-0,05099	-2,19778	0,048632	0,2756727	-5,3176
DVL3	-0,12073	0,097953	-2,36785	0,035812	0,2450007	-5,02661
Atg5	-0,12288	0,237612	-1,55304	0,146739	0,4944847	-6,31984
Akt2	-0,12584	0,225659	-2,02706	0,065797	0,3277096	-5,60036
p90RSK_pT	-0,12803	0,127706	-0,7199	0,485567	0,838916	-7,23208
B-Raf_pS44	-0,12818	0,108475	-2,89989	0,013501	0,1452348	-4,07489
MCT4	-0,12951	0,021705	-1,97009	0,072686	0,3442579	-5,69232
Rb_pS807_	-0,13049	0,274772	-2,54875	0,025761	0,1982695	-4,70887
B-Raf	-0,13096	0,001943	-1,52347	0,153913	0,502496	-6,36081
PKM2	-0,1319	-0,316375	-2,05104	0,063084	0,3251399	-5,56129
Raptor	-0,13285	0,029375	-2,05618	0,062517	0,3251399	-5,55289
ACVRL1	-0,133	-2,20E-04	-2,50766	0,027771	0,2099881	-4,7817
PKCa	-0,13419	0,324233	-2,56682	0,024923	0,1982695	-4,67674
RSK	-0,13516	-0,552241	-2,26519	0,0431	0,2645265	-5,20328
ZAP-70	-0,136	-0,001713	-1,22999	0,242596	0,5808818	-6,73672
ACSL1	-0,13646	0,059466	-2,56373	0,025065	0,1982695	-4,68226
p70-S6K1	-0,13687	0,134162	-1,90928	0,080769	0,362617	-5,789
MDM2_pS	-0,13705	0,002366	-1,85463	0,088727	0,3783138	-5,87452
B7-H4	-0,13796	-0,039993	-1,61214	0,133268	0,4786537	-6,23644
ER-a	-0,14017	-0,077341	-1,63761	0,127802	0,4628801	-6,19987
GSK-3a-b_	-0,14136	0,350958	-1,77147	0,10221	0,4078921	-6,00197
HER3_pY12	-0,14265	3,22E-04	-2,71721	0,018909	0,1733991	-4,40703
NF-kB-p65_	-0,14574	0,118054	-2,76059	0,017457	0,1656751	-4,32855
RPA32_pS4	-0,14623	0,003	-2,2038	0,048112	0,2756727	-5,30745
Mitofusin-2	-0,14717	0,07772	-1,53926	0,150045	0,5013137	-6,339
c-IAP2	-0,14783	-0,079066	-2,23909	0,045167	0,2666706	-5,24771

PAICS	-0,30731	-0,15572	-5,0976	2,75E-04	0,0021143	-0,48504
DVL3	-0,31665	0,097953	-6,21043	4,80E-05	5,31E-04	1,34919
mTOR	-0,31753	0,02475	-5,68632	1,07E-04	0,0010014	0,506244
Akt1	-0,32109	-0,036534	-1,77286	0,101971	0,2044161	-6,41284
Stat3_pY705	-0,3245	0,23668	-3,64101	0,003457	0,0163746	-3,10895
ERRalpha	-0,32454	-0,060608	-5,79938	8,97E-05	8,78E-04	0,691262
eEF2K	-0,33215	0,045657	-3,22723	0,007378	0,0308748	-3,87829
MLKL	-0,33376	-0,032043	-2,70875	0,019206	0,0587069	-4,83147
Wee1	-0,33378	-0,01496	-2,47799	0,029316	0,0801925	-5,24429
MITF	-0,33833	0,104632	-6,35878	3,86E-05	4,86E-04	1,580981
RPA32	-0,34532	-0,242082	-5,47934	1,48E-04	0,0012646	0,163012
Jak2	-0,34611	0,117213	-3,97961	0,001877	0,0102422	-2,48265
VAV1	-0,35286	-0,053778	-3,09766	0,00937	0,0363294	-4,11854
p38-MAPK	-0,35463	0,104174	-5,96883	6,91E-05	7,09E-04	0,965284
PTEN	-0,35736	0,327711	-5,061	2,92E-04	0,0021683	-0,54816
GCN5L2	-0,37165	-0,152105	-7,11823	1,31E-05	2,10E-04	2,721295
Tyro3	-0,38247	0,007668	-6,64415	2,55E-05	3,55E-04	2,018466
p70-S6K1	-0,39486	0,134162	-5,50817	1,41E-04	0,0012445	0,211165
Src_pY527	-0,39616	0,028145	-5,38593	1,72E-04	0,0014253	0,006224
Rictor	-0,39956	0,284828	-6,17974	5,03E-05	5,42E-04	1,300876
Heregulin	-0,4105	0,529459	-5,11474	2,67E-04	0,002093	-0,45554
PLC-gamma2_pY759	-0,42675	-0,048337	-4,85309	4,12E-04	0,0029141	-0,91
Akt	-0,4348	-0,027398	-6,82411	1,98E-05	2,88E-04	2,288736
Akt2_pS474	-0,44435	0,456442	-5,5679	1,29E-04	0,0011693	0,310577
Mcl-1	-0,44681	0,126503	-5,88719	7,83E-05	7,85E-04	0,833756
PKA-a	-0,45301	-0,147499	-5,47293	1,50E-04	0,0012646	0,152283
PHLPP	-0,45585	0,138066	-7,96525	4,30E-06	8,25E-05	3,905764
Sox2	-0,46061	0,345513	-7,94658	4,40E-06	8,25E-05	3,880591
GAPDH	-0,46402	-0,093054	-6,51693	3,06E-05	4,13E-04	1,824783
Akt1_pS473	-0,47762	0,132057	-7,75171	5,65E-06	1,02E-04	3,615459
Stat5a	-0,50318	0,376954	-6,37435	3,77E-05	4,86E-04	1,605132
RRM2	-0,52111	0,068136	-5,78366	9,19E-05	8,80E-04	0,665629
b-Actin	-0,5242	-0,123301	-5,56055	1,30E-04	0,0011693	0,298364
Shc_pY317	-0,5344	0,048967	-6,23445	4,64E-05	5,26E-04	1,386934
Stat3	-0,54545	0,455546	-4,81689	4,38E-04	0,003047	-0,97355
JNK_pT183_Y185	-0,5871	0,06726	-6,10202	5,65E-05	5,94E-04	1,177915
FAK_pY397	-0,655	0,127547	-5,15556	2,50E-04	0,0019937	-0,38541
MMP14	-0,68302	-0,048973	-10,5751	2,21E-07	5,96E-06	7,057217
c-Met_pY1234_Y1235	-0,6895	-0,612021	-6,81436	2,00E-05	2,88E-04	2,274211
Src_pY416	-0,71654	0,071301	-6,95875	1,64E-05	2,52E-04	2,488164
HER2_pY1248	-0,72084	0,074269	-5,08225	2,82E-04	0,0021304	-0,5115
HES1	-0,72648	-0,136402	-2,76109	0,017441	0,0548696	-4,73656
FRS2-a_pY196	-0,79217	0,09159	-6,24453	4,57E-05	5,26E-04	1,402745
cdc2_pY15	-0,80946	-0,23913	-11,0781	1,34E-07	3,85E-06	7,591198
Coup-TFII	-0,84072	0,178384	-11,8459	6,44E-08	2,14E-06	8,367896
Chk1	-0,85974	-0,197309	-15,041	4,50E-09	2,16E-07	11,18095
DUSP6	-0,90276	0,129979	-15,1602	4,12E-09	2,16E-07	11,27484
SHP-2_pY542	-0,91189	0,322221	-7,1277	1,30E-05	2,10E-04	2,735042
Rad51	-0,92203	0,041121	-8,78008	1,59E-06	3,26E-05	4,965088
Connexin-43	-1,00379	-0,052007	-18,3876	4,58E-10	3,78E-08	13,57667
Pyk2_pY402	-1,02345	0,131592	-8,81671	1,52E-06	3,26E-05	5,010972
Hif-1-alpha	-1,04734	0,203091	-9,8255	4,87E-07	1,10E-05	6,220818
Snail	-1,38606	0,389254	-18,747	3,67E-10	3,78E-08	13,80784

PKC-b-II_pS	-0,14893	0,022917	-1,32892	0,208933	0,5484457	-6,61656
Rictor	-0,1544	0,284828	-2,38797	0,03453	0,2450007	-4,99167
EMA	-0,15474	0,046986	-0,71361	0,489307	0,838916	-7,23675
IDO	-0,15487	-0,045778	-1,33572	0,206767	0,5484457	-6,60804
ATM	-0,15562	0,084602	-1,48376	0,164015	0,509804	-6,41498
PHLPP	-0,15905	0,138066	-2,77914	0,01687	0,1656751	-4,29492
PAK4	-0,16293	0,010678	-3,27263	0,006787	0,0886378	-3,38785
Akt	-0,16642	-0,027398	-2,61191	0,022946	0,1939187	-4,5963
ATRX	-0,16836	0,050761	-1,53137	0,151966	0,502496	-6,34991
GCLC	-0,1712	-0,02283	-2,37336	0,035457	0,2450007	-5,01705
FOXO3	-0,1723	0,039835	-1,67056	0,121025	0,4616099	-6,15204
PLC-gamma	-0,17834	-0,048337	-2,02811	0,065676	0,3277096	-5,59866
Erk5	-0,18134	-0,046955	-2,39804	0,033905	0,2450007	-4,97413
VASP	-0,18221	0,087681	-2,68052	0,020229	0,1816439	-4,47319
Gab2	-0,18294	0,045631	-2,14896	0,053053	0,2931512	-5,3995
MLKL	-0,18411	-0,032043	-1,4942	0,161307	0,509804	-6,40084
53BP1	-0,18424	0,410082	-2,10585	0,057269	0,3105905	-5,47115
Akt1	-0,18804	-0,036534	-1,03822	0,319917	0,676246	-6,94863
GSK-3B	-0,18819	0,32798	-1,83018	0,092514	0,3801027	-5,91233
Akt1_pS47	-0,18971	0,132057	-3,07886	0,009701	0,1226175	-3,74609
p53	-0,1926	0,265969	-2,25128	0,04419	0,2645265	-5,22698
PRAS40_pT	-0,19377	0,125751	-3,76293	0,002772	0,0522848	-2,47979
FRS2-a_pY	-0,19741	0,09159	-1,55616	0,146001	0,4944847	-6,3155
p70-S6K_p	-0,19983	0,269091	-1,59917	0,136127	0,4848836	-6,25491
GAPDH	-0,20186	-0,093054	-2,83502	0,015218	0,1561705	-4,19331
PDK1	-0,20918	-0,003713	-2,3071	0,039968	0,2610029	-5,13151
PTPN12	-0,2106	0,448663	-3,71753	0,003009	0,0522848	-2,56363
4E-BP1_pT	-0,21502	-0,149083	-2,76025	0,017468	0,1656751	-4,32919
Slfn11	-0,21667	-0,015227	-1,48223	0,164415	0,509804	-6,41704
Akt2_pS47	-0,21976	0,456442	-2,75364	0,017682	0,1656751	-4,34116
JNK_pT183	-0,22435	0,06726	-2,33181	0,038225	0,2534579	-5,08896
Rad23A	-0,22733	-0,014213	-1,28859	0,222167	0,5534901	-6,66638
Src_pY416	-0,22829	0,071301	-2,21701	0,046989	0,2736773	-5,28513
Akt_pT308	-0,23395	0,127704	-3,38497	0,00552	0,0793074	-3,17963
FAK_pY397	-0,23423	0,127547	-1,84365	0,090409	0,3783138	-5,89152
Pyk2_pY40	-0,23779	0,131592	-2,04848	0,063368	0,3251399	-5,56547
Akt_pS473	-0,24707	0,399518	-2,47815	0,029308	0,2177851	-4,83377
Gys_pS641	-0,24799	0,273752	-3,0151	0,010914	0,1237839	-3,86353
ULK1_pS75	-0,25007	0,319938	-4,66656	5,65E-04	0,0203077	-0,84296
mTOR	-0,26135	0,02475	-4,68024	5,52E-04	0,0203077	-0,8188
Src_pY527	-0,26204	0,028145	-3,56249	0,003989	0,0614001	-2,85053
IGF1R_pY1	-0,26236	-0,141052	-4,24708	0,001169	0,0296286	-1,59372
c-Met_pY1	-0,26245	-0,612021	-2,59384	0,02372	0,1965997	-4,6286
PTEN	-0,26701	0,327711	-3,78148	0,00268	0,0522848	-2,44556
mTOR_pS2	-0,2714	0,087497	-3,75756	0,002799	0,0522848	-2,48969
WIPI2	-0,27549	-0,02897	-2,09508	0,058371	0,3105905	-5,48895
SHP-2_pY5	-0,29038	0,322221	-2,26975	0,042748	0,2645265	-5,19549
Shc_pY317	-0,29926	0,048967	-3,49128	0,004543	0,0675184	-2,9825
Stat5a	-0,33847	0,376954	-4,28781	0,001088	0,0293093	-1,52003
HER2_pY1	-0,42823	0,074269	-3,01925	0,01083	0,1237839	-3,8559
S6_pS240_	-0,43847	0,524502	-3,69037	0,003161	0,0523966	-2,61383
S6_pS235_	-0,47719	0,525909	-2,61345	0,022882	0,1939187	-4,59355
JNK2	-0,55213	0,368288	-7,08148	1,38E-05	8,51E-04	3,03862

Table S8: Cell cycle distribution of the *MMTV-R26^{Met}* MGT cell lines - Statistical analysis was performed by two-way ANOVA followed by Tukey test.

		MGT2					MGT4					MGT9					MGT11					MGT13				
		G0	G1	S	G2	M	G0	G1	S	G2	M	G0	G1	S	G2	M	G0	G1	S	G2	M	G0	G1	S	G2	M
**MGT7**	G0	***					***					***					***					***				
	G1		***					***					***					***					***			
	S			*					***					***					***					***		
	G2				ns					***					***					***					***	
	M					ns					ns					ns					ns					ns
**MGT2**	G0						***					***					***					***				
	G1							*					***					ns					ns			
	S								***					***					***					ns		
	G2									***					ns					***					***	
	M										ns					ns					ns					ns
**MGT4**	G0											ns					ns					*				
	G1												ns					ns					***			
	S													ns					*					ns		
	G2														*					ns					**	
	M															ns					ns					ns
**MGT9**	G0																ns					**				
	G1																	***					***			
	S																		ns					**		
	G2																			ns					***	
	M																				ns					ns
**MGT11**	G0																					***				
	G1																						*			
	S																							***		
	G2																								***	
	M																									ns
**MGT13**	G0																									
	G1																									
	S																									
	G2																									
	M																									

Table S9: In vitro tumorigenic capacity of the *MMTV-R26*^{Met} cell lines, as determined by the tumor sphere assay - Statistical analysis was performed by One-way ANOVA followed by Tukey test.

		Passage 1								Passage 3			
		MGT2		MGT4		MGT11		MGT13		MGT11		MGT13	
		50-100µm	>100 µm	50-100µm	>100 µm	50-100µm	>100 µm	50-100µm	>100 µm	50-100µm	>100 µm	50-100µm	>100 µm
**MGT7**	50-100µm	0.034 (*)		0.086 (ns)		0.005 (**)		0.003 (**)					
	>100 µm		0.116 (ns)		0.116 (ns)		<0.0001 (***)		0.007 (**)				
**MGT2**	50-100µm			0.113 (ns)		0.006 (**)		0.003 (**)					
	>100 µm				>0.999 (ns)		<0.0001 (***)		0.008 (**)				
**MGT4**	50-100µm					0.055 (ns)		0.008 (**)		0.798 (ns)		0.032 (*)	
	>100 µm						<0.0001 (***)		0.008 (**)		0.009 (**)		0.021 (*)
**MGT11**	50-100µm							0.039 (*)				0.019 (*)	
	>100 µm								0.036 (*)				0.198 (ns)

Table S10 : Migrating capacity of the *MMTV-R26*^{Met} cell lines - Statistical analysis was performed by One-way ANOVA followed by Tukey test.

	MGT7	MGT2	MGT4	MGT9	MGT11	MGT13
MGT7		0.2249 (ns)	0.0178 (*)	0.0002 (***)	<0.0001 (***)	<0.0001 (***)
MGT2			0.6052 (ns)	0.0139 (*)	0.0053 (**)	<0.0001 (***)
MGT4				0.4207 (ns)	0.2363 (ns)	<0.0001 (***)
MGT9					0.9987 (ns)	<0.0001 (***)
MGT11						0.0002 (***)
MGT13						

Table S11: Cell cycle distribution of MGT11 treated cells - Statistical analysis was performed by two-way ANOVA followed by Tukey test.

		A11					Adav					A11 + Adav				
		G0	G1	S	G2	M	G0	G1	S	G2	M	G0	G1	S	G2	M
**no**	G0	ns					ns					ns				
	G1		ns					ns					***			
	S			ns					ns					ns		
	G2				ns					***					***	
	M					ns					ns					ns
**A11**	G0						ns					ns				
	G1							***					*			
	S								ns					ns		
	G2									***					***	
	M										ns					ns
**Adav**	G0											ns				
	G1												ns			
	S													ns		
	G2														ns	
	M															ns
**A11 + Adav**	G0															
	G1															
	S															
	G2															
	M															

Table S12: Antibodies used in the study.

Antibody	Company	Reference	Dilution	Used for	TritonX-100 (%) for IF
Estrogen receptor alpha (ERα)	Santa Cruz	sc-8005	1:100	IHC	0.5 %
Progesteron receptor (PR)	Santa Cruz	sc-810	1:200	IHC	0.5 %
Human EGFR2 (HER2)	Cell Signaling	2165	1:200	IHC	0.2 %
MET	Santa Cruz	sc-10	1:50	IHC	0.2 %
Ki67	Cell Signaling	9129	1:400	IHC	0.5 %
Ki67	Dako	M7249	1:100	IF	0.1 %
Krt14	Covalab	Mab71720	1:500	IF	0.5 %
Krt18	Covalab	Mab20042	1:500	IF	0.5 %
human MET	Assay Design	905-076	1:150	IF	0.5 %
$pY_{1234/35}$MET	Cell Signaling	3126L	1:50	IF	0.5 %
pH3 (S10)	Millipore	06-570	1 :500	IF	0.3 %
alpha-Tubulin	Sigma	T5168	1 :5000	IF	0.3 %
Vimentin	Abcam	Ab-8979	1:100	IF	0.5 %
pS_{139}H2AX (γH2AX)	Cell Signaling	9718	1 : 400	IF, WB	0.2 %
Actin	Sigma	A3853	1:6000	WB	
ATM	Cell Signaling	2873	1:1000	WB	
pS_{1987}ATM	Invitrogen	PA5-37346	1:1000	WB	
ATR	Cell Signaling	13934	1:1000	WB	
pS_{428}ATR	Cell Signaling	2853	1:1000	WB	
pS_{473}AKT	Cell Signaling	9271	1:2000	WB	
BCL-XL	Transduction Laboratory	B22620	1:500	WB	
BIM	Santa Cruz	sc-11425	1:1000	WB	
Cleaved-Caspase 3	Cell Signaling	9661	1:1000	WB	
CDC2 (CDK1)	Cell Signaling	28493	1:20000	WB	
pY_{15}CDC2(CDK1)	Cell Signaling	4539	1:1000	WB	
pT_{202}/Y_{204}ERKs	Cell Signaling	9106	1:10000	WB	
pY_{627}GAB1	Cell Signaling	3231	1:2000	WB	
MCL1	Santa Cruz	sc-819	1:1000	WB	
$pS_{217/221}$MEK	Cell Signaling	9121	1:1000	WB	
mouse MET	Santa Cruz	sc-8057	1:500	WB	
MET^{25H2}	Cell Signaling	3127	1:1000	WB	
human MET	Santa Cruz	sc-161	1:1000	WB	

$pY_{1234/35}MET$	Cell Signaling	3126	1:2000	WB	
PARP	Cell Signaling	9546S	1:2000	WB	
P53	Novocastra	CM5	1:1000	WB	
$pS_{15}P53$	Cell Signaling	9284	1:1000	WB	
RB	Cell Signaling	9313	1:1000	WB	
$pS_{795}RB$	Abcam	Ab47474	1:1000	WB	
RPA32 (B-4)	Santa Cruz	sc-271578	1:200	WB	
$pS_{33}RPA32$	Bethyl	A300-246A	1 :5000	WB	
RRM2	abbexa	Abx004031	1 :5000	WB	
$pY_{705}STAT3$	Cell Signaling	9145S	1:2000	WB	
XIAP	Transduction laboratory	610716	1:3000	WB	
Goat anti-rabbit IgG-peroxidase	Jackson Immuno Research	115-035-144	1:4000	WB	
Goat anti-mouse IgG-peroxidase	Jackson Immuno Research	115-035-146	1:4000	WB	
CD24-BV421 (clone M1/29)	Biolegend	101826	1:400	FACS	
CD29-FITC (clone HMb1-1)	eBioscience	11-0291-82	1:400	FACS	
CD61-biotin (clone 2C9.G3)	eBioscience	13-0611-81	1:400	FACS	
anti-Ki67-APC (clone SolA15)	eBioscience	17-5698-82	1:200	FACS	
Streptavidin, APC-AF750	Invitrogen	SA1027	1:200	FACS	
Annexin V	eBioscience	88-8005-72	1:20	FACS	

Table S13: Drugs used for cell viability assays, with the indicated targets and the concentrations used.

Drug	Company	Target	Concentration (µM)
3-Methyladdenine (3MA)	TargetMol	Autophagy inhibitor	1mM
5-Fluorouracil (5-FU)	Targetmol	Thymidylate synthase (nucleotide synthesis)	0.4, 2, 10
A-1155463	Selleckchem/Targetmol	Bcl-xL	0.3, 1, 3, 10
ABT-737	Selleckchem	Bcl-2,Bcl-xL,Bcl-w	1, 3, 10
ABT-199	Selleckchem	Bcl-2	1, 3, 10
Adavosertib	Selleckchem	Wee1	1, 3, 10
Adavosertib(MK-1775)	Targetmol	Wee1	1, 3, 10
AZD6738	Targetmol	ATR	1, 3, 10
CB-839	Selleckchem	Glutaminase	1, 3, 10
Cisplatin	MedChem Express	DNA replication	1, 3, 10
Docetaxel	Targetmol	Beta-tubulin (microtubule inhibitor)	0.4, 2, 10
Doxorubicin	Targetmol	intercalating agent	0.04, 0.2, 1
Erastin	TargetMol	Ferroptosis inducer	0,5 - 1
Ferrostatin-1	TargetMol	Ferroptosis inhibitor	10, 20, 50, 100
Gefitinib	Calbiochem	EGFR	1, 3, 10
JNJ-7706621	Selleckchem	pan-Cdk, Aurora A/B	1, 3, 10
LY294002	Calbiochem	PI3K	1, 3, 10
Necrostatin-1	TargetMol	Necroptosis inhibitor	10, 20, 50, 100
Olaparib	TargetMol	PARP1/PARP2	1, 3, 10
PF-431396	Selleckchem	Pyk2/Fak	1, 3, 10
PHA-665752	Tocris Bioscience	Met	0.3, 1, 3
R547	Selleckchem/Sigma	Cdk1/2/4	1, 3, 10
SB225022	TargetMol	CXCR antagonist	1, 3, 10
Selumetinib	Selleckchem	Mek1/2	1, 3, 10
Sorafenib	Selleckchem	Raf-1, B-Raf, Vegfr2, Pdgfrβ, Flt3,Kit	1, 3, 10
Staurosporine	TargetMol	Apotosis inducer	150nM
WEHI-539	Apexbio	Bcl-xL	1, 3, 10
Z-VAD-FMK	TargetMol	Apoptosis inhibitor	10, 20, 50, 100

Table S14: Oligonucleotides used for RT-qPCR experiments.

Oligonucleotide (name)	Sequence (Forward)	Sequence (Reverse)
Met EXT(mouse)	GTTCTGCTTGGCAACGAGAGCT	GGAGAATGCACTGTATTGCGTCG
Met (mouse)	GAATTGTCTGCCATTACAGG	CAGCTCTTACTGTTATTGGCGC
Met (human)	CTGAAGCCGTTTTATGCAGC	GCCACAGGAAAAACCCAAGTAG
Hgf	GTCCTGAAGGCTCAGACTTGGT	CCAGCCGTAAATACTGCAAGTGG
B2M	ACAGTTCCACCCGCCTCACATT	TAGAAAGACCAGTCCTTGCTGAAG

Table S15: Proliferation capacity (mitotic index) of the *MMTV-R26*^{Met} cell lines - Statistical analysis was performed by One-way ANOVA followed by Tukey test.

	MGT7	MGT2	MGT4	MGT9	MGT11	MGT13
MGT7		0.549 (ns)	<0.0001 (***)	<0.0001 (***)	<0.0001 (***)	<0.0001 (***)
MGT2			<0.0001 (***)	<0.0001 (***)	<0.0001 (***)	<0.0001 (***)
MGT4				0.8339 (ns)	0.6485 (ns)	0.9936 (ns)
MGT9					0.9996 (ns)	0.9824 (ns)
MGT11						0.9121 (ns)
MGT13						

Full unedited gels for Figure 3h

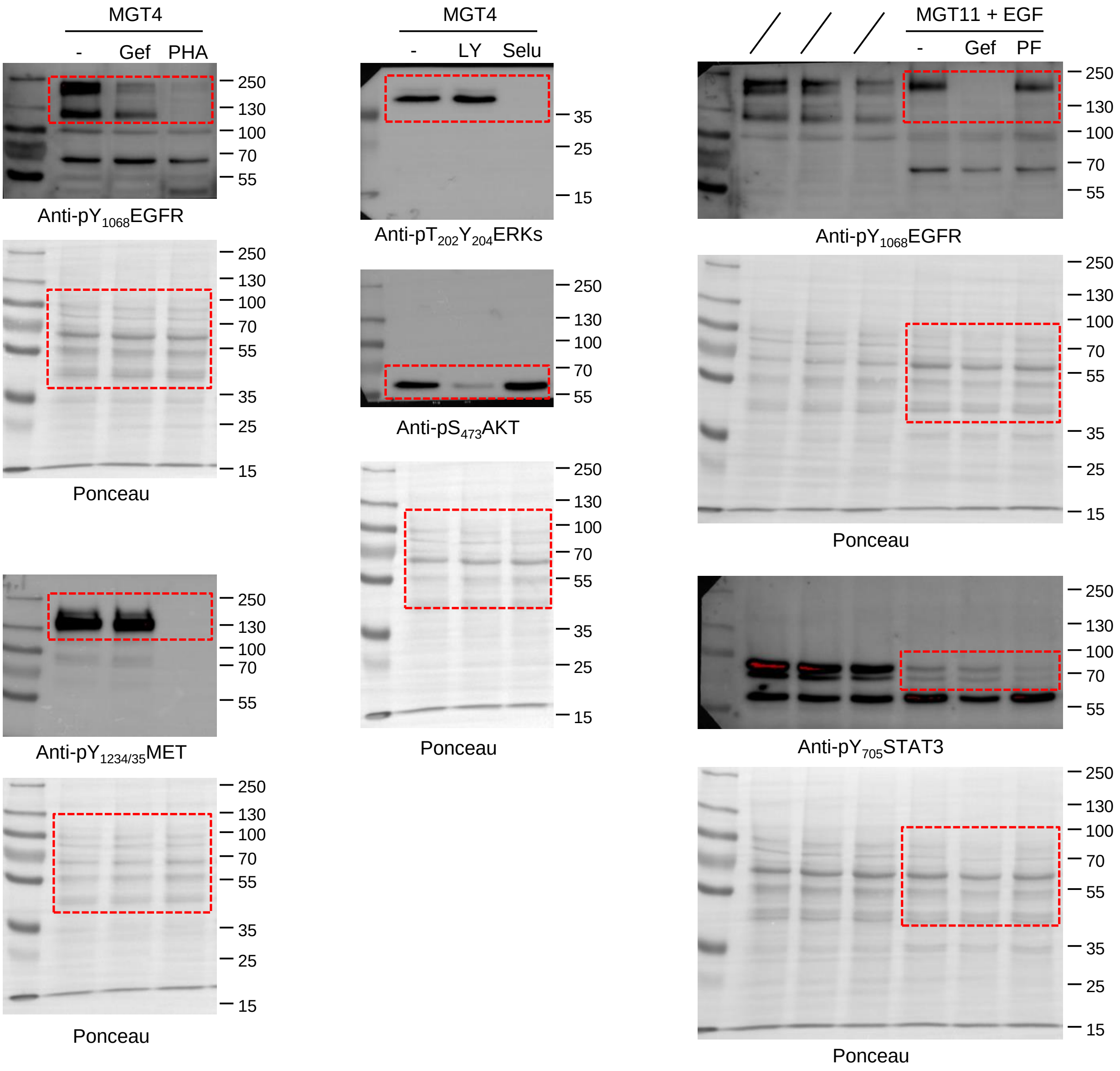

Full unedited gels for Figure 4f

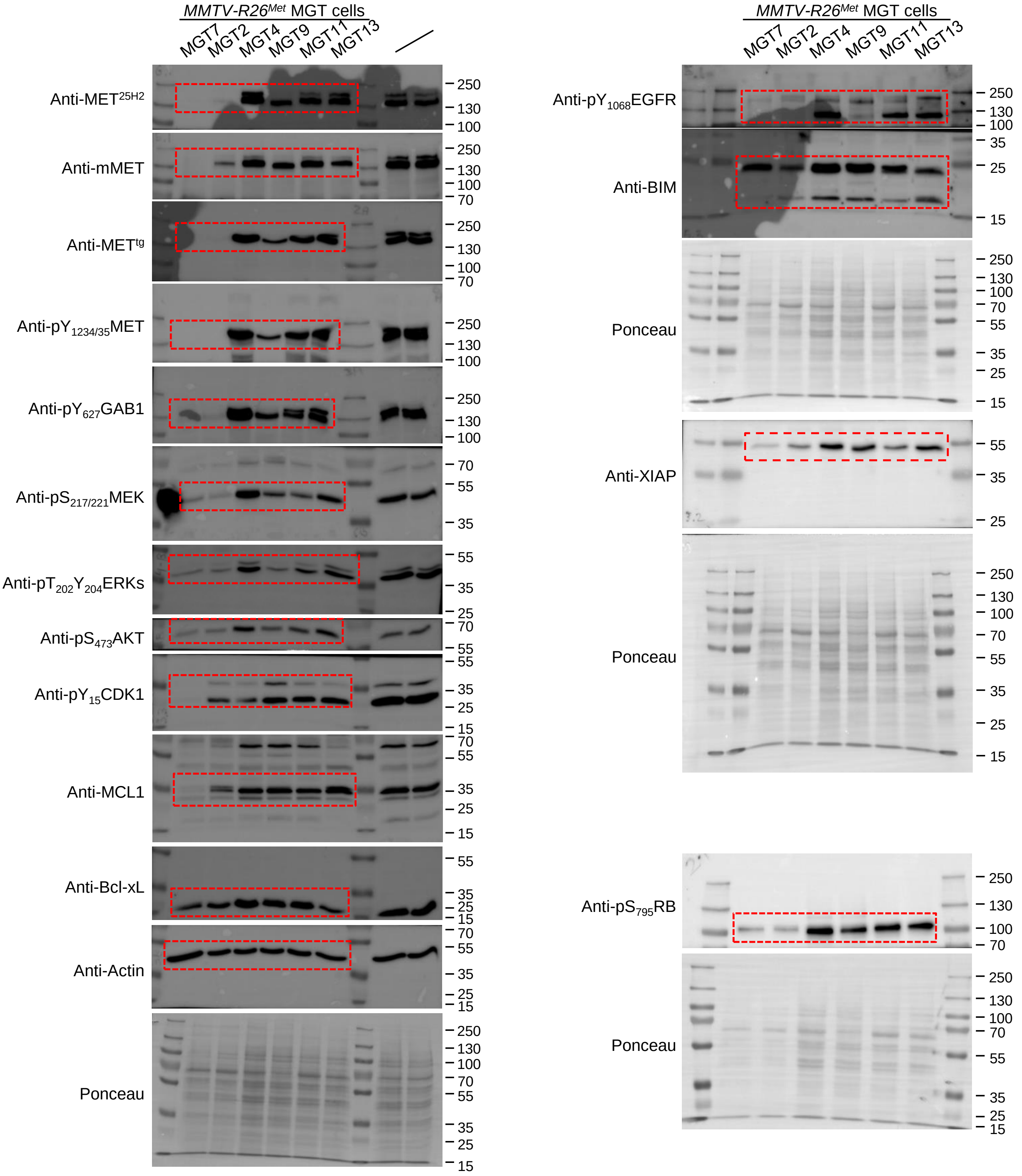

Full unedited gels for Figure 7a

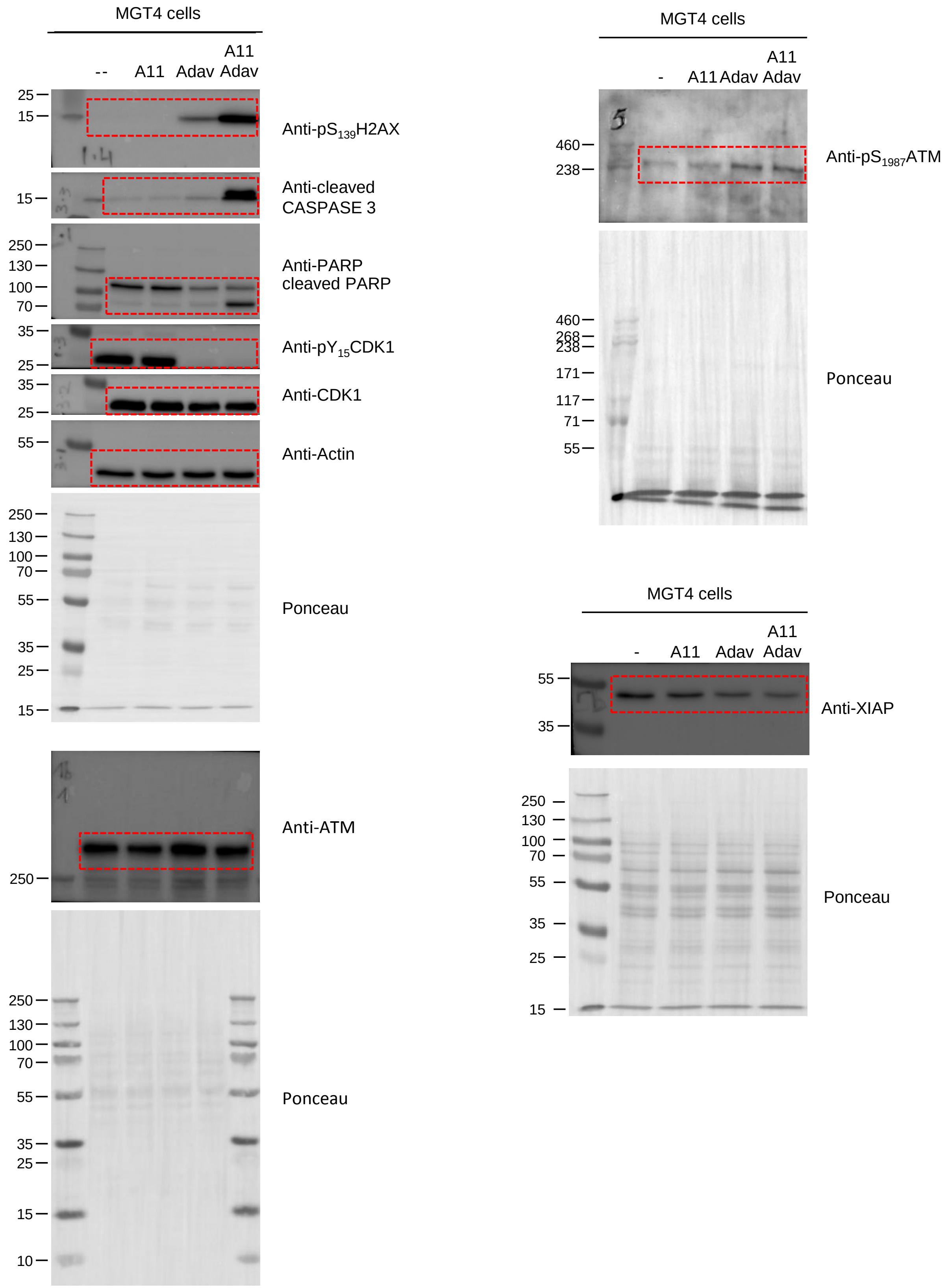

Full unedited gels for Figure 7a

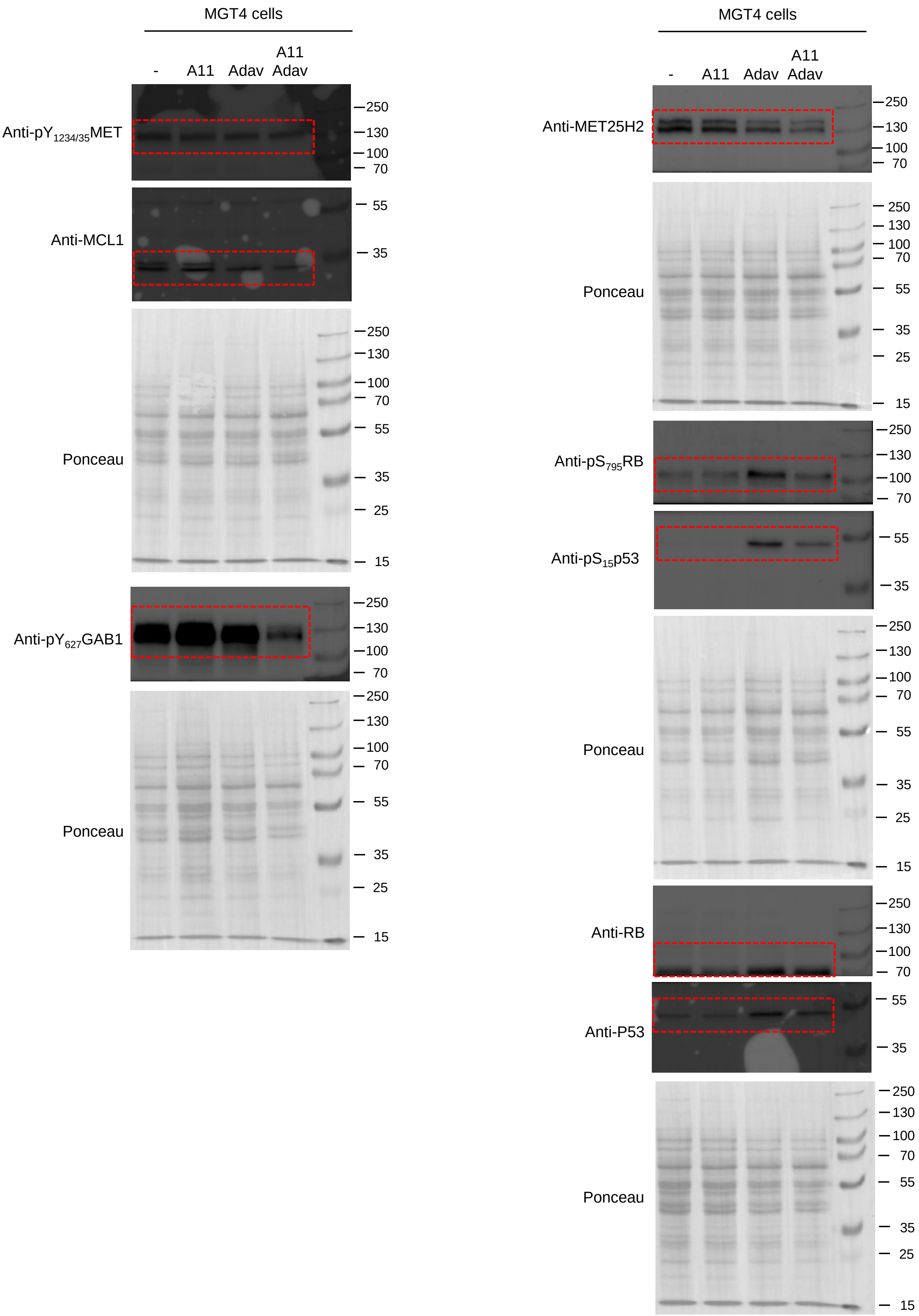

Full unedited gels for Figure 7a

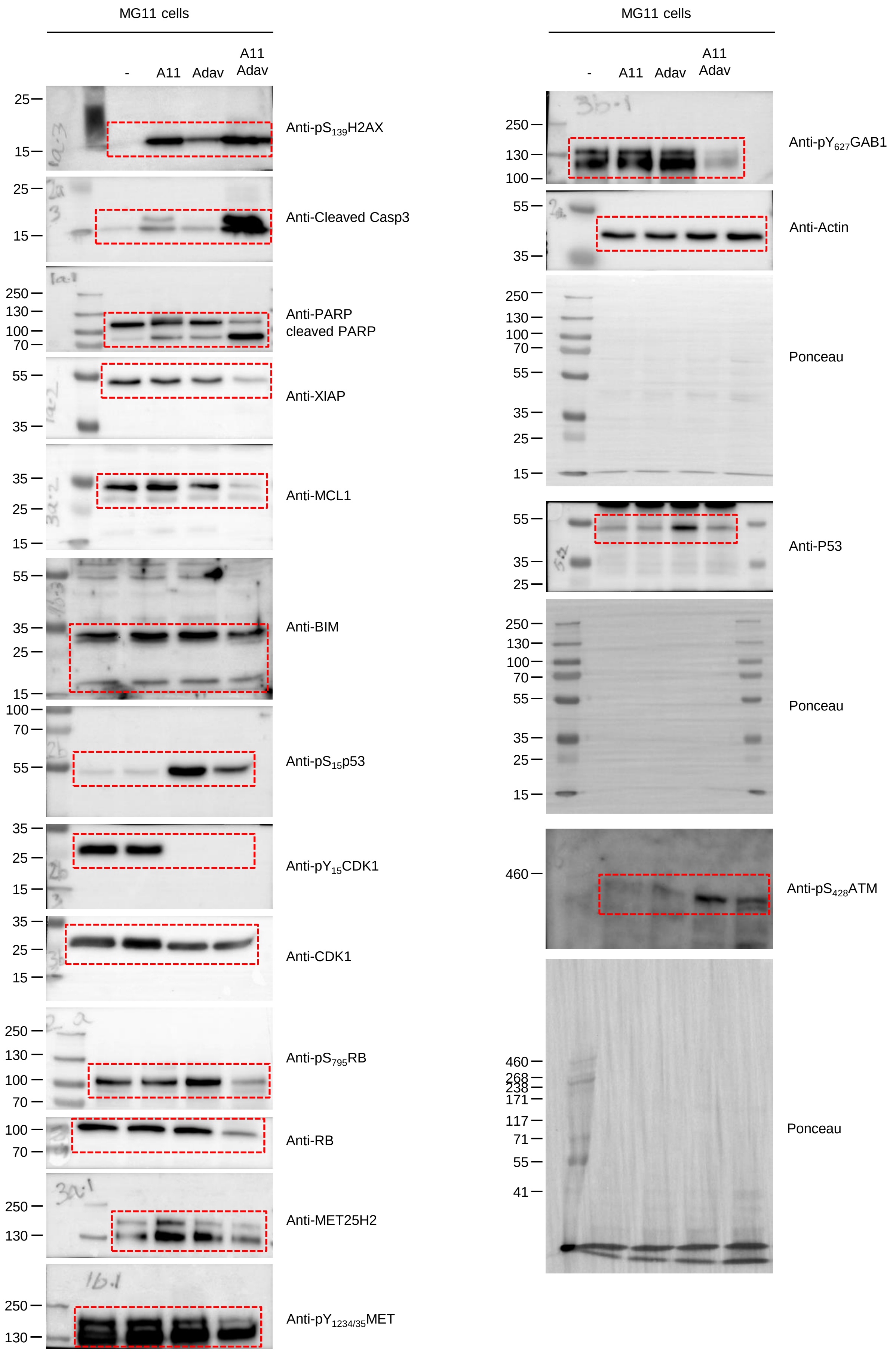

Full unedited gels for Figure 7a

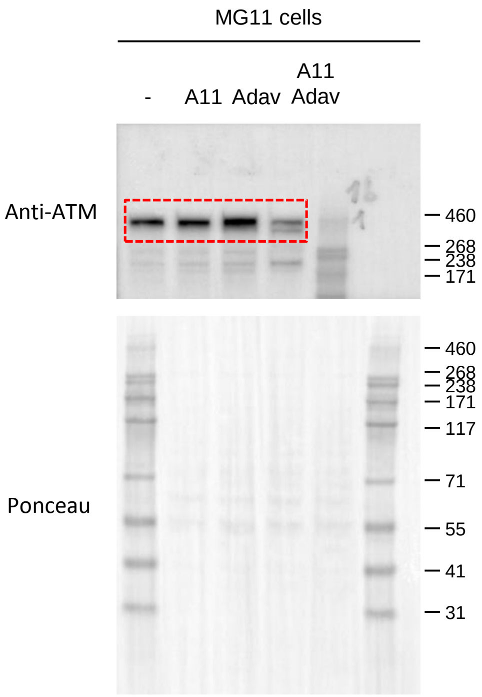

Full unedited gels for Figure 7d

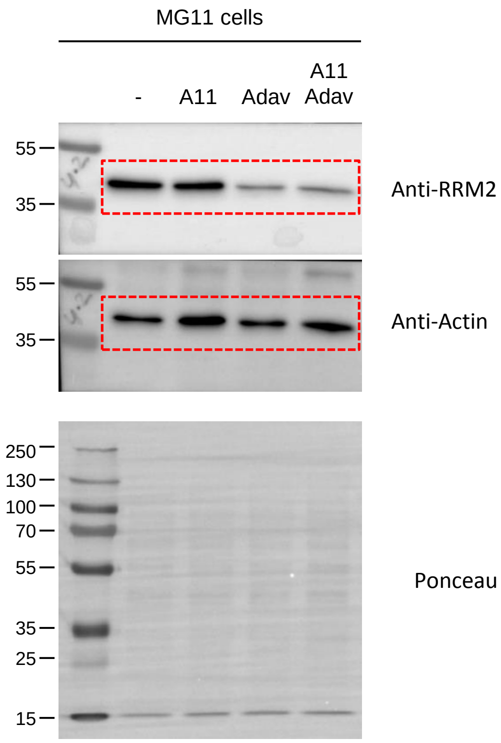

Full unedited gels for Figure S2b

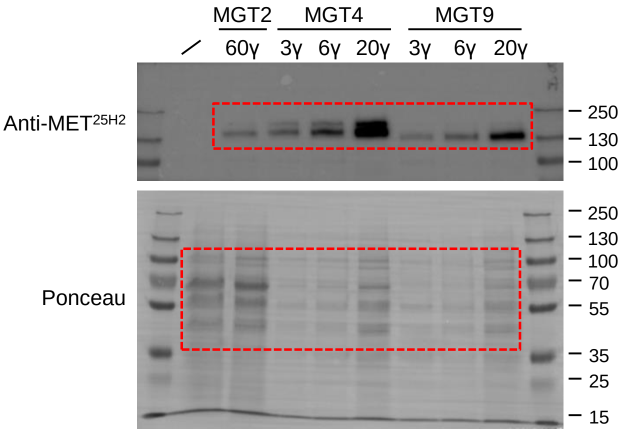

Full unedited gels for Figure S3e

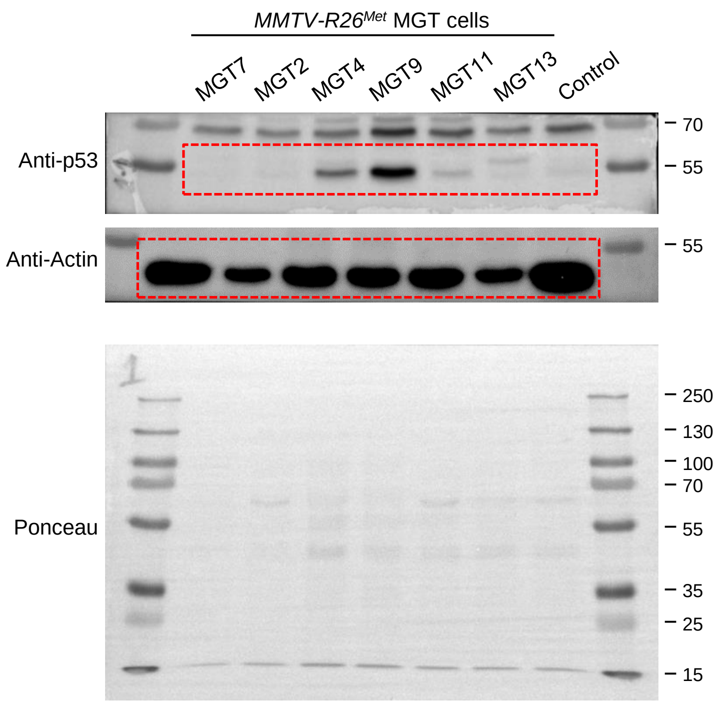

Full unedited gels for Figure S6a

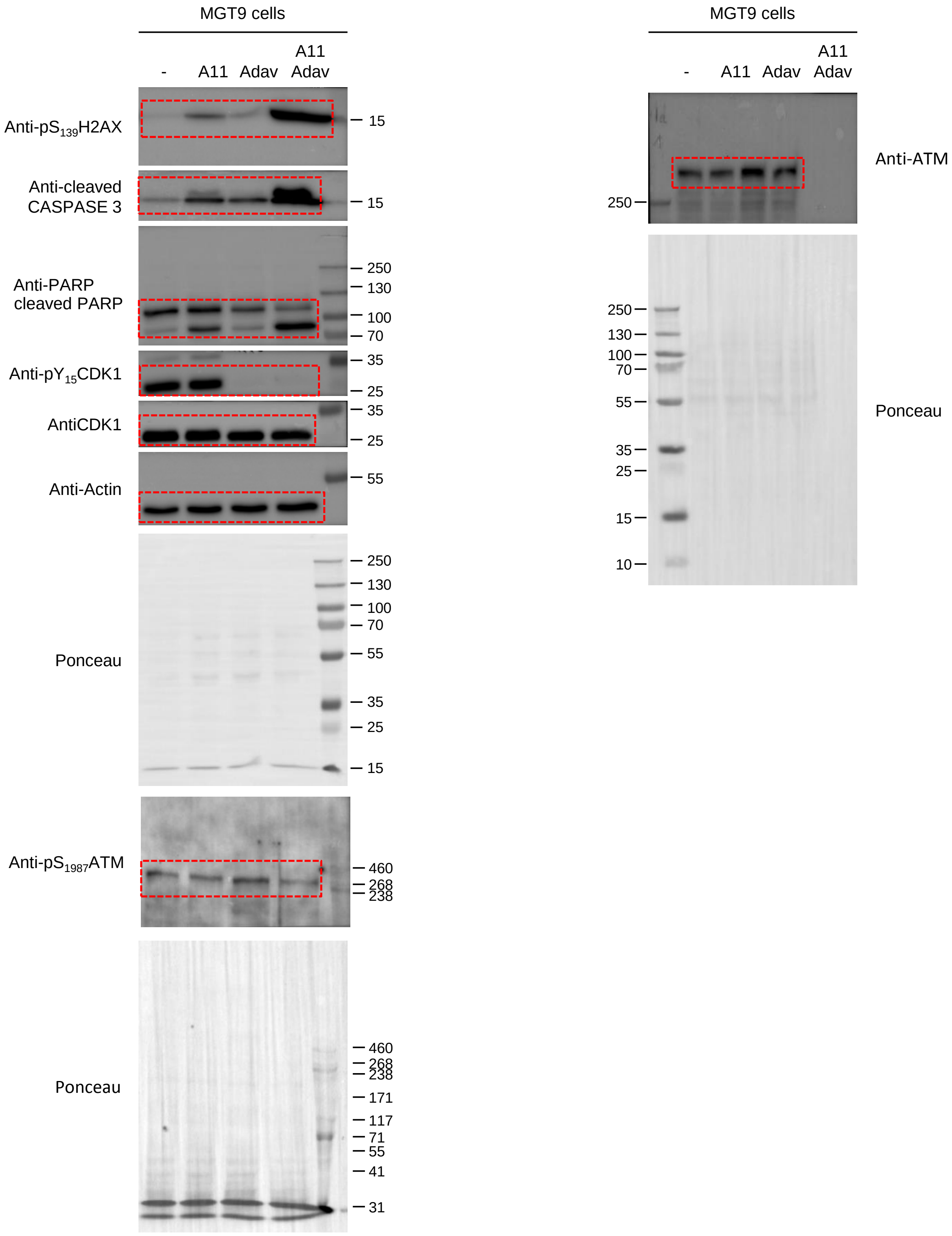

Full unedited gels for Figure S6a

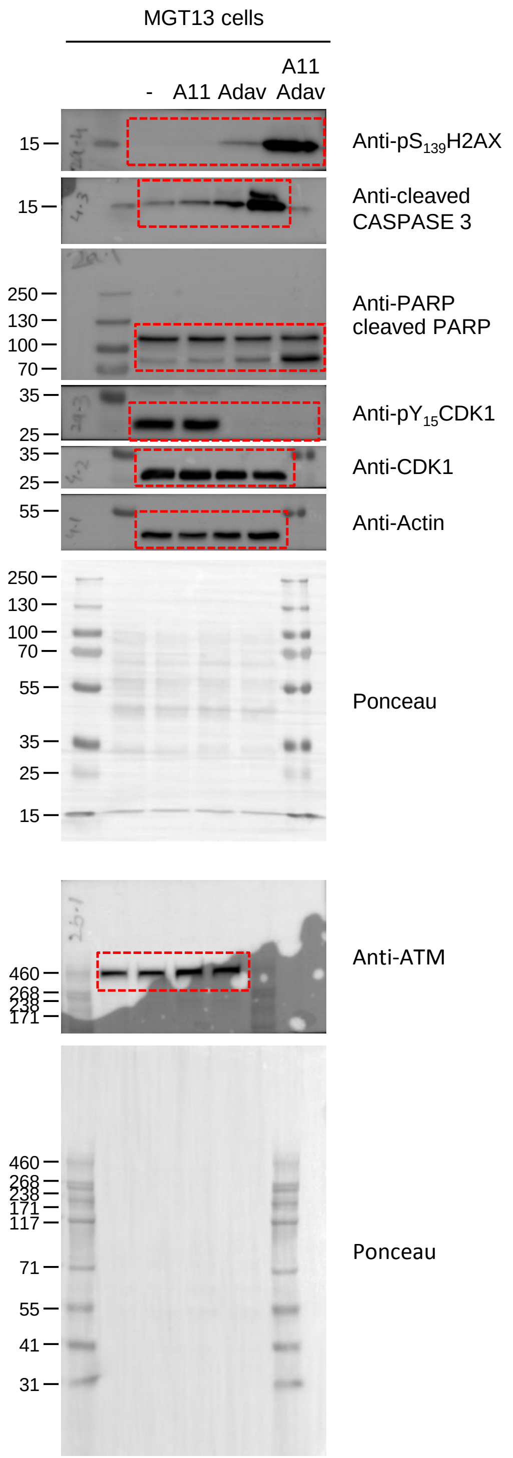

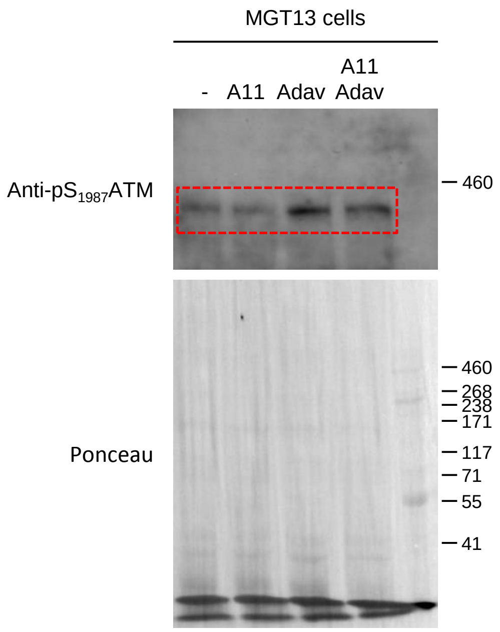

Full unedited gels for Figure S6b

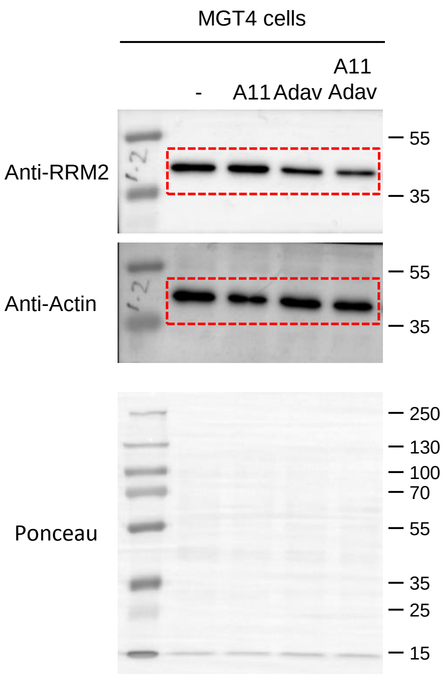

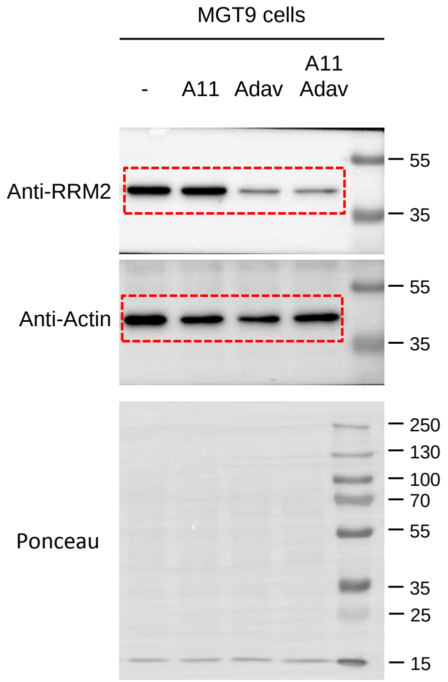

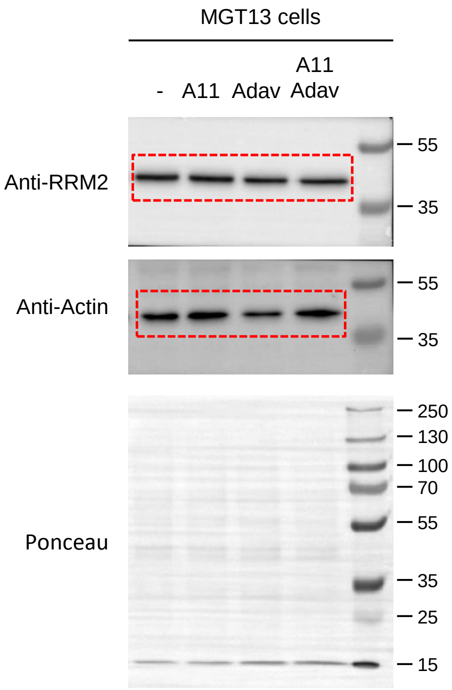

Supplement: Supplementary file 1 — Supporting Information [file ADVS-8-2003049-s001.pdf]
