# Supplementary material for: Modeling Heterogeneity of Triple‐Negative Breast Cancer Uncovers a Novel Combinatorial Treatment Overcoming Primary Drug Resistance
Source: Adv Sci (Weinh). 2020 Dec 16;8(3):2003049. doi: 10.1002/advs.202003049 (PMC7856896; doi:10.1002/advs.202003049)
Supplement: Supplementary file 2 — Supplemental Table 1 [file ADVS-8-2003049-s002.pdf]

Table S1: Mammary gland (tumors and controls) used for histopathological and RPPA analyses.

Mouse #	Genotype	Gross aspect of gland	Histopathological analysis						Lung metas-tases	RPPA (Tumor #)
			Tumor state	Grade	Necrosis	Prolif. status	ER/PR/ HER2 scoring	Met		
669 201	*MMTV-R26^{Met}*	Tumour	carcinoma	nd	nd	nd	0	nd	Yes	Yes (T1)
669 352	*MMTV-R26^{Met}*	Tumour	carcinoma	nd	+	nd	0	nd	Yes	Yes (T2)
669 713	*MMTV-R26^{Met}*	Tumour	carcinoma	nd	nd	nd	0	nd	_	Yes (T3)
669 163	*MMTV-R26^{Met}*	Tumour	carcinoma	nd	nd	nd	0	nd	_	Yes (T4)
669 789	*MMTV-R26^{Met}*	Tumour	carcinoma	nd	nd	nd	0	nd	Yes	Yes (T5)
669 722	*MMTV-R26^{Met}*	Tumour	carcinoma	nd	nd	nd	0	nd	Yes	Yes (T6)
669 175	*MMTV-R26^{Met}*	Tumour	carcinoma	nd	nd	nd	0	nd	_	Yes (T7)
669 715	*MMTV-R26^{Met}*	Tumour	carcinoma	nd	nd	nd	0	nd	_	Yes (T8)
671 802 (Tumor A)	*MMTV-R26^{Met}*	Tumour	carcinoma	3	+	3	0	+	_	Yes (T9)
671 607	*MMTV-R26^{Met}*	Tumour	carcinoma	3	+	3	0	+	_	Yes (T10)
671 662	*MMTV-R26^{Met}*	Tumour	carcinoma	3	++	2	0	+	_	Yes (T11)
671 670	*MMTV-R26^{Met}*	Tumour	carcinoma	3	+(+)	3	0	+	Yes	Yes (T12)
671 975	*MMTV-R26^{Met}*	Tumour	carcinoma	2	_	1	0	+	_	Yes (T13)
672 961	*MMTV-R26^{Met}*	Tumour	carcinoma	3	++	3	0	+	_	Yes (T14)
671 711	*MMTV-R26^{Met}*	Tumour	carcinoma	3	++	2	0	+	_	Yes (T15)
672 046	*MMTV-R26^{Met}*	Tumour	carcinoma	3	_	2	0	+	_	Yes (T16)
671 893	*MMTV-R26^{Met}*	Tumour	carcinoma	3	_	2	0	+	Yes	Yes (T17)
672 867	*MMTV-R26^{Met}*	Tumour	carcinoma	3	+	2	0	+	_	Yes (T18)
672 790	*MMTV-R26^{Met}*	Tumour	carcinoma	2	_	2	0	+	_	Yes (T19)
672 955	*MMTV-R26^{Met}*	Tumour	carcinoma	2-3	+	2	0	+	_	Yes (T20)
672 941	*MMTV-R26^{Met}*	Tumour	carcinoma	3	_	2	0	+	_	Yes (T23)

672 876	*MMTV-R26*^{Met}	Tumour	nd	nd	nd	nd	nd	nd	_	Yes (T21)
672 302	*MMTV-R26*^{Met}	Tumour	nd	nd	nd	nd	nd	nd	_	Yes (T22)
672 870	*MMTV-R26*^{Met}	Tumour	nd	nd	nd	nd	nd	nd	_	Yes (T24)
671 509	*MMTV-R26*^{Met}	Tumour	carcinoma	3	+	2	0	+	_	_
671 802 (Tumor B)	*MMTV-R26*^{Met}	Tumour	carcinoma	3	_	1	0	+	_	_
672 802	*MMTV-R26*^{Met}	Tumour	carcinoma	3	+	2	0	+	_	_
669 527	*MMTV-R26*^{Met}	Tumour	carcinoma	nd	nd	nd	nd	nd	_	_
669 355	*MMTV-R26*^{Met}	Tumour	carcinoma	nd	nd	nd	nd	nd	_	_
669 717	*MMTV-R26*^{Met}	Tumour	carcinoma	nd	nd	nd	nd	nd	_	_
669 854	*MMTV-R26*^{Met}	Tumour	carcinoma	nd	nd	nd	nd	nd	_	_
671 720	*MMTV-R26*^{Met}	Tumour	nd	nd	nd	nd	nd	nd	_	_
672 246	*MMTV-R26*^{Met}	Tumour	nd	nd	nd	nd	nd	nd	_	_
671 594	*MMTV*	Normal	_	nd	_	nd	nd	-	_	Yes (N1)
671 600	*MMTV*	Normal	_	nd	_	nd	nd	-	_	Yes (N2)
672 603	*MMTV-R26*^{Met}	Normal	_	nd	_	nd	nd	nd	_	Yes (N3)
