# Supplementary material for: Modeling Heterogeneity of Triple‐Negative Breast Cancer Uncovers a Novel Combinatorial Treatment Overcoming Primary Drug Resistance
Source: Adv Sci (Weinh). 2020 Dec 16;8(3):2003049. doi: 10.1002/advs.202003049 (PMC7856896; doi:10.1002/advs.202003049)
Supplement: Supplementary file 3 — Supplemental Table 2 [file ADVS-8-2003049-s003.pdf]

Table S2: Antibodies used for RPPA analysis of *MMTV-R26^{Met}* tumors and cells

#	Official Ab Name	Ab Name Reported on Dataset	Gene Name	Company	Catalog #	Species	RPPA Dilution
1	14-3-3 beta	14-3-3-beta	YWHAB	Santa Cruz	sc-628	Rabbit	1:75
2	14-3-3 epsilon	14-3-3-epsilon	YWHAE	Santa Cruz	SC-23957	Mouse	1:50
3	14-3-3 zeta	14-3-3-zeta	YWHAZ	Santa Cruz	sc-1019	Rabbit	1:5000
4	4E-BP1	4E-BP1	EIF4EBP1	CST	9452	Rabbit	1:100
5	4E-BP1 (phospho S65)	4E-BP1_pS65	EIF4EBP1	CST	9456	Rabbit	1:250
6	4E-BP1 (phospho T37/46)	4E-BP1-pT37-T46	EIF4EBP1	CST	9459	Rabbit	1:2000
7	53BP1	53BP1	TP53BP1	CST	4937	Rabbit	1:300
8	A1Up	UBQLN4	UBQLN4	Santa Cruz	sc-136145	Mouse	1:125
9	Acetyl-CoA-Carboxylase	ACC1	ACACA, B	Epitomics/ Abcam	1768-1/ ab45174	Rabbit	1:1500
10	Acetyl-CoA-Carboxylase (phospho S79)	ACC_pS79	ACACA, B	CST	3661	Rabbit	1:500
11	ACSL1 (D2H5)	ACSL1	ACSL1	CST	9189	Rabbit	1:500
12	ACVRL1	ACVRL1	ACVRL1	Epitomics/ Abcam	2940-1/ ab108207	Rabbit	1:30
13	ADAR1	ADAR1	ADAR	Abcam	ab88574	Mouse	1:100
14	Akt	Akt	AKT1, 2, 3	CST	4691	Rabbit	1:7500
15	Akt (phospho S473)	Akt_pS473	AKT1, 2, 3	CST	9271	Rabbit	1:150
16	Akt (phospho T308)	Akt_pT308	AKT1, 2, 3	CST	2965	Rabbit	1:250
17	Akt1	Akt1	AKT1	CST	2938	Rabbit	1:1000
18	Akt1 (phospho S473)	Akt1_pS473	AKT1	CST	9018	Rabbit	1:1000
19	Akt2	Akt2	AKT2	CST	3063	Rabbit	1:3000
20	Akt2 (phospho S474)	Akt2_pS474	AKT2	CST	8599	Rabbit	1:1000
21	Ambra1 (phospho S52)	Ambra1_pS52	AMBRA1	Millipore	ABC80	Rabbit	1:250
22	AMPK alpha 2 (phospho S345)	AMPK-a2_pS345	PRKAA1, 2	Abcam	ab129081	Rabbit	1:200
23	AMPKa	AMPKa	PRKAA1, 2	CST	2532	Rabbit	1:75

**24**	AMPKa (phospho T172)	AMPKa_pT172	PRKAA1, 2	CST	2535	Rabbit	1:100
**25**	Androgen Receptor (D6F11)	AR	AR	CST	5153	Rabbit	1:250
**26**	Annexin I	Annexin-I	ANXA1	BD Biosciences	610066	Mouse	1:5000
**27**	Annexin VII	Annexin-VII	ANXA7	BD Biosciences	610668	Mouse	1:20
**28**	A-Raf	A-Raf	ARAF	CST	4432	Rabbit	1:200
**29**	A-Raf (phospho S299)	A-Raf_pS299	ARAF	CST	4431	Rabbit	1:25
**30**	ARID1A	ARID1A	ARID1A	Sigma-Aldrich	HPA005456	Rabbit	1:1000
**31**	ASNS	ASNS	ASNS	Sigma-Aldrich	HPA029318	Rabbit	1:500
**32**	Atg3	Atg3	ATG3	CST	3415	Rabbit	1:72
**33**	Atg4B	Atg4B	ATG4B	CST	13507	Rabbit	1:200
**34**	Atg5	Atg5	ATG5	CST	12994	Rabbit	1:1000
**35**	Atg7	Atg7	ATG7	CST	8558	Rabbit	1:1000
**36**	ATM	ATM	ATM	CST	2873	Rabbit	1:250
**37**	ATM (phospho S1981)	ATM_pS1981	ATM	CST	5883	Rabbit	1:20
**38**	ATP5A	ATP5A	ATP5A	Abcam	ab14748	Mouse	1:500
**39**	ATP5H	ATP5H	ATP5H	Abcam	ab110275	Mouse	1:30
**40**	ATR	ATR	ATR	CST	2790	Rabbit	1:30
**41**	ATR (phospho S428)	ATR_pS428	ATR	Abcam	ab178407	Rabbit	1:1000
**42**	ATRX	ATRX	ATRX	Abcam	ab97508	Rabbit	1:300
**43**	Aurora B/AIM1	Aurora-B	AURKB	CST	3094	Rabbit	1:38
**44**	Axl	Axl	AXL	CST	8661	Rabbit	1:500
**45**	B7-H3	B7-H3	CD276	CST	14058	Rabbit	1:200
**46**	B7-H4	B7-H4	VTCN1	CST	14572	Rabbit	1:50
**47**	Bad (phospho S112)	Bad_pS112	BAD	CST	9291	Rabbit	1:50
**48**	Bak	Bak	BAK1	Epitomics/ Abcam	1542-1/ ab32371	Rabbit	1:400
**49**	BAP1	BAP1	BAP1	Santa Cruz	sc-28383	Mouse	1:200
**50**	Bax	Bax	BAX	CST	2772	Rabbit	1:100
**51**	b-Catenin	b-Catenin	CTNNB1	CST	9562	Rabbit	1:1500
**52**	Bcl2	Bcl2	BCL2	Dako	M0887	Mouse	1:50
**53**	Bcl2A1	Bcl2A1	BCL2A1	Abnova	PAB8528	Rabbit	1:250
**54**	Bcl-xL	Bcl-xL	BCL2L1	CST	2762	Rabbit	1:100

**55**	Beclin 1	Beclin	BECN1	ThermoFisher	PA1-16857	Rabbit	1:500
**56**	beta Actin	b-Actin	ACTB	CST	4970	Rabbit	1:50
**57**	beta Catenin (phospho T41/S45)	b-Catenin_pT41_S45	CTNNB1	CST	9565	Rabbit	1:30
**58**	Bid	Bid	BID	CST	2002	Rabbit	1:500
**59**	Bim (C34C5)	Bim	BCL2L11	Epitomics/ Abcam	1036-1/ ab32158	Rabbit	1:400
**60**	BiP/GRP78	BiP-GRP78	HSPA5	BD Biosciences	610978	Mouse	1:150
**61**	BMK1/Erk5 (phospho T218/Y220)	BMK1-Erk5_pT218_Y220	MAPK7	Millipore	07-507	Rabbit	1:500
**62**	B-Raf	B-Raf	BRAF	CST	14814	Rabbit	1:500
**63**	B-Raf (phospho S445)	B-Raf_pS445	BRAF	CST	2696	Rabbit	1:75
**64**	BRD4	BRD4	BRD4	CST	13440	Rabbit	1:1000
**65**	CA9 (CAIX)	CA9	CA9	CST	5649	Rabbit	1:200
**66**	c-Abl	c-Abl	ABL1	CST	2862	Rabbit	1:100
**67**	c-Abl (phospho Y412)	Abl_pY412	ABL1	CST	2865	Rabbit	1:200
**68**	Caspase 3 (cleaved asp175)	Caspase-3-cleaved	CASP3	CST	9661	Rabbit	1:500
**69**	Caspase 7 (cleaved)	Caspase-7-cleaved	CASP7	CST	9491	Rabbit	1:60
**70**	Caspase 8	Caspase-8	CASP8	CST	9746	Mouse	1:150
**71**	Caspase 8 (cleaved asp391)	Caspase-8-cleaved	CASP8	CST	9496	Rabbit	1:500
**72**	Caspase-3	Caspase-3	CASP3	Epitomics/ Abcam	1476-1/ ab32042	Rabbit	1:250
**73**	Caveolin 1	Caveolin-1	CAV1	CST	3238	Rabbit	1:3000
**74**	CD134/OX40	CD134	TNFRSF	Abcam	ab76000	Rabbit	1:100
**75**	CD171 (L1)	CD171	L1CAM	Biolegend	826701	Mouse	1:1000
**76**	CD20	CD20	MS4A1	Epitomics/ Abcam	1632-1/ ab78237	Rabbit	1:75
**77**	CD26	CD26	DPP4	Abcam	ab28340	Rabbit	1:1000
**78**	CD29	CD29	ITGB1	BD Biosciences	610467	Mouse	1:30
**79**	CD31	CD31	PECAM1	Dako/ Fisher	M0823/ MS353S	Mouse	1:25
**80**	CD38	CD38	CD38	Abcam	ab108403	Rabbit	1:250
**81**	CD4	CD4	CD4	Abcam	ab133616	Rabbit	1:500

**82**	CD44	CD44	CD44	CST	3570	Mouse	1:20
**83**	CD45	CD45	CD45	DAKO/ ThermoFisher	M070129-2/ MS355P	Mouse	1:1000
**84**	CD49b	CD49b	ITGA2	BD Biosciences	611016	Mouse	1:50
**85**	CD86	CD86	CD86	Abcam	ab53004	Rabbit	
**86**	Cdc2 (phospho Y15)	cdc2_pY15	CDK	CST	4539	Rabbit	1:38
**87**	cdc25C	cdc25C	CDC25C	CST	4688	Rabbit	1:250
**88**	CDK1/2/3 (phospho T14)	CDK1_pT14	CDK1, 2, 3	Abcam	ab32384	Rabbit	1:1000
**89**	CDKN2A/p16INK4 a	p16INK4a	CDKN2A	Abcam	ab81278	Rabbit	1:500
**90**	Chk1	Chk1	CHEK	CST	2360	Mouse	1:100
**91**	Chk1 (phospho S296)	Chk1_pS296	CHEK1	Abcam	ab79758	Rabbit	1:125
**92**	Chk1 (phospho S345)	Chk1_pS345	CHEK1	CST	2348	Rabbit	1:30
**93**	Chk2	Chk2	CHEK2	CST	3440	Mouse	1:50
**94**	Chk2 (phospho T68)	Chk2_pT68	CHEK2	CST	2197	Rabbit	1:250
**95**	c-IAP2	c-IAP2	BIRC3	CST	3130	Rabbit	1:50
**96**	CIITA	CIITA	CIITA	CST	3793	Rabbit	1:250
**97**	c-Jun (phospho S73)	c-Jun_pS73	JUN	CST	9164	Rabbit	1:30
**98**	c-Kit	c-Kit	KIT	Epitomics/ Abcam	1522-1/ ab32363	Rabbit	1:250
**99**	Claudin 7	Claudin-7	CLDN7	Abcam	ab79481	Rabbit	1:250
**100**	c-Myc	c-Myc	MYC	Santa Cruz	sc-764	Rabbit	1:250
**101**	COG3	COG3	COG3	ProteinTech	11130-1-AP	Rabbit	1:750
**102**	Collagen-VI/COL6A1	Collagen-VI	COL6A1	Santa Cruz	sc-20649	Rabbit	1:6000
**103**	Complex II Subunit	Complex-II-Subunit	SDHB	Life Technologies	459230	Mouse	1:200
**104**	Connexin 43	Connexin-43	GJA1	CST	3512	Rabbit	1:150
**105**	Coup-TFII	Coup-TFII	NR2F2	CST	6434	Rabbit	1:50
**106**	Cox2	Cox2	PTGS2	CST	4842	Rabbit	1:75
**107**	Cox-IV	Cox-IV	COX4I1	CST	4850	Rabbit	1:5000
**108**	C-Raf	C-Raf	RAF1	Millipore	04-739	Rabbit	1:100
**109**	C-Raf (phospho S338)	C-Raf_pS338	RAF1	CST	9427	Rabbit	1:200

**110**	Creb	Creb	CREB1	CST	9197	Rabbit	1:75
**111**	CSK	CSK	CSK	CST	4980	Rabbit	1:300
**112**	CtIP	CtIP	RBBP8	CST	9201	Rabbit	1:500
**113**	Cyclin B1	Cyclin B1	CCNB1	Epitomics/ Abcam	1495-1/ ab32053	Rabbit	1:1500
**114**	Cyclin D1	Cyclin-D1	CCND1	Millipore Sigma	SAB4502603	Rabbit	1:200
**115**	Cyclin D3	Cyclin D3	CCND3	CST	2936	Mouse	1:1000
**116**	Cyclin E1	Cyclin E1	CCNE1	Santa Cruz	sc-247	Mouse	1:25
**117**	Cyclophilin-F	Cyclophilin-F	PPIF	Abcam	MSA04/ ab110324	Mouse	1:50000
**118**	Cytokeratin 19	Cytokeratin-19	KRT19	Dako	M0888	Mouse	1:50
**119**	DAP Kinase 1 (phospho S308)	DAPK1_pS308	DAPK1	GeneTex	GTX10524	Mouse	1:200
**120**	DAP Kinase 2	DAPK2	DAPK2	Abcam	ab51601	Rabbit	1:250
**121**	DDB-1	DDB-1	DDB1	CST	6998	Rabbit	1:5000
**122**	Detyrosinated alpha-Tubulin	D-a-Tubulin	TUBA4A, TUBA3C	Abcam	ab48389	Rabbit	1:1500
**123**	Di-Methyl-Histone H3 (Lys4/C64G9)	DM-Histone-H3	HIST1H3A	CST	9725	Rabbit	1:100
**124**	Dimethyl-K9 Histone H3	DM-K9-Histone-H3	HIST3H3	Abcam	ab1220	Mouse	1:250
**125**	DNA Ligase IV	DNA-Ligase-IV	LIG4	CST	14649	Rabbit	1:1000
**126**	DNA Polymerase gamma (D1Y6R)	POLG	POLG	CST	13609	Rabbit	1:500
**127**	DNMT1 (D63A6)	DNMT1	DNMT1	CST	5032	Rabbit	1:500
**128**	DRP1 (D8H5)	DRP1	DNM1L	CST	5391	Rabbit	1:1000
**129**	DUSP4/MKP2	DUSP4	DUSP4	CST	5149	Rabbit	1:150
**130**	DUSP6	DUSP6	DUSP6	Abcam	ab76310	Rabbit	1:750
**131**	Dvl3	Dvl3	DVL3	CST	3218	Rabbit	1:30
**132**	E2F1	E2F1	E2F1	Santa Cruz	sc-251	Mouse	1:20
**133**	E-Cadherin	E-Cadherin	CDH1	CST	3195	Rabbit	1:150
**134**	eEF2	eEF2	EEF2	CST	2332	Rabbit	1:50
**135**	eEF2K	eEF2K	EEF2K	CST	3692	Rabbit	1:50
**136**	EGFR	EGFR	EGFR	CST	2232	Rabbit	1:75
**137**	EGFR (phospho Y1173)	EGFR_pY1173	EGFR	Epitomics/ Abcam	1124-1/ ab32578	Rabbit	1:300
**138**	eIF4E	eIF4E	EIF4E	CST	9742	Rabbit	1:75
**139**	eIF4E (phospho S209)	eIF4E_pS209	EIF4E	Abcam	ab76256	Rabbit	1:250

**140**	eIF4G	eIF4G	EIF4G1	CST	2498	Rabbit	1:1000
**141**	Elk1 (phospho S383)	Elk1_pS383	ELK1	CST	9181	Rabbit	1:50
**142**	Enolase-2 (D20H2)	Enolase-2	ENO2	CST	8171	Rabbit	1:250
**143**	ENY2	ENY2	ENY2	GeneTex	GTX629542	Mouse	1:500
**144**	Eph Receptor A2	EPHA2	EPHA2	Abcam	ab133501	Rabbit	1:1000
**145**	Epithelial Membrane Antigen	EMA	MUC1	DAKO	M061329-2	Mouse	1:750
**146**	ErbB3/HER3	HER3	ERBB3	Santa Cruz	sc-285	Rabbit	1:300
**147**	ErbB3/HER3 (phospho Y1289)	HER3_pY1289	ERBB3	CST	4791	Rabbit	1:50
**148**	ERCC1	ERCC1	ERCC1	Santa Cruz	sc-17809	Mouse	1:38
**149**	Erk5	Erk5	MAPK7	CST	3552	Rabbit	1:500
**150**	ERRalpha (E1G1J)	ERRalpha	ESRRA	CST	13826	Rabbit	1:500
**151**	ERRFI1/MIG6	MIG6	ERRFI1	Sigma-Aldrich	WH0054206 M1	Mouse	1:50
**152**	Estrogen Receptor	ER	ESR1	Lab Vision	RM-9101	Rabbit	1:40
**153**	Estrogen Receptor alpha	ER-a	ERSA	CST	13258	Rabbit	1:500
**154**	Estrogen Receptor alpha (phospho S118)	ER-a_pS118	ESR1	Epitomics/ Abcam	1091-1/ ab32396	Rabbit	1:500
**155**	Ets-1	Ets-1	ETS1	Bethyl	A303-501A	Rabbit	1:100
**156**	FAK	FAK	PTK2	Epitomics/ Abcam	1700-1/ ab40794	Rabbit	1:1000
**157**	FAK (phospho Y397)	FAK_pY397	PTK2	CST	3283	Rabbit	1:25
**158**	Fatty Acid Synthase	FASN	FASN	CST	3180	Rabbit	1:1000
**159**	FGF-basic	FGF-basic	FGF2	VWR	10775-082 (500-P18)	Rabbit	1:1000
**160**	Fibronectin	Fibronectin	FN1	Epitomics	1574-1	Rabbit	1:10000
**161**	FoxM1	FOXM1	FOXM1	CST	5436	Rabbit	1:30
**162**	FoxO3a	FoxO3a	FOXO3	CST	2497	Rabbit	1:20
**163**	FoxO3a (phospho S318/S321)	FoxO3a_pS318_S3 21	FOXO3	CST	9465	Rabbit	1:30
**164**	FRS2-a (phospho Y196)	FRS2-a_pY196	FRS2	CST	3864	Rabbit	1:100
**165**	G6PD	G6PD	G6PD	Santa Cruz	sc-373887	Mouse	1:75

**166**	Gab2	Gab2	GAB2	CST	3239	Rabbit	1:300
**167**	GAPDH	GAPDH	GAPDH	Ambion/ Invitrogen	AM4300	Mouse	1:75000
**168**	GATA3	GATA3	GATA3	BD Biosciences	558686	Mouse	1:150
**169**	GATA6	GATA6	GATA6	CST	5851	Rabbit	1:200
**170**	GCLC	GCLC	GCLC	Proteintech Group	12601-1-AP	Rabbit	1:500
**171**	GCLM	GCLM	GCLM	Abcam	ab124827	Rabbit	1:500
**172**	GCN5L2	GCN5L2	KAT2A	CST	3305	Rabbit	1:30
**173**	Gli1	Gli1	GLI1	CST	3538	Rabbit	1:3000
**174**	Gli3	Gli3	GLI3	Abcam	ab69838	Rabbit	1:1000
**175**	Glucose-6 Phosphate Dehydrogenase	G6PD	G6PD	CST	8866	Rabbit	1:30
**176**	Glutamate Dehydrogenase1/ 2	Glutamate-D1-2	GLUD1	Novus	NBP2-16679	Rabbit	1:500
**177**	Glutaminase	Glutaminase	GLS	Abcam	ab156876	Rabbit	1:150
**178**	Glycogen Synthase	Gys	GYS1	CST	3886	Rabbit	1:2000
**179**	Glycogen Synthase (phospho S641)	Gys_pS641	GYS1	CST	3891	Rabbit	1:300
**180**	GPBB	GPBB	PYGM	Novus	NBP1-32799	Rabbit	1:200
**181**	Granzyme B	Granzyme-B	GZMB	CST	4275	Rabbit	1:500
**182**	GRB7	GRB7	GRB7	Abcam	ab183737	Rabbit	1:500
**183**	Grp75 (D13H4)	Grp75	HSPA9	CST	3593	Rabbit	1:250
**184**	GSK-3alpha/beta	GSK-3a-b	GSK3A, B	Santa Cruz	sc-7291	Mouse	1:750
**185**	GSK-3alpha/beta (phospho S21/S9)	GSK-3a-b_pS21_S9	GSK3A, B	CST	9331	Rabbit	1:200
**186**	GSK-3B	GSK-3B	GSK3B	CST	9315	Rabbit	1:750
**187**	GSK-3beta (phospho S9)	GSK-3b_pS9	GSK3B	CST	5558	Rabbit	1:250
**188**	H2AX (phospho S140)	H2AX_pS140	H2AFX	Pierce Biotechnolog y	MA12022	Mouse	1:100
**189**	Hamartin/TSC1	TSC1	TSC1	CST	4906	Rabbit	1:200
**190**	HER2	HER2	ERBB2	Lab Vision	MS-325-P1	Mouse	1:300
**191**	HER2 (phospho Y1248)	HER2_pY1248	ERBB2	R&D systems	AF1768	Rabbit	1:1500
**192**	Heregulin	Heregulin	NRG1	CST	2573	Rabbit	01:30

**193**	HES1	HES1	HES1	CST	11988	Rabbit	1:500
**194**	Hexokinase II	Hexokinase II	HK2	CST	2106	Rabbit	1:100
**195**	Hif-1-alpha	Hif-1-alpha	HIF1A	BD Biosciences	610958	Mouse	1:20
**196**	Histone H3	Histone H3	HIST3H3	Abcam	ab1791	Rabbit	1:5000
**197**	HLA-DQA1	HLA-DQA1	HLA-DQA1	Abcam	ab128959	Rabbit	1:3000
**198**	HLA-DR/DP/DQ/DX	HLA-DR-DP-DQ-DX	HLA-DRA	Santa Cruz	sc-53302	Mouse	1:250
**199**	HMHA1	HMHA1	HMHA1	ProteinTech	14832-1-AP	Rabbit	1:3000
**200**	HSP27	HSP27	HSBP1	CST	2402	Mouse	1:75
**201**	HSP27 (phospho S82)	HSP27_pS82	HSBP1	CST	2401	Rabbit	1:75
**202**	HSP60	HSP60	HSP60	CST	12165	Rabbit	1:1000
**203**	HSP70	HSP70	HSPA1A	CST	4872	Rabbit	1:50
**204**	Hsp75/TRAP1	TRAP1	TRAP1	BD Biosciences	612344	Mouse	1:750
**205**	IDO	IDO	IDO1	CST	86630	Rabbit	1:200
**206**	IGF1R (phospho Y1135/Y1136	IGF1R_pY1135_Y1136	IGF1R, INSR	CST	3024	Rabbit	1:30
**207**	IGF-1Receptor beta	IGF1R-b	IGF1R	CST	3018	Rabbit	1:50
**208**	IGFBP2	IGFBP2	IGFBP2	CST	3922	Rabbit	1:50
**209**	IGFBP3	IGFBP3	IGFBP3	BD Biosciences	611504	Mouse	1:1000
**210**	IGFRb	IGFRb	IGF1R	CST	3027	Rabbit	1:250
**211**	IL-6	IL-6	IL6	CST	12153	Rabbit	1:250
**212**	INPP4b	INPP4b	INPP4B	CST	4039	Rabbit	01:30
**213**	Insulin Receptor beta	IR-b	INSR	CST	3025	Rabbit	1:100
**214**	IRF-1	IRF-1	IRF1	CST	8478	Rabbit	1:250
**215**	IRS1	IRS1	IRS1	Millipore	06-248	Rabbit	1:250
**216**	IRS2	IRS2	IRS2	CST	4502	Rabbit	1:100
**217**	JAB1	JAB1	COPS5	Santa Cruz	sc-13157	Mouse	1:30
**218**	Jagged1	Jagged1	JAG1	Abcam	ab109536	Rabbit	01:50
**219**	Jak2	Jak2	JAK2	CST	3230	Rabbit	1:750
**220**	JNK (phospho T183/Y185)	JNK_pT183_Y185	MAPK8	CST	4668	Rabbit	01:30
**221**	JNK2	JNK2	MAPK9	CST	4672	Rabbit	1:25
**222**	KAP1	KAP1	TRIM28	Abcam	ab10484	Rabbit	1:2000
**223**	KMT3A/HYPB/HIF-1	SETD2	SETD2	abcam	ab184190	Rabbit	1:1000

**224**	LAD1	LAD1	LAD1	Atlas	HPA028732	Rabbit	1:500
**225**	Lasu1/Ureb1	Lasu1	HUWE1	Bethyl	IHC-00439	Rabbit	1:1000
**226**	LC3A/B	LC3A-B	MAP1LC3A, B	CST	4108	Rabbit	1:250
**227**	Lck	Lck	LCK	CST	2752	Rabbit	1:75
**228**	LDHA	LDHA	LDHA	CST	3582	Rabbit	1:250
**229**	LRP6 (phospho S1490)	LRP6_pS1490	LRP6	CST	2568	Rabbit	1:250
**230**	MAPK (phospho T202/Y204)	MAPK_pT202/Y204	MAPK1, 3	CST	4377	Rabbit	1:25
**231**	Mcl-1	Mcl-1	MCL1	CST	5453	Rabbit	1:100
**232**	MDM2 (phospho S166)	MDM2_pS166	MDM2	CST	3521	Rabbit	1:60
**233**	MEK1	MEK1	MAP2K1	Epitomics/ Abcam	1235-1/ ab32576	Rabbit	1:1500
**234**	MEK1 (phospho S217/S221)	MEK1_p_S217/ S221	MAP2K1, 2	CST	9154	Rabbit	1:50
**235**	MEK2	MEK2	MAP2K2	CST	9125	Rabbit	1:50
**236**	MelanA	MelanA	MLANA	Abcam	ab51061	Rabbit	1:500
**237**	Melanoma gp100	Melan-gp100	PMEL	Abcam	ab137078	Rabbit	1:500
**238**	MERIT40	MERIT40	MERIT40	CST	12711	Rabbit	1:3000
**239**	MERIT40 (phospho S29)	MERIT40_pS29	BABAM1	CST	12110	Rabbit	1:300
**240**	Merlin/NF2	Merlin	NF2	Novus	22710002	Rabbit	1:250
**241**	MIF	MIF	MIF	Santa Cruz	sc-130329	Rabbit	1:100
**242**	MITF (D5G7V)	MITF	MITF	CST	12590	Rabbit	1:500
**243**	Mitofusin-1	Mitofusin-1	MFN1	CST	14739	Rabbit	1:500
**244**	Mitofusin-2	Mitofusin-2	MFN2	CST	11925	Rabbit	1:1000
**245**	MLH1 (4C9C7)	MLH1	MLH1	CST	3515	Mouse	1:500
**246**	MLKL	MLKL	MLKL	CST	14993	Rabbit	1:1000
**247**	MMP2	MMP2	MMP2	CST	4022	Rabbit	1:75
**248**	Mnk1	Mnk1	MKNK1	CST	2195	Rabbit	1:750
**249**	Monocarboxylic Acid Transporter 4	MCT4	SLC16A4	Millipore	AB3314P	Rabbit	1:500
**250**	MR1	MR1	MR1	Santa Cruz	sc-377312	Mouse	1:500
**251**	MRAP	MRAP	MRAP	Abcam	ab103319	Rabbit	1:500
**252**	MSH2 (D24B5)	MSH2	MSH2	CST	2017	Rabbit	1:750
**253**	MSH6	MSH6	MSH6	Novus	22030002	Rabbit	1:1000
**254**	MSI2 (EP1305Y)	MSI2	MSI2	Abcam	ab76148	Rabbit	1:1000

**255**	MTCO1	MTCO1	MTCO1	Abcam	ab14705	Mouse	1:500
**256**	mTOR	mTOR	MTOR	CST	2983	Rabbit	1:3000
**257**	mTOR (phospho S2448)	mTOR_pS2448	MTOR	CST	2971	Rabbit	1:50
**258**	MTSS1	MTSS1	MTSS1	Novus	H00009788-M01A	Mouse	1:250
**259**	Myosin Heavy Chain 11	Myosin-11	MYH11	Novus	21370002	Rabbit	1:1000
**260**	Myosin IIa	Myosin-IIa	MYH9	CST	3403	Rabbit	1:1000
**261**	Myosin IIa (phospho S1943)	Myosin-IIa_pS1943	MYH9	CST	5026	Rabbit	1:750
**262**	Myt1	Myt1	PKMYT1	CST	4282	Rabbit	1:100
**263**	NAPSIN-A	NAPSIN-A	NAPSA	Epitomics/ Abcam	5795-1/ ab129189	Rabbit	1:150
**264**	N-Cadherin	N-Cadherin	CDH2	CST	4061	Rabbit	1:25
**265**	NDRG1 (phospho T346)	NDRG1_pT346	NDRG1	CST	3217	Rabbit	01:50
**266**	NDUFB4	NDUFB4	NDUFB4	Abcam	ab110243	Mouse	1:25
**267**	NF-kB p65 (phospho S536)	NF-kB-p65_pS536	RELA	CST	3033	Rabbit	1:30
**268**	Notch1	Notch1	NOTCH1	CST	3268	Rabbit	01:30
**299**	Notch1 (Cleaved)	Notch1-cleaved	NOTCH1	CST	4147	Rabbit	1:100
**270**	Notch3	Notch3	NOTCH3	Novus	H00004854-M01	Mouse	1:250
**271**	NQO1	NQO1	NQO1	CST	3187	Mouse	1:15000
**272**	N-Ras	N-Ras	NRAS	Santa Cruz	sc-31	Mouse	1:50
**273**	NRF2	NRF2	NRF2	CST	12721	Rabbit	1:500
**274**	Oct-4	Oct-4	POU5F1	CST	2750	Rabbit	1:40
**275**	p16/INK4a	p16-INK4a	CDKN2A	Epitomics/ Abcam	1712-1/ ab40803	Rabbit	1:500
**276**	p21	p21	CDKN1A	Santa Cruz	sc-6246	Rabbit	1:150
**277**	p27 (phospho T157)	p27_pT157	CDKN1B	R&D Systems	AF1555	Rabbit	1:30
**278**	p27 (phospho T198)	p27_pT198	CDKN1B	Abcam	ab64949	Rabbit	01:50
**279**	p27 KIP 1	p27-Kip-1	CDKN1B	Epitomics/ Abcam	1591-1/ ab32034	Rabbit	01:40
**280**	p38 (phospho T180/Y182)	p38_pT180_Y182	MAPK11, 12, 13, 14	CST	9211	Rabbit	1:38
**281**	p38 alpha MAPK	p38-a	MAPK1	CST	9228	Mouse	1:300
**282**	p38 MAPK	p38-MAPK	MAPK11, 12, 14	CST	9212	Rabbit	1:1500

**283**	p38/MAPK (phospho T180/Y182)	p38-MAPK_pT180_Y182	MAPK14	CST	9215	Rabbit	1:250
**284**	p44/42 MAPK	p44-42-MAPK	MAPK1, 3	CST	4695	Rabbit	1:2000
**285**	p53	p53	TP53	CST	9282	Rabbit	1:2500
**286**	p70 S6 Kinase (phospho T389)	p70-S6K_pT389	RPS6KB1	CST	9205	Rabbit	1:50
**287**	p70/S6K1	p70-S6K1	RPS6KB1	Epitomics/ Abcam	1494-1/ ab32529	Rabbit	1:300
**288**	p90RSK (phospho T573)	p90RSK_pT573	RPS6K	CST	9346	Rabbit	1:25
**289**	PAI-1	PAI-1	SERPINE1	BD Biosciences	612024	Mouse	1:50
**290**	PAICS	PAICS	PAICS	Sigma-Aldrich	HPA035895	Rabbit	1:250
**291**	PAK1	PAK1	PAK1	CST	2602	Rabbit	1:750
**292**	PAK4	PAK4	PAK4	CST	3242	Rabbit	1:300
**293**	PAR	PAR	PAR	Trevigen	4336-BPC-100	Rabbit	1:30000
**294**	PARG	PARG	PARG	CST	66564	Rabbit	1:1000
**295**	PARK7/DJ1	DJ1	PARK7	Abcam	ab76008	Rabbit	1:5000
**296**	PARP	PARP	PARP1	CST	9532	Rabbit	1:1000
**297**	Patched	Patched	PTCH1	Abcam	ab53715	Rabbit	1:1000
**298**	Paxillin	Paxillin	PXN	CST	2542	Rabbit	1:250
**299**	P-Cadherin	P-Cadherin	CDH3	CST	2130	Rabbit	1:38
**300**	PCNA	PCNA	PCNA	CST	2586	Mouse	1:250
**301**	PD-1	PD-1	PDCD1	CST	43248	Mouse	1:500
**302**	Pdcd4	Pdcd4	PDCD4	Rockland	600-401-965	Rabbit	1:750
**303**	PDGFRB	PDGFR-b	PDGFRB	Invitrogen	MA5-15143	Rabbit	1:500
**304**	PDH	PDH	PDH	Abcam	ab110332	Mouse	1:100
**305**	PDHK1	PDHK1	PDHK1	CST	3820	Rabbit	1:300
**306**	PDK1	PDK1	PDPK1	CST	3062	Rabbit	01:50
**307**	PDK1 (phospho S241)	PDK1_pS241	PDPK1	CST	3061	Rabbit	01:50
**308**	PD-L1	PD-L1	CD274	CST	13684	Rabbit	1:250
**309**	PEA-15	PEA-15	PEA15	CST	2780S	Rabbit	1:100
**310**	PED/PEA-15 (phospho S116)	PEA-15_pS116	PEA15	Life Technologies	44836G	Rabbit	1:100
**311**	PHGDH	PHGDH	PHGDH	CST	13428	Rabbit	1:1000
**312**	PI3 Kinase p110 alpha	PI3K-p110-a	PIK3CA	CST	4255	Rabbit	1:50
**313**	PI3K p110 beta	PI3K-p110-b	PIK3BC	Santa Cruz	sc-376412	Mouse	1:40

**314**	PI3K p85	PI3K-p85	PIK3R1	Millipore	06-195	Rabbit	1:15000
**315**	PKA RI alpha	PKA-a	PRKAR1A	CST	5675	Rabbit	1:250
**316**	PKC alpha/beta II (phospho T638/641)	PKC-a-b-II_pT638_T641	PRKCA, B	CST	9375	Rabbit	1:1000
**317**	PKC(pan) beta II (phospho S660)	PKC-b-II_pS660	PRKCA, B, D, E, H, Q	CST	9371	Rabbit	1:200
**318**	PKC delta (phospho S664)	PKC-delta_pS664	PRKCD	Millipore	07-875	Rabbit	1:75
**319**	PKCalpha	PKCa	PRKCA	CST	2056	Rabbit	1:200
**320**	PKM2	PKM2	PKM	CST	4053	Rabbit	1:300
**321**	PLC gamma2 (phospho Y759)	PLC-gamma2_pY759	PLCG2	CST	3874	Rabbit	01:25
**322**	PLK1	PLK1	PLK1	CST	4513	Rabbit	1:125
**323**	Met (phospho Y1234/Y1235)	c-Met_pY1234_Y1235	MET	CST	3129	Rabbit	1:100
**324**	PMS2	PMS2	PMS2	Novus Biologicals	22510002	Rabbit	1:1500
**325**	PRAS40	PRAS40	AKT1S1	Life Technologies	AHO1031	Mouse	1:75
**326**	PRAS40 (phospho T246)	PRAS40_pT246	AKT1S1	Life Technologies	441100G	Rabbit	1:500
**327**	PREX1	PREX1	PREX1	Abcam	ab102739	Rabbit	1:100
**328**	Progesterone Receptor [YR85]	PR	PGR	abcam	206926	Rabbit	1:500
**329**	PTEN	PTEN	PTEN	CST	9552	Rabbit	1:500
**330**	PTPN12	PTPN12	PTPN12	Abcam	ab76942	Rabbit	1:500
**331**	Puma	Puma	BBC3	CST	4976	Rabbit	1:50
**332**	PYGB	PYGB	PYGB	Sigma-Aldrich	SAB2900066	Rabbit	1:750
**333**	PYGM	PYGM	PYGM	Novus	H00005837-M10	Mouse	1:500
**334**	Pyk2 (phospho Y402)	Pyk2_pY402	PYK2	CST	3291	Rabbit	1:500
**335**	Pyruvate Dehydrogenase	PDHA1	PDHA1	CST	3205	Rabbit	1:200
**336**	Rab11	Rab11	RAB11A, B	CST	3539	Rabbit	1:30
**337**	Rab25	Rab25	RAB25	CST	4314	Rabbit	1:30
**338**	Rac1/Cdc42	Cdc42	CDC42	CST	4651	Rabbit	1:100
**339**	Rad23A	Rad23A	RAD23A	CST	24555	Rabbit	1:1000
**340**	Rad50	Rad50	RAD50	CST	3427	Rabbit	1:250
**341**	Rad51	Rad51	RAD51	Millipore	ABE257	Rabbit	1:1000

**342**	Raptor	Raptor	RPTOR	CST	2280	Rabbit	1:300
**343**	Rb	Rb	RB1	CST	9309	Mouse	1:150
**344**	Rb (phospho S807/811)	Rb_pS807_S811	RB1	CST	9308	Rabbit	1:1000
**345**	RBM15	RBM15	RBM15	Novus	21390002	Rabbit	1:5000
**346**	Rheb	Rheb	RHEB	R&D Systems	MAB3426	Mouse	1:75
**347**	Rictor	Rictor	RICTOR	CST	2114	Rabbit	1:100
**348**	Rictor (phospho T1135)	Rictor_pT1135	RICTOR	CST	3806	Rabbit	1:200
**349**	RIP	RIP	RIP	CST	4926	Rabbit	1:75
**350**	RIP3	RIP3	RIP3	CST	13526	Rabbit	1:500
**351**	RPA32 (phospho S4/S8)	RPA32_pS4/S8	RPA2	Bethyl	A300-245A	Rabbit	1:250
**352**	RPA32/RPA2	RPA32	RPA2	CST	2208	Rat	1:150
**353**	RRM1	RRM1	RRM1	CST	3388	Rabbit	1:100
**354**	RRM2	RRM2	RRM2	Life Technologies	PA527856	Rabbit	1:250
**355**	RSK	RSK	RPS6KA1, 2, 3	CST	9347	Rabbit	1:150
**356**	S100A4	S100A4	S100A4	CST	13018	Rabbit	1:1000
**357**	S6 (phospho S235/236)	S6_pS235_S236	RPS6	CST	2211	Rabbit	1:2500
**358**	S6 (phospho S240/244)	S6_pS240_S244	RPS6	CST	2215	Rabbit	1:1000
**359**	S6 Ribosomal Protein	S6	RPS6	CST	2317	Mouse	1:750
**360**	SCD	SCD	SCD	Santa Cruz	sc-58420	Mouse	1:20
**361**	SDHA	SDHA	SDHA	CST	11998	Rabbit	1:250
**362**	SFRP1	SFRP1	SFRP1	CST	4690	Rabbit	1:500
**363**	Shc_pY317	Shc_pY317	SHC1	CST	2431	Rabbit	01:25
**364**	SHP-2 (phospho Y542)	SHP-2_pY542	PTPN11	CST	3751	Rabbit	1:75
**365**	SHP2 / PTPN11	SHP2	PTPN11	CST	3397	Rabbit	1:250
**366**	SLC1A5	SLC1A5	SLC1A5	Sigma-Aldrich	HPA035240	Rabbit	1:150000
**367**	Slfn11	Slfn11	SLFN11	Santa Cruz	sc-374339	Mouse	1:150
**368**	Smac	Smac	DIABLO	CST	2954	Mouse	1:150
**369**	Smad1	Smad1	SMAD1	Epitomics/ Abcam	1649-1/ ab33902	Rabbit	1:500
**370**	Smad3	Smad3	SMAD3	Epitomics/ Abcam	1735-1/ ab40854	Rabbit	1:150
**371**	Smad4	Smad4	SMAD4	Santa Cruz	sc-7966	Mouse	1:30

**372**	Snail	Snail	SNAI1	CST	3895	Mouse	1:50
**373**	SOD1	SOD1	SOD1	CST	4266	Mouse	1:500
**374**	SOD2 (D9V9C)	SOD2	SOD2	CST	13194	Rabbit	1:200
**375**	Sox2	Sox2	SOX2	CST	2748	Rabbit	1:50
**376**	Src	Src	SRC	Millipore	05-184	Mouse	1:50
**377**	Src (phospho Y416)	Src_pY419	SRC	CST	2101	Rabbit	1:25
**378**	Src (phospho Y527)	Src_pY527	SRC	CST	2105	Rabbit	1:150
**379**	SRSF1/SF2	SF2	SRSF1	Invitrogen	324500	Mouse	1:75
**380**	Stat3	Stat3	STAT3	CST	4904	Rabbit	1:3000
**381**	Stat3 (phospho Y705)	Stat3_pY705	STAT3	CST	9145	Rabbit	1:100
**382**	Stat5a	Stat5a	STAT5A	Epitomics/ Abcam	1289-1/ ab32043	Rabbit	1:300
**383**	Stathmin-1	Stathmin-1	STMN1	Epitomics/ Abcam	1972-1/ ab52630	Rabbit	1:75
**384**	STING	STING	TMEM173	CST	13647	Rabbit	1:250
**385**	Syk	Syk	SYK	Santa Cruz	sc-1240	Mouse	1:500
**386**	Tau	Tau	MAPT	Millipore	05-348	Mouse	1:100
**387**	TAZ	TAZ	WWTR1	CST	4883	Rabbit	1:300
**388**	TFAM	TFAM	TFAM	CST	7495	Rabbit	1:300
**389**	Transferrin R	TFRC	TFRC	Novus	22500002	Rabbit	1:15000
**390**	TIGAR	TIGAR	TIGAR	Epitomics/ Abcam	S1711/ ab137573	Rabbit	1:100
**391**	Transglutaminase	Transglutaminase	TGM2	Lab Vision	MS-224-P1	Mouse	1:150
**392**	TRIM25	TRIM25	TRIM25	Abcam	ab167154	Rabbit	1:3000
**393**	TTF1	TTF1	NKX2-1	Epitomics/ Abcam	2044-1/ ab76013	Rabbit	1:150
**394**	Tuberin	Tuberin	TSC2	Epitomics/ Abcam	1613-1/ ab32554	Rabbit	1:2500
**395**	Tuberin/TSC2 (phospho T1462)	Tuberin_pT1462	TSC2	CST	3617	Rabbit	1:38
**396**	TUFM	TUFM	TUFM	Abcam	ab173300	Rabbit	1:38
**397**	TWEAK Receptor/FN14	FN14	TNFRSF12A	CST	4403	Rabbit	1:1000
**398**	TWIST	TWIST	TWIST1	Santa Cruz	sc-81417	Mouse	1:30
**399**	Tyro3	Tyro3	TYRO3	CST	5585	Rabbit	1:30
**400**	UBAC1	UBAC1	UBAC1	Sigma-Aldrich	HPA005651	Rabbit	1:250
**401**	Ubiquityl-Histone H2B	U-Histone-H2B	HIST1H2BB	CST	5546	Rabbit	1:500

**402**	UGT1A	UGT1A	UGT1A1, 3, 4, 5, 7, 8, 10	Santa Cruz	sc-271268	Mouse	1:75
**403**	ULK1 (phospho S757)	ULK1_pS757	ULK1	CST	6888	Rabbit	1:300
**404**	UQCRC2	UQCRC2	UQCRC2	MitoSciences / Abcam	MS304/ ab14745	Mouse	01:50
**405**	UVRAG	UVRAG	UVRAG	CST	13115	Rabbit	1:100
**406**	VASP	VASP	VASP	CST	3112	Rabbit	1:100
**407**	Vav1	Vav1	VAV1	CST	2502	Rabbit	1:500
**408**	VDAC1/Porin	Porin	VDAC1	Abcam	ab14734	Mouse	1:100
**409**	VEGF Receptor 2	VEGFR-2	KDR	CST	2479	Rabbit	1:3000
**410**	VHL/EPPK1	VHL-EPPK1	EPPK1	BD Biosciences	556347	Mouse	1:1500
**411**	Vimentin	Vimentin	VIM	Dako/Fisher	M0725/ MS-129-P	Mouse	1:250
**412**	Vinculin	Vinculin	VCL	Sigma-Aldrich	SAB4200080	Mouse	1:25000
**413**	Wee1	Wee1	WEE1	CST	4936	Rabbit	1:250
**414**	Wee1 (phospho S642)	Wee1_pS642	WEE1	CST	4910	Rabbit	1:50
**415**	WIPI1	WIPI1	WIPI1	CST	12124	Rabbit	1:150
**416**	WIPI2	WIPI2	WIPI2	CST	8567	Rabbit	1:150
**417**	XBP-1	XBP-1	XBP1	Santa Cruz	sc-32136	Goat	1:200
**418**	XIAP	XIAP	XIAP	CST	2042	Rabbit	1:100
**419**	XPA	XPA	XPA	Santa Cruz	sc-56813	Mouse	1:75
**420**	XPF	XPF	ERCC4	Abcam	ab73720	Rabbit	1:100
**421**	XPG	ERCC5	ERCC5	Proteintech Group	11331-1-AP	Rabbit	1:250
**422**	XRCC1	XRCC1	XRCC1	CST	2735	Rabbit	1:20
**423**	YAP	YAP	YAP1	Santa Cruz	sc-376830	Mouse	1:300
**424**	YAP (phospho S127)	YAP_pS127	YAP1	CST	4911	Rabbit	1:250
**425**	YB1 (phospho S102)	YB1_pS102	YBX1	CST	2900	Rabbit	1:50
**426**	ZAP-70	ZAP-70	ZAP70	CST	2705	Rabbit	1:500
