# Supplementary material for: Modeling Heterogeneity of Triple‐Negative Breast Cancer Uncovers a Novel Combinatorial Treatment Overcoming Primary Drug Resistance
Source: Adv Sci (Weinh). 2020 Dec 16;8(3):2003049. doi: 10.1002/advs.202003049 (PMC7856896; doi:10.1002/advs.202003049)
Supplement: Supplementary file 7 — Supplemental Table 6 [file ADVS-8-2003049-s007.pdf]

Table S6. Comparisons of RPPA outcomes of *MMTV-R26* ^{Met} cells belonging to "subtype A" and "subtype B" versus those from the non-tumorigenic cells.

Subtype A (MGT4+9+11) vs. non-tumorigenic (MGT2 & 7)

Protein	logFC	AveExpr	t	P.Value	adj.P.Val
P-Met_pY1234_Y1235	3,771668	-1,0119078	10,90563	6,84E-08	5,77E-06
E-Cadherin	3,468199	1,5253849	10,61925	9,35E-08	5,77E-06
Claudin-7	1,911199	1,0822876	10,33591	1,28E-07	6,33E-06
RSK	1,524384	-0,6111489	7,299833	6,18E-06	1,53E-04
CDK1	1,448314	-0,1166139	4,725375	4,02E-04	0,00183884
ARID1A	1,446252	0,3963761	4,852469	3,20E-04	0,00155111
Bim	1,416402	0,0453894	4,942895	2,73E-04	0,00142332
53BP1	1,269014	0,5378448	5,401039	1,23E-04	7,89E-04
Rad50	1,061401	0,2447965	5,410073	1,21E-04	7,89E-04
HES1	0,981841	0,0343482	2,795423	0,0152226	0,03357122
Src_pY416	0,958743	-0,2971786	6,696275	1,52E-05	2,50E-04
ACC_pS79	0,939118	-0,11619	2,665268	0,0195073	0,04054706
Connexin-43	0,895784	0,2361452	3,028428	0,0097416	0,02382356
FASN	0,887145	-0,1825017	5,620712	8,49E-05	7,23E-04
Src_pY527	0,883245	-0,1438668	4,22756	9,98E-04	0,00347248
Rb_pS807_S811	0,870722	-0,0627291	2,38951	0,0328075	0,06157929
ACC1	0,865206	0,0651942	4,657714	4,54E-04	0,00196783
Cox2	0,828262	0,6923661	1,422211	0,1786289	0,24648788
4E-BP1_pS65	0,794498	0,2176293	6,006903	4,49E-05	5,28E-04
GCN5L2	0,78403	0,1609002	5,658902	7,96E-05	7,23E-04
Akt_pS473	0,706044	-0,29258	4,279576	9,06E-04	0,00334177
c-Jun_pS73	0,687669	0,1712913	6,835796	1,23E-05	2,17E-04
p90RSK_pT573	0,660934	-0,1175991	5,401963	1,23E-04	7,89E-04
PARP	0,639323	0,189449	6,210382	3,24E-05	4,44E-04
Akt_pT308	0,631021	-0,1776652	3,715662	0,0026124	0,00750295
HER3	0,584665	0,4124621	4,245167	9,66E-04	0,00340895
C-Raf_pS338	0,560357	-6,44E-04	5,077137	2,15E-04	0,0011679
PKC-b-II_pS660	0,557357	-0,279136	5,524983	9,97E-05	7,73E-04
IRS1	0,543443	0,197673	1,828737	0,0905815	0,1399653
VASP	0,536384	-0,1346535	3,548721	0,0035909	0,00996573
c-Myc	0,536074	0,1818384	5,782582	6,48E-05	6,96E-04
beta-Catenin	0,520031	0,2023753	3,444927	0,0043796	0,01163174
eIF4E	0,500608	-0,0795851	7,337273	5,85E-06	1,53E-04
4E-BP1	0,49076	0,0226499	2,387859	0,0329088	0,06157929
Notch1	0,475117	-0,2848443	2,481032	0,027637	0,0541772
p16_INK4a	0,460764	0,1754814	2,047757	0,061451	0,10328628
Caspase-3	0,432888	0,0986794	7,15535	7,63E-06	1,63E-04
RPA32_pS4_S8	0,422635	-0,0877175	4,922764	2,83E-04	0,00142496
NDRG1_pT346	0,421693	0,3033009	0,904163	0,3824397	0,47708386
RBM15	0,419121	0,4528309	1,104033	0,2896937	0,37859436
Paxillin	0,399942	-0,0701099	8,728997	8,82E-07	3,63E-05
PAICS	0,395387	0,2794241	2,228145	0,0442483	0,07751302
Bcl-xL	0,392312	0,1383302	4,062771	0,001357	0,00424273
14-3-3_zeta	0,388742	-0,0596922	4,658633	4,53E-04	0,00196783
Bid	0,381066	0,1070465	3,283919	0,0059639	0,01534459
HER2_pY1248	0,3781	0,0388001	2,664526	0,0195348	0,04054706
FAK	0,377805	-0,0713128	5,6286	8,38E-05	7,23E-04
Histone-H3	0,376417	0,0086542	3,004335	0,0102027	0,02470651
p38_pT180_Y182	0,363108	0,0857146	5,278316	1,52E-04	9,11E-04
Shc_pY317	0,352321	-0,2506858	4,352649	7,92E-04	0,00305704
c-Kit	0,345628	0,1667719	5,504168	1,03E-04	7,73E-04
AMPK_alpha	0,343258	-0,1949831	3,730602	0,0025393	0,00737877
PRAS40_pT246	0,333433	-0,1478686	1,602546	0,1331629	0,19578124
Smad3	0,32264	0,1278642	4,631279	4,76E-04	0,00202851
HSP70	0,316225	0,0233271	5,187279	1,78E-04	0,00102115
WIPI1	0,299004	0,2067124	1,857936	0,0860821	0,13542849
PDK1	0,288736	-0,0743783	4,052317	0,0013838	0,00427252
PAK1	0,288257	-0,0960324	1,206203	0,2493386	0,3329007
Bax	0,282279	-0,0402982	0,891662	0,3888601	0,48265551
DJ-1	0,28216	0,1007253	4,336833	8,16E-04	0,00309901
IR-b	0,271336	0,0634504	1,320554	0,2095391	0,28437448
Lck	0,270305	0,0645107	2,784147	0,0155539	0,0339984
Merlin	0,25649	0,0583102	2,001088	0,0668193	0,11002916
TUFM	0,255489	0,0967286	3,668656	0,0028567	0,00811037
Myt1	0,251577	-0,0590917	3,421506	0,0045805	0,01203606
PDK1_pS241	0,24966	0,0705303	1,828203	0,0906658	0,1399653
ATM	0,248594	0,0743071	1,061881	0,3077141	0,40002836
ER-alpha_pS118	0,247404	0,1571599	4,543131	5,59E-04	0,00222667
Bad_pS112	0,246678	-0,1849946	3,61229	0,0031806	0,00892728
Caspase-7_cleavedD19	0,233133	-0,1285137	2,305651	0,0383474	0,06814244
Bak	0,22832	-0,0479864	3,170981	0,0074085	0,01848373
Chk2_pT68	0,22803	-0,034172	2,891423	0,0126689	0,02897413
Wee1_pS642	0,223293	0,1465578	2,56915	0,0234061	0,04700253
Elk1_pS383	0,217788	-0,0541057	4,129958	0,0011969	0,00394175
Cdc2_pY15	0,208341	0,0433277	1,556484	0,1437134	0,20758604
b-Actin	0,207393	0,1279735	1,793327	0,0963213	0,14686021
EGFR_pY1173	0,201168	-0,0426149	4,551783	5,50E-04	0,00222667
STING	0,200475	0,1379571	0,41888	0,6821762	0,75222104
ATR_pS428	0,192485	0,00212	3,993173	0,0015462	0,00465732

Subtype B (MGT13) vs. non-tumorigenic (MGT2 & 7)

Protein	logFC	AveExpr	t	P.Value	adj.P.Val
P-Met_pY1234_Y1235	4,563148	-1,6674278	44,05051	1,05E-09	2,60E-07
RSK	2,114524	-0,7602672	6,313678	4,25E-04	0,00656047
Notch1	2,029212	0,2558908	10,54554	1,68E-05	5,19E-04
HES1	1,627067	0,0519271	6,696406	2,98E-04	0,0056618
Fibronectin	1,565388	0,9234731	10,57193	1,65E-05	5,19E-04
ACC_pS79	1,380298	-0,174792	3,366794	0,0122547	0,05132152
CDK1	1,366848	-0,5335007	3,823602	0,0067068	0,03524618
Rb_pS807_S811	1,304012	-0,1066745	2,532854	0,0395758	0,11217854
Akt_pS473	1,265382	-0,2390729	11,70825	8,45E-06	3,64E-04
IGFRb	1,122679	0,5041849	4,190776	0,004226	0,03070037
Bim	1,058487	-0,4815593	7,109478	2,07E-04	0,00451158
Src_pY416	1,017153	-0,5250009	18,32814	4,21E-07	5,08E-05
Akt_pT308	0,99835	-0,1866618	7,260346	1,82E-04	0,00448387
PKA-a	0,965586	0,2854551	17,31854	6,17E-07	5,08E-05
ACC1	0,852846	-0,1682892	3,899131	0,0060888	0,03379328
FASN	0,838035	-0,4375213	3,49291	0,0103442	0,0464548
LRP6_pS1490	0,671783	0,1657914	11,62867	8,84E-06	3,64E-04
p90RSK_pT573	0,607544	-0,3147928	3,325557	0,0129593	0,05334913
Shc_pY317	0,593491	-0,2402474	6,355699	4,08E-04	0,00656047
AMPK_pT172	0,585846	0,8578037	3,89801	0,0060975	0,03379328
Pdcd4	0,584895	0,116794	3,924916	0,0058923	0,03379328
PKC-b-II_pS660	0,563848	-0,4233496	5,389217	0,0010738	0,01386302
STING	0,544972	0,2327264	3,055622	0,0187941	0,06981802
MCT4	0,534748	0,5135805	0,88172	0,4076264	0,52833899
AMPK_alpha	0,481	-0,2264803	4,409915	0,003239	0,02424311
PRAS40_pT246	0,470494	-0,177067	8,623494	6,16E-05	0,00169047
IR-b	0,453404	0,0699186	1,657671	0,1420237	0,25420179
STAT5-alpha	0,451643	0,587122	2,638098	0,0339911	0,10494747
HER2_pY1248	0,445391	-0,0320792	4,41123	0,0032339	0,02424311
Tuberin	0,432484	0,1376954	4,905013	0,0018226	0,01911688
SLC1A5	0,412054	0,3457002	2,460839	0,0439391	0,11545689
Src_pY527	0,410403	-0,5794568	1,690426	0,1354647	0,24602776
Paxillin	0,406428	-0,1728096	7,041667	2,19E-04	0,00451158
Bad_pS112	0,397009	-0,1856245	4,420566	0,0031979	0,02424311
Caspase-7_cleavedD198	0,371346	-0,130765	3,639518	0,0085192	0,04125948
XRCC1	0,366656	0,0387943	3,039023	0,0192347	0,06986708
IGF1R_pY1135_Y1136	0,348902	0,1505626	3,239624	0,0145717	0,05900362
Bid	0,345436	-0,0087244	2,510036	0,0409076	0,11226863
Mcl-1	0,334732	0,3562144	2,893978	0,0235855	0,08205113
RIP	0,325051	0,1058554	1,440921	0,193453	0,30269376
ATM	0,31833	0,0386307	0,832604	0,4329695	0,55411127
COG3	0,306351	0,0145178	4,422202	0,0031917	0,02424311
14-3-3_zeta	0,305893	-0,197724	5,347493	0,0011225	0,01386302
eIF4E	0,300825	-0,2972351	4,907438	0,0018176	0,01911688
WIPI1	0,299748	0,1281728	3,925854	0,0058853	0,03379328
mTOR	0,29646	-0,0810146	4,04117	0,0050885	0,03307519
MAPK_pT202_Y204	0,294888	-0,0230378	2,702394	0,0309894	0,09801214
Chk2_pT68	0,285975	-0,0694781	3,509896	0,0101125	0,04625544
PAK1	0,283941	-0,1739056	1,541461	0,1677734	0,28383578
Wee1	0,282046	0,180511	1,947076	0,0932163	0,18604001
p21	0,275143	-0,1092629	3,873219	0,0062935	0,03379328
Myt1	0,267636	-0,1185596	3,748261	0,007392	0,03651662
FAK	0,266835	-0,2185122	3,366532	0,012259	0,05132152
YB1_pS102	0,248129	-0,0819382	3,803376	0,0068837	0,03529816
Caspase-3	0,244179	-0,0963645	4,070668	0,0049042	0,03273887
4E-BP1_pS65	0,236739	-0,2309479	1,380441	0,2105612	0,32505382
PLC-gamma2_pY759	0,23491	0,0072565	2,118022	0,0725491	0,1585807
Bak	0,215446	-0,11372	2,249705	0,0598096	0,14342683
UBAC1	0,214845	-0,092247	3,790107	0,0070025	0,03529816
Bax	0,208181	-0,146502	2,8076	0,026662	0,08780673
GATA6	0,201924	-0,0159812	2,874659	0,0242395	0,08315488
Cdc2_pY15	0,197787	-0,0161427	0,987127	0,3569608	0,49533325
PI3K-p85	0,197071	0,0344405	0,548609	0,6005859	0,70571753
GCN5L2	0,19554	-0,298087	0,966581	0,3664395	0,50283648
PARP	0,19536	-0,1694338	1,365077	0,2151167	0,32798665
Histone-H3	0,194263	-0,1686865	2,039951	0,081353	0,16967817
Stathmin	0,191596	-0,1444441	2,518617	0,0404014	0,11217854
ERCC5	0,189449	0,0877916	0,905446	0,3957795	0,52557809
beta-Catenin	0,171947	-0,083955	1,057484	0,3259429	0,46268912
EGFR_pY1173	0,169934	-0,1090561	2,744401	0,0291791	0,09360055
Elk1_pS383	0,169645	-0,1321767	2,395789	0,0483065	0,12300738
SDHA	0,16948	0,1233133	2,017755	0,0840447	0,17156231
PTEN	0,164032	0,2496951	0,94282	0,3776406	0,5125122
Connexin-43	0,158935	-0,3158979	0,379397	0,7158123	0,78931092
mTOR_pS2448	0,150508	-0,0301328	2,20943	0,0634461	0,14779565
Lck	0,150488	-0,0581287	1,887569	0,1016952	0,19935492
ATR_pS428	0,150049	-0,066832	2,208924	0,0634932	0,14779565
FOXO3a_pS318_S321	0,146765	-0,08466	2,59818	0,0360061	0,10715061
Tyro3	0,14106	-0,0157264	2,036651	0,0817478	0,16967817

DM-K9-Histone-H3	0,191106	-0,032935	2,955169	0,0112119	0,02688678
eEF2	0,187505	0,0403709	1,234271	0,2390563	0,32090713
Rictor	0,187175	-0,0121124	2,449399	0,0293286	0,05615625
eIF4G	0,186366	0,0353867	3,198586	0,0070258	0,017708
EGFR	0,182966	-0,0770142	2,536964	0,0248733	0,04954598
MSH6	0,182251	0,1297574	1,5399	0,1476837	0,21208063
MEK1_pS217_S221	0,17823	0,0065781	3,843738	0,0020492	0,00602553
XRCC1	0,173953	0,002085	1,931898	0,0755753	0,12362311
p27_pT198	0,17211	0,0723343	2,328813	0,0367341	0,06622864
Gab2	0,171561	0,0479602	0,624194	0,5433525	0,62422358
NAPSIN-A	0,170103	-0,1065433	2,9256	0,0118658	0,02764966
UBAC1	0,166895	-0,0687808	2,357465	0,0348277	0,06419738
Jagged1	0,158894	0,1041255	0,993534	0,3386726	0,43119655
TAZ	0,153525	0,1660362	2,347866	0,0354557	0,06439373
p53	0,147901	-0,0309641	1,44288	0,172831	0,24118219
Notch3	0,144246	0,0288049	2,942955	0,0114776	0,02699964
PAR	0,143159	0,7936152	0,264154	0,7958228	0,84364046
Stathmin	0,139431	-0,1300271	1,902231	0,079641	0,12857082
mTOR	0,136343	-0,1136771	1,652966	0,1223894	0,18321324
Aurora-B	0,133051	-0,0498797	1,806192	0,0941993	0,14451695
YB1_pS102	0,130054	-0,0982415	1,779569	0,0986378	0,14946961
PLC-gamma2_pY759	0,119652	-0,0105832	1,610908	0,131321	0,19539936
AMPK-a2_pS345	0,115932	-0,1005424	1,507819	0,1556308	0,22220123
Tuberin	0,115824	0,0325309	1,403352	0,1840597	0,25117539
Tyro3	0,092494	-0,0121462	1,858878	0,0859403	0,13542849
p27-Kip-1	0,091811	-0,0645913	2,350207	0,0353015	0,06439373
FOXO3a_pS318_S321	0,088969	-0,0859653	2,14559	0,0514697	0,08828487
Cyclin_D1	0,087443	-0,0020169	1,432363	0,175761	0,24389313
PEA15_pS116	0,085991	-0,0163314	0,631631	0,5386309	0,62422358
TIGAR	0,084525	0,0411889	0,976319	0,3468127	0,43929614
b-Catenin_pT41_S45	0,079037	0,0461708	1,01903	0,3268706	0,41832662
Chk1_pS296	0,071257	-0,0518607	1,16746	0,264095	0,34883135
GCLM	0,062402	-0,0037227	1,236505	0,2382525	0,32090713
Raptor	0,049775	0,0118633	0,593726	0,5629346	0,64075962
MDM2_pS166	0,04399	-0,1730021	0,553736	0,5892041	0,66758449
Akt	0,039874	-0,0891319	0,293054	0,7741296	0,82774897
DUSP4	0,037353	0,0838537	0,318001	0,7555598	0,81494878
MAPK_pT202_Y204	0,034884	-0,1252682	0,241066	0,8132833	0,85481267
Rab11	0,032688	-0,048905	0,381195	0,7092465	0,77514998
C-Raf	0,030052	-0,0492449	0,301115	0,7681125	0,82488607
Sox2	0,028709	0,2078454	0,150345	0,8828119	0,90329557
NF-kB-p65_pS536	0,027659	0,0087904	0,245802	0,8096926	0,85467557
YAP	0,026607	-0,0505988	0,45738	0,654985	0,72874462
cIAP	0,014036	-0,0504367	0,346067	0,7348567	0,79609472
Ets-1	0,006433	0,0497873	0,147502	0,8850102	0,90329557
Wee1	0,005595	0,0635073	0,032864	0,9742851	0,97428513
IGF1R_pY1135_Y1136	0,003058	0,0031501	0,042925	0,9664169	0,9703454
eIF4E_pS209	-0,0069	0,0259484	-0,10684	0,9165562	0,92403831
U-Histone-H2B	-0,01159	-0,0096519	-0,15162	0,8818259	0,90329557
JNK2	-0,01277	0,0018004	-0,1781	0,8614012	0,8939752
A-Raf	-0,01623	0,0166796	-0,14277	0,8886742	0,9033026
XPF	-0,02234	-0,1081235	-0,39114	0,7020631	0,7707093
HER3_pY1289	-0,0246	-0,0026466	-0,26741	0,7933662	0,84364046
PI3K-p110-a	-0,02523	-0,036344	-0,46261	0,6513318	0,72874462
eEF2K	-0,02755	0,178513	-0,16833	0,8689247	0,89801002
PI3K-p85	-0,02841	-0,069686	-0,13565	0,8941876	0,90518167
D-a-Tubulin	-0,03121	-0,0398156	-0,53242	0,6034589	0,68061341
SLC1A5	-0,03636	0,143932	-0,19651	0,8472655	0,88301506
RIP	-0,03699	-0,0590641	-0,21562	0,8326489	0,87145884
MMP2	-0,03721	0,055784	-0,96445	0,3525077	0,44423161
PEA15	-0,03872	0,1718814	-0,35493	0,7283627	0,79253564
PKA-a	-0,04	-0,1560579	-0,45884	0,6539663	0,72874462
LRP6_pS1490	-0,04168	-0,1509694	-0,71962	0,4845572	0,58099818
mTOR_pS2448	-0,04959	-0,1289684	-0,42059	0,6809608	0,75222104
p21	-0,05003	-0,2618167	-0,69608	0,4986958	0,58824698
JNK_pT183_pY185	-0,05417	0,0593339	-0,62554	0,5424947	0,62422358
Slfn11	-0,05446	-0,0775038	-0,82271	0,425579	0,52038616
Smad1	-0,05639	0,0367162	-0,69372	0,500129	0,58824698
Rad51	-0,06129	0,2265507	-0,6108	0,5519147	0,6311247
Glutamate-D1-2	-0,06848	0,0224619	-0,73836	0,4734825	0,57048871
c-Abl	-0,07024	-0,0653876	-1,56494	0,1417235	0,20591584
IGFBP2	-0,07332	-0,0240207	-0,64764	0,528544	0,61580364
p70-S6K1	-0,07489	0,1299386	-0,82936	0,4219433	0,51850749
IRF-1	-0,07708	0,0617817	-1,4848	0,161554	0,22802199
PREX1	-0,0774	0,2077197	-1,05361	0,3113465	0,40263137
p38_MAPK	-0,09257	0,0649005	-0,80506	0,4353339	0,52969197
Creb	-0,09465	0,0546613	-2,20983	0,0457627	0,07960132
PKC-delta_pS664	-0,10128	-0,0486037	-2,32125	0,0372539	0,06667902
ERCC5	-0,10161	-0,0637429	-0,77106	0,4545252	0,55033195
14-3-3_beta	-0,10446	-0,0199976	-2,04436	0,0618276	0,10328628
GSK-3a-b_pS21_S9	-0,12418	0,0017529	-0,68567	0,505028	0,59119387
Mnk1	-0,12863	0,1504279	-1,1942	0,2538398	0,33708831
Pdcd4	-0,13265	-0,2257078	-0,91752	0,3756589	0,47100381
VEGFR-2	-0,13295	0,0340055	-0,86012	0,4053808	0,50064526

S6_pS240_S244	0,127725	0,4220785	1,371766	0,2131229	0,32696494
ARID1A	0,115095	-0,5555488	0,353378	0,7343676	0,80617243
DM-K9-Histone-H3	0,1107	-0,1177962	1,125894	0,2978848	0,43027804
PDK1	0,106406	-0,2286699	0,953741	0,3724603	0,50827461
p38_pT180_Y182	0,09872	-0,1233594	1,03424	0,3359452	0,46880484
XPF	0,095511	-0,0517258	1,156349	0,2860453	0,41560697
Raptor	0,088684	0,0154113	0,808989	0,4455471	0,56435964
c-Kit	0,086084	-0,0356159	0,919586	0,3888417	0,51915619
PI3K-p110-a	0,082661	0,016552	0,925104	0,3861591	0,51837655
Akt	0,081209	-0,0819331	1,179535	0,2772956	0,40527812
c-Abl	0,079637	0,0173695	1,262439	0,2478299	0,37010532
PAICS	0,07913	0,0396074	0,710801	0,5005479	0,61942666
Aurora-B	0,075079	-0,1098153	0,899516	0,3987164	0,52664673
GSK-3a-b_pS21_S9	0,07313	0,1190676	0,398842	0,7020784	0,77763844
Caveolin-1	0,070314	0,2043218	0,209433	0,840166	0,88684184
YAP	0,068369	-0,039718	1,111025	0,3038096	0,4362847
IGFBP2	0,065561	0,0548377	0,461112	0,658917	0,75348374
MSI2	0,060932	0,120869	0,599495	0,5680198	0,67129616
U-Histone-H2B	0,059832	0,0240155	0,674241	0,522123	0,63473657
MEK1_pS217_S221	0,053707	-0,0937947	0,882184	0,4073923	0,52833899
Chk1_pS296	0,051428	-0,079152	0,534241	0,6099673	0,71066945
p27_pT198	0,050474	-0,0251875	0,621983	0,5539641	0,66101032
DJ-1	0,044436	-0,0755722	0,670191	0,5245494	0,63473657
p53	0,040754	-0,1158909	0,463467	0,6573101	0,75348374
C-Raf	0,03794	-0,0537902	0,312643	0,7637999	0,82744992
Stat3	0,033749	0,4534121	0,347239	0,7387735	0,80742064
C-Raf_pS338	0,028811	-0,3762361	0,414808	0,6908904	0,7721716
NAPSIN-A	0,026572	-0,2129188	0,285877	0,7833742	0,84494947
cIAP	0,026387	-0,0488455	0,417351	0,6891158	0,7721716
Bcl-xL	0,024299	-0,1228564	0,436409	0,6758845	0,76229901
CD20	0,016336	0,1297929	0,200478	0,8468943	0,89013995
GCLM	0,006495	-0,0441405	0,082951	0,9362486	0,95240706
c-Jun_pS73	0,004896	-0,302689	0,055213	0,9575349	0,96535153
Smad3	0,003638	-0,0939425	0,028646	0,9779587	0,98193417
MDM2_pS166	0,001828	-0,2026732	0,01909	0,9853102	0,98531024
TUFM	-0,0077	-0,0834473	-0,08199	0,9369835	0,95240706
MERIT40_pS29	-0,01226	0,1136221	-0,08342	0,935886	0,95240706
Wee1_pS642	-0,01489	-0,0144094	-0,11372	0,9127006	0,94325128
ER-alpha_pS118	-0,01584	-0,0209075	-0,21508	0,835933	0,88616077
AR	-0,01759	0,1283755	-0,14103	0,8918789	0,92951097
Rictor_pT1135	-0,02078	0,1220186	-0,14653	0,8876933	0,92906879
YAP_pS127	-0,02338	0,2413937	-0,0691	0,946876	0,95851789
4E-BP1	-0,03111	-0,3304412	-0,11791	0,9094996	0,94325128
HER3_pY1289	-0,03615	-0,0011075	-0,25026	0,8096846	0,86576661
JNK2	-0,03974	-0,0063912	-0,6728	0,5229878	0,63473657
PR	-0,03991	0,2736279	-0,66643	0,5268057	0,63473657
Stat3_pY705	-0,04201	0,325379	-0,33924	0,7445334	0,81013109
PKC-delta_pS664	-0,04845	7,52E-04	-0,71805	0,4963418	0,6191738
FoxM1	-0,04982	0,0565277	-0,50459	0,6295739	0,72665769
b-Catenin_pT41_S45	-0,0504	-0,0301463	-0,40772	0,6958495	0,77421098
PDK1_pS241	-0,05081	-0,1240855	-0,22416	0,8291295	0,88273697
D-a-Tubulin	-0,05104	-0,0400841	-0,60807	0,5626368	0,66813119
eIF4G	-0,05348	-0,1165538	-0,70028	0,5066953	0,62265539
TIGAR	-0,05361	-0,0403032	-0,8807	0,4081404	0,52833899
Notch3	-0,05379	-0,0941086	-0,73702	0,4854356	0,60864262
Rictor	-0,05741	-0,1662992	-0,92512	0,3861535	0,51837655
NDRG1_pT346	-0,06058	-0,0146028	-0,08866	0,9318744	0,95240706
Ets-1	-0,06377	0,0180023	-1,03536	0,3354585	0,46880484
Slfn11	-0,06517	-0,0677315	-0,79237	0,454551	0,572827
Rad50	-0,07267	-0,5211625	-0,27644	0,7903156	0,84873025
RPA32_pS4_S8	-0,07986	-0,4145376	-0,64032	0,5426597	0,65066474
p44-42-MAPK	-0,0827	0,0931581	-0,81917	0,4400946	0,56032659
eEF2	-0,08428	-0,1255586	-0,4373	0,6752698	0,76229901
CD134	-0,08434	0,1674613	-1,35055	0,2195032	0,33262135
p27-Kip-1	-0,08497	-0,1645682	-1,44029	0,193626	0,30269376
PKM2	-0,08995	0,0966772	-0,51679	0,6214707	0,72067255
A-Raf	-0,09276	-0,0118433	-0,70906	0,50156	0,61942666
G6PD	-0,09602	0,0621284	-0,87988	0,4085536	0,52833899
Cyclin_D1	-0,10112	-0,1058925	-1,48559	0,1816317	0,2990869
14-3-3_beta	-0,10458	0,0075003	-1,46237	0,1876933	0,30269376
OCT4	-0,11116	0,1129347	-2,05236	0,0798856	0,16864745
Hexokinase-II	-0,11298	0,1487511	-0,84776	0,4250306	0,5467841
eIF4E_pS209	-0,11764	-0,0196953	-1,19881	0,2701927	0,39872105
AMPK-a2_pS345	-0,11883	-0,23173	-1,54239	0,1675505	0,28383578
MSH6	-0,12202	-0,0487089	-1,25977	0,2487348	0,37010532
Creb	-0,12371	0,0671715	-2,12557	0,0717497	0,15823379
TAZ	-0,12866	0,0046104	-1,61369	0,1513017	0,26693938
WIPI2	-0,12954	0,2692593	-0,44044	0,6731039	0,76229901
cdc25C	-0,13853	0,2915575	-1,05224	0,328177	0,46319843
MMP2	-0,13915	0,0219057	-2,56676	0,0376792	0,11079485
Claudin-7	-0,14004	-0,2087561	-1,94576	0,0933966	0,18604001
IRS1	-0,14048	-0,2387615	-0,54511	0,6028599	0,70571753
N-Cadherin	-0,14183	0,0073635	-1,93337	0,0951062	0,1879298
Rab25	-0,14206	0,0114982	-1,60086	0,154112	0,26996927

GATA6	-0,1389	-0,1986784	-2,02354	0,0641846	0,10639995
Jak2	-0,14217	0,0189833	-2,3765	0,0336141	0,06242619
PAK4	-0,14445	0,0926862	-1,13604	0,2765507	0,36334052
OCT4	-0,15027	0,0565422	-3,86126	0,0019824	0,0058994
N-Cadherin	-0,1619	-0,0439391	-1,45835	0,1685958	0,23660887
P-Cadherin	-0,16595	0,1045818	-2,04382	0,0618881	0,10328628
AR	-0,1731	0,0160716	-1,84732	0,0876941	0,13709139
COG3	-0,1775	-0,2396591	-1,48881	0,1605088	0,2278487
CD20	-0,18028	-0,0020163	-2,72244	0,0174965	0,03757937
MERIT40_pS29	-0,18033	-0,0059637	-1,87899	0,0829643	0,13306605
MSI2	-0,18129	-0,0307509	-2,63475	0,0206712	0,04240027
FoxM1	-0,18286	-0,0487133	-2,49443	0,026949	0,05325125
ZAP-70	-0,20149	0,0909259	-2,62787	0,0209426	0,04240027
Rab25	-0,20263	-0,0678976	-2,90888	0,0122521	0,02828296
INPP4b	-0,20465	0,0035981	-4,54366	5,58E-04	0,00222667
ER-alpha	-0,20535	0,0807164	-4,26333	9,34E-04	0,00334396
FAK_pY397	-0,21048	0,3062533	-0,70205	0,4950855	0,58791402
FoxO3a	-0,21308	0,0217331	-3,44668	0,0043649	0,01163174
CD134	-0,21867	0,0522217	-4,13803	0,001179	0,00393535
ATM_pS1981	-0,21994	0,0095047	-4,08413	0,0013038	0,00412884
Caveolin-1	-0,2202	0,0217395	-0,70632	0,4925168	0,58768911
TTF1	-0,22137	0,0775741	-4,47425	6,34E-04	0,00248458
XBP1	-0,22244	0,0394977	-5,52061	1,00E-04	7,73E-04
Rictor_pT1135	-0,22379	-0,0240085	-1,66556	0,1198209	0,18046192
PKM2	-0,23357	-0,026473	-1,4102	0,1820713	0,24984234
cdc25C	-0,23387	0,1890208	-1,90632	0,0790684	0,12848621
Tuberin_pT1462	-0,23808	0,0478807	-3,2277	0,0066437	0,01691757
PD-L1	-0,25607	0,0026159	-6,13692	3,64E-05	4,69E-04
Hexokinase-II	-0,25661	0,0195186	-3,05028	0,0093414	0,02307335
Beclin	-0,26967	0,0575019	-5,26686	1,55E-04	9,11E-04
SDHA	-0,27331	-0,1385362	-4,27001	9,23E-04	0,00334396
B-Raf_pS445	-0,27469	0,0216048	-2,79904	0,0151178	0,03357122
MIF	-0,28066	-0,0150759	-4,2845	8,98E-04	0,00334177
G6PD	-0,28562	-0,094455	-2,45428	0,0290612	0,056079
DM-Histone-H3	-0,28801	0,0980327	-4,60032	5,04E-04	0,0021091
BRD4	-0,30036	-0,0010115	-4,09225	0,0012842	0,00411945
PR	-0,30594	0,0789249	-6,64866	1,63E-05	2,52E-04
CD4	-0,3083	0,0887386	-4,15699	0,0011381	0,00385073
WIPI2	-0,31893	0,1039822	-1,59248	0,1354101	0,19790704
B7-H4	-0,33225	0,0212483	-4,74741	3,86E-04	0,00180088
p44-42-MAPK	-0,33678	-0,104557	-2,86825	0,0132437	0,02973803
TSC1	-0,33822	-0,0948599	-1,60718	0,13214	0,19544057
SHP-2_pY542	-0,35906	-0,0189077	-4,10303	0,0012586	0,0040904
Mcl-1	-0,36634	-0,0408638	-1,86243	0,0854074	0,13542849
TFAM	-0,38584	0,2122181	-2,6807	0,0189432	0,03999125
PDHK1	-0,38987	-0,0669717	-6,22144	3,18E-05	4,44E-04
Fibronectin	-0,40037	-0,0245861	-1,03917	0,3177623	0,40878793
IGFRb	-0,40486	-0,2572525	-2,41451	0,03131	0,05948891
SOD2	-0,42029	0,0561037	-8,10914	1,99E-06	6,15E-05
PTEN	-0,44637	-0,1296311	-2,87312	0,0131207	0,02973227
LC3A-B	-0,44985	0,1000738	-2,23768	0,0434784	0,07670833
PMS2	-0,45375	0,2736831	-2,94846	0,0113571	0,02697308
MEK1	-0,45539	0,2784054	-3,46083	0,0042482	0,01153095
Atg7	-0,46796	0,0609753	-2,73123	0,0172061	0,03727985
Heregulin	-0,49019	-0,0687039	-2,46804	0,0283203	0,05507965
YAP_pS127	-0,49942	-0,0943395	-2,17194	0,049053	0,0847279
Collagen_VI	-0,50292	0,030011	-5,13438	1,95E-04	0,00109412
B-Raf	-0,51067	0,0724391	-5,4379	1,16E-04	7,89E-04
ULK1_pS757	-0,51347	0,0762755	-7,12813	7,94E-06	1,63E-04
Atg3	-0,51804	-0,0841261	-6,85944	1,18E-05	2,17E-04
Stat3_pY705	-0,54483	-0,0338044	-5,75694	6,76E-05	6,96E-04
Axl	-0,56846	-0,0268375	-8,15267	1,88E-06	6,15E-05
HSP27_pS82	-0,59208	0,1358536	-4,68489	4,32E-04	0,00194184
LDHA	-0,65569	0,079271	-4,93506	2,77E-04	0,00142332
STAT5-alpha	-0,67924	-0,0766854	-4,76584	3,74E-04	0,00177584
Gys_pS641	-0,68198	-0,0642531	-4,86307	3,14E-04	0,00155111
Glutaminase	-0,68524	-0,0335895	-4,03746	0,0014229	0,00433884
MYH11	-0,68998	0,094718	-3,28948	0,0059006	0,01534159
S6_pS240_S244	-0,6902	-0,1104895	-2,13149	0,0528086	0,08995675
TFRC	-0,69256	0,4027866	-3,52715	0,003742	0,01026971
PKC-alpha	-0,72642	-0,0970691	-5,45213	1,13E-04	7,89E-04
Granzyme-B	-0,78897	-0,0597221	-10,6371	9,17E-08	5,77E-06
p70S6K_pT389	-0,81525	-0,0130179	-4,20323	0,0010443	0,00358243
Cyclin_B1	-0,87661	0,244537	-5,39404	1,25E-04	7,89E-04
AMPK_pT172	-0,8829	-0,0045108	-6,11099	3,80E-05	4,69E-04
Gys	-0,90761	-0,0679323	-5,7821	6,49E-05	6,96E-04
Cox-IV	-0,94707	0,1957551	-5,62421	8,44E-05	7,23E-04
Myosin-IIa_pS1943	-0,99755	0,160807	-5,07158	2,18E-04	0,0011679
PLK1	-1,01791	0,1467133	-5,59318	8,89E-05	7,32E-04
MCT4	-1,02875	-0,4278113	-2,6302	0,0208504	0,04240027
Stat3	-1,03562	-0,278021	-5,65456	8,02E-05	7,23E-04
TRIM25	-1,08168	0,1025633	-5,34429	1,36E-04	8,37E-04
S6_pS235_S236	-1,21966	-0,0407079	-2,6995	0,0182779	0,03891927
PDGFR-b	-3,45067	-0,6152202	-14,695	1,90E-09	4,69E-07

ZAP-70	-0,14738	0,1672586	-1,76888	0,1209039	0,22568701
PDHK1	-0,14793	0,1395381	-1,46221	0,1877337	0,30269376
Atg3	-0,14802	0,2110792	-1,41202	0,2014673	0,31297125
XBP1	-0,14841	0,1298931	-2,51885	0,0403879	0,11217854
DM-Histone-H3	-0,15033	0,2330026	-2,06291	0,0786587	0,16835293
IRF-1	-0,15225	0,0498924	-2,39699	0,0482217	0,12300738
ATM_pS1981	-0,16042	0,0930213	-1,84521	0,1081839	0,20876115
Jak2	-0,16069	0,0485434	-2,48551	0,0423908	0,11506074
FoxO3a	-0,16263	0,0995509	-2,81837	0,0262562	0,08763892
p38_MAPK	-0,16318	0,0590571	-1,51641	0,173862	0,29016159
JNK_pT183_pY185	-0,16694	0,0252905	-1,63531	0,1466713	0,26063165
RBM15	-0,16872	0,0903623	-2,20324	0,0640248	0,14779565
Rab11	-0,17184	-0,1451814	-1,73941	0,1261882	0,23260071
Collagen_VI	-0,17461	0,3033535	-1,10641	0,3056669	0,43641459
HER3	-0,1871	-0,0724925	-2,16897	0,0673238	0,14981071
PD-L1	-0,18744	0,0995665	-3,17387	0,0159508	0,06155995
Smad1	-0,18966	-0,0055285	-3,21912	0,0149875	0,05970839
INPP4b	-0,19469	0,0618413	-2,99018	0,0205961	0,07372812
B7-H4	-0,19824	0,1663108	-1,85572	0,1065382	0,20720414
Mnk1	-0,20086	0,1533918	-1,46557	0,1868452	0,30269376
Rad51	-0,20229	0,1822849	-2,40296	0,0478038	0,12300738
MEK1	-0,20639	0,5052242	-1,19607	0,2711949	0,39872105
Tuberin_pT1462	-0,21457	0,1207483	-2,28442	0,0568454	0,13765495
PEA15_pS116	-0,21797	-0,1692776	-1,58466	0,1577332	0,27352611
B-Raf_pS445	-0,21915	0,1178505	-2,76894	0,0281732	0,09156284
CD4	-0,22355	0,2063683	-2,0594	0,0790645	0,16835293
EGFR	-0,23428	-0,3040888	-1,44886	0,1913027	0,30269376
LDHA	-0,23618	0,4319941	-1,51999	0,1729796	0,29016159
c-Myc	-0,23945	-0,2919121	-1,83696	0,1094939	0,20931118
DUSP4	-0,23995	-0,0448429	-1,69424	0,1347189	0,24602776
Beclin	-0,2442	0,139538	-3,88753	0,0061795	0,03379328
53BP1	-0,24702	-0,4465708	-1,76536	0,1215238	0,22568701
HSP70	-0,25318	-0,3041033	-2,59833	0,0359985	0,10715061
Glutamate-D1-2	-0,25518	-0,0394912	-2,01831	0,0839767	0,17156231
Granzyme-B	-0,2555	0,3769877	-2,69438	0,031348	0,09801214
Gys	-0,25676	0,4503697	-0,97707	0,3615775	0,49893661
BRD4	-0,2582	0,0962749	-2,32405	0,0536424	0,13249685
ER-alpha	-0,25858	0,1120595	-4,53533	0,0027905	0,02424311
TTF1	-0,25943	0,1196475	-4,01472	0,0052602	0,0333148
Axl	-0,26673	0,252402	-3,6237	0,0086982	0,04131634
TFAM	-0,27437	0,3617507	-1,67315	0,1388871	0,25040237
Sox2	-0,27551	0,0698959	-1,448	0,1915353	0,30269376
SHP-2_pY542	-0,28024	0,1095672	-2,47284	0,0431787	0,11545689
SOD2	-0,2808	0,2267275	-4,88818	0,0018575	0,01911688
Jagged1	-0,28263	-0,1270044	-2,2353	0,0610854	0,14507791
MIF	-0,29557	0,0525548	-4,60683	0,0025659	0,02424311
p70-S6K1	-0,29743	0,0543159	-4,58138	0,0026435	0,02424311
eEF2K	-0,32344	0,0589671	-2,17741	0,0664964	0,14931476
PREX1	-0,33267	0,1187313	-3,05014	0,0189385	0,06981802
ULK1_pS757	-0,33353	0,2888177	-3,99062	0,0054222	0,03348217
P-Cadherin	-0,33443	0,0761427	-2,94261	0,0220207	0,07770164
PEA15	-0,34867	0,0492564	-2,18148	0,0661002	0,14931476
VASP	-0,35052	-0,6562173	-2,5361	0,03939	0,11217854
S6_pS235_S236	-0,36186	0,6485896	-1,32262	0,2281608	0,34363247
Gys_pS641	-0,36883	0,2498172	-1,5819	0,1583572	0,27352611
b-Actin	-0,37532	-0,1764572	-5,26931	0,0012205	0,01435583
NF-kB-p65_pS536	-0,40735	-0,1849384	-4,54176	0,0027694	0,02424311
Merlin	-0,44466	-0,3098276	-4,07534	0,0048757	0,03273887
VEGFR-2	-0,44476	-0,0645609	-2,46791	0,0434894	0,11545689
PAK4	-0,45249	-0,0012319	-2,34393	0,0521064	0,13000281
Atg7	-0,45482	0,1900279	-1,79011	0,1172291	0,22103502
Glutaminase	-0,47371	0,2377846	-2,51829	0,0404206	0,11217854
LC3A-B	-0,47688	0,2071291	-1,44397	0,1926244	0,30269376
PMS2	-0,48922	0,378151	-2,08762	0,0758579	0,16435883
TFRC	-0,49996	0,6679799	-1,83278	0,1101638	0,20931118
B-Raf	-0,50455	0,2097444	-6,12404	5,10E-04	0,00740602
TSC1	-0,50695	-0,0779737	-1,56969	0,1611479	0,27641343
p70S6K_pT389	-0,52884	0,3247387	-1,98594	0,0880579	0,17828111
Heregulin	-0,57913	0,0224571	-2,18899	0,0653759	0,14931476
MYH11	-0,66165	0,2888351	-2,30342	0,055286	0,13520441
HSP27_pS82	-0,68487	0,2522355	-4,0859	0,0048119	0,03273887
FAK_pY397	-0,6886	0,1568537	-6,40614	3,89E-04	0,00656047
PKC-alpha	-0,71319	0,1001862	-13,2164	3,78E-06	2,34E-04
Cyclin_B1	-0,73721	0,5354757	-2,85891	0,0247867	0,0838673
E-Cadherin	-0,75573	-1,1995603	-1,49073	0,180314	0,29890978
Cox-IV	-0,80271	0,5074037	-3,0851	0,0180379	0,06854393
PLK1	-0,8331	0,494379	-3,20507	0,0152797	0,05990612
Myosin-IIa_pS1943	-0,9402	0,4484787	-3,38942	0,0118857	0,05132152
TRIM25	-0,9402	0,4484787	-3,38942	0,0118857	0,05132152
Gab2	-1,14244	-0,560432	-3,57162	0,0093168	0,04341993
p16_INK4a	-1,29666	-0,6992205	-5,94748	6,06E-04	0,00831592
Cox2	-1,35992	-0,4638683	-2,61733	0,0350245	0,10680296
PAR	-1,45606	0,0704788	-2,36477	0,0505441	0,12739176
PDGFR-b	-1,75802	1,0202718	-4,44228	0,0031161	0,02424311

Subtype A vs. MGT2

Protein	logFC	AveExpr	t	P.Value	adj.P.Val
P-Met_pY1	3,637657	-0,512787	7,504304	1,24E-05	4,37E-04
E-Cadherin	2,858419	2,07281	9,734716	1,02E-06	1,22E-04
Claudin-7	1,849309	1,334111	7,172082	1,88E-05	4,90E-04
NDRG1_pT	1,59218	0,143565	4,439983	0,001011	0,0078003
53BP1	1,390249	0,675538	4,255926	0,001372	0,0089284
HES1	1,388026	0,084085	3,031517	0,011495	0,0359412
Src_pY527	1,271446	-0,103271	5,51767	1,86E-04	0,0025465
Bim	1,1928	0,264333	3,052958	0,011065	0,0350382
RSK	0,955445	-0,315825	7,968723	7,06E-06	3,26E-04
Src_pY416	0,946306	-0,174236	4,702663	6,59E-04	0,0060253
4E-BP1	0,931205	0,004343	4,626002	7,46E-04	0,0063553
ARID1A	0,902423	0,6773	2,706231	0,020532	0,0533842
CDK1	0,841402	0,176039	2,594209	0,025065	0,0612974
Akt_pT308	0,828394	-0,134122	3,761853	0,003179	0,0153978
p16_INK4a	0,821494	0,167892	3,066624	0,010799	0,0350382
VASP	0,732469	-0,102788	3,829014	0,002831	0,0145695
Gab2	0,711829	-0,028675	2,434552	0,033271	0,0760914
IR-b	0,692957	0,020946	3,352691	0,006502	0,024048
PKC-b-II_p	0,687287	-0,232603	5,540261	1,79E-04	0,0025465
HER3	0,684612	0,467884	3,693476	0,003579	0,0164203
Rad50	0,638681	0,455258	3,340458	0,006644	0,0241119
PDK1_pS24	0,62223	0,034216	8,196864	5,40E-06	3,26E-04
Akt_pS473	0,603119	-0,184994	2,678357	0,021578	0,0549461
c-Jun_pS73	0,585643	0,276401	4,455335	9,85E-04	0,0078003
4E-BP1_pS	0,535982	0,364639	3,84237	0,002767	0,0145413
ACC1	0,510477	0,238597	2,540506	0,027575	0,0648665
HER2_pY12	0,504194	0,063467	2,666076	0,022055	0,0555883
FASN	0,501761	-7,63E-04	4,079585	0,001845	0,0109818
C-Raf_pS33	0,500747	0,080728	3,308351	0,007032	0,0248118
PAICS	0,500277	0,310122	2,043257	0,065897	0,136744
p38_pT180	0,481667	0,109864	6,322686	5,82E-05	0,0010269
GCN5L2	0,47722	0,315373	3,78688	0,003045	0,0153479
STING	0,468437	0,114471	0,704499	0,495859	0,612386
PARP	0,461149	0,302319	3,968254	0,00223	0,0122401
eIF4E	0,451018	-0,007555	4,968384	4,31E-04	0,004632
PDK1	0,445608	-0,066556	7,13043	1,98E-05	4,90E-04
Caspase-3	0,438372	0,152172	5,332925	2,45E-04	0,0031884
14-3-3_zet	0,423332	-0,017049	3,691773	0,00359	0,0164203
Paxillin	0,41394	-0,022313	6,874556	2,76E-05	6,21E-04
FAK	0,407971	-0,029242	5,123623	3,38E-04	0,004157
Histone-H3	0,385838	0,054322	2,196682	0,050528	0,1114322
p90RSK_pT	0,378022	0,017034	3,531836	0,004745	0,01947
Bax	0,368331	-0,020412	0,826717	0,426078	0,5396982
Bcl-xL	0,364332	0,192799	2,719429	0,020055	0,0526977
Jagged1	0,35794	0,087936	1,749995	0,1081	0,1964232
c-Myc	0,357068	0,281863	3,662234	0,003779	0,016971
Shc_pY317	0,338664	-0,203854	3,023125	0,011668	0,0360265
Caveolin-1	0,334515	-0,106836	0,950612	0,362349	0,4775705
RBM15	0,334174	0,521032	0,624377	0,545193	0,6474162
PRAS40_pT	0,323953	-0,104174	1,103932	0,293333	0,4188231
WIPI1	0,304193	0,243406	1,358826	0,201582	0,317138
TUFM	0,300465	0,120711	3,217371	0,008261	0,0283398
beta-Caten	0,289844	0,309686	1,608168	0,136277	0,2404317
ER-alpha_p	0,286177	0,181252	4,07261	0,001867	0,0109818
c-Kit	0,279571	0,222288	4,388641	0,0011	0,0079908
HSP70	0,27151	0,071262	3,377812	0,00622	0,0240053
DJ-1	0,265733	0,139229	2,994441	0,01228	0,0369909
RPA32_pS4	0,261734	-0,005264	2,862701	0,015532	0,044098
PLC-gamm	0,260835	-0,021192	3,350843	0,006523	0,024048
ACC_pS79	0,251971	0,126956	0,670382	0,516529	0,6254047
MSH6	0,246726	0,140975	1,52971	0,154501	0,2651031
b-Actin	0,245096	0,147224	1,529492	0,154554	0,2651031
AMPK_alph	0,241763	-0,133322	2,038531	0,066434	0,136744
Bid	0,241507	0,180387	1,811547	0,097594	0,1824368
eIF4G	0,239594	0,049167	3,538797	0,004687	0,01947
Bad_pS112	0,238065	-0,152378	2,575245	0,025924	0,0627778

Subtype B vs. MGT 2

Protein	logFC	AveExpr	t	P.Value	adj.P.Val
P-Met_pY1	4,429137	-0,83157	57,94388	2,68E-10	6,62E-08
HES1	2,033252	0,168379	33,24106	1,09E-08	9,01E-07
Notch1	1,710942	0,731063	31,59293	1,54E-08	9,49E-07
Fibronectir	1,599821	1,178052	9,284537	4,58E-05	4,52E-04
IGFRb	1,571705	0,517034	24,26581	8,87E-08	3,13E-06
MCT4	1,567953	0,191969	17,06514	9,06E-07	1,97E-05
RSK	1,545585	-0,170202	29,36662	2,50E-08	1,23E-06
Akt_pT308	1,195723	-0,094465	13,88509	3,49E-06	5,74E-05
Akt_pS473	1,162458	0,01902	12,2847	7,70E-06	1,06E-04
NDRG1_pT	1,109908	-0,493183	19,43433	3,85E-07	1,06E-05
Src_pY416	1,004716	-0,345657	16,91783	9,59E-07	1,97E-05
PKA-a	0,945893	0,458861	16,1482	1,30E-06	2,47E-05
IR-b	0,875025	-0,021004	6,686059	3,40E-04	0,0020021
Bim	0,834885	-0,210663	10,30311	2,37E-05	2,93E-04
STING	0,812935	0,218965	8,125268	1,05E-04	9,25E-04
Src_pY527	0,798604	-0,664382	7,787181	1,36E-04	0,0010837
CDK1	0,759937	-0,056419	12,81882	5,85E-06	9,03E-05
LRP6_pS14	0,702505	0,268665	11,88844	9,51E-06	1,24E-04
PKC-b-II_p	0,693778	-0,378662	9,59041	3,73E-05	4,39E-04
ACC_pS79	0,693151	0,336689	7,578502	1,60E-04	0,0012387
AMPK_pT1	0,684977	0,918582	4,163988	0,004651	0,0149183
SLC1A5	0,662634	0,316106	7,351803	1,93E-04	0,0013138
Caveolin-1	0,625031	-0,005511	6,850821	2,95E-04	0,0018659
Shc_pY317	0,579833	-0,133043	5,338815	0,001242	0,0061373
HER2_pY12	0,571485	-0,006164	8,016517	1,14E-04	9,50E-04
YAP_pS127	0,550479	0,007843	9,450377	4,10E-05	4,52E-04
ERCC5	0,506844	-0,006689	4,430638	0,003387	0,0124122
ACC1	0,498117	0,119805	6,114026	5,75E-04	0,0032184
IGF1R_pY1	0,494727	0,152044	7,286968	2,04E-04	0,0013228
PRAS40_pT	0,461013	-0,092619	7,99944	1,15E-04	9,50E-04
Tuberin	0,455819	0,202501	5,167735	0,001488	0,0068051
FASN	0,452651	-0,139704	5,22551	0,001399	0,0065509
MAPK_pT2	0,433972	-0,028119	5,953306	6,71E-04	0,0035067
Rb_pS807_	0,424155	0,468813	7,429753	1,81E-04	0,0013065
Paxillin	0,420426	-0,108736	6,765916	0,000317	0,0019426
4E-BP1	0,409332	-0,511953	6,093394	5,86E-04	0,0032184
Bad_pS112	0,388397	-0,114121	3,819178	0,007119	0,0204478
AMPK_alph	0,379505	-0,103425	3,568891	0,009808	0,0259636
PLC-gamm	0,376092	-0,008946	4,423101	0,003417	0,0124122
GSK-3a-b_p	0,371556	0,012234	5,765962	8,05E-04	0,0040593
Pdcd4	0,358724	0,30753	4,945374	0,001892	0,0080555
p21	0,354248	-0,093737	6,182004	5,39E-04	0,0030976
COG3	0,347162	0,050711	5,221219	0,001406	0,0065509
14-3-3_zet	0,340484	-0,159122	5,936645	6,81E-04	0,0035067
p90RSK_pT	0,324632	-0,097478	3,609566	0,009304	0,0255765
PDK1_pS24	0,321764	-0,281823	4,349438	0,003726	0,0129631
WIPI1	0,304937	0,177482	4,349768	0,003725	0,0129631
FAK	0,297001	-0,184835	5,008786	0,001765	0,007649
Bax	0,294233	-0,145235	5,048647	0,001691	0,0074564
Caspase-7_	0,293288	-0,035883	2,760869	0,029328	0,0619152
WIPI2	0,265893	0,088879	1,838137	0,110552	0,1870289
PDK1	0,263278	-0,273178	3,876257	0,006627	0,0197203
GATA6	0,252854	-0,001738	3,563194	0,009881	0,0259636
eIF4E	0,251234	-0,225829	4,329857	0,003814	0,0130825
Caspase-3	0,249663	-0,056699	3,822671	0,007088	0,0204478
YB1_pS102	0,247645	-0,039208	3,393642	0,012343	0,030293
UBAC1	0,237905	-0,064641	4,115195	0,004934	0,0154269
p38_pT180	0,217278	-0,15386	2,938324	0,022889	0,0504783
Myt1	0,209453	-0,049406	3,66189	0,008698	0,024413
XRCC1	0,207335	0,165378	2,451464	0,045538	0,086523
Bid	0,205877	0,106317	2,356773	0,052183	0,0969118
mTOR	0,205357	0,006248	3,857133	0,006787	0,0199584
Rictor_pT1	0,204619	0,028295	3,573809	0,009746	0,0259636
Histone-H3	0,203684	-0,139153	1,838476	0,110497	0,1870289
Chk2_pT68	0,203435	0,012562	3,233	0,015298	0,0363328
HER3_pY12	0,191942	-0,098544	3,108271	0,018117	0,0414349

p53	0,234861	-0,028158	1,717783	0,113997	0,2055273
Elk1_pS38	0,223288	-0,027692	3,153167	0,009258	0,0304905
EGFR	0,218885	-0,060514	3,053962	0,011045	0,0350382
ERCC5	0,21579	-0,13424	2,001351	0,070806	0,1432118
SLC1A5	0,214218	0,093795	0,925357	0,37476	0,4872195
TSC1	0,209308	-0,236983	1,440225	0,177827	0,2889694
Smad3	0,208068	0,189307	3,351871	0,006511	0,024048
Lck	0,206677	0,110104	1,586843	0,141034	0,2470589
Merlin	0,203843	0,100168	1,149809	0,27476	0,4057239
HER3_pY1	0,203492	-0,047216	2,946901	0,013366	0,0393039
Bak	0,193494	-0,012915	1,976923	0,073823	0,1461908
Myt1	0,193394	-0,016846	2,059608	0,06407	0,1341125
NAPSIN-A	0,193247	-0,08934	2,482779	0,030548	0,0705178
UBAC1	0,189955	-0,051966	1,969361	0,074781	0,1465416
Connexin-4	0,18699	0,477773	0,749889	0,469156	0,5852598
EGFR_pY11	0,182097	-0,013826	3,197102	0,008563	0,028975
Aurora-B	0,17813	-0,041328	1,975649	0,073983	0,1461908
PAK4	0,174688	0,016478	1,855111	0,09073	0,1723861
GSK-3a-b_	0,174242	-0,068138	0,819201	0,430172	0,5421047
MAPK_pT2	0,173967	-0,146165	0,912501	0,381192	0,4929548
ATR_pS428	0,173213	0,029853	2,740563	0,019313	0,0515209
Notch3	0,172453	0,041833	2,738095	0,019399	0,0515209
VEGFR-2	0,161657	-0,036295	0,967953	0,353999	0,4751501
MEK1_pS2	0,160906	0,032162	2,643838	0,022946	0,0572498
b-Catenin_	0,158045	0,041754	1,869614	0,088544	0,1695382
Notch1	0,156847	-0,167172	0,700098	0,498497	0,6125812
Caspase-7_	0,155075	-0,084976	1,138371	0,279303	0,4057239
TAZ	0,151172	0,185789	1,807623	0,098235	0,1824368
IGF1R_pY1	0,148883	-0,022979	2,229583	0,047709	0,106625
Chk2_pT68	0,14549	0,009538	1,474399	0,168586	0,2794674
Rictor	0,143098	0,019462	1,386335	0,193273	0,3079892
Tuberin	0,139159	0,042867	1,276209	0,228333	0,3481377
Cyclin_D1	0,129665	0,001313	1,650296	0,127294	0,2261986
TIGAR	0,129574	0,043638	1,09703	0,296209	0,4188231
YB1_pS102	0,12957	-0,081783	1,285143	0,225307	0,3461398
Glutamate-	0,127577	-0,021805	1,417977	0,184073	0,2971628
IRS1	0,116085	0,34378	0,316469	0,757609	0,8075234
AMPK-a2_	0,11558	-0,085886	1,095769	0,296737	0,4188231
PEA15	0,11237	0,139538	0,833019	0,422664	0,5396982
LC3A-B	0,109187	-0,058193	1,065715	0,309528	0,4319408
PEA15_pS1	0,101697	-0,008363	0,556717	0,588938	0,686168
p27_pT198	0,101498	0,106837	1,053753	0,314734	0,4355815
eEF2K	0,100409	0,15178	0,451722	0,660302	0,7379841
FOXO3a_p	0,094894	-0,075844	1,823714	0,095631	0,1803112
JNK_pT183	0,093291	0,025704	0,959186	0,358203	0,4756777
p27-Kip-1	0,090625	-0,052819	1,913175	0,082268	0,158752
Akt	0,089004	-0,093046	0,474568	0,644435	0,7235247
WIPI2	0,076508	-0,008059	0,424551	0,6794	0,7491602
YAP_pS127	0,074437	-0,261542	0,418235	0,683875	0,7507427
DM-K9-His	0,070306	0,013084	1,135106	0,28061	0,4057239
Stathmin	0,063385	-0,09865	0,67448	0,514019	0,6254047
Mnk1	0,061391	0,099689	0,508216	0,621402	0,7124215
p38_MAPK	0,056059	0,026224	0,387848	0,705576	0,7685687
PKM2	0,046362	-0,106769	0,241959	0,813292	0,8548214
mTOR	0,045241	-0,079951	0,426884	0,677751	0,7491602
Wee1_pS6	0,045056	0,207071	0,643198	0,533358	0,6395114
PREX1	0,044776	0,175763	0,55076	0,592877	0,6875148
IGFRb	0,044164	-0,389855	0,427417	0,677375	0,7491602
MDM2_pS	0,03625	-0,166058	0,330196	0,747494	0,8062492
p21	0,029076	-0,282497	0,317732	0,756676	0,8075234
cIAP	0,025597	-0,050772	0,50585	0,623008	0,7124215
Tyro3	0,016713	0,013275	0,306917	0,764675	0,8075234
XRCC1	0,014632	0,052949	0,148387	0,884739	0,9143541
U-Histone-	0,010909	-0,015202	0,117134	0,908879	0,9315062
MCT4	0,004452	-0,74516	0,017185	0,986599	0,992555
Rictor_pT1	0,001608	-0,09316	0,01033	0,991944	0,992555
Rad51	-0,00124	0,207918	-0,00955	0,992555	0,992555
Rb_pS807_	-0,00913	0,206846	-0,03008	0,976542	0,9885487
LRP6_pS14	-0,01096	-0,161801	-0,1418	0,889819	0,915772

PKM2	0,189979	-0,030715	2,921563	0,023427	0,0512087
RIP	0,187817	0,216472	1,363352	0,216825	0,3113704
Gys	0,185007	0,229644	3,15264	0,017055	0,0393704
PAICS	0,18402	0,011217	1,71356	0,132265	0,21214
Bak	0,18062	-0,062856	1,652588	0,144328	0,2270633
Elk1_pS38	0,175146	-0,105295	2,191381	0,066274	0,1186205
STAT5-alph	0,173442	0,775827	3,072549	0,019024	0,0431092
Stat3	0,173035	0,403483	3,030139	0,020164	0,0452767
Mcl-1	0,169579	0,479658	2,567735	0,038559	0,0767051
FOXO3a_p	0,15269	-0,06187	2,656238	0,033999	0,0694039
Stat3_pY70	0,15226	0,240467	2,857044	0,025629	0,0550477
EGFR_pY11	0,150863	-0,072296	2,273822	0,058821	0,1068297
S6_pS240_	0,1319	0,442304	1,231873	0,259488	0,3580641
ATR_pS428	0,130777	-0,033401	1,752501	0,125071	0,2059507
Akt	0,130338	-0,087664	2,386733	0,049979	0,0935216
p53	0,127715	-0,143689	1,597836	0,156039	0,2423994
Aurora-B	0,120157	-0,114976	1,811734	0,114845	0,1916666
CD20	0,11635	0,092588	1,985751	0,089289	0,1553125
Stathmin	0,115549	-0,081181	1,728994	0,129368	0,2102227
mTOR_pS2	0,114578	0,01004	1,580779	0,159865	0,2467916
Hexokinase	0,094923	0,04622	1,717849	0,131454	0,21214
SDHA	0,093427	0,182789	1,170325	0,281823	0,3762714
Lck	0,08686	-0,00688	1,189175	0,274816	0,370927
U-Histone-	0,082333	0,025272	1,155404	0,287475	0,3817538
LC3A-B	0,082153	-0,098236	1,329239	0,227257	0,3226006
MEK1	0,069103	0,359646	0,978753	0,361722	0,4600599
Tyro3	0,065279	0,038768	1,218976	0,264039	0,3603457
p44-42-MA	0,06256	0,020878	1,091774	0,312642	0,4085853
Wee1	0,057608	0,318637	0,960062	0,370391	0,4667683
S6_pS235_	0,051162	0,42135	0,330397	0,751193	0,8246435
NAPSIN-A	0,049716	-0,217621	0,466318	0,655773	0,7430087
MERIT40_	0,048588	0,087179	0,412583	0,692798	0,7813746
TSC1	0,040575	-0,38389	0,717029	0,497592	0,5995378
c-Abl	0,040335	0,046742	0,63463	0,546726	0,6523741
Collagen_V	0,040136	0,187521	0,356881	0,732159	0,8120777
cIAP	0,037947	-0,048946	0,566057	0,589807	0,6904381
TUFM	0,037279	-0,102757	0,355465	0,733171	0,8120777
MEK1_pS2	0,036383	-0,077658	0,55098	0,599535	0,6985145
b-Catenin_	0,02861	-0,070389	0,263338	0,800207	0,8519445
DJ-1	0,028009	-0,061384	0,382096	0,714224	0,8018785
ER-alpha_	0,022935	-0,039132	0,296226	0,776031	0,8336778
Gys_pS641	0,021184	0,030585	0,369732	0,722994	0,8080525
c-Kit	0,020027	0,005564	0,295583	0,776501	0,8336778
PARP	0,017185	-0,064674	0,149695	0,885413	0,904904
YAP	0,013803	-0,006171	0,246721	0,812515	0,8576546
PR	0,010656	0,24656	0,198898	0,848245	0,876638
eIF4G	-2,47E-04	-0,147012	-0,00395	0,99696	0,99696
Bcl-xL	-0,00368	-0,107499	-0,06513	0,949972	0,9577273
MDM2_pS	-0,00591	-0,199264	-0,05304	0,959248	0,9631474
PDHK1	-0,00776	0,058109	-0,12715	0,902555	0,9174122
TIGAR	-0,00856	-0,067512	-0,14815	0,886586	0,904904
DM-K9-His	-0,0101	-0,050499	-0,15294	0,882953	0,904904
Mnk1	-0,01085	0,042951	-0,11676	0,910477	0,9216714
p38_MAPK	-0,01454	-0,028369	-0,20919	0,840521	0,8723052
XPF	-0,01589	0,009206	-0,2963	0,775978	0,8336778
PAK1	-0,01791	-0,004488	-0,30113	0,77245	0,8336778
JNK_pT183	-0,01948	-0,062312	-0,34436	0,74113	0,8172287
p27_pT198	-0,02014	0,01171	-0,25504	0,806344	0,8547941
FoxM1	-0,02167	0,036726	-0,24184	0,816141	0,8578158
4E-BP1_pS	-0,02178	-0,086957	-0,2319	0,823542	0,8619269
LDHA	-0,02473	0,306928	-0,22367	0,829685	0,8646926
TFAM	-0,02557	0,215198	-0,29125	0,779674	0,8336778
Notch3	-0,02558	-0,114612	-0,32481	0,755233	0,8254095
Atg7	-0,02732	-0,058942	-0,499	0,633749	0,734911
G6PD	-0,02819	0,018532	-0,47475	0,650055	0,7430087
Rab25	-0,02846	-0,058297	-0,46709	0,655251	0,7430087
C-Raf_pS3	-0,0308	-0,347453	-0,4849	0,643199	0,7423844
B7-H4	-0,03884	0,068568	-0,70454	0,504849	0,6053288
PI3K-p110-	-0,04162	0,080436	-0,76123	0,472466	0,5748723

PAK1	-0,0136	-0,004866	-0,04448	0,965322	0,9812126
Rab11	-0,01981	-0,035245	-0,1721	0,866507	0,9030688
P-Cadherin	-0,02388	0,057863	-0,30645	0,765022	0,8075234
GCLM	-0,02593	0,020193	-0,50009	0,626924	0,713596
p70-S6K1	-0,02627	0,111673	-0,21259	0,835559	0,8745042
MMP2	-0,02702	0,049248	-0,55704	0,588724	0,686168
YAP	-0,02796	-0,037328	-0,37465	0,715085	0,7746759
JNK2	-0,03743	0,004678	-0,38679	0,706336	0,7685687
Atg7	-0,04046	-0,075657	-0,30686	0,764719	0,8075234
Chk1_pS29	-0,04384	-0,021965	-0,73728	0,476481	0,5914106
Hexokinase	-0,0487	-0,050583	-0,80062	0,440407	0,5521855
Ets-1	-0,04892	0,060662	-0,97684	0,349774	0,4746931
Creb	-0,05367	0,035296	-1,02208	0,328831	0,4487363
Cox2	-0,05874	0,957895	-0,08284	0,935477	0,9548053
PKA-a	-0,05969	-0,157512	-0,4941	0,631013	0,7149549
FAK_pY397	-0,06338	0,253015	-0,15167	0,882213	0,9143541
PMS2	-0,07143	0,147057	-0,59999	0,560745	0,6626989
N-Cadherin	-0,07154	-0,080749	-0,47941	0,641096	0,7230625
Smad1	-0,07287	0,032616	-0,65233	0,527671	0,6357796
CD20	-0,08026	-0,042893	-1,05163	0,315664	0,4355815
NF-kB-p65_	-0,08295	0,032383	-0,56537	0,583242	0,6860037
mTOR_pS2	-0,08552	-0,128678	-0,52021	0,61329	0,7078633
Slfn11	-0,08637	-0,078558	-0,96595	0,354957	0,4751501
GATA6	-0,08797	-0,225422	-0,96402	0,355882	0,4751501
Rab25	-0,08902	-0,114058	-1,14255	0,277638	0,4057239
Jak2	-0,09052	-0,008305	-1,16934	0,267136	0,3974848
IRF-1	-0,09924	0,056109	-1,47526	0,168358	0,2794674
D-a-Tubulin	-0,10018	-0,031204	-1,40656	0,187348	0,3004864
eEF2	-0,10324	0,116836	-0,63082	0,541127	0,6456925
Tuberin_pT	-0,10398	-0,006469	-1,31947	0,213982	0,3324126
ATM_pS19	-0,10456	-0,039159	-2,17966	0,052048	0,1137689
Raptor	-0,10909	0,047014	-1,25124	0,236964	0,3590808
c-Abl	-0,10954	-0,067083	-1,96493	0,075347	0,1465416
ZAP-70	-0,10962	0,048859	-1,13442	0,280886	0,4057239
Cdc2_pY15	-0,11392	0,128145	-1,08775	0,300112	0,4211795
MERIT40_p	-0,11948	-0,039726	-1,17769	0,263926	0,3950895
OCT4	-0,12349	0,032758	-2,62763	0,023618	0,0583365
eIF4E_pS20	-0,12707	0,046931	-2,2271	0,047917	0,106625
XPF	-0,13373	-0,090681	-2,36272	0,037769	0,0855867
COG3	-0,13669	-0,269422	-0,82924	0,424707	0,5396982
TFAM	-0,13704	0,118415	-0,8328	0,422781	0,5396982
cdc25C	-0,13826	0,142199	-0,92531	0,374784	0,4872195
BRD4	-0,1446	-0,06714	-2,08815	0,060995	0,1289559
PI3K-p110-	-0,14952	-0,016923	-3,41611	0,005814	0,0231627
FoxM1	-0,15471	-0,076849	-1,74971	0,108152	0,1964232
C-Raf	-0,15709	-0,011436	-1,46843	0,170171	0,2802145
PKC-delta_	-0,15902	-0,050856	-3,52424	0,004808	0,01947
14-3-3_bet	-0,17231	-0,020808	-2,84679	0,01598	0,0448519
B7-H4	-0,17286	-0,049555	-2,91179	0,01423	0,0413515
RIP	-0,17423	-0,038769	-0,94825	0,363495	0,4775705
MEK1	-0,1799	0,170994	-1,37431	0,196868	0,3117081
DUSP4	-0,18444	0,128881	-1,47938	0,167273	0,2794674
INPP4b	-0,19089	-0,024663	-3,62402	0,004039	0,0178147
p44-42-MA	-0,19152	-0,17336	-1,28421	0,225621	0,3461398
SHP-2_pY5	-0,19254	-0,09438	-2,0873	0,061084	0,1289559
CD4	-0,20334	0,03085	-2,55706	0,026776	0,063593
CD134	-0,20466	0,02215	-2,95002	0,013292	0,0393039
MYH11	-0,20505	-0,080303	-1,09766	0,295948	0,4188231
DM-Histon	-0,21518	0,048536	-2,76747	0,018408	0,0505211
ER-alpha	-0,21616	0,056836	-3,38394	0,006153	0,0240053
MIF	-0,21648	-0,062072	-2,56651	0,02633	0,0631408
G6PD	-0,21778	-0,142741	-1,51267	0,158728	0,2703856
A-Raf	-0,21779	0,051285	-1,71144	0,115192	0,2061771
Wee1	-0,21884	0,105018	-1,02382	0,328047	0,4487363
PD-L1	-0,21999	-0,036177	-4,263	0,001355	0,0089284
IGFBP2	-0,2265	-0,005399	-1,78229	0,102466	0,1888741
Beclin	-0,23185	0,016681	-3,53411	0,004726	0,01947
FoxO3a	-0,23736	-6,74E-04	-2,86391	0,015499	0,044098
XBP1	-0,24793	0,016132	-4,894	4,85E-04	0,0049924

cdc25C	-0,04292	0,229565	-0,47012	0,65319	0,7430087
ATM_pS19	-0,04503	0,019368	-0,75503	0,475937	0,5762568
N-Cadherin	-0,05146	-0,053096	-0,97553	0,363205	0,4600599
ZAP-70	-0,05551	0,105245	-0,78236	0,460763	0,5634078
MSH6	-0,05754	-0,095416	-0,59024	0,574397	0,6779627
beta-Caten	-0,05824	0,037597	-0,58706	0,576405	0,6779627
Cyclin_D1	-0,0589	-0,140117	-0,90985	0,394464	0,4920839
Glutamate-	-0,05912	-0,161659	-1,01008	0,347548	0,444789
Chk1_pS29	-0,06366	-0,024299	-0,88828	0,405158	0,5018846
JNK2	-0,0644	-0,003338	-1,11781	0,302133	0,3990735
Raptor	-0,07018	0,094162	-1,1847	0,276466	0,371125
CD134	-0,07033	0,1474	-1,07888	0,317957	0,413344
DM-Histon	-0,07749	0,178097	-1,21892	0,264059	0,3603457
Tuberin_pT	-0,08047	0,030326	-1,523	0,173486	0,2628894
GCLM	-0,08184	-0,007694	-1,31115	0,232966	0,3288146
Creb	-0,08273	0,029571	-1,46449	0,188359	0,2819681
Jagged1	-0,08358	-0,255073	-1,56374	0,163774	0,2498952
OCT4	-0,08438	0,083166	-1,61517	0,152237	0,2379901
MSI2	-0,08455	0,189506	-1,42272	0,199681	0,2918411
p27-Kip-1	-0,08616	-0,17866	-1,44399	0,19384	0,2864007
HER3	-0,08715	-0,144546	-1,5632	0,163899	0,2498952
IGFBP2	-0,08762	0,127348	-0,93491	0,382307	0,4793395
Slfn11	-0,09708	-0,066141	-1,05563	0,327722	0,4216005
c-Jun_pS73	-0,09713	-0,261039	-1,35224	0,220177	0,3143565
Rictor	-0,10149	-0,15851	-1,69216	0,136386	0,2173371
BRD4	-0,10243	-0,010294	-1,44056	0,194772	0,2864007
TFRC	-0,10351	0,423692	-0,59613	0,570677	0,6776791
PKC-delta_	-0,10618	0,01554	-1,94196	0,095138	0,1631877
PMS2	-0,1069	0,14136	-1,28062	0,242886	0,3389432
Jak2	-0,10903	3,33E-04	-1,77367	0,12132	0,2011138
p70S6K_pT	-0,10932	0,066268	-0,88584	0,406384	0,5018846
Smad3	-0,11093	-0,04749	-1,06217	0,324952	0,4202255
GCN5L2	-0,11127	-0,141842	-1,10175	0,30858	0,4054211
SHP-2_pY5	-0,11372	-0,005081	-1,81284	0,114661	0,1916666
CD4	-0,11859	0,126065	-1,40431	0,204861	0,2976512
Ets-1	-0,11913	0,029213	-2,19915	0,065532	0,118149
AMPK-a2_p	-0,11918	-0,25196	-1,38655	0,209974	0,3032958
D-a-Tubulin	-0,12001	-0,021247	-1,47906	0,18455	0,2779508
PTEN	-0,1206	0,391667	-2,1152	0,07401	0,1315135
Cdc2_pY15	-0,12447	0,146667	-2,01365	0,08575	0,1502142
53BP1	-0,12579	-0,537412	-0,87951	0,409567	0,503299
MMP2	-0,12896	-0,006025	-2,28913	0,057534	0,1052666
TAZ	-0,13101	-0,016504	-1,67688	0,139403	0,2207208
PAK4	-0,13335	-0,206457	-2,56304	0,038818	0,0767051
Rad51	-0,14224	0,123587	-2,51801	0,041397	0,0798835
Granzyme-	-0,14226	0,287889	-1,94506	0,094712	0,1631877
C-Raf	-0,1492	0,027571	-2,73584	0,03038	0,0635929
VEGFR-2	-0,15015	-0,258649	-2,51138	0,041792	0,0800201
PD-L1	-0,15136	0,053002	-2,54202	0,04	0,0777962
VASP	-0,15443	-0,79474	-1,73271	0,128679	0,2102227
Glutaminas	-0,16944	0,03487	-2,42971	0,046984	0,0885875
14-3-3_bet	-0,17243	0,016716	-2,63356	0,035111	0,0705078
XBP1	-0,17389	0,114646	-2,80976	0,027382	0,0583048
IRF-1	-0,17441	0,032658	-2,67768	0,032982	0,0678885
MYH11	-0,17671	-0,018565	-3,17057	0,016645	0,0387865
INPP4b	-0,18093	0,022965	-3,00863	0,02077	0,046217
FoxO3a	-0,18691	0,081383	-3,20605	0,015864	0,0373192
Axl	-0,18778	0,175097	-3,47248	0,011124	0,0283261
P-Cadherin	-0,19236	-0,038015	-3,31283	0,013744	0,0332818
Wee1_pS6	-0,19312	0,054334	-3,72218	0,008052	0,0228591
eEF2K	-0,19548	-0,047663	-1,28135	0,242646	0,3389432
PEA15	-0,19758	-0,070949	-1,24298	0,255622	0,354712
EGFR	-0,19836	-0,358619	-1,20859	0,267753	0,363379
Atg3	-0,19938	0,20625	-2,87195	0,025102	0,0543879
Claudin-7	-0,20193	-0,300118	-3,44577	0,011522	0,0290396
PEA15_pS1	-0,20226	-0,212926	-1,44046	0,194799	0,2864007
Smad1	-0,20614	-0,031447	-3,39845	0,012265	0,030293
Beclin	-0,20638	0,082546	-3,28545	0,014257	0,0341886
AR	-0,20926	0,202032	-4,06792	0,005227	0,0161384

PDHK1	-0,24969	-0,141532	-4,83918	5,29E-04	0,0052289
PR	-0,25538	0,031221	-4,62549	7,46E-04	0,0063553
Sox2	-0,27523	0,266721	-1,20916	0,252109	0,3797007
TTF1	-0,27763	0,059937	-4,58585	7,96E-04	0,0065523
Collagen_V	-0,28817	-0,072339	-3,15669	0,009201	0,0304905
Gys_pS641	-0,29197	-0,221008	-4,19661	0,001515	0,0093546
TFRC	-0,29611	0,24353	-1,45362	0,174154	0,2848743
MSI2	-0,32677	-0,02712	-5,08369	3,60E-04	0,004157
B-Raf_pS44	-0,33541	-0,001932	-2,50787	0,029219	0,0680857
SDHA	-0,34936	-0,159111	-4,4032	0,001074	0,0079908
Stat3_pY7(	-0,35056	-0,137707	-3,70164	0,003529	0,0164203
Pdcd4	-0,35882	-0,201283	-2,09119	0,060675	0,1289559
HSP27_pS8	-0,36439	0,019929	-2,68632	0,021274	0,0547365
AR	-0,36478	0,029133	-3,98232	0,002177	0,0122401
Fibronectir	-0,36594	-0,081243	-0,67256	0,515194	0,6254047
Glutamina:	-0,38097	-0,175164	-1,99715	0,071316	0,1432118
ATM	-0,3847	0,220743	-2,75482	0,018829	0,0511061
SOD2	-0,39075	-0,00217	-5,75394	1,31E-04	0,0021542
p70S6K_pT	-0,39572	-0,191915	-2,10826	0,058912	0,1276423
B-Raf	-0,39896	-0,012151	-3,33268	0,006736	0,0241119
ULK1_pS75	-0,42069	-0,005227	-4,72419	6,36E-04	0,0060253
LDHA	-0,44425	-0,041709	-2,82167	0,016712	0,0463804
Cyclin_B1	-0,44665	0,056019	-4,26203	0,001358	0,0089284
Gys	-0,46584	-0,262499	-6,48289	4,67E-05	9,61E-04
Axl	-0,48951	-0,112747	-5,70622	1,40E-04	0,0021659
Cox-IV	-0,51711	-0,001632	-4,23952	0,00141	0,0089284
Mcl-1	-0,5315	-0,056949	-2,00174	0,070759	0,1432118
Myosin-IIa_	-0,56759	-0,042934	-3,00941	0,011957	0,0364621
Atg3	-0,5694	-0,139995	-7,22159	1,77E-05	4,90E-04
PLK1	-0,58795	-0,05959	-3,76235	0,003177	0,0153978
PI3K-p85	-0,63576	0,037165	-9,3692	1,48E-06	1,22E-04
TRIM25	-0,65172	-0,111768	-3,25323	0,007752	0,0269689
Granzyme-	-0,67573	-0,179623	-7,87182	7,92E-06	3,26E-04
S6_pS240_	-0,68602	-0,198127	-1,49949	0,162064	0,2741772
PTEN	-0,731	-0,134067	-4,24212	0,001404	0,0089284
PKC-alpha	-0,73215	-0,187465	-3,91269	0,002452	0,0131676
AMPK_pT1	-0,78377	-0,133669	-3,97227	0,002215	0,0122401
S6_pS235_	-0,80664	-0,269325	-1,32602	0,211876	0,3312243
Stat3	-0,89634	-0,433704	-3,59133	0,004276	0,0185294
PAR	-0,90248	1,00175	-1,54287	0,151303	0,2631822
Heregulin	-0,93558	-0,049425	-5,06575	3,70E-04	0,004157
STAT5-alph	-0,95745	-0,111603	-6,36315	5,50E-05	0,0010269
PDGFR-b	-2,77677	-1,172098	-30,5446	6,21E-12	1,53E-09

PREX1	-0,2105	0,01283	-2,29758	0,056836	0,1047657
Rab11	-0,22434	-0,153641	-2,10942	0,074633	0,1316734
MIF	-0,23139	-0,023785	-4,21668	0,004365	0,0141847
eIF4E_pS2(	-0,23782	0,008209	-4,27932	0,004049	0,0133362
ULK1_pS75	-0,24075	0,194529	-3,39095	0,012387	0,030293
RPA32_pS4	-0,24076	-0,363868	-2,55644	0,039186	0,0768163
p70-S6K1	-0,24881	-0,016118	-3,88946	0,006518	0,0196337
SOD2	-0,25126	0,166774	-4,31333	0,003889	0,0131586
RBM15	-0,25367	0,095418	-4,00354	0,005657	0,0172507
ER-alpha	-0,2694	0,072059	-4,38667	0,003566	0,0127663
B-Raf_pS44	-0,27987	0,104569	-3,51451	0,010528	0,0273731
A-Raf	-0,29433	0,052879	-4,72652	0,002412	0,0099295
HSP70	-0,29789	-0,329619	-2,71675	0,03121	0,0647802
Cyclin_B1	-0,30724	0,237113	-4,47017	0,003235	0,0122912
ATM	-0,31496	0,346519	-2,63756	0,034912	0,0705078
TTF1	-0,31568	0,097674	-5,23531	0,001385	0,0065509
b-Actin	-0,33762	-0,255878	-4,66094	0,002598	0,0104183
Cox-IV	-0,37275	0,197812	-4,62509	0,002706	0,0106087
eEF2	-0,37502	-0,023708	-3,48264	0,010977	0,028242
B-Raf	-0,39284	0,078568	-7,40208	1,85E-04	0,0013065
PLK1	-0,40314	0,179577	-5,07954	0,001635	0,0073431
PI3K-p85	-0,41028	0,311163	-5,98445	6,51E-04	0,0034941
c-Myc	-0,41846	-0,261359	-4,65506	0,002615	0,0104183
ARID1A	-0,42873	-0,318287	-4,87571	0,002042	0,0085497
HSP27_pS8	-0,45719	0,043754	-4,29815	0,00396	0,0132169
DUSP4	-0,46174	0,002738	-8,67448	7,00E-05	6,40E-04
Rad50	-0,49539	-0,364531	-4,59453	0,002802	0,0108141
Merlin	-0,4973	-0,364996	-4,13384	0,004824	0,0152748
Myosin-IIa_	-0,51024	0,115317	-3,59979	0,009423	0,0255765
TRIM25	-0,51024	0,115317	-3,59979	0,009423	0,0255765
NF-kB-p65_	-0,51796	-0,210527	-8,6935	6,90E-05	6,40E-04
FAK_pY397	-0,54151	-0,02003	-7,45072	1,78E-04	0,0013065
Connexin-4	-0,54986	-0,005134	-6,74757	3,22E-04	0,0019426
IRS1	-0,56784	-0,091901	-9,31209	4,49E-05	4,52E-04
Sox2	-0,57945	0,144242	-7,32754	1,97E-04	0,0013138
Gab2	-0,60218	-0,972387	-9,2849	4,58E-05	4,52E-04
PKC-alpha	-0,71892	-0,019783	-12,4502	7,07E-06	1,03E-04
p16_INK4a	-0,93593	-1,065797	-14,4963	2,63E-06	4,65E-05
Heregulin	-1,02453	0,101335	-18,0143	6,35E-07	1,57E-05
PDGFR-b	-1,08412	0,449338	-19,4359	3,85E-07	1,06E-05
E-Cadherin	-1,36551	-1,085202	-4,44167	0,003344	0,0124122
Cox2	-2,24692	-0,342198	-34,5506	8,46E-09	9,01E-07
PAR	-2,5017	0,239122	-25,1183	7,06E-08	2,90E-06

Subtype A vs. MGT7

Protein	logFC	AveExpr	t	P.Value	adj.P.Val
E-Cadherin	4,077979	1,851072	13,59682	3,49E-08	1,73E-06
P-Met_pY1	3,905679	-0,561518	8,062376	6,39E-06	8,49E-05
RSK	2,093323	-0,522712	17,44287	2,58E-09	2,12E-07
CDK1	2,055225	-0,044656	6,337862	5,75E-05	3,23E-04
ARID1A	1,990081	0,479544	5,962624	9,74E-05	4,63E-04
Claudin-7	1,973089	1,311605	7,625586	1,08E-05	1,03E-04
Rb_pS807_	1,75058	-0,113102	5,758942	1,31E-04	5,87E-04
Cox2	1,715259	0,635351	2,416961	0,034359	0,0606194
Bim	1,640005	0,183023	4,214912	0,001475	0,0044977
ACC_pS79	1,626265	-0,122915	4,328442	0,001221	0,0039119
Connexin-4	1,604578	0,22003	6,424789	5,10E-05	3,02E-04
Rad50	1,484122	0,301541	7,84141	8,30E-06	8,92E-05
FASN	1,27253	-0,140903	10,44204	5,14E-07	1,50E-05
ACC1	1,219935	0,109605	6,171473	7,24E-05	0,000389
PAR	1,188793	0,62152	2,029457	0,067524	0,1030536
53BP1	1,147778	0,719624	3,536219	0,004722	0,01178
GCN5L2	1,090841	0,203806	9,041021	2,13E-06	4,04E-05
4E-BP1_pS	1,053014	0,270633	8,000785	6,87E-06	8,49E-05
Src_pY416	0,971179	-0,178759	4,825654	5,44E-04	0,0019745
IRS1	0,9708	0,188377	2,63659	0,023276	0,0438869
p90RSK_pT	0,943846	-0,085843	8,897744	2,49E-06	4,39E-05
ATM	0,881887	-0,009546	6,501237	4,59E-05	2,91E-04
PARP	0,817498	0,237528	7,295409	1,62E-05	1,29E-04
Akt_pS473	0,808969	-0,222421	3,568563	0,004462	0,0114804
Notch1	0,793386	-0,282907	3,537211	0,004713	0,01178
c-Jun_pS73	0,789696	0,239301	6,09568	8,06E-05	4,06E-04
beta-Caten	0,750219	0,225981	4,315244	0,001248	0,0039119
c-Myc	0,71508	0,21677	7,349606	1,52E-05	1,25E-04
C-Raf_pS3	0,619967	0,059052	4,13766	0,001679	0,0050585
PAK1	0,590111	-0,114631	1,930564	0,079928	0,1189295
RPA32_pS4	0,583537	-0,063773	6,43274	5,04E-05	3,02E-04
PI3K-p85	0,578939	-0,183689	8,546006	3,67E-06	5,90E-05
HES1	0,575656	0,231788	1,256218	0,23527	0,3026657
eIF4E	0,550199	-0,025588	6,108774	7,91E-05	4,06E-04
Cdc2_pY15	0,5306	0,01096	4,900449	4,83E-04	0,0018066
Bid	0,520625	0,129638	3,549615	0,004612	0,0117445
RBM15	0,504069	0,490142	0,941461	0,366848	0,4441738
Src_pY527	0,495044	0,037893	2,216448	0,048859	0,0807804
HER3	0,484719	0,504228	2,581701	0,025662	0,0473706
eEF2	0,478251	0,01111	2,996859	0,01225	0,0254274
AMPK_alph	0,444753	-0,170229	4,313517	0,001251	0,0039119
Smad3	0,437212	0,147645	5,818215	1,20E-04	5,59E-04
Akt_pT308	0,433648	-0,06235	1,968314	0,074962	0,1129002
PKC-b-II_p	0,427428	-0,185356	3,442722	0,005562	0,0134699
Caspase-3	0,427404	0,154166	5,252264	2,79E-04	0,0011333
Bcl-xL	0,420292	0,182625	3,147138	0,009378	0,020498
c-Kit	0,411685	0,198267	5,25049	2,80E-04	0,0011333
Wee1_pS6	0,40153	0,142258	4,645249	7,26E-04	0,0023914
Paxillin	0,385944	-0,017222	6,852062	2,87E-05	2,07E-04
Histone-H3	0,366995	0,057748	2,099147	0,059892	0,0954415
Shc_pY317	0,365979	-0,208821	3,786027	0,003059	0,0084894
HSP70	0,360941	0,055002	4,667178	7,01E-04	0,0023714
14-3-3_zet	0,354151	-0,00447	3,090472	0,010371	0,0222748
FAK	0,34764	-0,018272	3,843346	0,002771	0,0077789
PRAS40_pT	0,342914	-0,107622	1,167702	0,267813	0,3392298
VASP	0,3403	-0,031485	1,781609	0,102633	0,1491199
Lck	0,333933	0,086966	2,531525	0,028053	0,0504822
XRCC1	0,333274	-0,004986	3,395825	0,006041	0,0142101
Sox2	0,332648	0,156197	1,460706	0,172288	0,2325423
DM-K9-His	0,311906	-0,030843	4,64969	7,21E-04	0,0023914
Caspase-7_	0,31119	-0,113361	2,543016	0,027486	0,0499203
Chk2_pT68	0,310569	-0,020476	3,082131	0,010526	0,0224127
Myt1	0,30976	-0,038003	3,199444	0,008547	0,019191
Merlin	0,309137	0,081024	1,768057	0,104967	0,1516185
DJ-1	0,298587	0,133255	3,448291	0,005508	0,0134699
WIPI1	0,293815	0,245293	1,298115	0,221019	0,288845

subtype B vs. MGT 7

Protein	logFC	AveExpr	t	P.Value	adj.P.Val
P-Met_pY1	4,697158	-0,938778	74,92326	1,46E-10	3,62E-08
RSK	2,683463	-0,625353	54,86156	1,05E-09	1,29E-07
Notch1	2,347481	0,476448	45,8311	3,26E-09	2,03E-07
Rb_pS807_	2,18387	-0,235072	39,93065	7,76E-09	3,83E-07
ACC_pS79	2,067445	-0,213028	23,94125	1,93E-07	4,51E-06
CDK1	1,97376	-0,541948	38,45157	9,84E-09	4,05E-07
Fibronectir	1,530955	1,205599	9,730992	4,91E-05	2,53E-04
Akt_pS473	1,368307	-0,06332	13,46006	6,88E-06	6,30E-05
Bim	1,28209	-0,389545	24,19337	1,81E-07	4,51E-06
FASN	1,22342	-0,448012	14,95374	3,60E-06	3,58E-05
HES1	1,220882	0,493327	21,1277	4,21E-07	6,94E-06
ACC1	1,207576	-0,163979	21,16275	4,17E-07	6,94E-06
Src_pY416	1,029589	-0,355606	18,66111	9,13E-07	1,11E-05
PKA-a	0,985279	0,443107	19,2266	7,59E-07	1,04E-05
ATM	0,951623	-0,160116	8,725685	9,39E-05	4,37E-04
p90RSK_pT	0,890456	-0,323807	10,28256	3,53E-05	2,03E-04
Connexin-4	0,867729	-0,57217	10,61205	2,92E-05	1,76E-04
Pdcd4	0,811065	0,126594	11,36853	1,93E-05	1,22E-04
PI3K-p85	0,804418	-0,174715	12,15262	1,29E-05	9,34E-05
Akt_pT308	0,800977	0,063433	9,42914	5,93E-05	2,93E-04
STAT5-alph	0,729843	0,553266	12,95944	8,68E-06	6,70E-05
IGFRb	0,673653	0,876255	13,66633	6,27E-06	5,96E-05
ARID1A	0,658924	-0,75335	7,53846	2,20E-04	8,26E-04
LRP6_pS14	0,641061	0,293243	11,98267	1,40E-05	9,88E-05
Shc_pY317	0,607148	-0,143969	9,197737	6,87E-05	3,33E-04
PAK1	0,585795	-0,245972	11,11462	2,21E-05	1,36E-04
AMPK_alph	0,582495	-0,184621	10,14781	3,82E-05	2,15E-04
XRCC1	0,525977	0,037921	6,388376	5,63E-04	0,0018298
Cdc2_pY15	0,520045	-0,11114	7,186166	2,90E-04	0,0010225
Wee1	0,506484	0,139087	8,132213	1,42E-04	6,15E-04
GCN5L2	0,502351	-0,38729	5,969369	8,19E-04	0,0023802
Mcl-1	0,499886	0,347536	7,515356	2,24E-04	8,26E-04
4E-BP1_pS	0,495256	-0,29377	8,153183	1,40E-04	6,15E-04
AMPK_pT1	0,486715	0,997887	4,799688	0,002606	0,0060716
Bid	0,484994	-0,00533	3,802381	0,00811	0,0147343
PRAS40_pT	0,479974	-0,100203	8,915874	8,26E-05	3,93E-04
RIP	0,462285	0,106685	1,844754	0,112134	0,1465453
Caspase-7_	0,449403	-0,098329	8,343125	1,22E-04	5,59E-04
PTEN	0,448663	0,163963	9,436692	5,90E-05	2,93E-04
PKC-b-II_p	0,433918	-0,274718	6,139734	7,02E-04	0,0021397
Tuberin	0,409148	0,22117	4,123747	0,005535	0,0110677
Bad_pS112	0,405622	-0,121011	5,186137	0,001744	0,0045816
beta-Caten	0,402134	-0,146554	6,042147	7,66E-04	0,0023086
Paxillin	0,39243	-0,097537	7,902622	1,68E-04	6,86E-04
mTOR	0,387562	-0,066634	7,952201	1,62E-04	6,77E-04
PARP	0,373534	-0,207213	3,540071	0,011208	0,0197741
Chk2_pT68	0,368514	-0,05347	5,439334	0,001355	0,0035975
eIF4E	0,350416	-0,265501	6,760994	4,10E-04	0,0013834
Rad50	0,350054	-0,702708	3,519606	0,0115	0,0201447
Myt1	0,325818	-0,095952	5,030162	0,002046	0,0050525
HER2_pY1	0,319297	0,094711	4,232412	0,004881	0,0101314
WIPI1	0,294558	0,181634	3,502838	0,011745	0,0204291
IRS1	0,286879	-0,433787	3,969903	0,006632	0,0130014
STING	0,27701	0,433335	2,789512	0,029918	0,0461539
14-3-3_zet	0,271302	-0,131449	5,086183	0,001931	0,0049116
Stathmin	0,267642	-0,142018	4,610754	0,003195	0,0072399
COG3	0,26554	0,083359	3,802082	0,008113	0,0147343
Bak	0,250272	-0,090717	3,83869	0,007761	0,0143458
YB1_pS102	0,248613	-0,039595	3,901744	0,007194	0,0135278
Raptor	0,247552	-0,032933	4,238046	0,00485	0,0101314
SDHA	0,245534	0,121946	3,433324	0,012824	0,0216948
Caspase-3	0,238695	-0,052312	3,961999	0,006695	0,0130208
FAK	0,23667	-0,160703	2,734696	0,032237	0,0485526
DM-K9-His	0,231501	-0,147139	3,087097	0,020079	0,0324157
C-Raf	0,225082	-0,122143	4,418151	0,003954	0,0085667
IGFBP2	0,218739	0,004805	1,89843	0,103935	0,1372836

PAICS	0,290497	0,348264	1,187647	0,260186	0,3312678
Bak	0,263146	-0,025579	3,326898	0,006821	0,0157461
DUSP4	0,259141	0,04823	2,047837	0,065427	0,1010029
Bad_pS112	0,25529	-0,15551	3,110719	0,010004	0,021676
HER2_pY12	0,252006	0,109319	1,324687	0,212352	0,2813903
p38_pT180	0,244549	0,152977	3,217093	0,008283	0,0187706
p27_pT198	0,242723	0,08116	2,729391	0,019731	0,0386796
Rictor	0,231252	0,003434	2,248235	0,04622	0,077663
Wee1	0,230033	0,023405	1,07217	0,306787	0,3827096
mTOR	0,227446	-0,113079	2,147609	0,055074	0,0883326
EGFR_pY11	0,220239	-0,020761	4,315428	0,001247	0,0039119
C-Raf	0,217194	-0,079488	2,02889	0,06759	0,1030536
Stathmin	0,215478	-0,126303	2,337273	0,039539	0,0682952
Elk1_pS383	0,212288	-0,025692	3,610465	0,004147	0,0107835
ATR_pS428	0,211757	0,022845	3,414579	0,005845	0,0138809
TUFM	0,210513	0,137065	2,61265	0,024288	0,0454489
Raptor	0,208643	-0,010756	2,375235	0,036984	0,0647868
ER-alpha_p	0,208631	0,195351	2,953036	0,013245	0,0270372
Bax	0,196227	0,010879	0,440265	0,66834	0,7219861
MEK1_pS2	0,195554	0,025863	3,328584	0,006801	0,0157461
Chk1_pS29	0,18635	-0,063817	2,978386	0,01266	0,0260589
A-Raf	0,185335	-0,02201	1,464524	0,171263	0,2324288
b-Actin	0,169691	0,160934	1,054955	0,314248	0,3900466
Tyro3	0,168276	-0,014282	3,054048	0,011065	0,0233592
TAZ	0,155878	0,184933	1,815747	0,096962	0,1425579
GCLM	0,150735	-0,011929	3,187728	0,008726	0,0194173
EGFR	0,147047	-0,047453	1,591412	0,140054	0,1943441
NAPSIN-A	0,146958	-0,080924	1,935652	0,079242	0,1186224
UBAC1	0,143835	-0,04358	1,511115	0,159169	0,2172084
NF-kB-p65_	0,138268	-0,007839	0,931245	0,371876	0,4480651
eIF4G	0,133138	0,068523	1,787907	0,101565	0,1484409
PDK1	0,131864	-0,009512	2,086559	0,061208	0,0959359
YB1_pS102	0,130538	-0,081959	1,322557	0,213036	0,2813903
MSH6	0,117776	0,164421	0,748335	0,470083	0,5425722
AMPK-a2_p	0,116284	-0,086014	1,096294	0,296559	0,3737254
Notch3	0,116038	0,05209	2,258598	0,04539	0,0767905
eIF4E_pS20	0,113283	0,00323	1,683749	0,12059	0,1708614
RIP	0,100239	-0,088672	0,438797	0,669372	0,7219861
p16_INK4a	0,100033	0,299067	0,373217	0,716137	0,7657393
PI3K-p110-	0,09905	-0,062117	2,304663	0,04187	0,0718194
Pdcd4	0,093523	-0,283527	0,544331	0,597163	0,658033
p27-Kip-1	0,092997	-0,05325	1,861886	0,089752	0,1327472
Tuberin	0,092488	0,051353	0,816781	0,431528	0,5075587
XPF	0,08906	-0,131189	1,587525	0,14093	0,1944682
Aurora-B	0,087973	-0,024936	0,885671	0,394897	0,4689401
Rab11	0,085186	-0,054335	0,738243	0,475947	0,5467861
FOXO3a_p	0,083044	-0,073689	1,614393	0,134967	0,1883432
YAP	0,081174	-0,057171	1,089494	0,299416	0,3754096
IGFBP2	0,079857	-0,061101	0,593631	0,564863	0,6341875
PEA15_pS1	0,070285	-0,002652	0,382432	0,709486	0,7619261
Ets-1	0,06179	0,040532	1,220786	0,247895	0,3172537
p53	0,06094	0,003464	0,455481	0,657695	0,7156414
MDM2_pS	0,05173	-0,168872	0,477976	0,642101	0,7017648
4E-BP1	0,050315	0,164505	0,250601	0,80678	0,8408209
Cyclin_D1	0,045221	0,016667	0,563676	0,584375	0,6472668
TIGAR	0,039477	0,060019	0,334432	0,744394	0,7824057
D-a-Tubulir	0,037758	-0,056284	0,627801	0,543051	0,6152922
AR	0,018574	-0,040568	0,197774	0,846858	0,8727302
JNK2	0,011897	-0,004291	0,121746	0,905314	0,9240183
cIAP	0,002476	-0,046568	0,050399	0,960715	0,96587
b-Catenin_	2,89E-05	0,070485	3,62E-04	0,999718	0,9997176
Akt	-0,00926	-0,07518	-0,0488	0,96196	0,96587
mTOR_pS2	-0,01366	-0,141743	-0,08319	0,935205	0,9467033
PKA-a	-0,0203	-0,164673	-0,16907	0,868835	0,8904658
PLC-gamma	-0,02153	0,030147	-0,33716	0,742393	0,7824057
Slfn11	-0,02255	-0,09016	-0,25396	0,804249	0,8408209
c-Abl	-0,03094	-0,081375	-0,58467	0,570663	0,6377998
U-Histone-	-0,03409	-0,00702	-0,3345	0,744342	0,7824057
MSI2	-0,03581	-0,080021	-0,57022	0,580082	0,645407

Tyro3	0,216841	-0,021857	4,213207	0,00499	0,0102712
Lck	0,214116	-0,057782	2,749367	0,031598	0,0478822
PI3K-p110-	0,206945	-0,018991	4,176665	0,005205	0,0105379
XPF	0,206908	-0,079911	4,263259	0,004712	0,0100325
eEF2	0,20647	-0,256304	2,235929	0,064486	0,0899894
MSI2	0,206411	0,073123	3,944605	0,006835	0,0130871
IGF1R_pY1	0,203077	0,268704	3,15669	0,018323	0,0301385
p21	0,196038	-0,030453	3,725141	0,008911	0,0159489
UBAC1	0,191785	-0,046192	3,92717	0,006979	0,0132594
EGFR_pY11	0,189005	-0,087553	3,705302	0,00913	0,0162243
mTOR_pS2	0,186438	-0,018703	2,700916	0,033761	0,0502354
Histone-H3	0,184841	-0,131616	1,711879	0,135249	0,1695762
AR	0,174091	0,04869	3,060825	0,020789	0,0333433
ATR_pS428	0,169321	-0,048818	2,39691	0,051438	0,0734402
Chk1_pS29	0,166521	-0,116373	2,190569	0,068747	0,0953957
Elk1_pS383	0,164145	-0,100894	3,258162	0,016054	0,0267929
Wee1_pS6	0,163351	-0,088256	1,790287	0,121102	0,1557927
SLC1A5	0,161474	0,51657	2,657443	0,035836	0,0526878
MAPK_pT2	0,155805	0,083148	1,900907	0,103572	0,1372836
c-Kit	0,15214	-0,047281	1,584196	0,161743	0,1997521
GATA6	0,150994	0,039006	3,044622	0,02124	0,0338469
FOXO3a_p	0,140839	-0,05713	2,660797	0,035671	0,0526878
S6_pS240_	0,12355	0,445644	1,144789	0,293792	0,3406887
YAP	0,122936	-0,049824	2,363145	0,053928	0,0765528
Bax	0,122129	-0,076393	2,416638	0,050038	0,0718576
p27_pT198	0,121086	-0,04478	2,287947	0,059933	0,0841099
c-Abl	0,118939	0,015301	2,172045	0,070568	0,0973764
Smad3	0,11821	-0,139148	0,958144	0,373195	0,4267554
A-Raf	0,108797	-0,10837	1,961889	0,095005	0,1275342
c-Jun_pS73	0,106923	-0,34266	1,761632	0,126097	0,1605466
GCLM	0,094828	-0,078361	1,893753	0,104626	0,1374601
PLC-gamma	0,093728	0,104	1,902321	0,103365	0,1372836
C-Raf_pS33	0,08842	-0,395141	1,749967	0,128188	0,1623709
RPA32_pS4	0,08104	-0,492589	0,878326	0,411911	0,4667061
MEK1_pS2	0,071031	-0,091517	1,206356	0,27089	0,3171079
DJ-1	0,060863	-0,074525	0,935965	0,383663	0,4367036
Bcl-xL	0,052279	-0,129883	1,046435	0,333722	0,3833918
U-Histone-	0,037331	0,043273	0,397148	0,704325	0,7531093
Akt	0,032079	-0,04836	0,486619	0,642945	0,6965234
IR-b	0,031783	0,316294	0,225578	0,828649	0,8457697
Aurora-B	0,030001	-0,078913	0,331881	0,750711	0,7919369
Sox2	0,028433	-0,098909	0,367761	0,725056	0,7686211
Src_pY527	0,022202	-0,353822	0,418916	0,689144	0,7400808
D-a-Tubulir	0,017926	-0,076422	0,328062	0,753462	0,7919369
cIAP	0,014827	-0,039698	0,238545	0,819006	0,8393962
MDM2_pS	0,009568	-0,205456	0,088181	0,932461	0,9400729
PKC-delta_	0,009284	-0,030647	0,158939	0,878675	0,8894786
NAPSIN-A	0,003427	-0,199106	0,033002	0,974691	0,9746906
eIF4E_pS20	0,002538	-0,087934	0,0336	0,974232	0,9746906
Ets-1	-0,00842	-0,015073	-0,16254	0,875957	0,8894786
Rictor	-0,01333	-0,193772	-0,24154	0,816783	0,8393962
JNK2	-0,01507	-0,023068	-0,25977	0,803298	0,8336747
DUSP4	-0,01817	-0,174693	-0,30705	0,768672	0,8044994
p38_pT180	-0,01984	-0,059012	-0,276	0,791352	0,824742
PAICS	-0,02576	0,095129	-0,24407	0,814904	0,8393962
Slfn11	-0,03327	-0,091667	-0,36891	0,72424	0,7686211
14-3-3_bet	-0,03672	-0,03757	-0,60925	0,563605	0,6159749
p53	-0,04621	-0,07412	-0,70626	0,505217	0,5595896
PDK1	-0,05047	-0,14768	-0,74929	0,480636	0,5371812
TUFM	-0,05267	-0,066776	-0,69002	0,514708	0,5675577
ER-alpha_p	-0,05461	-0,008113	-0,71014	0,502968	0,5595896
c-Myc	-0,06045	-0,404563	-0,68072	0,520194	0,5710574
MERIT40_p	-0,07311	0,13586	-0,5356	0,610552	0,6643448
FoxM1	-0,07797	0,059247	-0,85536	0,42359	0,4777478
Claudin-7	-0,07815	-0,34963	-1,25117	0,255196	0,3001594
Notch3	-0,08199	-0,092046	-1,52618	0,175339	0,2133429
CD20	-0,08368	0,172599	-1,58464	0,161643	0,1997521
RBM15	-0,08377	0,02746	-1,53398	0,173451	0,2120923
p27-Kip-1	-0,08378	-0,179609	-1,35737	0,221134	0,2625964

14-3-3_bet	-0,0366	-0,045484	-0,61735	0,549669	0,6199463
Smad1	-0,0399	0,026621	-0,35583	0,728754	0,775872
Jagged1	-0,04015	0,160316	-0,19628	0,847997	0,8727302
PKC-delta_	-0,04355	-0,07185	-0,89381	0,390713	0,4684759
Heregulin	-0,04479	-0,211387	-0,24282	0,812649	0,8433794
MMP2	-0,0474	0,052953	-0,98348	0,346679	0,426019
IRF-1	-0,05491	0,04805	-0,81101	0,434694	0,5088597
STING	-0,06749	0,211912	-0,10141	0,921061	0,9362228
LRP6_pS14	-0,0724	-0,15063	-0,94618	0,36454	0,4441738
MAPK_pT2	-0,1042	-0,095589	-0,54094	0,599423	0,658033
Rad51	-0,12134	0,229754	-0,88946	0,392944	0,4688757
PDK1_pS24	-0,12291	0,169696	-1,70236	0,116972	0,1679774
p70-S6K1	-0,12351	0,129352	-1,00516	0,336594	0,4156942
p21	-0,12913	-0,253731	-1,41581	0,18474	0,246653
Creb	-0,13563	0,050198	-2,6463	0,022878	0,0434674
IGF1R_pY1	-0,14277	0,030049	-2,1569	0,054193	0,0874881
IR-b	-0,15029	0,174263	-0,7185	0,487553	0,5575264
eEF2K	-0,15551	0,19831	-0,71006	0,492563	0,5606592
PTEN	-0,16174	-0,237569	-0,94207	0,366548	0,4441738
TTF1	-0,16512	0,039481	-2,9145	0,014186	0,0284879
OCT4	-0,17705	0,042496	-3,68896	0,003618	0,00961
FoxO3a	-0,1888	-0,009503	-2,29361	0,04269	0,0727205
PEA15	-0,1898	0,194478	-1,43683	0,178817	0,2400428
GATA6	-0,18983	-0,206902	-2,21413	0,049057	0,0807804
Jak2	-0,19383	0,01048	-2,52848	0,028205	0,0504822
ER-alpha	-0,19453	0,052902	-3,15482	0,009251	0,0204008
XBP1	-0,19696	0,006864	-4,28252	0,001317	0,0040676
SDHA	-0,19726	-0,186767	-2,58089	0,025699	0,0473706
PREX1	-0,19958	0,220191	-2,42653	0,033783	0,0600322
Mcl-1	-0,20119	-0,117005	-0,75625	0,465519	0,5398267
JNK_pT183	-0,20163	0,079326	-2,08504	0,061368	0,0959359
FoxM1	-0,21101	-0,066612	-2,36236	0,037832	0,065806
B-Raf_pS44	-0,21397	-0,024011	-1,6566	0,126047	0,176895
COG3	-0,21831	-0,254581	-1,31567	0,215261	0,282817
INPP4b	-0,2184	-0,019661	-3,74148	0,003304	0,0090666
CD134	-0,23267	0,027245	-3,45868	0,005409	0,0133595
MERIT40_p	-0,24118	-0,017598	-2,18225	0,051857	0,0842679
p38_MAPK	-0,24121	0,080272	-1,68139	0,121056	0,1708614
N-Cadherin	-0,25227	-0,047889	-1,68977	0,119409	0,1704862
HER3_pY12	-0,2527	0,035728	-3,6702	0,003738	0,0098224
Glutamate-	-0,26454	0,049489	-2,943	0,013484	0,0272993
CD20	-0,28029	-0,006524	-3,7106	0,003485	0,0094595
SLC1A5	-0,28694	0,184915	-1,26037	0,233826	0,3023821
PD-L1	-0,29215	-0,023057	-5,77988	1,27E-04	5,80E-04
ZAP-70	-0,29337	0,082267	-3,03637	0,011418	0,0239012
Beclin	-0,30749	0,030434	-4,99438	4,16E-04	0,0015821
P-Cadherin	-0,30802	0,109523	-3,43219	0,005666	0,0135884
Rab25	-0,31623	-0,072747	-4,07211	0,001876	0,005516
Mnk1	-0,31864	0,168786	-2,7471	0,019118	0,037778
cdc25C	-0,32948	0,176967	-2,05459	0,064672	0,1004658
ATM_pS19	-0,33532	0,002799	-7,52054	1,23E-05	1,12E-04
MIF	-0,34484	-0,038735	-4,13008	0,001701	0,0050618
G6PD	-0,35346	-0,118073	-2,2265	0,048009	0,080123
PR	-0,35651	0,049608	-6,40147	5,26E-05	3,02E-04
FAK_pY397	-0,35757	0,306504	-0,85553	0,410652	0,4853166
DM-Histon	-0,36085	0,075023	-4,6957	6,69E-04	0,0023286
Gab2	-0,36871	0,167786	-1,26068	0,233719	0,3023821
Tuberin_pT	-0,37218	0,042294	-4,68225	6,84E-04	0,0023466
STAT5-alph	-0,40104	-0,212767	-2,65449	0,022546	0,0431703
CD4	-0,41325	0,069014	-5,17645	3,14E-04	0,0012104
ERCC5	-0,419	-0,018824	-3,96449	0,002252	0,0065448
GSK-3a-b_p	-0,42261	0,040381	-1,99485	0,071644	0,1085653
VEGFR-2	-0,42756	0,070836	-2,55996	0,026672	0,0488002
Fibronectin	-0,4348	-0,068722	-0,80195	0,439698	0,5122902
Rictor_pT1	-0,44919	-0,011196	-2,89756	0,014621	0,0291246
SOD2	-0,44982	0,008572	-6,68145	3,60E-05	2,41E-04
BRD4	-0,45613	-0,010498	-6,96157	2,49E-05	1,86E-04
PAK4	-0,46359	0,132529	-4,87975	4,99E-04	0,0018389
Hexokinase	-0,46452	0,025019	-7,4514	1,34E-05	1,18E-04

PR	-0,09048	0,287013	-1,77088	0,124464	0,1592882
Atg3	-0,09665	0,16516	-0,84611	0,428357	0,4809286
CD134	-0,09835	0,158608	-1,7218	0,133374	0,1680785
TIGAR	-0,09866	-0,031474	-1,83394	0,113861	0,1472443
Stat3	-0,10554	0,514912	-2,07753	0,080657	0,1100685
eIF4G	-0,1067	-0,10443	-1,39794	0,209231	0,2508744
AMPK-a2_p	-0,11848	-0,252242	-1,3651	0,21882	0,2611044
Rab11	-0,11934	-0,195639	-1,11099	0,307043	0,3543911
XBP1	-0,12292	0,094257	-2,48741	0,045337	0,0654866
TAZ	-0,12631	-0,018387	-1,53397	0,173452	0,2120923
ERCC5	-0,12795	0,247226	-1,15828	0,28864	0,3362926
b-Catenin_	-0,12941	-0,007183	-1,28495	0,243892	0,2882362
IRF-1	-0,13009	0,014927	-2,03833	0,085258	0,1157078
Heregulin	-0,13374	-0,254981	-2,65088	0,036161	0,0528507
OCT4	-0,13794	0,10459	-2,72506	0,032665	0,0488979
FoxO3a	-0,13835	0,061959	-2,61028	0,03824	0,0555612
Cyclin_D1	-0,14334	-0,106339	-2,13945	0,073894	0,1013985
E-Cadherin	-0,14595	-1,573026	-0,44404	0,671813	0,7246191
MMP2	-0,14934	0,002127	-2,8531	0,027448	0,0426387
B-Raf_pS44	-0,15844	0,055994	-2,77263	0,030613	0,0466748
G6PD	-0,16386	0,072803	-1,40337	0,207682	0,2502312
Creb	-0,16469	0,062356	-3,28144	0,015578	0,0261749
Smad1	-0,17317	-0,044635	-2,91578	0,025225	0,0396852
MSH6	-0,18649	-0,043836	-2,31558	0,057649	0,0813681
TTF1	-0,20318	0,052673	-4,3103	0,004466	0,0095913
INPP4b	-0,20844	0,033968	-3,0191	0,021972	0,0347891
HSP70	-0,20846	-0,365392	-1,98403	0,092073	0,1242738
Jak2	-0,21235	0,04166	-3,77507	0,008384	0,015115
DM-Histon	-0,22316	0,236368	-3,83638	0,007783	0,0143458
PD-L1	-0,22352	0,081866	-4,11713	0,005577	0,0110677
GSK-3a-b_p	-0,22529	0,250974	-4,24644	0,004803	0,0101314
p44-42-MA	-0,22796	0,137085	-4,1135	0,005601	0,0110677
N-Cadherin	-0,23219	0,019196	-4,78009	0,002661	0,0061419
PEA15_pS1	-0,23367	-0,200361	-1,60621	0,156849	0,1956648
cdc25C	-0,23414	0,306053	-1,84101	0,112729	0,1465474
Stat3_pY70	-0,23629	0,395886	-5,00692	0,002095	0,0051244
ZAP-70	-0,23925	0,178742	-3,47374	0,012184	0,0208984
Rictor_pT1	-0,24618	0,208616	-5,06932	0,001965	0,0049116
ER-alpha	-0,24776	0,063404	-4,6993	0,002902	0,0066368
Rab25	-0,25567	0,032587	-4,47119	0,003726	0,0081454
Rad51	-0,26234	0,171627	-3,13177	0,018932	0,0307647
HER3_pY12	-0,26425	0,083933	-4,52409	0,003514	0,0078198
EGFR	-0,2702	-0,329884	-1,42435	0,201796	0,2443318
ATM_pS19	-0,2758	0,111675	-5,53879	0,001229	0,0033368
Beclin	-0,28202	0,112803	-5,74327	0,001011	0,0028377
HER3	-0,28705	-0,064588	-4,18439	0,005159	0,0105306
PDHK1	-0,2881	0,170248	-4,58055	0,003302	0,0074152
NF-kB-p65_	-0,29675	-0,299014	-4,50104	0,003605	0,0079502
SOD2	-0,31034	0,190406	-5,81696	9,43E-04	0,0027094
p38_MAPK	-0,31181	0,090537	-5,08912	0,001925	0,0049116
JNK_pT183	-0,3144	0,055657	-6,22359	6,51E-04	0,0020354
Hexokinase	-0,32089	0,212545	-5,7514	0,001003	0,0028377
CD4	-0,3285	0,210027	-3,89766	0,007229	0,0135278
Axl	-0,34568	0,238258	-6,02639	7,77E-04	0,0023137
p70-S6K1	-0,34604	0,022775	-6,00728	7,91E-04	0,0023264
Tuberin_pT	-0,34867	0,137605	-6,78324	4,02E-04	0,0013797
B7-H4	-0,35764	0,196086	-7,28396	2,68E-04	9,60E-04
MIF	-0,35975	0,027556	-7,53573	2,21E-04	8,26E-04
53BP1	-0,36826	-0,440424	-2,78543	0,030084	0,0461539
Granzyme-	-0,36875	0,378485	-4,9506	0,002222	0,0053806
PKM2	-0,36988	0,193229	-5,66983	0,001084	0,0030082
Collagen_V	-0,38936	0,359319	-3,44023	0,012712	0,0216539
Mnk1	-0,39088	0,194965	-5,10519	0,001894	0,0049116
Merlin	-0,39201	-0,407113	-3,48727	0,011977	0,0206882
PAR	-0,41043	-0,597385	-3,94903	0,006799	0,0130871
b-Actin	-0,41302	-0,225716	-5,63988	0,001115	0,0030611
BRD4	-0,41397	0,114319	-6,88796	3,69E-04	0,001283
PDK1_pS24	-0,42338	0,016233	-6,74715	4,14E-04	0,0013834
ULK1_pS75	-0,42631	0,268754	-6,28088	6,19E-04	0,0019593

Atg3	-0,46667	-0,158672	-4,70623	6,58E-04	0,0023222
p44-42-MA	-0,48204	-0,120539	-3,22509	0,008167	0,0186778
B7-H4	-0,49165	0,008408	-8,41028	4,28E-06	6,21E-05
PKM2	-0,5135	-0,004977	-2,67252	0,021835	0,0421338
SHP-2_pY5	-0,52558	-0,033828	-5,68291	1,46E-04	6,45E-04
PDHK1	-0,53004	-0,09056	-9,83574	9,32E-07	2,09E-05
ULK1_pS75	-0,60626	0,028512	-6,83676	2,93E-05	2,07E-04
B-Raf	-0,62237	0,028469	-5,20918	2,98E-04	0,001188
TFAM	-0,63463	0,208887	-3,86464	0,002672	0,0076739
Axl	-0,64741	-0,084037	-7,38521	1,45E-05	1,24E-04
S6_pS240_	-0,69437	-0,196608	-1,51629	0,157871	0,2166337
WIPI2	-0,71436	0,135735	-3,69479	0,003582	0,00961
Collagen_V	-0,71767	0,005751	-7,86435	8,08E-06	8,92E-05
PKC-alpha	-0,72069	-0,189548	-3,85003	0,00274	0,0077785
MEK1	-0,73088	0,271173	-5,64225	1,55E-04	6,73E-04
Stat3_pY7	-0,7391	-0,067062	-7,84177	8,30E-06	8,92E-05
NDRG1_pT	-0,74879	0,569197	-2,08564	0,061304	0,0959359
Caveolin-1	-0,77492	0,094879	-2,19405	0,050804	0,0831027
HSP27_pS8	-0,81976	0,102724	-6,4187	5,14E-05	3,02E-04
PMS2	-0,83606	0,28608	-7,01512	2,32E-05	1,79E-04
IGFRb	-0,85389	-0,226573	-8,50891	3,82E-06	5,90E-05
LDHA	-0,86714	0,035181	-6,00222	9,21E-05	4,46E-04
TSC1	-0,88574	-0,037883	-6,09638	8,05E-05	4,06E-04
Atg7	-0,89547	0,079798	-6,81611	3,01E-05	2,07E-04
Granzyme-	-0,90222	-0,138443	-10,3752	5,48E-07	1,50E-05
AMPK_pT1	-0,98203	-0,097621	-5,5402	1,81E-04	7,69E-04
Glutaminas	-0,9895	-0,064521	-5,18491	3,10E-04	0,0012104
LC3A-B	-1,00888	0,145092	-9,86349	9,06E-07	2,09E-05
Gys_pS641	-1,07199	-0,079185	-15,6395	8,13E-09	5,02E-07
YAP_pS127	-1,07328	-0,052866	-6,0415	8,70E-05	4,30E-04
TFRC	-1,08901	0,387694	-5,3443	2,43E-04	0,0010155
Stat3	-1,17491	-0,383054	-4,7085	6,56E-04	0,0023222
MYH11	-1,17492	0,096037	-6,28033	6,22E-05	3,42E-04
p70S6K_pT	-1,23478	-0,039359	-6,59755	4,03E-05	2,62E-04
Cyclin_B1	-1,30657	0,212369	-12,9819	5,63E-08	2,32E-06
Gys	-1,34938	-0,101855	-18,948	1,07E-09	1,33E-07
Cox-IV	-1,37704	0,154718	-11,6248	1,74E-07	6,15E-06
Myosin-IIa_	-1,42752	0,113416	-7,65622	1,04E-05	1,03E-04
PLK1	-1,44787	0,096759	-9,42633	1,42E-06	2,91E-05
TRIM25	-1,51165	0,044582	-7,62208	1,08E-05	1,03E-04
S6_pS235_	-1,63268	-0,119137	-2,68167	0,021482	0,041779
MCT4	-2,06196	-0,369449	-8,01736	6,74E-06	8,49E-05
PDGFR-b	-4,12457	-0,927044	-45,2463	9,13E-14	2,25E-11

SHP-2_pY5	-0,44676	0,128133	-7,35017	2,55E-04	9,25E-04
LDHA	-0,44762	0,476085	-8,05559	1,50E-04	6,38E-04
Glutamate	-0,45124	-0,004813	-8,2162	1,34E-04	6,00E-04
eEF2K	-0,4514	0,054703	-3,15247	0,018425	0,0301385
PREX1	-0,45485	0,110573	-4,87965	0,002394	0,0056852
4E-BP1	-0,47156	-0,159597	-7,88824	1,69E-04	6,86E-04
Cox2	-0,47292	-1,051796	-7,54332	2,19E-04	8,26E-04
P-Cadherin	-0,4765	0,075639	-5,44259	0,00135	0,0035975
Jagged1	-0,48167	-0,095837	-9,99258	4,19E-05	2,20E-04
MEK1	-0,48188	0,58004	-7,78747	1,83E-04	7,27E-04
Caveolin-1	-0,4844	0,438262	-4,86887	0,002421	0,0056956
MCT4	-0,49846	1,018534	-6,5153	5,04E-04	0,0016613
PEA15	-0,49975	0,049919	-3,19957	0,017324	0,028719
TFAM	-0,52316	0,414235	-6,14579	6,98E-04	0,0021397
WIPI2	-0,52497	0,405226	-2,88814	0,026181	0,0409279
VASP	-0,5466	-0,637872	-6,32922	5,93E-04	0,001902
YAP_pS127	-0,59724	0,466929	-11,6211	1,69E-05	1,16E-04
B-Raf	-0,61625	0,167933	-13,0013	8,51E-06	6,70E-05
Gys	-0,69854	0,583061	-13,0449	8,34E-06	6,70E-05
PKC-alpha	-0,70746	-0,024366	-13,1365	7,99E-06	6,70E-05
VEGFR-2	-0,73937	-0,022961	-13,2104	7,72E-06	6,70E-05
Gys_pS641	-0,75884	0,342594	-14,9396	3,62E-06	3,58E-05
PAK4	-0,77163	0,048855	-14,9624	3,59E-06	3,58E-05
S6_pS235_	-0,77487	0,751764	-4,92621	0,002279	0,0054662
Glutaminas	-0,77798	0,278284	-11,5562	1,75E-05	1,17E-04
FAK_pY397	-0,8357	0,097646	-12,5867	1,04E-05	7,77E-05
PMS2	-0,87153	0,44721	-10,5314	3,06E-05	1,80E-04
Atg7	-0,88232	0,28306	-18,5744	9,40E-07	1,11E-05
TFRC	-0,89641	0,740853	-5,06731	0,001969	0,0049116
HSP27_pS8	-0,91256	0,225903	-11,3629	1,93E-05	1,22E-04
p70S6K_pT	-0,94837	0,401891	-7,75291	1,87E-04	7,34E-04
LC3A-B	-1,03592	0,348992	-17,8253	1,21E-06	1,36E-05
TSC1	-1,05448	0,054131	-20,1759	5,62E-07	8,67E-06
MYH11	-1,14658	0,369384	-21,5179	3,76E-07	6,94E-06
Cyclin_B1	-1,16717	0,581082	-23,7859	2,01E-07	4,51E-06
NDRG1_pT	-1,23107	0,443207	-22,2046	3,09E-07	6,36E-06
Cox-IV	-1,23267	0,541782	-18,8318	8,63E-07	1,11E-05
PLK1	-1,26306	0,523547	-19,7823	6,35E-07	9,23E-06
Myosin-IIa_	-1,37016	0,459287	-10,0217	4,12E-05	2,20E-04
TRIM25	-1,37016	0,459287	-10,0217	4,12E-05	2,20E-04
p16_INK4a	-1,65739	-0,777213	-27,0609	8,95E-08	2,76E-06
Gab2	-1,68271	-0,540172	-27,826	7,52E-08	2,65E-06
PDGFR-b	-2,43191	0,988457	-45,7566	3,29E-09	2,03E-07
