# Supplementary material for: Modeling Heterogeneity of Triple‐Negative Breast Cancer Uncovers a Novel Combinatorial Treatment Overcoming Primary Drug Resistance
Source: Adv Sci (Weinh). 2020 Dec 16;8(3):2003049. doi: 10.1002/advs.202003049 (PMC7856896; doi:10.1002/advs.202003049)
Supplement: Supplementary file 8 — Supplemental Table 7 [file ADVS-8-2003049-s008.pdf]

Table S7. Changes in protein expression and/or phosphorylation levels occurring in *MMTV-R26* ^{Met} MGT4 cells when exposed to single or combined treatment targeting BCL-XL (A1155463) and WEE1 (Adavosertib).

MGT4 + A11 + ADAV vs. MGT4 + A11

Protein	logFC	AveExpr	t	P.Value	adj.P.Val	B
PAR	2,628347	-0,447016	37,96932	9,93E-14	4,28E-11	22,00919
Histone-H3	1,565677	-0,22751	20,15493	1,59E-10	2,29E-08	14,67206
CDK1_pT14	1,240134	-0,263332	23,3577	2,90E-11	6,24E-09	16,42536
Caspase-7-cleaved-	1,200468	0,044123	18,16382	5,27E-10	3,78E-08	13,43042
p53	1,089415	0,265969	12,73397	2,90E-08	1,14E-06	9,21298
Caspase-3-cleaved	1,049189	-0,157487	12,46308	3,68E-08	1,32E-06	8,960888
DM-Histone-H3	0,923131	0,044083	13,51466	1,50E-08	6,45E-07	9,913311
JNK2	0,794061	0,368288	10,1844	3,32E-07	8,30E-06	6,627543
H2AX_pS140	0,79091	-0,061435	16,25579	1,87E-09	1,15E-07	12,10571
KAP1	0,786688	-0,155818	3,727147	0,002957	0,0144311	-2,94911
Vinculin	0,759792	-0,081115	2,868058	0,014318	0,0484051	-4,54142
p38-MAPK-_pT180_Y182	0,699031	0,014931	11,65955	7,66E-08	2,36E-06	8,18345
H2AX_pS139	0,627217	-0,076421	10,14326	3,47E-07	8,30E-06	6,581517
RBM15	0,586131	0,009748	6,343481	3,95E-05	4,86E-04	1,557214
Caveolin-1	0,497422	-0,149569	3,132231	0,008791	0,0347618	-4,05453
S6_pS235_S236	0,492271	0,525909	2,696065	0,019659	0,0596703	-4,85442
Cyclophilin-F	0,461977	-0,349202	3,065413	0,009945	0,0375981	-4,17819
SOD1	0,44144	-0,066911	7,220316	1,14E-05	1,97E-04	2,86881
ER-a_pS118	0,422159	-0,029565	4,553872	6,86E-04	0,0043459	-1,43986
CREB_pS133	0,416132	0,0246	5,047487	2,98E-04	0,0021796	-0,57152
p90RSK_pT573	0,409055	0,127706	2,299995	0,040483	0,1020363	-5,55473
MAPK_pT202_Y204	0,372642	0,259927	3,404308	0,005328	0,0244283	-3,54909
AMPKa_pT172	0,369015	0,069402	6,31148	4,14E-05	4,95E-04	1,507402
Bcl2	0,36725	0,005942	3,090251	0,0095	0,0363294	-4,13225
S6	0,350749	-0,221281	2,27055	0,042686	0,1063461	-5,60527
TRAP1	0,348811	-0,205144	3,026304	0,01069	0,0390462	-4,25044
MLH1	0,344489	-0,022308	4,364849	9,51E-04	0,0056929	-1,77969
Claudin-7	0,334078	-0,262171	3,942495	0,002006	0,0107897	-2,55099
Caspase-8	0,325171	-0,049334	2,832361	0,015293	0,0503401	-4,60671
c-Jun_pS73	0,324502	-0,075111	4,255394	0,001152	0,0067998	-1,97811
C-Raf_pS338	0,313519	-0,094016	3,878419	0,002251	0,0116171	-2,66918
ATP5A	0,288493	-0,041445	3,192491	0,007866	0,0326002	-3,94279
Myosin-IIa	0,276154	0,125587	1,109796	0,289133	0,4311983	-7,28761
MTCO1	0,273638	0,022592	1,95599	0,07449	0,1583829	-6,12722
MDM2_pS166	0,271517	0,002366	3,674281	0,003254	0,0155852	-3,04718
Tau	0,268593	0,023815	3,868508	0,002291	0,0116171	-2,68748
GRB7	0,257698	-0,033547	3,184705	0,00798	0,0327566	-3,95724
MIG6	0,254672	-0,034288	4,738494	5,00E-04	0,0033696	-1,1117
Bad_pS112	0,252335	0,10143	5,30182	1,97E-04	0,0016013	-0,13596
Wee1_pS642	0,250104	0,077241	4,470019	7,92E-04	0,0049491	-1,59016
SF2	0,248546	-0,07376	2,060932	0,061995	0,1406317	-5,95706
Mitofusin-1	0,242395	0,041273	1,385346	0,191507	0,3275377	-6,95901
VHL-EPPK1	0,24024	-0,032711	1,062236	0,309329	0,4488914	-7,33848
IRF-1	0,235718	0,017763	2,498176	0,028256	0,0787308	-5,20861
EGFR	0,232973	0,023382	3,722864	0,00298	0,0144311	-2,95705
Ambra1_pS52	0,22697	0,096777	2,945236	0,012417	0,0438658	-4,3998
Annexin-VII	0,224964	-0,018589	2,973672	0,011782	0,0423152	-4,34747
SOD2	0,220183	0,043969	2,537959	0,026275	0,0749969	-5,13801
cdc25C	0,219803	0,066872	3,056027	0,010119	0,0375981	-4,19554
Caspase-8-cleaved	0,212609	-0,011594	3,900048	0,002165	0,0113787	-2,62926
Fibronectin	0,212218	0,223085	3,165356	0,00827	0,0333121	-3,99313
ADAR1	0,207581	-0,023956	2,583851	0,024158	0,0708299	-5,05617
PAX8	0,206482	0,134307	2,679777	0,020257	0,0610549	-4,88384
eIF4E_pS209	0,204506	0,048507	4,403309	8,89E-04	0,0053993	-1,71025
GATA3	0,204245	-0,050683	2,249395	0,04434	0,1098298	-5,64141
DNA-Ligase-IV	0,202696	0,096328	1,72486	0,11055	0,2127097	-6,48506
Cyclin-B1	0,199964	-5,99E-04	2,47646	0,029398	0,0801925	-5,247
EPHA2	0,197891	-0,649423	4,186227	0,001301	0,0075762	-2,10406
Stathmin-1	0,186112	-0,093131	3,249935	0,007076	0,0307184	-3,83613
p70-S6K_pT389	0,185756	0,269091	1,486528	0,163293	0,2849368	-6,82504
SETD2	0,183254	-0,035564	2,919092	0,013031	0,0456613	-4,44785
Collagen-VI	0,18283	0,063135	2,602209	0,023358	0,0694306	-5,02332
PKC-b-II_pS660	0,182643	0,022917	1,629754	0,129467	0,2374481	-6,62459
ATM	0,182023	0,084602	1,735465	0,108601	0,2117958	-6,4692
14-3-3-beta	0,177872	0,022584	2,057712	0,062348	0,1406908	-5,96235
DUSP4	0,17682	-0,042366	1,63932	0,127443	0,2347347	-6,61078
RIP3	0,169619	-0,015111	2,76372	0,017357	0,0548696	-4,73178
D-a-Tubulin	0,165056	0,01165	2,771548	0,017108	0,0548696	-4,71755
Aurora-A	0,164932	0,026589	2,274496	0,042385	0,106208	-5,59851
N-Ras	0,160593	-0,002013	2,913742	0,01316	0,0457428	-4,45767

MGT4 + A11 + ADAV vs. MGT4 + ADAV

Protein	logFC	AveExpr	t	P.Value	adj.P.Val	B
PAR	1,77538	-0,447016	25,64729	9,77E-12	4,21E-09	17,41064
Histone-H3	1,24939	-0,22751	16,08338	2,11E-09	4,54E-07	12,17029
Caspase-3-	0,923867	-0,157487	10,97441	1,48E-07	1,60E-05	7,791781
Caspase-7-	0,852249	0,044123	12,89506	2,52E-08	3,63E-06	9,630045
DM-Histon	0,679048	0,044083	9,941275	4,30E-07	3,70E-05	6,681096
KAP1	0,640602	-0,155818	3,035028	0,010519	0,1237839	-3,82686
HES1	0,614045	-0,136402	2,333775	0,038089	0,2534579	-5,08557
Histone-H3	0,476422	0,032165	6,248991	4,54E-05	0,002023	1,790387
H2AX_pS14	0,413435	-0,061435	8,497446	2,22E-06	1,60E-04	4,957271
H2AX_pS13	0,399667	-0,076421	6,46336	3,31E-05	0,0017839	2,120802
ER-a_pS11	0,368771	-0,029565	3,97797	0,001883	0,0450839	-2,08416
Aurora-AB	0,359293	0,130098	4,511615	7,37E-04	0,0244475	-1,11808
ERRalpha	0,348421	-0,060608	6,226119	4,69E-05	0,002023	1,75476
CREB_pS13	0,316702	0,0246	3,841448	0,002405	0,0518363	-2,33502
Bcl2	0,303528	0,005942	2,554058	0,025512	0,1982695	-4,69945
Cdc6	0,285005	0,183566	4,31231	0,001042	0,0293093	-1,47578
Caveolin-1	0,265426	-0,149569	1,671369	0,120864	0,4616099	-6,15086
Stathmin-1	0,25082	-0,093131	4,37989	9,26E-04	0,0285206	-1,35404
RBM15	0,242631	0,009748	2,625912	0,022364	0,1939187	-4,57124
MLH1	0,241882	-0,022308	3,064759	0,009957	0,1226175	-3,77209
Cyclophilin	0,227372	-0,349202	1,508709	0,157604	0,5031641	-6,38106
Sox2	0,223536	0,345513	3,856503	0,002341	0,0518363	-2,3073
SOD1	0,222717	-0,066911	3,642829	0,003446	0,0550062	-2,70177
Cox2	0,219464	0,165493	1,294413	0,220215	0,553027	-6,65926
c-Myc	0,211255	-0,068852	1,897212	0,082467	0,3664253	-5,80799
SOD2	0,195935	0,043969	2,258454	0,043624	0,2645265	-5,21476
PARP	0,194419	-0,075124	3,713172	0,003033	0,0522848	-2,57168
Caspase-8	0,193593	-0,049334	1,686268	0,117908	0,4616099	-6,12903
Fibronecti	0,193584	0,223085	2,887415	0,013816	0,1452348	-4,0977
S6	0,190408	-0,221281	1,232593	0,241658	0,5808818	-6,73365
PAX8	0,182963	0,134307	2,374533	0,035381	0,2450007	-5,01501
TRAP1	0,178947	-0,205144	1,552559	0,146854	0,4944847	-6,32052
ATP5A	0,175825	-0,041445	1,945699	0,075833	0,3514424	-5,73128
MITF	0,175276	0,104632	3,294253	0,006522	0,0878406	-3,34778
E-Cadherin	0,172151	-0,718665	0,390062	0,703421	0,9060647	-7,42456
Claudin-7	0,166088	-0,262171	1,960017	0,07397	0,3465357	-5,70844
MTCO1	0,161766	0,022592	1,156321	0,270364	0,6298742	-6,82152
GRB7	0,15997	-0,033547	1,97695	0,071822	0,3439474	-5,68132
Caspase-8-	0,159073	-0,011594	2,918004	0,013057	0,1442984	-4,04174
S100A4	0,15705	0,323293	2,0956	0,058317	0,3105905	-5,48809
Mcl-1	0,151739	0,126503	1,999326	0,069071	0,3382926	-5,6453
eEF2K	0,149796	0,045657	1,455442	0,171561	0,5186339	-6,45301
IRF-1	0,149114	0,017763	1,580331	0,140378	0,4944847	-6,28158
DNA_POLG	0,141829	0,048448	1,331618	0,208071	0,5484457	-6,61318
C-Raf_pS3	0,141795	-0,094016	1,754088	0,105252	0,4161802	-6,0282
EGFR	0,141214	0,023382	2,256576	0,043772	0,2645265	-5,21796
Bcl-xL	0,139267	-0,052409	3,339261	0,006004	0,0834711	-3,26437
GATA3	0,138063	-0,050683	1,520525	0,154643	0,502496	-6,36486
Mitofusin-	0,136821	0,041273	0,781969	0,449599	0,8074041	-7,18398
SDHA	0,132624	-0,103483	1,987231	0,070546	0,3416321	-5,66479
Bid	0,127081	0,009543	1,777647	0,101149	0,4074308	-5,99263
b-Actin	0,124978	-0,123301	1,32571	0,209962	0,5484457	-6,62056
Annexin-I	0,12349	-0,035592	1,300454	0,218205	0,553027	-6,65185
ACC_pS79	0,119903	-0,237276	1,313618	0,213876	0,5519799	-6,6356
Cox-IV	0,119143	-0,046332	0,595235	0,562898	0,8406864	-7,31756
Annexin-VI	0,118431	-0,018589	1,565464	0,143813	0,4944847	-6,30248
SF2	0,115018	-0,07376	0,953722	0,359303	0,7221004	-7,03256
IGFBP2	0,10919	-0,058578	1,465885	0,168745	0,5158084	-6,43905
Transgluta	0,108853	0,012303	0,825141	0,425615	0,787296	-7,14842
Tyro3	0,108323	0,007668	1,881758	0,084691	0,3724655	-5,83223
Heregulin	0,107308	0,529459	1,337036	0,206349	0,5484457	-6,60639
DM-K9-His	0,105642	0,012197	1,29297	0,220698	0,553027	-6,66103
Cyclin-B1	0,103239	-5,99E-04	1,278566	0,22556	0,5561111	-6,67859
Coup-TFII	0,102543	0,178384	1,444853	0,174457	0,5186339	-6,46711
PARG	0,101702	2,89E-04	1,46844	0,168062	0,5158084	-6,43562
Collagen-V	0,100183	0,063135	1,425896	0,179744	0,5199318	-6,49215
YAP	0,098425	0,05181	1,853687	0,08887	0,3783138	-5,87597
XRCC1	0,097395	-0,039169	1,241531	0,238459	0,5773912	-6,72306
Aurora-A	0,097341	0,026589	1,342384	0,20466	0,5484457	-6,59966
TFAM	0,096845	0,020213	1,434267	0,177393	0,5186339	-6,48112

Notch1-cleaved	0,158484	-0,006006	3,237015	0,007247	0,0307184	-3,86013
Bid	0,156599	0,009543	2,19056	0,049264	0,1186182	-5,74123
DNMT1	0,154231	-0,244014	2,403719	0,033557	0,0886222	-5,3748
Notch3	0,153731	-0,012206	3,150047	0,008507	0,0339491	-4,02151
PARG	0,149382	2,89E-04	2,156876	0,052311	0,1232036	-5,79787
CDT1	0,148304	0,052388	2,370184	0,035661	0,0920362	-5,43329
c-Myc	0,147521	-0,068852	1,324838	0,210242	0,3481555	-7,03587
YB1_pS102	0,145909	-0,035683	2,504083	0,027953	0,0787308	-5,19815
Src	0,145128	-0,207681	2,113457	0,056503	0,1309293	-5,87032
YAP	0,144603	0,05181	2,723384	0,018695	0,0575552	-4,80498
Cdc42	0,143783	0,025328	3,121586	0,008966	0,0351291	-4,07425
Transglutaminase	0,143116	0,012303	1,084873	0,299588	0,4421999	-7,3145
CD49b	0,14114	-0,01632	3,030203	0,010613	0,0390462	-4,24324
COG3	0,140287	0,05772	2,201553	0,048306	0,1169655	-5,72266
ACC_pS79	0,137803	-0,237276	1,509724	0,157347	0,2756776	-6,79341
ACVRL1	0,136731	-2,20E-04	2,577992	0,024419	0,0710245	-5,06665
Syk	0,135082	0,002397	2,203825	0,04811	0,1169655	-5,71882
Annexin-I	0,130742	-0,035592	1,37682	0,19406	0,3305924	-6,96999
MSI2	0,130695	-0,049706	1,753761	0,10531	0,2072541	-6,44171
Enolase-1	0,130538	-0,337237	1,671938	0,12075	0,2272622	-6,5633
Rb	0,129048	0,015017	2,10733	0,057119	0,1316497	-5,88049
MERIT40_pS29	0,127443	-0,058898	1,981759	0,071222	0,1543987	-6,08584
PR	0,124794	0,032851	1,604207	0,135011	0,2455262	-6,66122
FOXM1	0,120926	0,001348	2,420301	0,032562	0,0871685	-5,34578
S6_pS240_S244	0,116998	0,524502	0,984701	0,344479	0,4821139	-7,41742
PYGM	0,115132	-0,031874	1,512618	0,156619	0,2755208	-6,78944
p21	0,115076	0,016374	1,64396	0,126471	0,233945	-6,60406
ATR_pS428	0,113369	-0,007961	1,726828	0,110186	0,2127097	-6,48212
CA9	0,112877	0,007759	1,93114	0,077772	0,1643119	-6,16685
CD4	0,11198	0,001958	0,807017	0,435579	0,5758734	-7,57877
CtIP	0,111624	-0,049514	2,168387	0,05125	0,1220383	-5,77856
XBP-1	0,111304	0,073656	2,002886	0,068643	0,1517183	-6,05172
GATA6	0,11099	0,007955	1,729934	0,109613	0,2127097	-6,47748
MRAP	0,11001	0,030253	1,90027	0,082033	0,171633	-6,21572
VHL	0,10966	0,077473	1,514189	0,156224	0,2755208	-6,78729
DAPK1_pS308	0,108951	0,013646	1,995388	0,069548	0,1521588	-6,06385
BiP-GRP78	0,106921	0,01833	1,916699	0,079739	0,1676473	-6,18977
Rheb	0,106119	-0,006062	2,004555	0,068443	0,1517183	-6,04902
4E-BP1	0,105809	-0,121582	1,78429	0,100018	0,2014382	-6,39547
Chk1_pS296	0,104451	0,030022	1,066203	0,307605	0,4488914	-7,33431
HER3	0,099278	-0,121268	2,08075	0,059867	0,1365218	-5,92445
PRAS40	0,096575	-0,009817	1,849889	0,08945	0,1844629	-6,29457
Complex-II-Subunit	0,093413	-0,044895	1,761166	0,104004	0,206595	-6,43054
Rad50	0,09257	-0,199697	1,149163	0,273189	0,4160578	-7,24415
DNA_POLG	0,091061	0,048448	0,854964	0,409549	0,553341	-7,538
IGFBP2	0,090832	-0,058578	1,219425	0,246434	0,3849555	-7,16359
PD-L1	0,089645	0,04206	0,910622	0,380672	0,5241836	-7,48808
NAPSIN-A	0,089171	-0,101967	1,385731	0,191392	0,3275377	-6,95851
Chk2_pT68	0,087271	-0,002757	1,324465	0,210362	0,3481555	-7,03633
14-3-3-zeta	0,086245	-0,152677	1,296713	0,219448	0,3583302	-7,07073
Aurora-B	0,085383	0,031936	1,371811	0,195573	0,3318584	-6,97642
CIITA	0,084159	-0,028549	1,312669	0,214186	0,3523437	-7,05102
BAP1	0,08283	0,003482	1,65086	0,125039	0,233297	-6,59405
Cyclin-D1	0,081793	0,034803	1,015438	0,330212	0,4728286	-7,38673
VEGF-164	0,081308	0,03077	1,646642	0,125913	0,2339157	-6,60017
IL-6	0,077952	-0,01241	1,323006	0,210832	0,3481555	-7,03815
CD31	0,077369	0,01401	1,611638	0,133377	0,2435831	-6,65061
PDH	0,074612	0,038497	1,3871	0,190985	0,3275377	-6,95674
HSP27	0,07056	-0,024798	1,145432	0,27467	0,4168409	-7,24832
MIF	0,06894	-0,020019	1,455836	0,171454	0,2967739	-6,86638
Bim	0,06879	-0,479038	0,546765	0,594702	0,7184027	-7,76222
DM-K9-Histone-H3	0,065462	0,012197	0,801204	0,438807	0,5783662	-7,58357
PKC-a-b-II_pT638_T641	0,06133	0,193816	1,012725	0,331454	0,473035	-7,38947
MEK2	0,061318	-0,028089	1,201854	0,252923	0,3893209	-7,18409
HSP70	0,061097	-0,104212	1,115365	0,286835	0,4307522	-7,28154
Calnexin	0,060677	-0,064072	1,069006	0,306391	0,4488914	-7,33135
SOX17	0,060094	-0,001645	1,23338	0,241375	0,382473	-7,14715
GCLM	0,058653	0,022262	1,009472	0,332947	0,4735979	-7,39275
Bak	0,058551	-0,029684	1,035187	0,321276	0,4646641	-7,36659
FASN	0,055383	-0,105082	0,906937	0,382539	0,5250773	-7,49147
Hexokinase-I	0,055313	0,008558	1,064861	0,308187	0,4488914	-7,33572
PAI-1	0,055135	-0,036353	0,850796	0,41177	0,5546027	-7,54163
Patched	0,053392	-0,044418	0,814183	0,431622	0,5723966	-7,57281
TFAM	0,053257	0,020213	0,788736	0,445783	0,5857693	-7,59376
Smad4	0,052687	-0,014953	0,936875	0,367553	0,5081363	-7,46358
CDKN2A	0,051102	0,017704	0,593121	0,564265	0,6988459	-7,73437
AceCS1	0,05108	0,175013	0,574185	0,576596	0,7040022	-7,746

Lck	0,095801	-0,0272	1,382813	0,192263	0,5355361	-6,5482
CD44	0,091911	-0,048103	1,116863	0,28622	0,6492664	-6,86522
DUSP4	0,090852	-0,042366	0,8423	0,416321	0,7788793	-7,13381
GATA6	0,08737	0,007955	1,361785	0,198631	0,5484457	-6,5751
Vinculin	0,081391	-0,081115	0,307234	0,764006	0,9216856	-7,45546
COG3	0,079435	0,05772	1,246599	0,236659	0,5762717	-6,71704
Chk1_pS29	0,079142	0,030022	0,807856	0,435114	0,7946369	-7,16286
Hexokinase	0,077651	-0,321777	0,604989	0,556611	0,8406864	-7,31142
Ambra1_pS	0,076344	0,096777	0,990665	0,341677	0,7012504	-6,99661
Cdc42	0,075523	0,025328	1,639634	0,127377	0,4628801	-6,19695
CD171	0,074913	-0,179758	0,339746	0,740003	0,9216856	-7,44419
CD86	0,074342	-0,059701	1,297426	0,219211	0,553027	-6,65557
Enolase-1	0,074317	-0,337237	0,951851	0,360212	0,7221004	-7,03435
Notch1-cle	0,074168	-0,006006	1,514867	0,156054	0,502496	-6,37263
YAP_pS127	0,073161	0,902732	0,610637	0,552989	0,8406864	-7,30782
RPA32	0,071498	-0,242082	1,134492	0,27905	0,6431574	-6,84585
DNA-Ligase	0,071404	0,096328	0,607623	0,554921	0,8406864	-7,30974
c-Jun_pS73	0,070647	-0,075111	0,926444	0,372726	0,7369042	-7,05836
D-a-Tubulir	0,070607	0,01165	1,185596	0,259046	0,6101032	-6,78831
4E-BP1	0,06702	-0,121582	1,130174	0,280793	0,643733	-6,85062
FN14	0,067017	0,021789	0,89254	0,389895	0,7549078	-7,08951
U-Histone-	0,066739	0,011354	0,599676	0,560031	0,8406864	-7,31477
SETD2	0,065918	-0,035564	1,050022	0,314682	0,676246	-6,93644
Grp75	0,064968	-0,171403	1,010149	0,332636	0,6925892	-6,97718
SOX17	0,064347	-0,001645	1,320654	0,211591	0,5493724	-6,62686
RIP3	0,063802	-0,015111	1,039567	0,319318	0,676246	-6,94725
Snail	0,06212	0,389254	0,840201	0,41745	0,7788793	-7,13561
CDKN2A	0,061682	0,017704	0,715931	0,487923	0,838916	-7,23503
Tuberin_p1	0,061148	0,059566	0,778142	0,451765	0,8079288	-7,18705
MERIT40_p	0,05818	-0,058898	0,904712	0,383669	0,7529167	-7,07844
Aurora-B	0,057969	0,031936	0,931361	0,370281	0,7354429	-7,05376
p27-Kip1	0,057885	0,01314	0,643284	0,53231	0,8406864	-7,2864
PREX1	0,05325	0,008897	0,796223	0,441585	0,7988712	-7,17243
VHL-EPPK1	0,052152	-0,032711	0,230596	0,821568	0,9334929	-7,47761
ADAR1	0,050936	-0,023956	0,634024	0,53813	0,8406864	-7,29258
AR	0,050638	0,027125	0,903435	0,384319	0,7529167	-7,07961
EPHA2	0,049966	-0,649423	1,05699	0,31162	0,6761695	-6,92918
Rad50	0,049587	-0,199697	0,615577	0,549831	0,8406864	-7,30464
N-Ras	0,048613	-0,002013	0,88201	0,395336	0,7549078	-7,09898
Wee1_pS6	0,048258	0,077241	0,862498	0,405556	0,7632947	-7,11627
cdc25C	0,047852	0,066872	0,665306	0,518614	0,8406864	-7,27136
IR-b	0,047754	-0,023481	0,437321	0,669754	0,8984516	-7,40372
G6PD	0,046291	-0,105384	0,479332	0,64044	0,8984516	-7,38327
Hexokinase	0,046108	0,008558	0,887644	0,392419	0,7549078	-7,09393
BiP-GRP78	0,045527	0,01833	0,816135	0,430547	0,7896418	-7,15598
SLC1A5	0,044073	-0,020076	0,337309	0,741792	0,9216856	-7,44507
Tau	0,043839	0,023815	0,631406	0,539782	0,8406864	-7,29431
SCD	0,042921	0,046091	0,293097	0,774525	0,9216856	-7,46002
CD4	0,04268	0,001958	0,307589	0,763742	0,9216856	-7,45534
Hif-1-alpha	0,041821	0,203091	0,392344	0,70178	0,9060647	-7,42361
HSP70	0,041687	-0,104212	0,761019	0,461544	0,8134175	-7,20062
PRAS40	0,041436	-0,009817	0,793706	0,442994	0,7988712	-7,17448
Bim	0,040894	-0,479038	0,325037	0,750828	0,9216856	-7,44943
Rb	0,039722	0,015017	0,648649	0,528954	0,8406864	-7,28278
PD-1	0,038567	0,012291	0,678756	0,510351	0,8406864	-7,26195
Complex-II-	0,038169	-0,044895	0,719624	0,485728	0,838916	-7,23228
GCLM	0,037951	0,022262	0,653179	0,52613	0,8406864	-7,2797
Syk	0,03754	0,002397	0,612455	0,551825	0,8406864	-7,30665
P-Cadherin	0,037431	0,004036	0,400759	0,69574	0,9060647	-7,42005
CD49b	0,037159	-0,01632	0,797779	0,440716	0,7988712	-7,17116
HSP27	0,036715	-0,024798	0,596016	0,562393	0,8406864	-7,31707
Cyclin-E1	0,036164	0,131132	0,413665	0,686518	0,9058509	-7,41444
Myosin-IIa	0,034994	0,125587	0,140632	0,890526	0,9453616	-7,49561
STING	0,034502	0,244125	0,152961	0,881006	0,9446507	-7,49366
Cyclin-D3	0,034441	0,043783	0,46426	0,650887	0,8984516	-7,39081
FOXM1	0,033707	0,001348	0,674636	0,512873	0,8406864	-7,26485
Merlin	0,032685	-0,057375	0,380642	0,710215	0,9083162	-7,42844
Notch3	0,030738	-0,012206	0,62983	0,540778	0,8406864	-7,29535
MEK1	0,030651	-0,124464	0,435224	0,671233	0,8984516	-7,4047
RRM1	0,030246	-0,036564	0,552337	0,590999	0,8634592	-7,34345
BAP1	0,029835	0,003482	0,594628	0,56329	0,8406864	-7,31794
PD-L1	0,029369	0,04206	0,298333	0,770623	0,9216856	-7,45835
CDT1	0,028754	0,052388	0,459549	0,654169	0,8984516	-7,39312
CD29	0,028668	0,008186	0,539145	0,599785	0,8733357	-7,35104
LRP6_pS14	0,027337	0,099524	0,369061	0,718602	0,9115925	-7,43307
CA9	0,027105	0,007759	0,463726	0,651259	0,8984516	-7,39108
ERCC5	0,026896	0,04876	0,306631	0,764453	0,9216856	-7,45566

c-Abl	0,048813	0,06195	0,666521	0,517864	0,6584053	-7,68593
AR	0,047109	0,027125	0,840475	0,417303	0,5603042	-7,55054
PI3K-p110-b	0,044352	-0,043716	0,696791	0,499395	0,640593	-7,66442
DAPK2	0,041487	0,006769	0,828256	0,423917	0,5639149	-7,56097
MelanA	0,040197	-0,07913	0,498708	0,627127	0,7364894	-7,78881
E2F1	0,039477	-0,015219	0,67728	0,511254	0,6519245	-7,67838
N-Cadherin	0,039105	0,036274	0,749226	0,468355	0,6080152	-7,6251
SDHA	0,037689	-0,103483	0,564726	0,582808	0,709577	-7,75168
UQCRC2	0,036529	-0,059451	0,777295	0,452246	0,5906607	-7,60299
WIPI2	0,03641	-0,02897	0,276899	0,786636	0,857131	-7,88071
HLA-DR-DP-DQ-DX	0,035343	-0,01581	0,62175	0,5459	0,6870819	-7,7161
PD-1	0,034964	0,012291	0,615337	0,549984	0,6870819	-7,72026
IRS2	0,03452	0,497198	0,366643	0,720358	0,8191933	-7,84971
Jagged1	0,034389	-0,002552	0,762569	0,460653	0,5998229	-7,61468
BRD4	0,034241	-0,067661	0,390973	0,702766	0,8034269	-7,83985
14-3-3-epsilon	0,033517	0,009189	0,537422	0,600937	0,7234746	-7,76757
YAP_pS127	0,03327	0,902732	0,277689	0,786044	0,857131	-7,88048
Cox-IV	0,03326	-0,046332	0,166166	0,870832	0,9148983	-7,90717
XPA	0,03231	0,037377	0,531151	0,605142	0,7256483	-7,77111
Bim_1	0,031572	-0,20752	0,385155	0,706957	0,8060805	-7,84226
CD134	0,030915	-5,21E-04	0,462164	0,652347	0,7598955	-7,80746
Cdc6	0,029744	0,183566	0,450054	0,660806	0,7676748	-7,81334
Gli1	0,026904	0,045725	0,472737	0,645001	0,7533757	-7,8022
PDHK1	0,026736	-0,003486	0,43086	0,674315	0,7812626	-7,82235
Histone-H3_pS10	0,025865	0,032165	0,339264	0,740356	0,8288143	-7,86007
XRCC1	0,025248	-0,039169	0,321849	0,753182	0,8388149	-7,86625
CD38	0,025135	-0,02424	0,30849	0,763073	0,8454618	-7,87077
MEK1_p_S217_S221	0,022659	0,022895	0,349353	0,732963	0,8248228	-7,85634
Grp75	0,019198	-0,171403	0,298493	0,770504	0,8515054	-7,87403
TFRC	0,018874	-0,023369	0,199007	0,845636	0,90023	-7,90069
Enolase-2	0,018453	-7,87E-04	0,278176	0,785679	0,857131	-7,88033
SFRP1	0,018111	0,054441	0,343537	0,737222	0,8274549	-7,85851
S100A4	0,017364	0,323293	0,231702	0,820728	0,8821289	-7,89309
Myt1	0,01455	-0,055936	0,242365	0,812647	0,8756272	-7,89037
AMPK-a2_pS345	0,012675	-0,111954	0,111822	0,912839	0,9367472	-7,91533
Ets-1	0,010683	-0,022047	0,226562	0,824631	0,8841192	-7,89436
P-Cadherin	0,008282	0,004036	0,088668	0,930829	0,9484329	-7,91785
TAZ	0,004935	0,101754	0,093992	0,926689	0,946452	-7,91732
UGT1A	0,004202	0,268485	0,061073	0,952321	0,963498	-7,92008
TUFM	0,002065	0,039828	0,025695	0,979929	0,9844974	-7,92174
CD29	0,001455	0,008186	0,027363	0,978626	0,9844974	-7,9217
Granzyme-B	-1,43E-05	0,194614	-1,67E-04	0,99987	0,9998699	-7,9221
CD44	-0,00108	-0,048103	-0,01308	0,989782	0,9920842	-7,92201
PREX1	-0,00274	0,008897	-0,04091	0,968053	0,9771211	-7,9212
CD20	-0,00443	-0,016963	-0,06167	0,951859	0,963498	-7,92004
Creb	-0,00689	-0,033283	-0,11874	0,907476	0,9366229	-7,91447
Rab11	-0,00727	-0,004335	-0,11758	0,908372	0,9366229	-7,91462
NDUFB4	-0,00742	-0,047676	-0,0856	0,933217	0,9486234	-7,91813
Glutaminase	-0,00773	0,32211	-0,10419	0,918765	0,9405882	-7,91623
LC3A-B	-0,00793	-0,040287	-0,15307	0,880923	0,9215483	-7,90943
RIP	-0,00839	0,030057	-0,12257	0,904506	0,9366229	-7,91397
p27_pT157	-0,00864	0,027337	-0,16407	0,872444	0,9148983	-7,90754
Puma	-0,01034	-0,083658	-0,12816	0,900173	0,9348782	-7,91321
MMP2	-0,01059	-0,02074	-0,18605	0,855555	0,9050349	-7,90338
CD45	-0,01091	-0,03593	-0,13195	0,897239	0,934082	-7,91268
Notch1	-0,01174	-0,054826	-0,1945	0,849086	0,9013696	-7,90165
HSP60	-0,01259	-0,120894	-0,19863	0,845924	0,90023	-7,90077
NQO1	-0,01405	-0,013101	-0,11318	0,911783	0,9367472	-7,91517
Glutamate-D1-2	-0,01573	0,097722	-0,357	0,727381	0,8206834	-7,85345
U-Histone-H2B	-0,01654	0,011354	-0,14862	0,884354	0,9228969	-7,91015
PAK1	-0,01795	0,121987	-0,31199	0,760475	0,8447543	-7,8696
Lck	-0,0183	-0,0272	-0,26408	0,796261	0,8622829	-7,88444
TRIP13	-0,01846	-0,047949	-0,27571	0,787526	0,857131	-7,88107
ERCC5	-0,01861	0,04876	-0,21214	0,835612	0,8936689	-7,89778
FOXO3	-0,01893	0,039835	-0,18357	0,857459	0,9050349	-7,90388
LRP6_pS1490	-0,02046	0,099524	-0,27617	0,787181	0,857131	-7,88093
ATM_pS1981	-0,022	-0,044366	-0,26952	0,792169	0,860012	-7,88288
c-Kit	-0,02353	-0,017567	-0,3334	0,744666	0,8314791	-7,86219
SCD	-0,02662	0,046091	-0,18177	0,858838	0,9050349	-7,90423
Porin	-0,02718	-0,05308	-0,583	0,570837	0,7018063	-7,74063
RPA32_pS4_S8	-0,02793	0,003	-0,42086	0,6814	0,7857182	-7,82689
INPP4b	-0,02835	-0,042678	-0,57718	0,574635	0,7036018	-7,74418
VASP	-0,02857	0,087681	-0,42029	0,681807	0,7857182	-7,82715
Mnk1	-0,02874	0,189059	-0,35786	0,726748	0,8206834	-7,85312
p38-a	-0,03069	-0,037713	-0,59052	0,565952	0,6989258	-7,73599
ENY2	-0,03274	-0,11689	-0,54623	0,595057	0,7184027	-7,76252
Beclin	-0,03315	0,065552	-0,72247	0,484039	0,6246131	-7,64549

Mnk1	0,026279	0,189059	0,327209	0,749226	0,9216856	-7,44867
PR	0,024936	0,032851	0,320548	0,754143	0,9216856	-7,45098
XBP-1	0,024663	0,073656	0,4438	0,665194	0,8984516	-7,40069
HER3	0,024184	-0,121268	0,506872	0,621558	0,8900047	-7,36888
MEK1_p_S	0,024074	0,022895	0,371172	0,71707	0,9115925	-7,43224
PDH	0,023612	0,038497	0,438967	0,668594	0,8984516	-7,40295
Chk1	0,022605	-0,197309	0,395465	0,699537	0,9060647	-7,42229
ATR_pS428	0,022486	-0,007961	0,342503	0,73798	0,9216856	-7,44318
NAPSIN-A	0,021669	-0,101967	0,336743	0,742208	0,9216856	-7,44528
MRAP	0,019173	0,030253	0,33119	0,746293	0,9216856	-7,44726
A-Raf_pS29	0,018922	0,023098	0,284755	0,780755	0,9216856	-7,4626
NRF2	0,018762	-0,109994	0,230723	0,821471	0,9334929	-7,47758
eIF4E	0,017942	0,084361	0,279318	0,784823	0,9216856	-7,46425
14-3-3-beta	0,01776	0,022584	0,205453	0,840711	0,9334929	-7,48351
PDHK1	0,017523	-0,003486	0,282392	0,782522	0,9216856	-7,46332
PYGM	0,015961	-0,031874	0,209693	0,837474	0,9334929	-7,48256
BRD4	0,015721	-0,067661	0,179509	0,860576	0,9390078	-7,48889
N-Cadherin	0,013995	0,036274	0,268145	0,793206	0,9236947	-7,46754
MIG6	0,013683	-0,034288	0,254587	0,803413	0,9283403	-7,47135
c-Abl	0,013308	0,06195	0,181709	0,858887	0,9390078	-7,48847
Src	0,012614	-0,207681	0,183699	0,85736	0,9390078	-7,48807
VHL	0,012525	0,077473	0,172944	0,865619	0,9407674	-7,49014
DAPK1_pS3	0,012352	0,013646	0,226228	0,824885	0,9334929	-7,47868
INPP4b	0,012192	-0,042678	0,248189	0,808243	0,9314245	-7,47308
IRS1	0,011322	0,130579	0,149376	0,883773	0,9446507	-7,49424
MIF	0,010669	-0,020019	0,225312	0,825581	0,9334929	-7,4789
Chk2	0,009345	0,017318	0,079352	0,938079	0,9695736	-7,50289
Jagged1	0,009333	-0,002552	0,206953	0,839566	0,9334929	-7,48318
Bak	0,008164	-0,029684	0,144345	0,887658	0,9446507	-7,49504
UQCRC2	0,007903	-0,059451	0,168173	0,869287	0,9407674	-7,49102
MelanA	0,007046	-0,07913	0,087413	0,931806	0,9677306	-7,50216
Calnexin	0,006476	-0,064072	0,114087	0,911082	0,9634726	-7,49926
Rab11	0,006038	-0,004335	0,097628	0,923862	0,9664673	-7,50114
IL-6	0,002941	-0,01241	0,04991	0,961027	0,9815226	-7,50495
4E-BP1_pS	0,002867	0,087254	0,06086	0,952486	0,9797651	-7,50429
Ets-1	0,002202	-0,022047	0,0467	0,963531	0,9817537	-7,50511
HLA-DR-DP	0,002019	-0,01581	0,035511	0,972264	0,98338	-7,50561
Glutaminas	0,001874	0,32211	0,02525	0,980277	0,9848467	-7,50595
CtIP	0,001697	-0,049514	0,032963	0,974253	0,98338	-7,5057
c-Abl_pY41	0,001207	0,027149	0,015088	0,988214	0,9905119	-7,50617
PEA-15	-9,00E-04	0,046253	-0,01204	0,990591	0,9905914	-7,50621
TUFM	-0,00207	0,039828	-0,02577	0,979869	0,9848467	-7,50593
MMP14	-0,00221	-0,048973	-0,03419	0,973293	0,98338	-7,50566
Bad_pS112	-0,00291	0,10143	-0,06122	0,952205	0,9797651	-7,50427
Bim_1	-0,00343	-0,20752	-0,04185	0,967317	0,983287	-7,50535
p21	-0,00365	0,016374	-0,05213	0,959296	0,9815226	-7,50482
PEA-15_pS	-0,00498	-0,061818	-0,05254	0,958974	0,9815226	-7,5048
MMP2	-0,00527	-0,02074	-0,09262	0,927756	0,9677306	-7,50166
MTSS1	-0,0055	0,031705	-0,08813	0,931247	0,9677306	-7,50209
GCN5L2	-0,00582	-0,152105	-0,11148	0,913107	0,9634726	-7,49958
Notch1	-0,006	-0,054826	-0,09932	0,922543	0,9664673	-7,50096
CD134	-0,00669	-5,21E-04	-0,10006	0,921969	0,9664673	-7,50088
DAPK2	-0,00723	0,006769	-0,14434	0,887665	0,9446507	-7,49504
Atg3	-0,00855	0,143336	-0,18255	0,858238	0,9390078	-7,4883
VAV1	-0,00942	-0,053778	-0,08274	0,935443	0,9691734	-7,50259
TRIP13	-0,01005	-0,047949	-0,15009	0,883224	0,9446507	-7,49413
UVRAG	-0,01046	0,098398	-0,16605	0,870919	0,9407674	-7,4914
TAZ	-0,01086	0,101754	-0,20688	0,83962	0,9334929	-7,48319
Connexin-4	-0,01109	-0,052007	-0,20308	0,842526	0,9334929	-7,48403
Chk2_pT68	-0,01126	-0,002757	-0,17091	0,86718	0,9407674	-7,49052
PCNA	-0,01142	-0,015273	-0,22479	0,825977	0,9334929	-7,47903
HSP60	-0,01235	-0,120894	-0,1948	0,848858	0,9380966	-7,48581
14-3-3-eps	-0,01304	0,009189	-0,20904	0,83797	0,9334929	-7,48271
PLK1	-0,01322	-0,086862	-0,10995	0,914293	0,9634726	-7,49976
cdc2_pY15	-0,01332	-0,23913	-0,18235	0,858397	0,9390078	-7,48834
TTF1	-0,01375	-0,024739	-0,21028	0,83703	0,9334929	-7,48243
LDHA	-0,01514	-0,094454	-0,15648	0,878295	0,9446507	-7,49307
PAI-1	-0,01557	-0,036353	-0,24027	0,814234	0,9334929	-7,47516
PI3K-p85	-0,01773	0,139813	-0,29183	0,775467	0,9216856	-7,46041
Smad1	-0,01782	0,042839	-0,29273	0,7748	0,9216856	-7,46013
MSH6	-0,018	-0,01407	-0,26778	0,793476	0,9236947	-7,46764
LAD1	-0,01827	-0,060637	-0,28425	0,781131	0,9216856	-7,46276
ENY2	-0,01914	-0,11689	-0,31929	0,755076	0,9216856	-7,45141
Rheb	-0,0195	-0,006062	-0,36834	0,719122	0,9115925	-7,43336
E2F1	-0,02116	-0,015219	-0,3631	0,722936	0,9137405	-7,43541
Axl	-0,02181	-0,091902	-0,20745	0,839182	0,9334929	-7,48307
Smac	-0,02201	-0,021718	-0,26562	0,795106	0,9236947	-7,46826

ERCC1	-0,03329	-0,05099	-0,60719	0,555201	0,6906695	-7,72549
MSH6	-0,03372	-0,01407	-0,5017	0,62508	0,7364894	-7,78722
XPF	-0,03464	0,014147	-0,65202	0,52685	0,6659012	-7,69591
PEA-15	-0,03548	0,046253	-0,47475	0,643608	0,7533757	-7,80119
MEK1	-0,03586	-0,124464	-0,50914	0,620013	0,7361582	-7,78323
A-Raf_pS299	-0,03724	0,023098	-0,56041	0,585652	0,711031	-7,75424
TRIM25	-0,03884	-0,075383	-0,29147	0,775736	0,8550956	-7,87626
eIF4E	-0,03966	0,084361	-0,61738	0,548679	0,6870819	-7,71894
4E-BP1_pS65	-0,0405	0,087254	-0,85972	0,407028	0,5516632	-7,53385
NRF2	-0,04068	-0,109994	-0,50021	0,626098	0,7364894	-7,78801
PDHA1	-0,04123	-0,038326	-0,58192	0,571541	0,7018063	-7,74129
FAK	-0,046	-0,154643	-0,60585	0,556061	0,6906695	-7,72635
Atg3	-0,04635	0,143336	-0,98955	0,3422	0,4821139	-7,41263
Chk2	-0,04795	0,017318	-0,40719	0,691138	0,7922348	-7,83293
G6PD	-0,0489	-0,105384	-0,50634	0,62192	0,7363942	-7,78474
Bax	-0,04902	-0,033866	-0,94164	0,365208	0,5077567	-7,45908
VEGFR-2	-0,05228	0,135944	-0,51965	0,612894	0,729716	-7,77751
CD86	-0,05365	-0,059701	-0,93629	0,367839	0,5081363	-7,46413
UVRAG	-0,05463	0,098398	-0,86756	0,402887	0,547774	-7,52695
C-Raf	-0,05464	-0,023119	-0,9846	0,344527	0,4821139	-7,41752
Gli3	-0,05536	2,76E-05	-0,89343	0,389435	0,5328465	-7,50379
Axl	-0,05542	-0,091902	-0,52721	0,607794	0,7256483	-7,77332
FoxO3a_pS318_S321	-0,05552	0,075277	-0,87614	0,398394	0,5433789	-7,51934
p27-Kip1	-0,05562	0,01314	-0,61814	0,548194	0,6870819	-7,71845
b-Catenin_pT41_S45	-0,05894	-0,033569	-1,02025	0,328017	0,4712516	-7,38185
FN14	-0,06271	0,021789	-0,83517	0,420168	0,5623988	-7,55509
Smac	-0,06479	-0,021718	-0,78191	0,449629	0,5890278	-7,59928
PKC-delta_pS664	-0,06491	-0,045451	-1,1406	0,276599	0,4182959	-7,25371
MTSS1	-0,06642	0,031705	-1,06452	0,308337	0,4488914	-7,33608
RRM1	-0,06676	-0,036564	-1,2192	0,246514	0,3849555	-7,16385
PEA-15_pS116	-0,06865	-0,061818	-0,72444	0,482873	0,6246131	-7,64401
oct-04	-0,06945	-0,096692	-0,24856	0,807962	0,8727614	-7,88873
HER2	-0,07422	-0,099699	-0,52788	0,607338	0,7256483	-7,77294
PI3K-p85	-0,07433	0,139813	-1,22322	0,245051	0,3849555	-7,15914
TTF1	-0,07948	-0,024739	-1,21583	0,247749	0,3854873	-7,1678
PLK1	-0,07955	-0,086862	-0,66161	0,520897	0,6603134	-7,68933
Rictor_pT1135	-0,08016	0,247853	-1,47281	0,166898	0,2900529	-6,84359
Chk1_pS345	-0,08126	0,094896	-1,22724	0,24359	0,3845689	-7,1544
TIGAR	-0,08135	-0,036293	-1,24129	0,238546	0,3799484	-7,13777
GSK-3a-b_pS21_S9	-0,08229	0,350958	-1,03118	0,323074	0,4657027	-7,3707
PDK1_pS241	-0,08381	0,104414	-1,52827	0,152728	0,2742739	-6,76789
LAD1	-0,08522	-0,060637	-1,32578	0,209938	0,3481555	-7,03468
GCLC	-0,0873	-0,02283	-1,21022	0,249816	0,3873051	-7,17436
Smad3	-0,08751	-0,00225	-1,26743	0,229378	0,3702701	-7,10643
PHGDH	-0,08832	-0,245847	-0,68661	0,505559	0,6465758	-7,67175
CD171	-0,09058	-0,179758	-0,41079	0,688567	0,7913926	-7,83136
PCNA	-0,09148	-0,015273	-1,80117	0,097195	0,1970097	-6,3697
Elk1_pS383	-0,09276	0,002093	-1,08983	0,297484	0,4406034	-7,30918
UBQLN4	-0,09283	-0,037809	-1,09481	0,295384	0,4390016	-7,30383
B-Raf_pS445	-0,09538	0,108475	-2,15781	0,052225	0,1232036	-5,79631
TSC1	-0,09703	0,030918	-1,76109	0,104017	0,206595	-6,43065
Tuberin_pT1462	-0,1018	0,059566	-1,29542	0,21988	0,3583302	-7,07232
c-Abl_pY412	-0,10324	0,027149	-1,29055	0,221507	0,3589076	-7,07828
Lasu1	-0,10349	-0,040635	-1,97293	0,072326	0,1558632	-6,10004
Rab25	-0,10376	0,029024	-1,16243	0,267973	0,4095612	-7,22923
BMK1-Erk5_pT218_Y220	-0,10552	0,018014	-1,80016	0,097362	0,1970097	-6,37124
B-Raf	-0,10806	0,001943	-1,25705	0,232982	0,3746839	-7,11892
ATRX	-0,10841	0,050761	-0,98604	0,34385	0,4821139	-7,41611
Cyclin-E1	-0,10864	0,131132	-1,24274	0,238029	0,3799484	-7,13604
PRAS40_pT246	-0,10991	0,125751	-2,13437	0,054446	0,1273271	-5,8355
MR1	-0,11097	0,031104	-0,82882	0,423612	0,5639149	-7,5605
CSK	-0,11147	0,00617	-1,82065	0,094028	0,1929812	-6,33979
Myosin-IIa_pS1943	-0,11326	-0,007141	-0,96548	0,353625	0,4932439	-7,43621
DRP1	-0,11511	0,073839	-2,38655	0,034619	0,0904295	-5,40478
LDHA	-0,11512	-0,094454	-1,18991	0,257411	0,3948196	-7,19789
B7-H3	-0,11647	-0,017587	-1,66719	0,121704	0,2280618	-6,57025
DJ1	-0,11723	-0,237303	-1,20642	0,251223	0,38809	-7,17878
BCL2A1	-0,11938	-0,035601	-1,24029	0,2389	0,3799484	-7,13895
Pdcd4	-0,12498	0,463311	-1,2941	0,220319	0,3583302	-7,07393
ULK1_pS757	-0,12592	0,319938	-2,34976	0,037004	0,0942433	-5,46876
Hexokinase-II	-0,1285	-0,321777	-1,00113	0,3368	0,4775027	-7,40112
PMS2	-0,1285	-0,057549	-1,52496	0,153543	0,2745932	-6,77246
eEF2	-0,12857	0,086276	-1,98123	0,071288	0,1543987	-6,0867
p44-42-MAPK	-0,12865	-0,015364	-1,11057	0,288815	0,4311983	-7,28678
PKCa	-0,12908	0,324233	-2,46911	0,029794	0,0805153	-5,25997
MERIT40	-0,12921	-0,004042	-1,35436	0,200922	0,3382707	-6,99868
PKM2	-0,1303	-0,316375	-2,0262	0,065897	0,1479243	-6,01385

VEGF-164	-0,0225	0,03077	-0,45574	0,656828	0,8984516	-7,39498
PKC-delta_	-0,0231	-0,045451	-0,40587	0,692084	0,9060647	-7,41785
CD38	-0,02375	-0,02424	-0,29152	0,775699	0,9216856	-7,46051
Elk1_pS383	-0,02471	0,002093	-0,29035	0,776576	0,9216856	-7,46088
Myt1	-0,02563	-0,055936	-0,42693	0,677093	0,8993053	-7,4085
YB1_pS102	-0,02588	-0,035683	-0,44413	0,664964	0,8984516	-7,40053
ARID1A	-0,02634	-0,041475	-0,23253	0,820103	0,9334929	-7,47713
Glutamate-	-0,02647	0,097722	-0,60081	0,559302	0,8406864	-7,31406
IGFRb	-0,02756	0,161739	-0,38599	0,706352	0,9060647	-7,42624
CD20	-0,02824	-0,016963	-0,39313	0,701211	0,9060647	-7,42327
CD31	-0,02923	0,01401	-0,60879	0,554171	0,8406864	-7,30899
AMPK-a2_	-0,02925	-0,111954	-0,25807	0,80079	0,9277965	-7,47039
Stat3	-0,0301	0,455546	-0,26584	0,79494	0,9236947	-7,4682
A-Raf	-0,03046	-0,001133	-0,48284	0,63802	0,8984516	-7,38148
PI3K-p110-	-0,03069	-0,043716	-0,48218	0,638473	0,8984516	-7,38181
XPA	-0,03157	0,037377	-0,51895	0,613365	0,8841479	-7,36233
FASN	-0,03189	-0,105082	-0,52224	0,611144	0,8839029	-7,36052
c-Kit	-0,0321	-0,017767	-0,45476	0,65751	0,8984516	-7,39545
DNMT1	-0,03266	-0,244014	-0,50902	0,620094	0,8900047	-7,36772
FGF-basic	-0,03273	0,070481	-0,46194	0,652506	0,8984516	-7,39196
PDK1_pS24	-0,03406	0,104414	-0,62099	0,546383	0,8406864	-7,30114
b-Catenin_	-0,03481	-0,033569	-0,60254	0,558185	0,8406864	-7,31296
Creb	-0,03563	-0,033283	-0,61429	0,550651	0,8406864	-7,30547
Smad4	-0,03589	-0,014953	-0,6382	0,535498	0,8406864	-7,2898
Atg7	-0,03594	-0,03646	-0,41261	0,68727	0,9058509	-7,41491
Puma	-0,03601	-0,083658	-0,44623	0,663488	0,8984516	-7,39954
SFRP1	-0,03692	0,054441	-0,7004	0,497222	0,840403	-7,24644
TRIM25	-0,03733	-0,075383	-0,28018	0,784178	0,9216856	-7,46399
PHGDH	-0,03738	-0,245847	-0,2906	0,776386	0,9216856	-7,4608
XIAP	-0,0377	0,151229	-0,57579	0,575542	0,8467108	-7,32952
PKC-a-b-II_	-0,03815	0,193816	-0,63003	0,54065	0,8406864	-7,29522
RRM2	-0,03856	0,068136	-0,42793	0,676386	0,8993053	-7,40805
14-3-3-zeta	-0,03932	-0,152677	-0,59116	0,565537	0,8406864	-7,3201
Cyclin-D1	-0,03934	0,034803	-0,48836	0,63422	0,8984516	-7,37864
Melanoma	-0,04041	-0,02963	-0,61095	0,552792	0,8406864	-7,30762
Jak2	-0,04052	0,117213	-0,46591	0,64974	0,8984516	-7,39
Smad3	-0,04057	-0,00225	-0,58763	0,567826	0,8410075	-7,32228
Rad51	-0,04059	0,041121	-0,38651	0,705979	0,9060647	-7,42603
PDHA1	-0,04079	-0,038326	-0,5757	0,575606	0,8467108	-7,32958
ATR	-0,04326	-0,111535	-0,42547	0,67813	0,8993053	-7,40916
Patched	-0,04336	-0,044418	-0,66118	0,521163	0,8406864	-7,27421
DDB-1	-0,04551	-0,14144	-0,5306	0,60551	0,8787029	-7,35587
HLA-DQA1	-0,04576	-0,058912	-0,44999	0,660853	0,8984516	-7,39775
DJ1	-0,04596	-0,237303	-0,47295	0,644852	0,8984516	-7,38649
CD45	-0,04603	-0,03593	-0,55679	0,588051	0,8620749	-7,34085
TIGAR	-0,04622	-0,036293	-0,70536	0,49424	0,838916	-7,24282
CIITA	-0,04667	-0,028549	-0,72799	0,480781	0,838916	-7,22602
WIPI1	-0,04676	0,170801	-0,81812	0,429454	0,7896418	-7,15431
NQO1	-0,04801	-0,013101	-0,38672	0,705829	0,9060647	-7,42595
MSI2	-0,0481	-0,049706	-0,64548	0,530934	0,8406864	-7,28492
HER2	-0,04935	-0,099699	-0,351	0,731758	0,9194979	-7,44003
HMHA1	-0,04968	-0,095438	-0,62103	0,54636	0,8406864	-7,30111
MSH2	-0,05021	-0,305486	-1,08214	0,30075	0,6647354	-6,90266
AMPKa_pT	-0,05151	0,069402	-0,88103	0,395845	0,7549078	-7,09986
p38-a	-0,05154	-0,037713	-0,99185	0,341123	0,7012504	-6,99544
RIP	-0,0529	0,030057	-0,773	0,45469	0,8097984	-7,19116
ATM_pS19	-0,05387	-0,044366	-0,65984	0,521991	0,8406864	-7,27514
p44-42-MA	-0,05544	-0,015364	-0,47859	0,640954	0,8984516	-7,38365
CD26	-0,05677	-0,017648	-1,023	0,326769	0,6836772	-6,96419
Pdcd4	-0,05707	0,463311	-0,59097	0,565659	0,8406864	-7,32021
C-Raf	-0,05729	-0,023119	-1,03231	0,322566	0,6781759	-6,9547
EGFR_pY11	-0,05841	0,002371	-0,86987	0,401674	0,7595346	-7,10978
p27_pT157	-0,05885	0,027337	-1,11707	0,286136	0,6492664	-6,865
UBAC1	-0,06053	-0,032503	-1,06014	0,310244	0,6761695	-6,92589
BMK1-Erk5	-0,06084	0,018014	-1,03786	0,320079	0,676246	-6,949
DUSP6	-0,06199	0,129979	-1,041	0,31868	0,676246	-6,94577
NDUFB4	-0,06276	-0,047676	-0,72411	0,483069	0,838916	-7,22893
Beclin	-0,0634	0,065552	-1,3817	0,192594	0,5355361	-6,54963
MR1	-0,06499	0,031104	-0,48545	0,636224	0,8984516	-7,38014
TFRC	-0,06518	-0,023369	-0,68724	0,505179	0,8406864	-7,25592
eIF4E_pS20	-0,06587	0,048507	-1,41837	0,18188	0,5226028	-6,50203
FAK	-0,06604	-0,154643	-0,86964	0,401796	0,7595346	-7,10999
UGT1A	-0,067	0,268485	-0,9738	0,349646	0,7108366	-7,01317
MERIT40	-0,06756	-0,004042	-0,70813	0,492579	0,838916	-7,24079
Stat3_pY70	-0,06769	0,23668	-0,75956	0,462383	0,8134175	-7,20176
AceCS1	-0,06829	0,175013	-0,76761	0,457764	0,8119186	-7,19543
Atg4B	-0,06833	0,127254	-1,27786	0,225799	0,5561111	-6,67945

JAB1	-0,13096	0,007015	-1,74349	0,107147	0,20991	-6,45717
Melanoma-gp100	-0,13262	-0,02963	-2,00519	0,068366	0,1517183	-6,04798
UBAC1	-0,13402	-0,032503	-2,34725	0,037173	0,0942433	-5,47311
IGFRb	-0,13476	0,161739	-1,88747	0,083862	0,1746119	-6,23586
AMPKa	-0,13744	0,044799	-2,49132	0,028612	0,0790486	-5,22074
FGF-basic	-0,1388	0,070481	-1,95918	0,074079	0,1583829	-6,12212
Bcl-xL	-0,1428	-0,052409	-3,42397	0,005139	0,0238166	-3,51251
mTOR_pS2448	-0,14429	0,087497	-1,99768	0,06927	0,1521588	-6,06015
EGFR_pY1173	-0,14614	0,002371	-2,17636	0,050528	0,1209862	-5,76516
NDRG1_pT346	-0,14764	0,175484	-1,68748	0,117672	0,223421	-6,54049
Atg7	-0,14892	-0,03646	-1,70957	0,113414	0,2162903	-6,50781
IR-b	-0,14912	-0,023481	-1,3656	0,197461	0,3337486	-6,98436
eIF4G	-0,15161	0,075302	-2,36542	0,03597	0,0922811	-5,44157
ACSL1	-0,15219	0,059466	-2,85931	0,014551	0,0486177	-4,55744
PAK4	-0,15378	0,010678	-3,08881	0,009525	0,0363294	-4,13492
GSK-3a-b	-0,15507	0,145096	-2,83211	0,015301	0,0503401	-4,60717
HLA-DQA1	-0,15593	-0,058912	-1,53343	0,151463	0,2731411	-6,76075
STING	-0,15757	0,244125	-0,69858	0,498317	0,640593	-7,66313
E-Cadherin	-0,15879	-0,718665	-0,35979	0,725345	0,8206834	-7,85238
p27_pT198	-0,1621	-0,028398	-1,80739	0,096174	0,1964504	-6,36017
c-IAP2	-0,16608	-0,079066	-2,51557	0,027372	0,0776151	-5,17778
b-Catenin	-0,16648	-0,058081	-2,96857	0,011893	0,0423626	-4,35686
PI3K-p110-a	-0,1675	0,200034	-2,40674	0,033374	0,0886222	-5,36952
PTPN12	-0,16914	0,448663	-2,9857	0,011523	0,0417335	-4,32532
HMHA1	-0,17056	-0,095438	-2,13223	0,054653	0,1273271	-5,83907
53BP1	-0,17105	0,410082	-1,95516	0,074598	0,1583829	-6,12855
ARID1A	-0,1716	-0,041475	-1,51473	0,15609	0,2755208	-6,78655
WIPI1	-0,17492	0,170801	-3,0604	0,010038	0,0375981	-4,18746
Atg5	-0,17764	0,237612	-2,24504	0,044687	0,1100586	-5,64884
Mitofusin-2	-0,17782	0,07772	-1,8598	0,087944	0,18223	-6,27915
Atg4B	-0,18092	0,127254	-3,38343	0,005536	0,0251152	-3,58793
CD26	-0,18096	-0,017648	-3,26078	0,006936	0,0305055	-3,81598
IDO	-0,18098	-0,045778	-1,56092	0,144877	0,2623608	-6,72244
Raptor	-0,18612	0,029375	-2,88077	0,013986	0,0478425	-4,51815
Paxillin	-0,18614	-0,099849	-2,60589	0,023201	0,0694306	-5,01673
Merlin	-0,18912	-0,057375	-2,20243	0,04823	0,1169655	-5,72118
Gys	-0,19124	0,329948	-2,46735	0,02989	0,0805153	-5,26306
Erk5	-0,19318	-0,046955	-2,55467	0,025484	0,0732234	-5,10826
PYGB	-0,19334	0,004676	-3,3156	0,006271	0,0281526	-3,71409
RSK	-0,19347	-0,552241	-3,24239	0,007175	0,0307184	-3,85016
Slfn11	-0,1971	-0,015227	-1,34841	0,202773	0,3400593	-7,00623
A-Raf	-0,20048	-0,001133	-3,17752	0,008087	0,0328805	-3,97057
SHP2	-0,2016	-0,044912	-2,76312	0,017376	0,0548696	-4,73287
HER3_pY1289	-0,20666	3,22E-04	-3,93653	0,002028	0,0107897	-2,56198
HSP27_pS82	-0,21049	-0,021664	-1,51486	0,156057	0,2755208	-6,78637
B7-H4	-0,21369	-0,039993	-2,49705	0,028314	0,0787308	-5,21059
4E-BP1_pT37_T46	-0,2144	-0,149083	-2,75225	0,017727	0,0553663	-4,75262
NF-kB-p65_pS536	-0,21507	0,118054	-4,07391	0,001587	0,0089997	-2,30944
GSK-3B	-0,21579	0,32798	-2,09856	0,058013	0,1329982	-5,89503
Aurora-ABC_pT288_pT23	-0,21695	0,130098	-2,72428	0,018665	0,0575552	-4,80337
IRS1	-0,21722	0,130579	-2,8659	0,014376	0,0484051	-4,54537
MSH2	-0,21838	-0,305486	-4,70709	5,28E-04	0,0034993	-1,16725
SLC1A5	-0,21948	-0,020076	-1,67974	0,119195	0,2253199	-6,55186
ACC1	-0,21957	-0,14479	-4,01458	0,001764	0,0097457	-2,41834
Tuberin	-0,22095	0,001667	-2,78026	0,016836	0,054558	-4,7017
ER-a	-0,22121	-0,077341	-2,58447	0,024131	0,0708299	-5,05508
DDR1	-0,22555	-0,018116	-3,87368	0,00227	0,0116171	-2,67794
Gys_pS641	-0,23075	0,273752	-2,80554	0,016069	0,052468	-4,65566
Rb_pS807_S811	-0,23533	0,274772	-4,59662	6,37E-04	0,0040988	-1,36353
IGF1R_pY1135_Y1136	-0,23661	-0,141052	-3,83021	0,002455	0,0123014	-2,75826
EMA	-0,24261	0,046986	-1,11883	0,285412	0,430114	-7,27775
ATR	-0,24415	-0,111535	-2,40103	0,033722	0,0886222	-5,3795
Gab2	-0,24626	0,045631	-2,8928	0,013679	0,047166	-4,4961
Akt_pT308	-0,25003	0,127704	-3,61772	0,003607	0,0168977	-3,15222
PARP	-0,25049	-0,075124	-4,78407	4,63E-04	0,0031692	-1,0313
ASNS	-0,25176	-0,022748	-3,78878	0,002645	0,0131036	-2,83492
Akt_pS473	-0,25672	0,399518	-2,57498	0,024554	0,0710245	-5,07203
ZAP-70	-0,26283	-0,001713	-2,37703	0,035222	0,0914502	-5,42138
MCT4	-0,27109	0,021705	-4,12357	0,001453	0,0083507	-2,21851
DDB-1	-0,28114	-0,14144	-3,27753	0,006726	0,0298838	-3,78485
Akt2	-0,28563	0,225659	-4,60106	6,32E-04	0,0040988	-1,35562
XIAP	-0,292	0,151229	-4,4592	8,07E-04	0,0049705	-1,6096
PDK1	-0,29333	-0,003713	-3,23528	0,00727	0,0307184	-3,86335
Smad1	-0,29718	0,042839	-4,88157	3,93E-04	0,0028245	-0,86011
Cox2	-0,29779	0,165493	-1,75637	0,104848	0,2072541	-6,43778
Cyclin-D3	-0,30003	0,043783	-4,04432	0,001673	0,0093629	-2,36373
Rad23A	-0,30217	-0,014213	-1,71281	0,112802	0,2160781	-6,503

Paxillin	-0,07021	-0,099849	-0,9829	0,345331	0,7053908	-7,00427
JAB1	-0,07169	0,007015	-0,95434	0,359005	0,7221004	-7,03197
MAPK_pT2	-0,07337	0,259927	-0,67024	0,515574	0,8406864	-7,26793
Tuberin	-0,07413	0,001667	-0,93279	0,369572	0,7354429	-7,05241
ACC1	-0,07419	-0,14479	-1,35655	0,200244	0,5484457	-6,58176
B7-H3	-0,07435	-0,017587	-1,06426	0,308448	0,6761695	-6,92157
p27_pT198	-0,07463	-0,028398	-0,8321	0,421831	0,7836602	-7,14253
CSK	-0,07506	0,00617	-1,22592	0,24407	0,5811843	-6,74151
UBQLN4	-0,07516	-0,037809	-0,8865	0,393009	0,7549078	-7,09496
p38-MAPK	-0,07553	0,104174	-1,27125	0,228063	0,5584958	-6,68746
eIF4G	-0,07654	0,075302	-1,19416	0,255807	0,6057853	-6,77848
PAK1	-0,07698	0,121987	-1,33781	0,206103	0,5484457	-6,60541
Rab25	-0,0791	0,029024	-0,88615	0,393191	0,7549078	-7,09527
PI3K-p110-	-0,07947	0,200034	-1,14186	0,276096	0,6397701	-6,83768
CDK1_pT14	-0,08039	-0,263332	-1,51417	0,156228	0,502496	-6,37358
b-Catenin	-0,0806	-0,058081	-1,43733	0,176541	0,5186339	-6,47708
Gli1	-0,08148	0,045725	-1,43177	0,178092	0,5186339	-6,48442
DDR1	-0,08456	-0,018116	-1,45234	0,172404	0,5186339	-6,45715
LC3A-B	-0,08521	-0,040287	-1,64471	0,126315	0,4628801	-6,18961
Enolase-2	-0,0863	-7,87E-04	-1,30098	0,218032	0,553027	-6,6512
p38-MAPK-	-0,08633	0,014931	-1,43997	0,175807	0,5186339	-6,47358
Porin	-0,08667	-0,05308	-1,85912	0,088046	0,3783138	-5,86753
TSC1	-0,09035	0,030918	-1,63988	0,127326	0,4628801	-6,1966
eEF2	-0,09043	0,086276	-1,39348	0,189097	0,535152	-6,53444
MEK2	-0,09143	-0,028089	-1,79209	0,098705	0,4013369	-5,97068
PMS2	-0,09192	-0,057549	-1,09079	0,29708	0,6600066	-6,89343
Gli3	-0,09223	2,76E-05	-1,48857	0,162762	0,509804	-6,40847
PAICS	-0,09402	-0,15572	-1,55957	0,145196	0,4944847	-6,31074
HSP27_pS8	-0,09473	-0,021664	-0,68177	0,508509	0,8406864	-7,25982
Wee1	-0,09497	-0,01496	-0,7051	0,494396	0,838916	-7,24301
NDRG1_pT	-0,09594	0,175484	-1,09657	0,294646	0,6600066	-6,88722
Lasu1	-0,09597	-0,040635	-1,82963	0,0926	0,3801027	-5,91318
PYGB	-0,09771	0,004676	-1,67562	0,120014	0,4616099	-6,14464
Bax	-0,09977	-0,033866	-1,91636	0,079786	0,362617	-5,77782
oct-04	-0,09997	-0,096692	-0,35778	0,726812	0,9159527	-7,43746
Granzyme-	-0,10128	0,194614	-1,17624	0,262624	0,615167	-6,799
AMPKa	-0,10175	0,044799	-1,84434	0,090303	0,3783138	-5,89046
SHP2	-0,10227	-0,044912	-1,40168	0,186694	0,5328811	-6,52381
IRS2	-0,10313	0,497198	-1,09538	0,295144	0,6600066	-6,8885
ASNS	-0,10411	-0,022748	-1,56679	0,143505	0,4944847	-6,30062
Rictor_pT1	-0,10416	0,247853	-1,91373	0,08015	0,362617	-5,78198
FoxO3a_pS	-0,10503	0,075277	-1,65761	0,12365	0,4628801	-6,17091
BCL2A1	-0,10513	-0,035601	-1,09228	0,296452	0,6600066	-6,89183
VEGFR-2	-0,10621	0,135944	-1,05567	0,312199	0,6761695	-6,93056
XPF	-0,10753	0,014147	-2,02401	0,06615	0,3277096	-5,60532
Gys	-0,10778	0,329948	-1,39052	0,189973	0,535152	-6,53827
Chk1_pS34	-0,1098	0,094896	-1,65826	0,123518	0,4628801	-6,16997
DRP1	-0,11062	0,073839	-2,29345	0,040963	0,2635093	-5,15494
PKA-a	-0,11139	-0,147499	-1,34574	0,203606	0,5484457	-6,59543
Myosin-IIa_	-0,11771	-0,007141	-1,00337	0,335763	0,6957393	-6,98398
GSK-3a-b	-0,11995	0,145096	-2,19071	0,04925	0,2756727	-5,32951
ERCC1	-0,12051	-0,05099	-2,19778	0,048632	0,2756727	-5,3176
DVL3	-0,12073	0,097953	-2,36785	0,035812	0,2450007	-5,02661
Atg5	-0,12288	0,237612	-1,55304	0,146739	0,4944847	-6,31984
Akt2	-0,12584	0,225659	-2,02706	0,065797	0,3277096	-5,60036
p90RSK_pT	-0,12803	0,127706	-0,7199	0,485567	0,838916	-7,23208
B-Raf_pS4	-0,12818	0,108475	-2,89989	0,013501	0,1452348	-4,07489
MCT4	-0,12951	0,021705	-1,97009	0,072686	0,3442579	-5,69232
Rb_pS807_	-0,13049	0,274772	-2,54875	0,025761	0,1982695	-4,70887
B-Raf	-0,13096	0,001943	-1,52347	0,153913	0,502496	-6,36081
PKM2	-0,1319	-0,316375	-2,05104	0,063084	0,3251399	-5,56129
Raptor	-0,13285	0,029375	-2,05618	0,062517	0,3251399	-5,55289
ACVRL1	-0,133	-2,20E-04	-2,50766	0,027771	0,2099881	-4,7817
PKCa	-0,13419	0,324233	-2,56682	0,024923	0,1982695	-4,67674
RSK	-0,13516	-0,552241	-2,26519	0,0431	0,2645265	-5,20328
ZAP-70	-0,136	-0,001713	-1,22999	0,242596	0,5808818	-6,73672
ACSL1	-0,13646	0,059466	-2,56373	0,025065	0,1982695	-4,68226
p70-S6K1	-0,13687	0,134162	-1,90928	0,080769	0,362617	-5,789
MDM2_pS	-0,13705	0,002366	-1,85463	0,088727	0,3783138	-5,87452
B7-H4	-0,13796	-0,039993	-1,61214	0,133268	0,4786537	-6,23644
ER-a	-0,14017	-0,077341	-1,63761	0,127802	0,4628801	-6,19987
GSK-3a-b_	-0,14136	0,350958	-1,77147	0,10221	0,4078921	-6,00197
HER3_pY12	-0,14265	3,22E-04	-2,71721	0,018909	0,1733991	-4,40703
NF-kB-p65_	-0,14574	0,118054	-2,76059	0,017457	0,1656751	-4,32855
RPA32_pS4	-0,14623	0,003	-2,2038	0,048112	0,2756727	-5,30745
Mitofusin-2	-0,14717	0,07772	-1,53926	0,150045	0,5013137	-6,339
c-IAP2	-0,14783	-0,079066	-2,23909	0,045167	0,2666706	-5,24771

PAICS	-0,30731	-0,15572	-5,0976	2,75E-04	0,0021143	-0,48504
DVL3	-0,31665	0,097953	-6,21043	4,80E-05	5,31E-04	1,34919
mTOR	-0,31753	0,02475	-5,68632	1,07E-04	0,0010014	0,506244
Akt1	-0,32109	-0,036534	-1,77286	0,101971	0,2044161	-6,41284
Stat3_pY705	-0,3245	0,23668	-3,64101	0,003457	0,0163746	-3,10895
ERRalpha	-0,32454	-0,060608	-5,79938	8,97E-05	8,78E-04	0,691262
eEF2K	-0,33215	0,045657	-3,22723	0,007378	0,0308748	-3,87829
MLKL	-0,33376	-0,032043	-2,70875	0,019206	0,0587069	-4,83147
Wee1	-0,33378	-0,01496	-2,47799	0,029316	0,0801925	-5,24429
MITF	-0,33833	0,104632	-6,35878	3,86E-05	4,86E-04	1,580981
RPA32	-0,34532	-0,242082	-5,47934	1,48E-04	0,0012646	0,163012
Jak2	-0,34611	0,117213	-3,97961	0,001877	0,0102422	-2,48265
VAV1	-0,35286	-0,053778	-3,09766	0,00937	0,0363294	-4,11854
p38-MAPK	-0,35463	0,104174	-5,96883	6,91E-05	7,09E-04	0,965284
PTEN	-0,35736	0,327711	-5,061	2,92E-04	0,0021683	-0,54816
GCN5L2	-0,37165	-0,152105	-7,11823	1,31E-05	2,10E-04	2,721295
Tyro3	-0,38247	0,007668	-6,64415	2,55E-05	3,55E-04	2,018466
p70-S6K1	-0,39486	0,134162	-5,50817	1,41E-04	0,0012445	0,211165
Src_pY527	-0,39616	0,028145	-5,38593	1,72E-04	0,0014253	0,006224
Rictor	-0,39956	0,284828	-6,17974	5,03E-05	5,42E-04	1,300876
Heregulin	-0,4105	0,529459	-5,11474	2,67E-04	0,002093	-0,45554
PLC-gamma2_pY759	-0,42675	-0,048337	-4,85309	4,12E-04	0,0029141	-0,91
Akt	-0,4348	-0,027398	-6,82411	1,98E-05	2,88E-04	2,288736
Akt2_pS474	-0,44435	0,456442	-5,5679	1,29E-04	0,0011693	0,310577
Mcl-1	-0,44681	0,126503	-5,88719	7,83E-05	7,85E-04	0,833756
PKA-a	-0,45301	-0,147499	-5,47293	1,50E-04	0,0012646	0,152283
PHLPP	-0,45585	0,138066	-7,96525	4,30E-06	8,25E-05	3,905764
Sox2	-0,46061	0,345513	-7,94658	4,40E-06	8,25E-05	3,880591
GAPDH	-0,46402	-0,093054	-6,51693	3,06E-05	4,13E-04	1,824783
Akt1_pS473	-0,47762	0,132057	-7,75171	5,65E-06	1,02E-04	3,615459
Stat5a	-0,50318	0,376954	-6,37435	3,77E-05	4,86E-04	1,605132
RRM2	-0,52111	0,068136	-5,78366	9,19E-05	8,80E-04	0,665629
b-Actin	-0,5242	-0,123301	-5,56055	1,30E-04	0,0011693	0,298364
Shc_pY317	-0,5344	0,048967	-6,23445	4,64E-05	5,26E-04	1,386934
Stat3	-0,54545	0,455546	-4,81689	4,38E-04	0,003047	-0,97355
JNK_pT183_Y185	-0,5871	0,06726	-6,10202	5,65E-05	5,94E-04	1,177915
FAK_pY397	-0,655	0,127547	-5,15556	2,50E-04	0,0019937	-0,38541
MMP14	-0,68302	-0,048973	-10,5751	2,21E-07	5,96E-06	7,057217
c-Met_pY1234_Y1235	-0,6895	-0,612021	-6,81436	2,00E-05	2,88E-04	2,274211
Src_pY416	-0,71654	0,071301	-6,95875	1,64E-05	2,52E-04	2,488164
HER2_pY1248	-0,72084	0,074269	-5,08225	2,82E-04	0,0021304	-0,5115
HES1	-0,72648	-0,136402	-2,76109	0,017441	0,0548696	-4,73656
FRS2-a_pY196	-0,79217	0,09159	-6,24453	4,57E-05	5,26E-04	1,402745
cdc2_pY15	-0,80946	-0,23913	-11,0781	1,34E-07	3,85E-06	7,591198
Coup-TFII	-0,84072	0,178384	-11,8459	6,44E-08	2,14E-06	8,367896
Chk1	-0,85974	-0,197309	-15,041	4,50E-09	2,16E-07	11,18095
DUSP6	-0,90276	0,129979	-15,1602	4,12E-09	2,16E-07	11,27484
SHP-2_pY542	-0,91189	0,322221	-7,1277	1,30E-05	2,10E-04	2,735042
Rad51	-0,92203	0,041121	-8,78008	1,59E-06	3,26E-05	4,965088
Connexin-43	-1,00379	-0,052007	-18,3876	4,58E-10	3,78E-08	13,57667
Pyk2_pY402	-1,02345	0,131592	-8,81671	1,52E-06	3,26E-05	5,010972
Hif-1-alpha	-1,04734	0,203091	-9,8255	4,87E-07	1,10E-05	6,220818
Snail	-1,38606	0,389254	-18,747	3,67E-10	3,78E-08	13,80784

PKC-b-II_pS	-0,14893	0,022917	-1,32892	0,208933	0,5484457	-6,61656
Rictor	-0,1544	0,284828	-2,38797	0,03453	0,2450007	-4,99167
EMA	-0,15474	0,046986	-0,71361	0,489307	0,838916	-7,23675
IDO	-0,15487	-0,045778	-1,33572	0,206767	0,5484457	-6,60804
ATM	-0,15562	0,084602	-1,48376	0,164015	0,509804	-6,41498
PHLPP	-0,15905	0,138066	-2,77914	0,01687	0,1656751	-4,29492
PAK4	-0,16293	0,010678	-3,27263	0,006787	0,0886378	-3,38785
Akt	-0,16642	-0,027398	-2,61191	0,022946	0,1939187	-4,5963
ATRX	-0,16836	0,050761	-1,53137	0,151966	0,502496	-6,34991
GCLC	-0,1712	-0,02283	-2,37336	0,035457	0,2450007	-5,01705
FOXO3	-0,1723	0,039835	-1,67056	0,121025	0,4616099	-6,15204
PLC-gamma	-0,17834	-0,048337	-2,02811	0,065676	0,3277096	-5,59866
Erk5	-0,18134	-0,046955	-2,39804	0,033905	0,2450007	-4,97413
VASP	-0,18221	0,087681	-2,68052	0,020229	0,1816439	-4,47319
Gab2	-0,18294	0,045631	-2,14896	0,053053	0,2931512	-5,3995
MLKL	-0,18411	-0,032043	-1,4942	0,161307	0,509804	-6,40084
53BP1	-0,18424	0,410082	-2,10585	0,057269	0,3105905	-5,47115
Akt1	-0,18804	-0,036534	-1,03822	0,319917	0,676246	-6,94863
GSK-3B	-0,18819	0,32798	-1,83018	0,092514	0,3801027	-5,91233
Akt1_pS47	-0,18971	0,132057	-3,07886	0,009701	0,1226175	-3,74609
p53	-0,1926	0,265969	-2,25128	0,04419	0,2645265	-5,22698
PRAS40_pT	-0,19377	0,125751	-3,76293	0,002772	0,0522848	-2,47979
FRS2-a_pY	-0,19741	0,09159	-1,55616	0,146001	0,4944847	-6,3155
p70-S6K_p	-0,19983	0,269091	-1,59917	0,136127	0,4848836	-6,25491
GAPDH	-0,20186	-0,093054	-2,83502	0,015218	0,1561705	-4,19331
PDK1	-0,20918	-0,003713	-2,3071	0,039968	0,2610029	-5,13151
PTPN12	-0,2106	0,448663	-3,71753	0,003009	0,0522848	-2,56363
4E-BP1_pT	-0,21502	-0,149083	-2,76025	0,017468	0,1656751	-4,32919
Slfn11	-0,21667	-0,015227	-1,48223	0,164415	0,509804	-6,41704
Akt2_pS47	-0,21976	0,456442	-2,75364	0,017682	0,1656751	-4,34116
JNK_pT183	-0,22435	0,06726	-2,33181	0,038225	0,2534579	-5,08896
Rad23A	-0,22733	-0,014213	-1,28859	0,222167	0,5534901	-6,66638
Src_pY416	-0,22829	0,071301	-2,21701	0,046989	0,2736773	-5,28513
Akt_pT308	-0,23395	0,127704	-3,38497	0,00552	0,0793074	-3,17963
FAK_pY397	-0,23423	0,127547	-1,84365	0,090409	0,3783138	-5,89152
Pyk2_pY40	-0,23779	0,131592	-2,04848	0,063368	0,3251399	-5,56547
Akt_pS473	-0,24707	0,399518	-2,47815	0,029308	0,2177851	-4,83377
Gys_pS641	-0,24799	0,273752	-3,0151	0,010914	0,1237839	-3,86353
ULK1_pS75	-0,25007	0,319938	-4,66656	5,65E-04	0,0203077	-0,84296
mTOR	-0,26135	0,02475	-4,68024	5,52E-04	0,0203077	-0,8188
Src_pY527	-0,26204	0,028145	-3,56249	0,003989	0,0614001	-2,85053
IGF1R_pY1	-0,26236	-0,141052	-4,24708	0,001169	0,0296286	-1,59372
c-Met_pY1	-0,26245	-0,612021	-2,59384	0,02372	0,1965997	-4,6286
PTEN	-0,26701	0,327711	-3,78148	0,00268	0,0522848	-2,44556
mTOR_pS2	-0,2714	0,087497	-3,75756	0,002799	0,0522848	-2,48969
WIPI2	-0,27549	-0,02897	-2,09508	0,058371	0,3105905	-5,48895
SHP-2_pY5	-0,29038	0,322221	-2,26975	0,042748	0,2645265	-5,19549
Shc_pY317	-0,29926	0,048967	-3,49128	0,004543	0,0675184	-2,9825
Stat5a	-0,33847	0,376954	-4,28781	0,001088	0,0293093	-1,52003
HER2_pY12	-0,42823	0,074269	-3,01925	0,01083	0,1237839	-3,8559
S6_pS240_	-0,43847	0,524502	-3,69037	0,003161	0,0523966	-2,61383
S6_pS235_	-0,47719	0,525909	-2,61345	0,022882	0,1939187	-4,59355
JNK2	-0,55213	0,368288	-7,08148	1,38E-05	8,51E-04	3,03862
