# Supplementary material for: Modeling Heterogeneity of Triple‐Negative Breast Cancer Uncovers a Novel Combinatorial Treatment Overcoming Primary Drug Resistance
Source: Adv Sci (Weinh). 2020 Dec 16;8(3):2003049. doi: 10.1002/advs.202003049 (PMC7856896; doi:10.1002/advs.202003049)
Supplement: Supplementary file 10 — Supplemental Table 9 [file ADVS-8-2003049-s010.pdf]

Table S9: In vitro tumorigenic capacity of the *MMTV-R26^{Met}* cell lines, as determined by the tumor sphere assay - Statistical analysis was performed by One-way ANOVA followed by Tukey test.

		Passage 1								Passage 3			
		MGT2		MGT4		MGT11		MGT13		MGT11		MGT13	
		50-100µm	>100 µm	50-100µm	>100 µm	50-100µm	>100 µm	50-100µm	>100 µm	50-100µm	>100 µm	50-100µm	>100 µm
**MGT7**	50-100µm	0.034 (*)		0.086 (ns)		0.005 (**)		0.003 (**)					
	>100 µm		0.116 (ns)		0.116 (ns)		<0.0001 (***)		0.007 (**)				
**MGT2**	50-100µm			0.113 (ns)		0.006 (**)		0.003 (**)					
	>100 µm				>0.999 (ns)		<0.0001 (***)		0.008 (**)				
**MGT4**	50-100µm					0.055 (ns)		0.008 (**)		0.798 (ns)		0.032 (*)	
	>100 µm						<0.0001 (***)		0.008 (**)		0.009 (**)		0.021 (*)
**MGT11**	50-100µm							0.039 (*)				0.019 (*)	
	>100 µm								0.036 (*)				0.198 (ns)
