# Supplementary material for: Modeling Heterogeneity of Triple‐Negative Breast Cancer Uncovers a Novel Combinatorial Treatment Overcoming Primary Drug Resistance
Source: Adv Sci (Weinh). 2020 Dec 16;8(3):2003049. doi: 10.1002/advs.202003049 (PMC7856896; doi:10.1002/advs.202003049)
Supplement: Supplementary file 11 — Supplemental Table 10 [file ADVS-8-2003049-s011.pdf]

Table S10 : Migrating capacity of the *MMTV-R26*^{Met} cell lines - Statistical analysis was performed by One-way ANOVA followed by Tukey test.

	MGT7	MGT2	MGT4	MGT9	MGT11	MGT13
**MGT7**		0.2249 (ns)	0.0178 (*)	0.0002 (***)	<0.0001 (***)	<0.0001 (***)
**MGT2**			0.6052 (ns)	0.0139 (*)	0.0053 (**)	<0.0001 (***)
**MGT4**				0.4207 (ns)	0.2363 (ns)	<0.0001 (***)
**MGT9**					0.9987 (ns)	<0.0001 (***)
**MGT11**						0.0002 (***)
**MGT13**						
