# Supplementary material for: Modeling Heterogeneity of Triple‐Negative Breast Cancer Uncovers a Novel Combinatorial Treatment Overcoming Primary Drug Resistance
Source: Adv Sci (Weinh). 2020 Dec 16;8(3):2003049. doi: 10.1002/advs.202003049 (PMC7856896; doi:10.1002/advs.202003049)
Supplement: Supplementary file 12 — Supplemental Table 11 [file ADVS-8-2003049-s012.pdf]

Table S11: Cell cycle distribution of MGT11 treated cells - Statistical analysis was performed by two-way ANOVA followed by Tukey test.

		A11					Adav					A11 + Adav				
		G0	G1	S	G2	M	G0	G1	S	G2	M	G0	G1	S	G2	M
**no**	G0	ns					ns					ns				
	G1		ns					ns					***			
	S			ns					ns					ns		
	G2				ns					***					***	
	M					ns					ns					ns
**A11**	G0						ns					ns				
	G1							***					*			
	S								ns					ns		
	G2									***					***	
	M										ns					ns
**Adav**	G0											ns				
	G1												ns			
	S													ns		
	G2														ns	
	M															ns
**A11 + Adav**	G0															
	G1															
	S															
	G2															
	M															
