# Supplementary material for: Modeling Heterogeneity of Triple‐Negative Breast Cancer Uncovers a Novel Combinatorial Treatment Overcoming Primary Drug Resistance
Source: Adv Sci (Weinh). 2020 Dec 16;8(3):2003049. doi: 10.1002/advs.202003049 (PMC7856896; doi:10.1002/advs.202003049)
Supplement: Supplementary file 13 — Supplemental Table 12 [file ADVS-8-2003049-s013.pdf]

Table S12: Antibodies used in the study.

Antibody	Company	Reference	Dilution	Used for	TritonX-100 (%) for IF
Estrogen receptor alpha (ERα)	Santa Cruz	sc-8005	1:100	IHC	0.5 %
Progesteron receptor (PR)	Santa Cruz	sc-810	1:200	IHC	0.5 %
Human EGFR2 (HER2)	Cell Signaling	2165	1:200	IHC	0.2 %
MET	Santa Cruz	sc-10	1:50	IHC	0.2 %
Ki67	Cell Signaling	9129	1:400	IHC	0.5 %
Ki67	Dako	M7249	1:100	IF	0.1 %
Krt14	Covalab	Mab71720	1:500	IF	0.5 %
Krt18	Covalab	Mab20042	1:500	IF	0.5 %
human MET	Assay Design	905-076	1:150	IF	0.5 %
$pY_{1234/35}MET$	Cell Signaling	3126L	1:50	IF	0.5 %
pH3 (S10)	Millipore	06-570	1 :500	IF	0.3 %
alpha-Tubulin	Sigma	T5168	1 :5000	IF	0.3 %
Vimentin	Abcam	Ab-8979	1:100	IF	0.5 %
$pS_{139}H2AX$ (γH2AX)	Cell Signaling	9718	1 : 400	IF, WB	0.2 %
Actin	Sigma	A3853	1:6000	WB	
ATM	Cell Signaling	2873	1:1000	WB	
$pS_{1987}ATM$	Invitrogen	PA5-37346	1:1000	WB	
ATR	Cell Signaling	13934	1:1000	WB	
$pS_{428}ATR$	Cell Signaling	2853	1:1000	WB	
$pS_{473}AKT$	Cell Signaling	9271	1:2000	WB	
BCL-XL	Transduction Laboratory	B22620	1:500	WB	
BIM	Santa Cruz	sc-11425	1:1000	WB	
Cleaved-Caspase 3	Cell Signaling	9661	1:1000	WB	
CDC2 (CDK1)	Cell Signaling	28493	1:20000	WB	
$pY_{15}CDC2(CDK1)$	Cell Signaling	4539	1:1000	WB	
$pT_{202}/Y_{204}ERKs$	Cell Signaling	9106	1:10000	WB	
$pY_{627}GAB1$	Cell Signaling	3231	1:2000	WB	
MCL1	Santa Cruz	sc-819	1:1000	WB	
$pS_{217/221}MEK$	Cell Signaling	9121	1:1000	WB	
mouse MET	Santa Cruz	sc-8057	1:500	WB	
MET^{25H2}	Cell Signaling	3127	1:1000	WB	
human MET	Santa Cruz	sc-161	1:1000	WB	

$pY_{1234/35}$MET	Cell Signaling	3126	1:2000	WB	
PARP	Cell Signaling	9546S	1:2000	WB	
P53	Novocastra	CM5	1:1000	WB	
pS_{15}P53	Cell Signaling	9284	1:1000	WB	
RB	Cell Signaling	9313	1:1000	WB	
pS_{795}RB	Abcam	Ab47474	1:1000	WB	
RPA32 (B-4)	Santa Cruz	sc-271578	1:200	WB	
pS_{33}RPA32	Bethyl	A300-246A	1 :5000	WB	
RRM2	abbexa	Abx004031	1 :5000	WB	
pY_{705}STAT3	Cell Signaling	9145S	1:2000	WB	
XIAP	Transduction laboratory	610716	1:3000	WB	
Goat anti-rabbit IgG-peroxidase	Jackson Immuno Research	115-035-144	1:4000	WB	
Goat anti-mouse IgG-peroxidase	Jackson Immuno Research	115-035-146	1:4000	WB	
CD24-BV421 (clone M1/29)	Biolegend	101826	1:400	FACS	
CD29-FITC (clone HMb1-1)	eBioscience	11-0291-82	1:400	FACS	
CD61-biotin (clone 2C9.G3)	eBioscience	13-0611-81	1:400	FACS	
anti-Ki67-APC (clone SolA15)	eBioscience	17-5698-82	1:200	FACS	
Streptavidin, APC-AF750	Invitrogen	SA1027	1:200	FACS	
Annexin V	eBioscience	88-8005-72	1:20	FACS	
