# Supplementary material for: Modeling Heterogeneity of Triple‐Negative Breast Cancer Uncovers a Novel Combinatorial Treatment Overcoming Primary Drug Resistance
Source: Adv Sci (Weinh). 2020 Dec 16;8(3):2003049. doi: 10.1002/advs.202003049 (PMC7856896; doi:10.1002/advs.202003049)
Supplement: Supplementary file 14 — Supplemental Table 13 [file ADVS-8-2003049-s014.pdf]

Table S13: Drugs used for cell viability assays, with the indicated targets and the concentrations used.

Drug	Company	Target	Concentration (µM)
3-Methyladdenine (3MA)	TargetMol	Autophagy inhibitor	1mM
5-Fluorouracil (5-FU)	Targetmol	Thymidylate synthase (nucleotide synthesis)	0.4, 2, 10
A-1155463	Selleckchem/Targetmol	Bcl-xL	0.3, 1, 3, 10
ABT-737	Selleckchem	Bcl-2,Bcl-xL,Bcl-w	1, 3, 10
ABT-199	Selleckchem	Bcl-2	1, 3, 10
Adavosertib	Selleckchem	Wee1	1, 3, 10
Adavosertib(MK-1775)	Targetmol	Wee1	1, 3, 10
AZD6738	Targetmol	ATR	1, 3, 10
CB-839	Selleckchem	Glutaminase	1, 3, 10
Cisplatin	MedChem Express	DNA replication	1, 3, 10
Docetaxel	Targetmol	Beta-tubulin (microtubule inhibitor)	0.4, 2, 10
Doxorubicin	Targetmol	intercalating agent	0.04, 0.2, 1
Erastin	TargetMol	Ferroptosis inducer	0,5 - 1
Ferrostatin-1	TargetMol	Ferroptosis inhibitor	10, 20, 50, 100
Gefitinib	Calbiochem	EGFR	1, 3, 10
JNJ-7706621	Selleckchem	pan-Cdk, Aurora A/B	1, 3, 10
LY294002	Calbiochem	PI3K	1, 3, 10
Necrostatin-1	TargetMol	Necroptosis inhibitor	10, 20, 50, 100
Olaparib	TargetMol	PARP1/PARP2	1, 3, 10
PF-431396	Selleckchem	Pyk2/Fak	1, 3, 10
PHA-665752	Tocris Bioscience	Met	0.3, 1, 3
R547	Selleckchem/Sigma	Cdk1/2/4	1, 3, 10
SB225022	TargetMol	CXCR antagonist	1, 3, 10
Selumetinib	Selleckchem	Mek1/2	1, 3, 10
Sorafenib	Selleckchem	Raf-1, B-Raf, Vegfr2, Pdgfrβ, Flt3,Kit	1, 3, 10
Staurosporine	TargetMol	Apotosis inducer	150nM
WEHI-539	Apexbio	Bcl-xL	1, 3, 10
Z-VAD-FMK	TargetMol	Apoptosis inhibitor	10, 20, 50, 100
