# Supplementary material for: Modeling Heterogeneity of Triple‐Negative Breast Cancer Uncovers a Novel Combinatorial Treatment Overcoming Primary Drug Resistance
Source: Adv Sci (Weinh). 2020 Dec 16;8(3):2003049. doi: 10.1002/advs.202003049 (PMC7856896; doi:10.1002/advs.202003049)
Supplement: Supplementary file 15 — Supplemental Table 14 [file ADVS-8-2003049-s015.pdf]

Table S14: Oligonucleotides used for RT-qPCR experiments.

Oligonucleotide (name)	Sequence (Forward)	Sequence (Reverse)
Met EXT(mouse)	GTTCTGCTTGGCAACGAGAGCT	GGAGAATGCACTGTATTGCGTCG
Met (mouse)	GAATTGTCTGCCATTACAGG	CAGCTCTTACTGTTATTGGCGC
Met (human)	CTGAAGCCGTTTTATGCAGC	GCCACAGGAAAAACCCAAGTAG
Hgf	GTCCTGAAGGCTCAGACTTGGT	CCAGCCGTAAATACTGCAAGTGG
B2M	ACAGTTCCACCCGCCTCACATT	TAGAAAGACCAGTCCTTGCTGAAG
