# Supplementary material for: Modeling Heterogeneity of Triple‐Negative Breast Cancer Uncovers a Novel Combinatorial Treatment Overcoming Primary Drug Resistance
Source: Adv Sci (Weinh). 2020 Dec 16;8(3):2003049. doi: 10.1002/advs.202003049 (PMC7856896; doi:10.1002/advs.202003049)
Supplement: Supplementary file 16 — Supplemental Table 15 [file ADVS-8-2003049-s016.pdf]

Table S15: Proliferation capacity (mitotic index) of the *MMTV-R26^{Met}* cell lines - Statistical analysis was performed by One-way ANOVA followed by Tukey test.

	MGT7	MGT2	MGT4	MGT9	MGT11	MGT13
MGT7		0.549 (ns)	<0.0001 (***)	<0.0001 (***)	<0.0001 (***)	<0.0001 (***)
MGT2			<0.0001 (***)	<0.0001 (***)	<0.0001 (***)	<0.0001 (***)
MGT4				0.8339 (ns)	0.6485 (ns)	0.9936 (ns)
MGT9					0.9996 (ns)	0.9824 (ns)
MGT11						0.9121 (ns)
MGT13						
