# Supplementary material for: Modeling Heterogeneity of Triple‐Negative Breast Cancer Uncovers a Novel Combinatorial Treatment Overcoming Primary Drug Resistance
Source: Adv Sci (Weinh). 2020 Dec 16;8(3):2003049. doi: 10.1002/advs.202003049 (PMC7856896; doi:10.1002/advs.202003049)
Supplement: Supplementary file 9 — Supplemental Table 8 [file ADVS-8-2003049-s009.pdf]

Table S8: Cell cycle distribution of the *MMTV-R26*^{Met} MGT cell lines - Statistical analysis was performed by two-way ANOVA followed by Tukey test.

		MGT2					MGT4					MGT9					MGT11					MGT13				
		G0	G1	S	G2	M	G0	G1	S	G2	M	G0	G1	S	G2	M	G0	G1	S	G2	M	G0	G1	S	G2	M
MGT7	G0	***					***					***					***					***				
	G1		***					***					***					***					***			
	S			*					***					***					***					***		
	G2				ns					***					***					***					***	
	M					ns					ns					ns					ns					ns
MGT2	G0						***					***					***					***				
	G1							*					***					ns					ns			
	S								***					***					***					ns		
	G2									***					ns					***					***	
	M										ns					ns					ns					ns
MGT4	G0											ns					ns					*				
	G1												ns					ns					***			
	S													ns					*					ns		
	G2														*					ns					**	
	M															ns					ns					ns
MGT9	G0																ns					**				
	G1																	***					***			
	S																		ns					**		
	G2																			ns					***	
	M																				ns					ns
MGT11	G0																					***				
	G1																						*			
	S																							***		
	G2																								***	
	M																									ns
MGT13	G0																									
	G1																									
	S																									
	G2																									
	M																									
